# A comparison of methods for training population optimization in genomic selection

**DOI:** 10.1007/s00122-023-04265-6

**Published:** 2023-03-09

**Authors:** Javier Fernández-González, Deniz Akdemir, Julio Isidro y Sánchez

**Affiliations:** 1 Centro de Biotecnologia y Genómica de Plantas (CBGP, UPM-INIA), Universidad Politécnica de Madrid (UPM) - Instituto Nacional de Investigación y Tecnologia Agraria y Alimentaria (INIA), Campus de Montegancedo-UPM, 28223 Madrid, Spain; 2 CIBMTR (Center for International Blood and Marrow Transplant Research), National Marrow Donor Program/Be The Match, Minneapolis, USA

## Abstract

**Key message:**

Maximizing CDmean and Avg_GRM_self were the best criteria for training set optimization. A training set size of 50–55% (targeted) or 65–85% (untargeted) is needed to obtain 95% of the accuracy.

**Abstract:**

With the advent of genomic selection (GS) as a widespread breeding tool, mechanisms to efficiently design an optimal training set for GS models became more relevant, since they allow maximizing the accuracy while minimizing the phenotyping costs. The literature described many training set optimization methods, but there is a lack of a comprehensive comparison among them. This work aimed to provide an extensive benchmark among optimization methods and optimal training set size by testing a wide range of them in seven datasets, six different species, different genetic architectures, population structure, heritabilities, and with several GS models to provide some guidelines about their application in breeding programs. Our results showed that targeted optimization (uses information from the test set) performed better than untargeted (does not use test set data), especially when heritability was low. The mean coefficient of determination was the best targeted method, although it was computationally intensive. Minimizing the average relationship within the training set was the best strategy for untargeted optimization. Regarding the optimal training set size, maximum accuracy was obtained when the training set was the entire candidate set. Nevertheless, a 50–55% of the candidate set was enough to reach 95–100% of the maximum accuracy in the targeted scenario, while we needed a 65–85% for untargeted optimization. Our results also suggested that a diverse training set makes GS robust against population structure, while including clustering information was less effective. The choice of the GS model did not have a significant influence on the prediction accuracies.

**Supplementary Information:**

The online version contains supplementary material available at 10.1007/s00122-023-04265-6.

## Introduction

Genome-wide selection or genomic selection (GS) has become a standard tool in plant and animal breeding since it was proposed by Meuwissen et al. (2001). In GS, breeding values are obtained for quantitative traits based on genome-wide markers by the estimation of marker (or line) effects in a single or two steps (Isidro et al. 2011; Crossa et al. 2017; Karimi et al. 2019). It has been demonstrated that selection of an optimized training set is a critical factor for accurate genomic predictions (Zhong et al. 2009; Lorenz and Smith 2015; Zhang et al. 2019; Akdemir and Isidro-Sánchez 2019).

Training set optimization consists of selecting a **training set** as an optimal subset of the **candidate set**. The candidate set contains all available genotypes, and the genotypes of the candidate set that are not included in the training set belong to the **remaining set**. The optimization aims to maximize the accuracy of the predictions made on a **test set** while minimizing the size of the training set, which reduces phenotyping costs. More details about training set optimization schemes are available in Isidro y Sánchez and Akdemir (2021), Rio et al. (2022).

The training set optimization under the GS framework started with Rincent et al. (2012) who introduced the mean of the coefficient of determination (CDmean) and the mean of the prediction error variance (PEVmean) (Laloë 1993) as two related optimization criteria. These two criteria have been widely used in the literature (Rincent et al. 2012; Isidro et al. 2015; Akdemir et al. 2015; Bustos-Korts et al. 2016; Rincent et al. 2017; Neyhart et al. 2017; Momen and Morota 2018; Akdemir and Isidro-Sánchez 2019; Ou and Liao 2019; Mangin et al. 2019; Guo et al. 2019; Mendonça and Fritsche-Neto 2020; Olatoye et al. 2020; Roth et al. 2020; Sarinelli et al. 2019; Tayeh et al. 2015; Atanda et al. 2021; Ben-Sadoun et al. 2020; Heslot and Feoktistov 2020; Akdemir et al. 2021; Kadam et al. 2021; Rio et al. 2021b), but many other alternatives have been developed, such as uniform sampling (Bustos-Korts et al. 2016), optimal design algorithms such as A-opt and D-opt (Akdemir and Isidro-Sánchez 2019), estimated theoretical accuracy (EthAcc) (Mangin et al. 2019), upper bound of reliability (Karaman et al. 2016; Yu et al. 2020), Rscore (Ou and Liao 2019), algorithms that maximize the relationship between training set and the test set (Rincent et al. 2017; Roth et al. 2020; Atanda et al. 2021), maximization of connectedness and diversity (MaxCD) for hybrid breeding (Guo et al. 2019), stratified sampling (Isidro et al. 2015), fast and unique representative subset selection (FURS) and partitioning around medoids (PAM) (Guo et al. 2019). These optimization methods can be classified based on whether (**targeted**) or not (**untargeted**) they take into consideration the information about the test set while building the training set (Akdemir and Isidro-Sánchez 2019). In the literature, we can find some comparisons among optimization methods. For example, stratified sampling outperformed CDmean and PEVmean under a strong population structure (Isidro et al. 2015). Other criteria such as A-opt, D-opt, and uniform sampling (Akdemir and Isidro-Sánchez 2019; Bustos-Korts et al. 2016) performed similarly to CD and PEVmean. Maximization of connectedness and diversity (Guo et al. 2019) outperformed CD and PEVmean, but it can only perform targeted optimization and is very specific for hybrid breeding. The estimated theoretical accuracy (Mangin et al. 2019) also outperformed CDmean but needed to start the optimization from a close to optimal solution. Other methods, Rscore (Ou and Liao 2019), FURS, PAM, (Guo et al. 2019; Rio et al. 2021b), maximizing the relationship between the training and the test sets (Rincent et al. 2017; Atanda et al. 2021; Roth et al. 2020) usually showed similar results than CDmean and PEVmean. In general, these comparisons had some drawbacks, i.e., (i) optimization methods could not perform under all the training set optimization scenarios i.e., untargeted vs. targeted optimization, (ii) they could not always start optimization from a random solution, (iii) the comparisons between optimization methods did not include the whole set of options under the same dataset conditions, and (iv) each comparison took place in only 1–2 datasets. Once the training set has been built using the different methods proposed in the literature, the breeders must update it with the new information coming from the breeding pipeline. For updating the training population, Neyhart et al. (2017) proposed a different approach to the use of training set optimization algorithms. Neyhart et al. (2017) selected the individuals with top and/or bottom genotypic values and added them to the existing training set. This approach outperformed CDmean and PEVmean, although it is important to note that it is not an optimization problem but a model selection problem, since to be able to compute the genotypic values the phenotypic information must be provided from a pre-existing training set from previous cycles.

In this study, we aim to establish a comprehensive guideline for training set optimization by comparing a wide range of optimization methods under different datasets, genetic architectures, heritability values, and levels of population structure to establish a training set optimization guideline for its implementation in plant breeding programs. In addition, optimization methods are usually employed to optimize the composition of the training set, but they can also be used to optimize its size. Therefore, here we will test the performance of the different methods in the optimization of both the training set size and its composition. It is important to note that in this study we will only address training set optimization when both the training set and the test set belong to the same population. Further research will be needed for the scenario in which the training set and the test set belong to independent populations.

## Materials and methods

### Datasets

Table 1 summarizes the key features of the seven datasets covering 6 species that were used in this study. We used published datasets with contrasting characteristics that were previously studied to evaluate GS models. The number of genotypes in these datasets ranged from 327 to 5014, the number of markers from 4234 to 244781, and the number of environments from 2 to 4. The genotypes were genotyped with RNAseq, genotype by sequencing (GBS), single nucleotide polymorphism arrays, and exome capture sequencing. The raw phenotypic data for some of these datasets was not available. Therefore, we used the genotypic values of the lines for the available traits in the form of best linear unbiased predictors (BLUPs) as the trait values to be predicted. More details about the experiments can be found in the original studies (Table 1). Furthermore, a simulated trait with the heritability value of 0.5 was generated for each dataset using random marker effects and residuals to check if the training set optimization methods performed similarly using the simulated and real traits. A different simulated trait was randomly generated for each iteration of the cross-validation.

**Table 1:** Summary of the datasets used in this project.

Dataset	Traits	# Genotypes	# Markers	Marker Type	# Env	Heritability	Reference
Maize	HT, FT, YLD	391	244,781	RNAseq	2	–	Hirsch et al. (2014), Hansey et al. (2011)
Rice	HT, FT, YLD	327	57,542	GBS	4	0.30–0.35, 0.33–0.44, 0.31–0.32	Spindel et al. (2015)
RicePopStr	HT, FT, FP, PC	357	36,901	SNPchip	2	0.81, 0.73, 0.69, 0.50	Zhao et al. (2011), Guo et al. (2014)
Sorghum	HT, MO, YLD	451	56,299	GBS	2	0.29–0.88, 0.67, 0.26	Fernandes et al. (2018)
Soybean	HT, R8, YLD	5014	4234	SNPchip	2	0.48–0.52, 0.32–0.37, 0.41–0.49	Xavier et al. (2016)
Spruce	HT, DBH, DE	1722	6930	SNPchip	2	0.39–0.57, 0.32–0.39, 0.33–0.34	Beaulieu et al. (2014)
Switchgrass	HT, ST, AN	514	217,150	ECS	2	–	Evans et al. (2018), Lipka et al. (2014)

#Genotypes; the number of lines, #Markers; the number of markers, #Env; the number of environments, GBS; genotyping by sequencing, ECS; exome capture sequencing, HT; plant height, FT; flowering time, YLD; yield, FP; florets per panicle, PC; protein content, MO; moisture content, R8; days to maturity, DBH; diameter at breast height, DE; density, ST; standability, AN; anthesis date. The values in the heritability column (when present) are displayed in the same order as the traits to which they correspond, and they have been taken from the cited papers. For some traits a range of heritabilities is given and they correspond to measurements in different environments or at different times. RicePopStr indicates rice germplasm with a high population structure.

### Training set optimization methods

Table S1 shows a summary of the training set selection methods used in this study and the equations used to implement them. We selected stratified sampling (StratSamp, Isidro et al. (2015)), mean coefficient of determination (CDmean, Laloë (1993), Rincent et al. (2012)), Rscore (Ou and Liao 2019), generalized average genomic relationship (gAvg_GRM, derived from Atanda et al. (2021)) and partitioning around medoids (PAM, Guo et al. (2019)) as the main methods for comparison. It is important to note that all the aforementioned methods except StratSamp and PAM are evaluation criteria for a training set, and they have to be used in combination with a search heuristic that selects which training sets will be evaluated. In this work we used TrainSel (Akdemir et al. 2021), which combines a genetic algorithm and simulated annealing (Table S1). We based our selection of methods on the following criteria, (i) the wide use in the literature (ii) the suggestions for comparisons in other research studies (iii) the complexity of the analysis, and (iv), the ability to produce a comprehensive study without selecting all the possible combinations. In this sense, we omitted methods such as PEVmean because results have demonstrated that it is very similar to CDmean (Neyhart et al. 2017; Akdemir and Isidro-Sánchez 2019; Kadam et al. 2021). We did not include the upper bound of reliability (U) (Karaman et al. 2016) because our preliminary analysis (Fernández González 2021) showed that it i) struggled to reach the performance of CDmean and PEVmean, and ii) the use of the marker data instead of the relationship matrix made it slower than other alternatives. The estimated theoretical accuracy (Mangin et al. 2019), needed an optimal training set as a starting point, so we discarded it in this research study. Tails and Top methods (Neyhart et al. 2017) were not included because they are suited to update the training set, not to design one *de novo* without a preexisting training set from previous generations used to calculate the genomic estimated breeding values (GEBVs) of the candidate set.

Next, we will briefly describe the different methods used in this study (summarized in supplementary materials, Table S1). For more information check the original references:

*Stratified sampling:* this method consists of dividing the candidate set into previously identified clusters and taking a random sample from them. The number of individuals sampled from each cluster is proportional to the total size of the cluster (Isidro et al. 2015). We performed hierarchical clustering using the R function “hclust” with the ward.D2 method. The number of clusters was decided using the information in the literature (Hansey et al. 2011; Spindel et al. 2015; Zhao et al. 2011; Fernandes et al. 2018; Xavier et al. 2016; Beaulieu et al. 2014; Lipka et al. 2014) combined with the tree generated by hierarchical clustering and a visualization of the clusters in the genetic space. We have defined genetic space as a multivariate space in which every genotype of a dataset is characterized by its genome-wide markers encoded in a numeric form. Insight into this space can be obtained through dimensionality reduction. Principal component analysis (PCA) on the multivariate marker data allows summarizing a substantial amount of the existing variance in the genetic space in a new 2-dimensional space consisting of the first 2 principal components (PCs), which can be plotted and interpreted.

*Partitioning around medoids (PAM):* this algorithm is centered around a dissimilarity matrix that contains the pairwise Euclidean distance between all genotypes in the dataset. These distances have been calculated from genome-wide marker data. The candidate set is divided into as many clusters as individuals will be in the optimized training set in such a way that the sum of dissimilarities within the clusters is minimized (see Table S1). The individuals sampled for the training set are the medoids that represent each cluster (Guo et al. 2019).

*Mean coefficient of determination (CDmean):* The coefficient of determination is the expected correlation between the true and predicted genotypic values. For more information, see Laloë (1993) and Rincent et al. (2012). It can be used to measure the suitability of a training set to make predictions over a target population. We set that the target population was the test set for targeted optimization and the remaining set for untargeted optimization. Training set optimization can be performed by maximizing CDmean. The implementation of CDmean we used was described by Rio et al. (2021a) and it can be seen in Table S1, with 1 as the value for the shrinkage parameter ($\lambda$). We further performed two variations of CDmean to take into account the population structure of the datasets (Isidro et al. 2015). Firstly, within cluster CDmean (WIClustCDmean) consists of dividing the dataset into the same clusters used for stratified sampling and performing training set optimization in each of them independently from one another. Then, the optimized training sets for each cluster are taken together to obtain the desired combined training set. Secondly, overall clustered CDmean (OvClustCDmean) is similar to the standard CDmean with the additional constraint that, in the optimized training set, the number of individuals from each cluster is forced to be proportional to the cluster size in the candidate set. For further details, a diagram showing the differences between the CDmean variants can be found in supplementary materials, Figure S1.

*Rscore:* this criterion is derived from Pearson’s correlation between the GEBVs and the phenotype, and it has to be maximized during optimization (see equation at Ou and Liao (2019) or Table S1). A matrix *X* containing the genome-wide marker data (columns) for all individuals in the dataset (rows) is used as input for the calculation of Rscore, and it can be modified to accelerate optimization by replacing the markers in *X* with its principal components (PCs). In this study, we used as many PCs as genotypes were in the dataset. Similarly to CDmean, Rscore evaluates training set that will be used to make predictions over a target population. We considered that the target population was the test set in targeted optimization and the candidate set in untargeted optimization.

*Generalized average genomic relationship (gAvg_GRM):* The calculation of gAvg_GRM relies on a relationship matrix *A* and it has to be maximized during optimization. We propose this criterion to balance the maximization of the relationship between the training set and the test set and the minimization of the relationship within the training set (Pszczola et al. 2012). It is important to note that the maximization of the relationship between the training set and test set is only possible in the targeted scenario. In untargeted optimization, as the marker data for the test set is unavailable, a target population different to the test set such as the candidate set or the remaining set has to be used as a placeholder. In this sense, gAvg_GRM could be expressed as:

1 $$\begin{aligned} \text {gAvg}\_\text{GRM}= & {} a \cdot mean(A\_{TRS;TP}) \nonumber \\{} & {} - b \cdot mean(A\_{TRS;TRS}) \end{aligned}$$

where *TRS* and *TP* are the training set and target population respectively, *A_TRS;TRS* is the relationship matrix for the individuals in the training set, *A_TRS;TP* is a subset of the relationship matrix whose rows and columns correspond to the individuals in the training set and target population respectively and $mean({\cdot })$ indicates that the average of all elements of a matrix is calculated. This metric can be tuned depending on what weight is given to the relationship between the training set and the target population using the pondering parameters *a* and *b* (Table S1). We have tested 3 combinations of these parameters. Firstly, we focused only on the relationship training set–target population ($a = 1, b = 0$). This is the original criterion described by Atanda et al. (2021), called Avg_GRM. Next, we focused on minimizing the relationship within the training set ($a = 0, b = 1$) and we called it Avg_GRM_self. Finally, we tested a balanced approach, Avg_GRM_MinMax, with parameters $a = 1, b = 1$. As explained before, the target population should always be the test set in the targeted scenario, but a different target population has to be used in untargeted optimization. In the untargeted scenario we used the candidate set as target population for Avg_GRM and the remaining set for Avg_GRM_MinMax. The reason behind this difference is that, as the candidate set contains the training set, using it as target population for Avg_GRM_MinMax could result in a lowered penalization for a high relationship between the individuals in the selected training set. It is important to highlight that for Avg_GRM_self, targeted optimization is not possible as the target population is not taken into account in its calculation.

### Models

In this study, we tested three different models. Two of them were additive models: GBLUP (Karimi et al. 2019) is commonly used in GS and assumes that the trait is controlled by many small-effect quantitative trait loci (QTL) distributed throughout the genome. BayesB (Meuwissen et al. 2001) has the prior assumption that the trait is controlled by a few QTL with large effects. The third model can accommodate for interaction effects: RKHS (Gianola and van Kaam 2008) model is similar to GBLUP substituting the additive relationship matrix (*A*) with a kernel matrix (*K*) calculated using a Gaussian kernel. More details about the GS models used can be found in supplementary materials, Note 1.

### Cross-validation scheme

The cross-validation scheme allows to evaluate the performance of the different methods for optimizing the training set composition for different training set sizes. Next, the results will also be used to explore how the training set size could be optimized.

The first step in the cross-validation consisted of randomly splitting each dataset into a candidate set (85% of the dataset) and a test set (the remaining 15%). Next, the training set, which is a subset of the candidate set, was selected through random sampling and training set optimization. Random sampling was used as a baseline to which the different training set optimization methods can be compared. The optimization was carried out for both untargeted (no information about the test set used) and targeted (information about the test set used) optimization when possible and several training set sizes were tested (10, 20, 40, 60, 80 and 100% of the candidate set). Genomic selection models were built upon every training set obtained. GBLUP, BayesB, and RKHS were tested, and all 3 models were trained for every trait in the dataset, every training set optimization method, and every training set size. Finally, model accuracies were calculated as the correlation between the predictions of the model (GEBVs) and the genotypic values in the test set.

The accuracy comparisons and other results were obtained from an average of 40 replications of the cross-validation (CV) scheme.

We applied this CV scheme for all datasets except soybean, since its number of genotypes (5014) was too high and dimensionality reduction was needed to decrease the computational burden. First, for each iteration, we split the dataset into test set (15%) and candidate set (85%). Then, we used untargeted CDMEAN2 to preselect 1000 genotypes which would act as a reduced candidate set. Finally, we proceeded as normal with training set optimization using the reduced candidate set. We used CDMEAN2 for the preselection step because it is a modification of CDmean which can be accelerated by incorporating dimensionality reduction via PCA (more details in Akdemir (2017) and Table S1).

### Area under the curve calculation

The area under the curve (AUC) was used to summarize the results of the cross-validation. It is important to clarify that this AUC is not related to the commonly used area under the receiver operating characteristic (ROC) curve. For each optimization method, model and dataset-trait combination, the accuracy can be plotted against the training set size, as has been done in Fig. 2. AUC is the estimation of the area under a curve that would follow the discrete accuracy values available between training set size $=$ 10% of the candidate set and training set size $=$ 80% of the candidate set. Training set size $=$ 100% of the candidate set is omitted because no optimization can take place for it as the entire candidate set is selected. Equation 2 shows how AUC is calculated:

2 $$\begin{aligned} \text {AUC}= & {} \sum _{n = 1}^{nTRS-1} \Biggl[ {\frac{acc_{n} + acc_{n+1}}{2}}\cdot (size_{n+1} - size_{n}) \end{aligned} \Biggr]$$

where *nTRS* $=$ 5, which is the number of training set sizes considered; *size*_*n*_ is the training set size corresponding to *n* and *acc*_*n*_ is the accuracy obtained for *size*_*n*_. The advantage of using AUC is that it allows summarizing the performance of an optimization method across training set sizes in a single value.

### Optimization of the training set size

The previously explained evaluation criteria used for the optimization of the training set composition were also employed to optimize its size due to their ability to function as an evaluation metric for a given training set. The criteria tested in this role were Avg_GRM_self in the untargeted scenario, Avg_GRM_MinMax for targeted optimization, and CDmean and Rscore in both scenarios. To this end, the first step was evaluating the training set obtained during the cross-validation using the aforementioned criteria. For instance, the value of Avg_GRM_self was calculated for the training set obtained through optimization with Avg_GRM_self for all the tested training set sizes (10, 20, 40, 60, 80 and 100 % of the candidate set) in the 40 iterations of the cross-validation. Next, the value of the evaluation metric was plotted against the training set size (supplementary materials, Figures S25–S30) and the following function was fitted to it:

3 $$\begin{aligned} evaluation\_metric = {{ln(size-d)}\over {m(size-d)^{p}}} + n \end{aligned}$$

where *d*, *m*, *p* and *n* are the parameters used to fit the function to the observed data, *size* is the size of the training set and *ln* is the natural logarithm. Equation 3 was chosen for its ability to fit the 3 types of curve observed (Ratkowsky 1993): rapid growth followed by slower growth ($p < 0.5$), fast growth followed by a plateau ($0.5< p < 1$) and rapid growth followed by a slow decline ($p > 1$) (Figures S25 - S30). Optimization *per se* was only possible in the latter type of curve, as it is the only one with a maximum within the range of tested training set sizes. For the rest, the fitted function was always increasing, which makes actual optimization impossible. Instead, we selected the training set size that would result in an acceptable accuracy loss. To that end, we used the fitted function (Eq. 3) to select the training set sizes for which the evaluation metric reached 95% and 99% of the value it had for the entire candidate set, aiming to find a training set able to generate a GS model with a target accuracy of 95% and 99% of the maximum accuracy obtained when the entire candidate set is the training set. This assumes that the evaluation metric and the actual accuracy are correlated, and this assumption had to be validated using the cross-validation results to interpolate the accuracy that would have been obtained with the selected training set sizes. This analysis was performed in all datasets except for the soybean data, as we do not have the complete information across the entire range of training set sizes due to the preselection step as explained in the cross-validation section.

### Statistical software and hardware used

All calculations were implemented using R programming language version 3.6.1 (R Core Team 2021). The data and code used are available in https://github.com/TheRocinante-lab/Publications/tree/main/2022/Fernandez-Gonzalez_et_al_2022_Comparison. We used TrainSel for training set optimization (Akdemir et al. 2021), rrBLUP (Endelman 2011), BGLR (Pérez and de los Campos 2014) for modelling, agricolae package (de Mendiburu and Yaseen 2020) to calculate AUDPC, and nlsLM in minpack.lm package to fit Eq. 3 to the observed values in the training set size optimization. We ran our analysis in a cluster with Lenovo Think system SD530 compute nodes with the following characteristics: 2 × Intel^®^ Xeon^®^ Gold 6230 20C 2,1 GHz Cache 20 Cores, 12 x 16 GB DDR4 2666 MHz ECC Reg, 2 × ThinkSystem 2.5” Intel S4510 240 GB and RAID 930-8i 2 GB Flash PCIe 12 Gb Adapter.

## Results

### Population structure

**Fig. 1:**
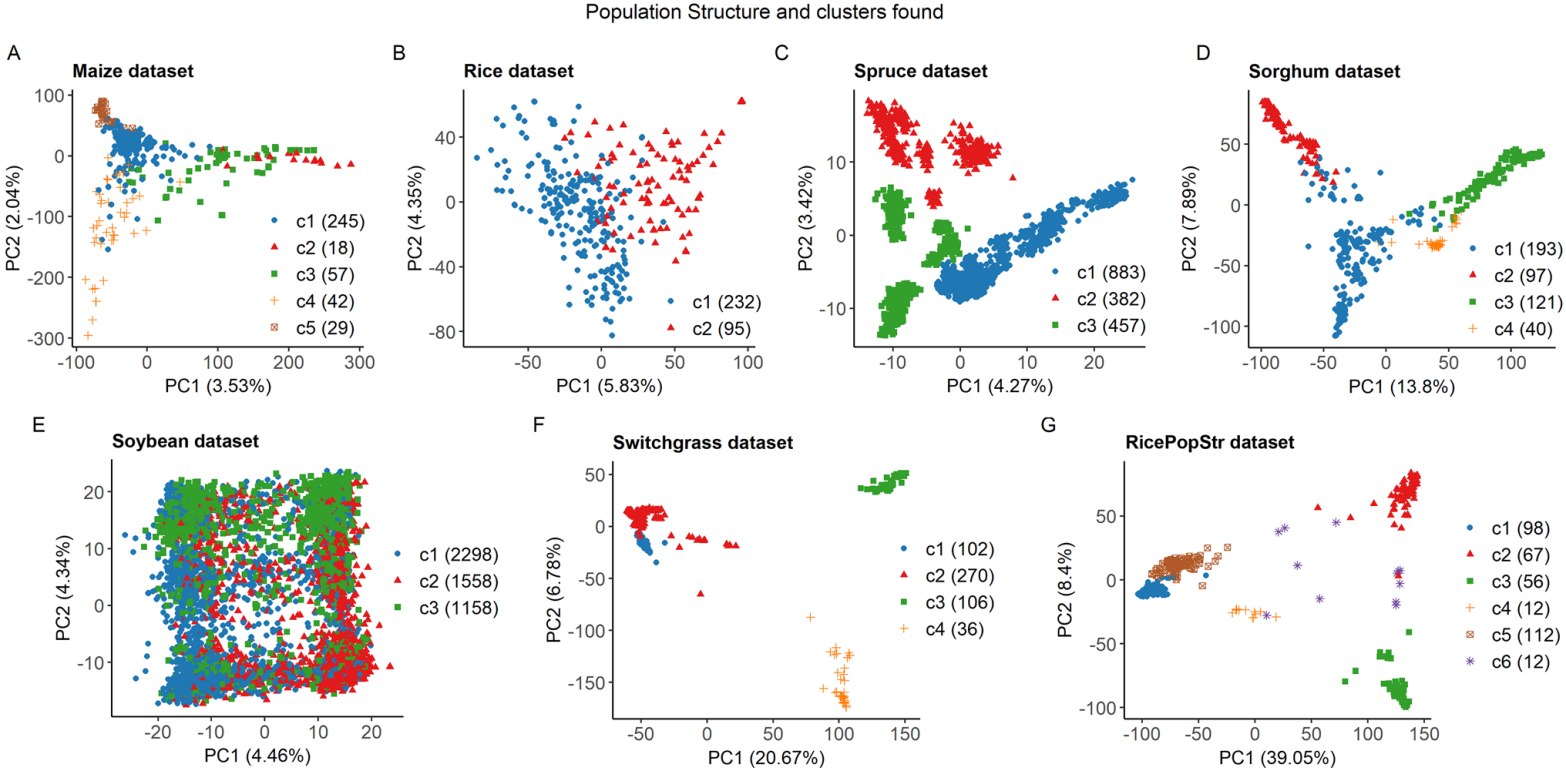
Plots of the first two principal components and the cluster (c) analysis on all datasets. Each solid circle represents a genotype and the colors indicate cluster membership. Number of genotypes per cluster are given by the figure legends in brackets

The population structure and the clustering of the datasets are displayed using the first two principal components (PCs) in Fig. 1. These results indicate that not all datasets presented the same population structure. Maize, rice, soybean, and spruce datasets showed weak population structure, as their first two PCs explained less than 11% of the genetic variability. Sorghum and switchgrass had an intermediate level of population structure, with between 20 and 30% of the genetic variance explained, and ricePopStr had the strongest level of population structure with close to 50% of the genetic variance. Population sizes within clusters varied from 12 in ricePopStr to 2298 in the soybean dataset. The soybean dataset’s shape is because it is derived from 40 biparental families. The spruce dataset showed weak population structure, but individuals could be separated into small subpopulations within clusters (Fig. 1C).

### Model performance

The performances of the different models tested were very similar across datasets and different training sets (see supplementary materials, Table S2). On average, RKHS showed the highest accuracy (0.463) followed by BayesB (0.462) and GBLUP (0.457), although there were no great differences between them. Our results also indicated that the choice of a model barely had any influence on the performance of the training set optimization methods as can be seen in the Tables S32–S52, where the differences in the average performance of the optimization methods across models were hardly ever greater than the standard error of the mean (SEM). As a consequence, we reported here just the simplest model (GBLUP) or the average across models to make comparisons between optimization methods.

### Cross-validation results for the optimization methods

**Fig. 2:**
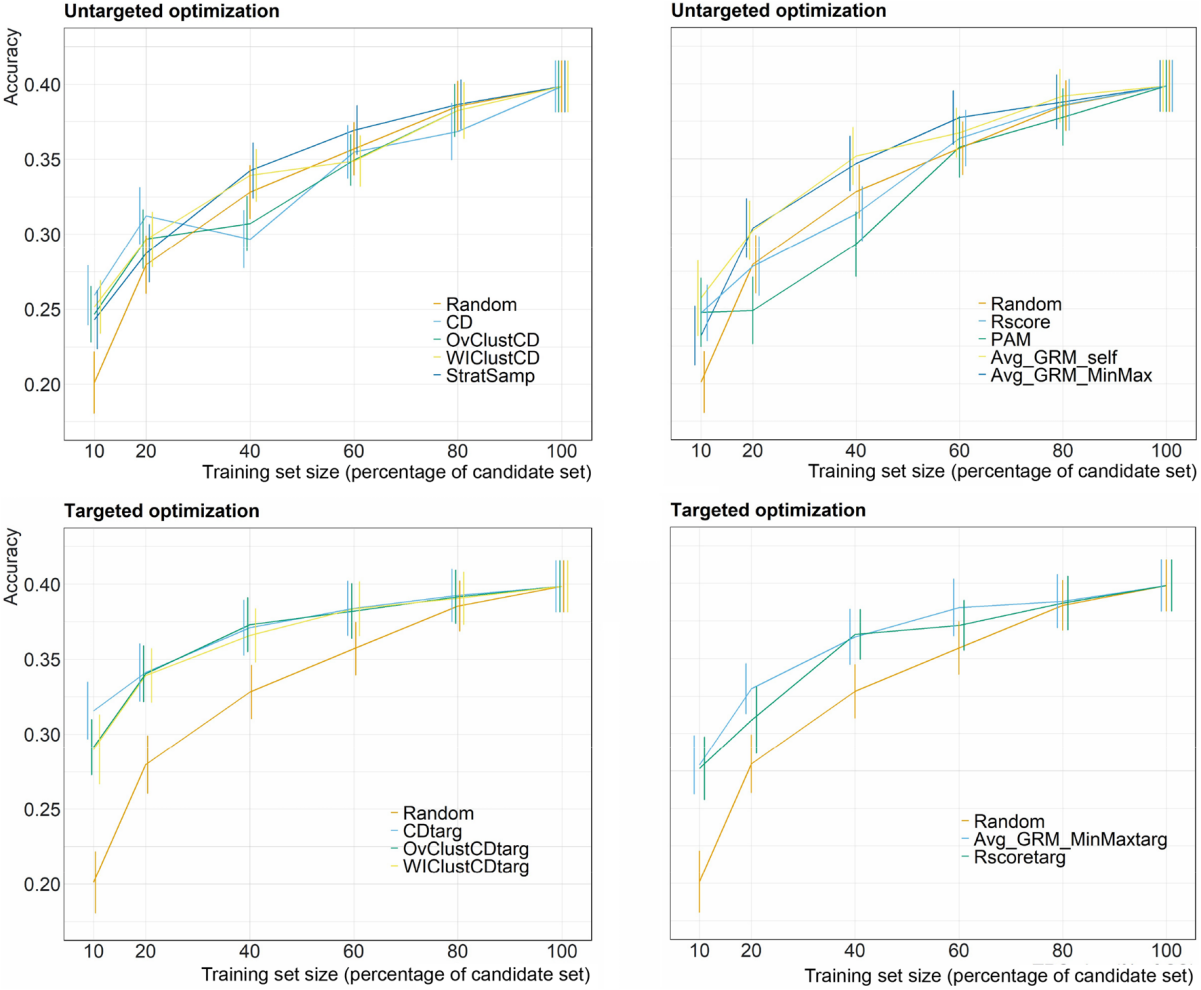
GBLUP prediction accuracies for yield trait in the Sorghum dataset. Each value is the average over the 40 repetitions with their standard errors of the mean. Five different training set sizes 10%, 20%, 40%, 60% and 80% of the total candidate set size were used for the optimization. We also used the 100% of the candidate set in the cross-validation, which does not involve optimization. Avg_GRM is not shown here as its poor performance disrupts the scale of the plot, but it can be found in supplementary materials, Tables S3–S31

Figure 2 shows the comparison of the mean predictive ability for different training set optimization methods across the different training set sizes for yield in the sorghum dataset. The results for all dataset-trait combinations are available in Tables S3–S31. The accuracies in Fig. 2 ranged from 0.20 to 0.39. The smaller the training set sizes, the larger the differences between random sampling and optimization criteria. Maximum accuracies were obtained with the largest training set size, although the differences between the lowest and the largest training set size were less than 16% on average across optimization methods. If we compare training sets of size 80% vs. 40% across optimization criteria, then the differences drop to 5%. As a general trend, optimized samples showed a 6% greater accuracy on average than random sampling. Our results also showed that the best accuracies were reached when the test set information was included in building the training set (Fig. 2). Furthermore, we picked the dataset-trait combination of sorghum and yield in Fig. 2 because it was a good example of the general interactions found between optimization performance and training set size. In general, the relative performance of the tested optimization methods was not consistently affected by the training set size, but there are two exceptions. First, in 3 out of the 29 dataset-trait combinations we observed a drop in accuracy in untargeted CDmean variants for training set size of 40% of the candidate set when compared with their performance for 20% of the candidate set. Two out of the 3 occurrences happened in datasets with intermediate or strong population structure. Secondly, PAM showed great performance for a training set size of 10% of the candidate set followed by a mediocre performance for larger training sets in 10 out of the 29 dataset-trait combinations, 9 of which corresponded to intermediate or strong population structure datasets.

**Fig. 3:**
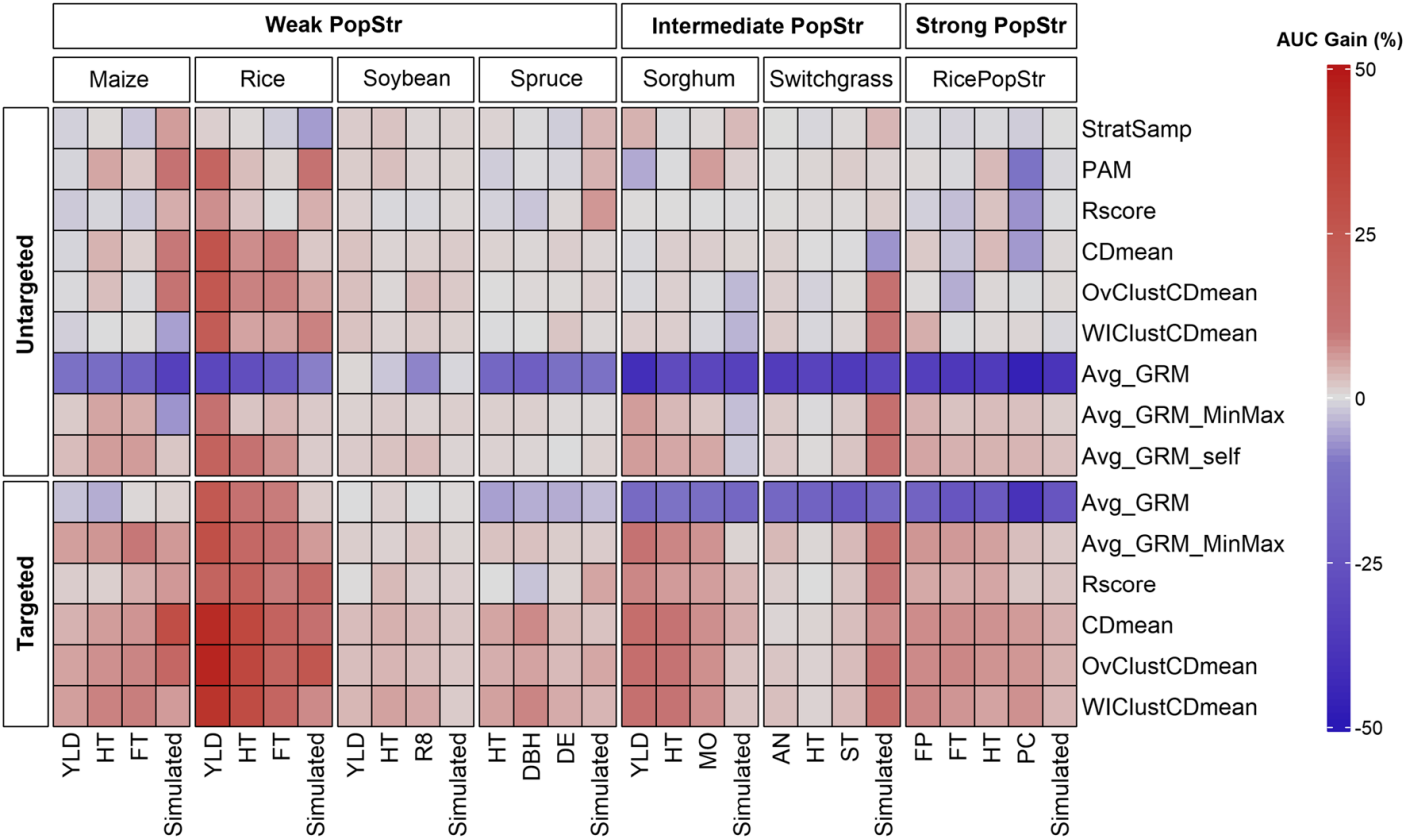
Percentage gain in the area under the curve (AUC) for all optimization methods to random sampling for each dataset-trait combination. The values shown correspond to the average across genomic selection models and the 40 repetitions. Positive values (red) indicate that optimization outperformed random sampling and the opposite is true for negative values (blue). The optimization methods are divided in targeted and untargeted and the datasets are grouped according to their population structure (PopStr) into weak population structure, intermediate population structure and strong population structure. HT; plant height, FT; flowering time, YLD; yield, FP; florets per panicle, PC; protein content, MO; moisture content, R8; days to maturity, DBH; diameter at breast height, DE; density, ST; standability, AN; anthesis date

To simplify the results and to assess the relative performance of the different optimization methods across all training set sizes, we estimated the area under the curve (AUC) for all optimized training set sizes. The percentage of gain in AUC to random sampling for every optimization method in all dataset-trait-model combinations is shown in supplementary materials, Tables S32–S52. We have summarized the AUC results in Fig. 3 and Tables 2 and 3, which are good overviews to explain the general trends on training set optimization.

#### Targeted versus untargeted optimization

Our results indicated that the use of the test set while building the training set resulted in better performance than untargeted optimization (Table 2). This table contains the average percentage of gain in AUC to random sampling across optimization methods for each dataset-trait combination. We can determine from it that, if both optimization approaches (targeted and untargeted) are considered simultaneously, performing optimization yielded on average a 4.93% greater AUC than random sampling. If only targeted methods are considered, the average gain in performance was approximately a 4-fold increase compared to untargeted (7.91% vs. 1.95%). The superiority of targeted vs. untargeted, was observed for every dataset-trait combination (Table 2).

In addition, we compared the individual methods within targeted and untargeted scenarios in Fig. 3 and Table 3. Targeted optimization methods performed better than their untargeted counterparts almost universally across datasets, traits, and models. Both targeted and untargeted methods usually outperformed random sampling, but targeted ones did it by a wider margin than untargeted ones. For instance, for yield in sorghum dataset and GBLUP model (Table S44), untargeted CDmean was a 0.85% worse than random (non-significant difference) while targeted CDmean was a 14.08% better. Rscore went from a gain of 0.47% with untargeted optimization to 8.47% with targeted, Avg_GRM went from −42.01% to −15.25% and Avg_GRM_MinMax went from 6.05% to 11.16%.

**Table 2:** Average performance (measured as the percentage of gain in AUC compared to random sampling) across training set optimization methods for all dataset-trait combinations in both targeted and untargeted optimization.

Percentage of gain in AUC overview									
		Dataset							
		Weak PopStr				Intermediate PopStr		Strong PopStr	
Optimization scenario	Trait	Maize	Rice	Soybean	Spruce	Sorghum	Switchgrass	RicePopStr	Trait Average
Untargeted	HT	2.88	5.12	1.40	0.20	1.52	−0.12	2.10	1.87
	YLD	−0.15	16.52	1.85		1.52			4.93
	FT	1.24	4.22					−0.58	1.63
	R8			1.39					1.39
	DBH				0.05				0.05
	DE				0.38				0.38
	MO					1.74			1.74
	AN						1.07		1.07
	ST						0.91		0.91
	FP							1.86	1.86
	PC							−2.29	−2.29
	Simulated	3.93	3.54	1.17	2.30	-0.82	5.60	0.50	2.32
	Dataset average (non-simulated traits)	1.32	8.62	1.55	0.21	1.59	0.62	0.27	1.95
Targeted	HT	6.01	26.54	3.59	3.57	9.17	0.69	6.07	7.95
	YLD	4.56	35.92	2.24		11.53			13.56
	FT	7.72	15.39					6.72	9.94
	R8			2.96					2.96
	DBH				4.38				4.38
	DE				2.73				2.73
	MO					7.02			7.02
	AN						2.12		2.12
	ST						2.93		2.93
	FP							7.09	7.09
	PC							5.03	5.03
	Simulated	13.16	13.40	1.62	3.65	2.70	11.48	3.22	7.03
	Dataset average (non-simulated traits)	6.10	25.95	2.93	3.56	9.24	1.91	6.23	7.91
	Global dataset average (non-simulated traits)	3.71	17.29	2.24	1.88	5.42	1.27	3.25	4.93

Avg_GRM was not taken into account for the calculation of the average as its low performance was considered an outlier. The datasets are grouped according to their level of population structure (PopStr) into weak population structure, intermediate population structure, and strong population structure. The average for each dataset across all non-simulated traits is also displayed both within targeted and untargeted optimization and globally. It is important to note that in the intersections between the dataset averages and the trait average the value displayed is the mean across all datasets and non-simulated traits. HT; plant height, FT; flowering time, YLD; yield, FP; florets per panicle, PC; protein content, MO; moisture content, R8; days to maturity, DBH; diameter at breast height, DE; density, ST; standability, AN; anthesis date.

**Table 3:** Qualitative overview of the performance of the training set optimization methods across datasets

Qualitative overview of the algorithm performance									
Optimization scenario	Optimization algorithm	Dataset							
		Weak PopStr				Intermediate PopStr		Strong PopStr	
		Maize	Rice	Soybean	Spruce	Sorghum	Switchgrass	RicePopStr	Total
Untargeted optimization	StratSamp	ns	ns	+	ns	+	ns	ns	+2
	CDmean	+	++	+	+	ns	+	+-	+6
	OvClustCDmean	ns	++	+	ns	ns	+	–	+3
	WICIustCDmean	ns	++	+	+	ns	+	+	+6
	Rscore	ns	ns	+	+	ns	ns	+-	+2
	Avg_GRM	–	–	–	–	–	–	–	−18
	Avg_GRM_MinMax	+	+	+	ns	++	+	++	+8
	Avg_GRM_self	++	++	++	ns	++	+	+++	+12
	PAM	+	+	+	ns	+	+-	+-	+4
	Untargeted Total	+2	+8	+8	0	+3	+2	+2	+25
Targeted optimization	CDmean	+++	++	+++	++	++	+	+++	+16
	OvClustCDmean	++	+++	+++	++	++	+	+++	+16
	WICIustCDmean	++	++	+++	++	++	+	+++	+15
	Rscore	+	+++	+	ns	++	+	+	+9
	Avg_GRM	ns	++	ns	–	–	–	–	−8
	Avg_GRM_MinMax	++	++	+	+	++	++	+	+11
	Targeted Total	+10	+14	+11	+5	+8	+3	+8	+59
	Global Total	+12	+22	+19	+5	+11	+5	+10	+84

A sign “$+$” indicates that the optimization led to a significantly larger area under the curve (AUC) than random sampling. If the optimized training set performed worse than a randomly sampled one we used “−”. If no significant differences were found, “ns” is displayed. A single sign in a cell indicates that the difference occurred in at least one trait for at least one model. Two signs mean that the difference happened in all non-simulated traits for at least one model. Three signs mean that the difference happens in all traits for at least one model. The “$+-$” sign means that the optimization was significantly better than random for some traits and worse for other traits. The sum of each row is shown in the last column. We assigned a value of 1 to each “$+$” and $-1$ to each “−”. We calculated the sum of each column similarly within untargeted optimization (Untargeted Total), within targeted optimization (Targeted Total), and globally (Global Total).

#### Dataset and trait role on training set optimization

The dataset had a key impact on the effectiveness of the training set optimization. In all datasets, optimization outperformed random sampling, although the margin by which they did it varied widely (Table 2). Rice and switchgrass datasets showed the greatest and lowest performance when considering the overall average training set optimization performance, with values of 17.29% and 1.27%, respectively (bottom row of Table 2). Sorghum showed an average increase of 5.42%, maize and ricePopStr presented between 3 and 4% of increase, and soybean and spruce had around 2%.

No pattern for the effect of the trait on the average performance of training set optimization could be found. Optimization with respect to the trait architecture was not consistent across all datasets. For instance, yield showed the highest increase in accuracy in the rice dataset optimization, but not in maize, sorghum, and soybean datasets for both untargeted and targeted scenarios (Table 2). Our results showed that the average performance for each trait across datasets was more influenced by the datasets in which each trait belongs than by the genetic architecture of the trait. For example, Table 2 shows that the anthesis date trait was only present in the switchgrass dataset and had the lowest average in targeted optimization (2.12%). This dataset had the worst average performance of training set optimization (1.91%). Nevertheless, within switchgrass, the anthesis date showed an average performance of optimization over 3 times larger than plant height (2.12% and 0.69% respectively). However, because plant height was present in all datasets, it showed a larger average performance (7.95%) with respect to the anthesis date.

The heritability of the trait (Table 1) did not have a consistent effect on the average optimization performance (Table 2). In rice and sorghum datasets the performance of optimization was larger for traits with lower heritability such as yield. However, the opposite was also true for ricePopStr dataset, and we did not find a clear influence of the trait heritability for the soybean or the spruce datasets.

The simulated trait did not show consistency in performance across datasets and optimization scenarios. For instance, simulated traits presented on average higher gain than non-simulated traits for untargeted (2.32% vs. 1.95%) but not for targeted (7.03% vs. 7.91%) optimization (Table 2). Its effect on performance was not consistent across datasets either. It showed greater performance than the average of the real traits in maize, spruce, and switchgrass and worse performance in rice, soybean and sorghum for both targeted and untargeted optimization. For ricePopStr dataset, it reached higher performance than the empirical traits for untargeted optimization and lower performance for targeted optimization (Table 2). It is important to note that, as a different simulated trait was generated for each cross-validation repetition, its average performance showed a higher standard error of the mean than non-simulated traits (Tables S32–S52).

Further results concerning the effect of the population structure of the dataset and the heritability of the trait on the average performance of individual optimization methods can be found in supplementary materials, Figures S23, S24. As a general trend, most targeted methods were robust to changes in population structure and increased their performance as heritability decreased while the opposite happened in the untargeted scenario.

#### Prediction accuracy comparison among optimization methods

The qualitative overview of the performance of the training set optimization methods across datasets in Table 3 and the quantitative summary in Fig. 3 allow us to easily detect the trends related to the relative performance of the methods within both optimization scenarios:

*Targeted optimization:* CDmean was the criterion that outperformed random sampling most frequently, with a total value of +16 in Table 3. There were no clear differences between CDmean and its clustered variants, as OvClustCDmean had a total of +16 and WIClustCDmean had +15 in Table 3 and showed small and inconsistent differences in Fig. 3. The highest percentage of gain in AUC reached by any method corresponds to the targeted OvClustCDmean for yield in the rice dataset, reaching 49.28%. It is closely followed by CDmean at 47.07% and WIClustCDmean at 43.41% (Table S35). It is important to note that OvClustCDmean was not consistently better than the other CDmean variants across dataset and traits. The next best criterion was Avg_GRM_MinMax, with a total score of +11 in Table 3, followed by Rscore, with +9. Finally, Avg_GRM was usually worse than random sampling, as indicated by its negative total value of -8 (Table 3). The relative performance of targeted optimization methods was very consistent across datasets and traits with the exception of Avg_GRM. This criterion, despite its usual poor performance, outperformed random sampling for rice dataset and was similar to it for maize and soybean (Table 3, Fig. 3). Its low performance was found specially in intermediate-strong population structure datasets (sorghum, switchgrass, ricePopStr).

*Untargeted optimization:* the criterion that showed the best performance in untargeted optimization was Avg_GRM_self, with a total score in Table 3 of +12. It is followed by Avg_GRM_MinMax (+8), CDmean variants with a total value ranging between +3 and +6, PAM (+4), and Rscore and StratSamp with +2. Avg_GRM showed the worst performance against random sampling in all datasets (-18). The high total score of Avg_GRM_self in Table 3 occurred because it was very consistent across datasets and traits regardless of population structure (Fig. 3), only failing to outperform random sampling in the spruce dataset, which was the worst dataset for untargeted optimization with a total score of 0 (Table 3). CDmean reached the highest performance obtained by an untargeted method in any dataset-trait combination for yield in the rice dataset (27.59% vs 18.88% for Avg_GRM_self, see Table S35), but it was inconsistent across datasets and traits, being similar or worse than Avg_GRM_self in almost all circumstances outside the rice dataset (Fig. 3). It also presented a poor performance when population structure was intermediate or strong (Table 3).

### Optimization selection strategy and computational time

**Fig. 4:**
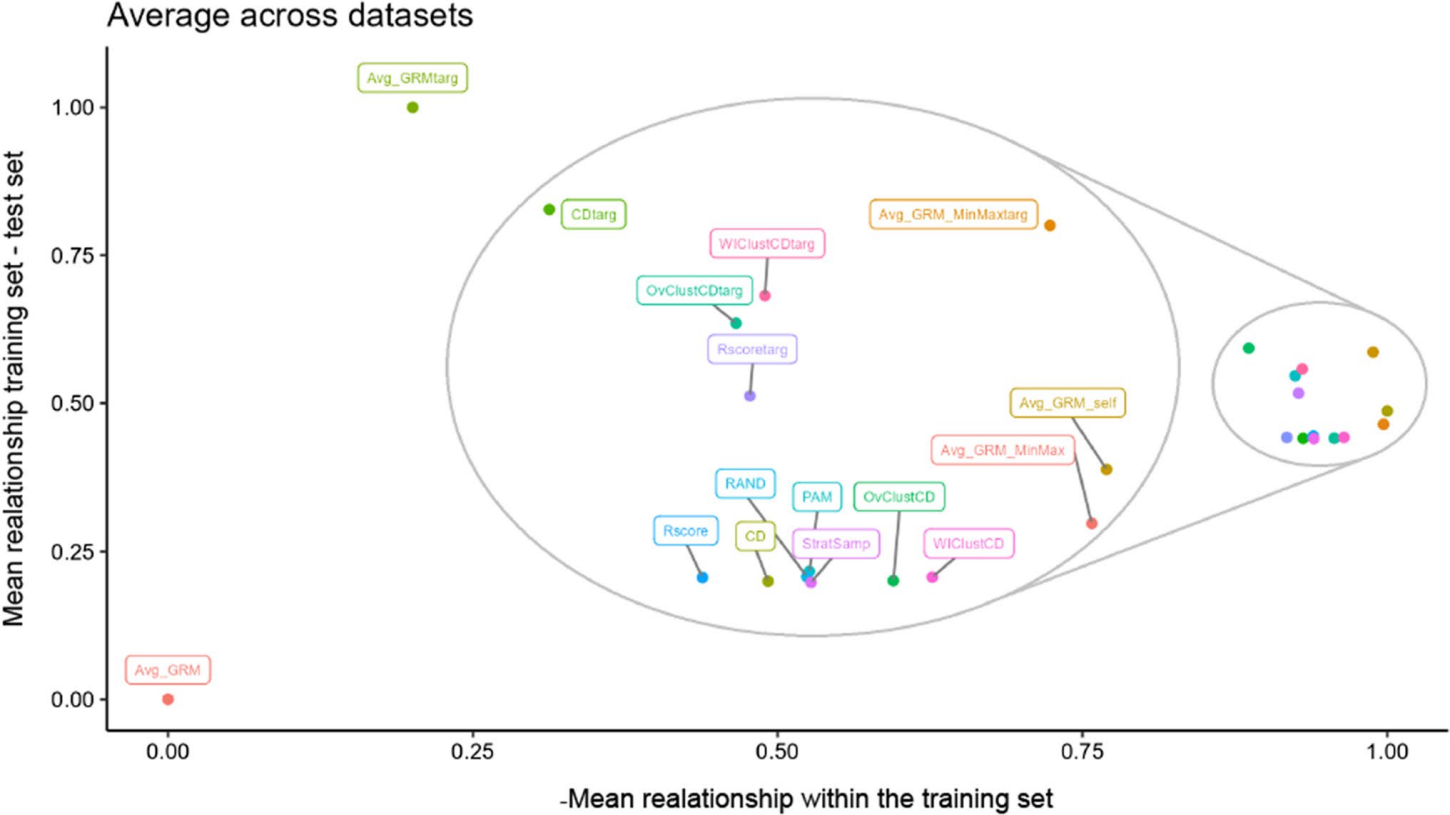
Relative position of the training sets obtained by the different optimization methods when comparing the trade-off in the maximization of the average relationship between the training set and the test set and the minimization of the relationship within the training set (maximization of the negative) for all optimization methods. Zoom has been made on the group of methods on the right of the plot to better appreciate their relative positions. The addition of “targ” at the end of the name of a method indicates that targeted optimization was performed. Otherwise, it corresponds to the untargeted scenario. For each method, the average across the 40 repetitions in all dataset-trait combinations is shown. The average values within each dataset were scaled and centered before combining them and they were scaled again between 0 and 1 afterward. Targeted optimization always showed a higher relationship training set–test set than untargeted and presented a comparable relationship within the training set

In this section, we explore the existing trade-off in training set optimization between maximizing the relationship training set–test set and minimizing the relationship of individuals within the training set (Fig. 4). In this sense, we assumed that training set optimization can be considered a Pareto optimization with an unknown Pareto front (for more details, see Pszczola et al. (2012); Isidro y Sánchez and Akdemir (2021)). The Pareto front contains a series of nondominated solutions (i.e., solutions in which it is impossible to improve one of the criteria without deteriorating the other) and all possible combinations of individuals in the training set would have to be tested to find them with certainty, which is not viable. Despite the absence of the Pareto front, Fig. 4 allows us to explore the relative position of the training sets obtained by the different optimization methods in the aforementioned trade-off. Targeted optimization always showed a higher relationship training set–test set than untargeted and presented a similar relationship within the training set. Among the targeted methods, the highest relationship training set–test set was achieved by Avg_GRM, followed by CDmean and Avg_GRM_MinMax, WIClustCDmean, OvClustCDmean, and Rscore. Avg_GRM_MinMax had the most diverse training set, followed by WIClustCDmean, OvClustCDmean, Rscore, CDmean, and Avg_GRM. In the untargeted scenario, Avg_GRM_self presented the highest relationship training set–test set and the smallest relationship within the training set. Avg_GRM_MinMax was very close to Avg_GRM_self and Avg_GRM had the lowest relationship training set–test set and the highest relationship within the training set. The other methods presented a similar level of relationship training set–test set and varying degrees of relationship within the training set. Among them, WIClustCDmean and OvClustCDmean had the most varied training sets, followed by stratified sampling, CDmean, random sampling, PAM, and Rscore. It is important to note that in both optimization scenarios (targeted and untargeted), Avg_GRM showed an extremely high relationship within the training set and, as a result, it is far from the other optimization methods in Fig. 4.

More details about how each optimization method samples individuals from the genetic space to include them in the training set can be found in supplementary materials, Figures S2–S22. As a general trend, it can be observed that, in the untargeted scenario, CDmean and Rscore to a lesser extent sampled many individuals from the edge of the genetic space for small training set sizes and focused on the center when the training set size was equal to or larger than 40% of the candidate set. On the contrary, in the targeted scenario, both methods made an even sampling of the genetic space regardless of the training set size. PAM tended to sample with high-frequency isolated individuals and Avg_GRM only focused on a small part of the genetic space, while the other optimization methods made an even sampling of the genetic space for both optimization scenarios.

The computational time for the different methods was analyzed and the results are shown in Figure S53. The slowest ones were Rscore and CDmean, with all variants of Avg_GRM and PAM being one and three orders of magnitude faster respectively for small datasets. The time needed for CDmean, Rscore, and PAM escalated in a cubic way with the dimensions of the dataset while Avg_GRM variants escalated in a quadratic way (Figure S53). Finally, stratified sampling would be close to instant for any dataset size.

### Optimization of the training set size

**Fig. 5:**
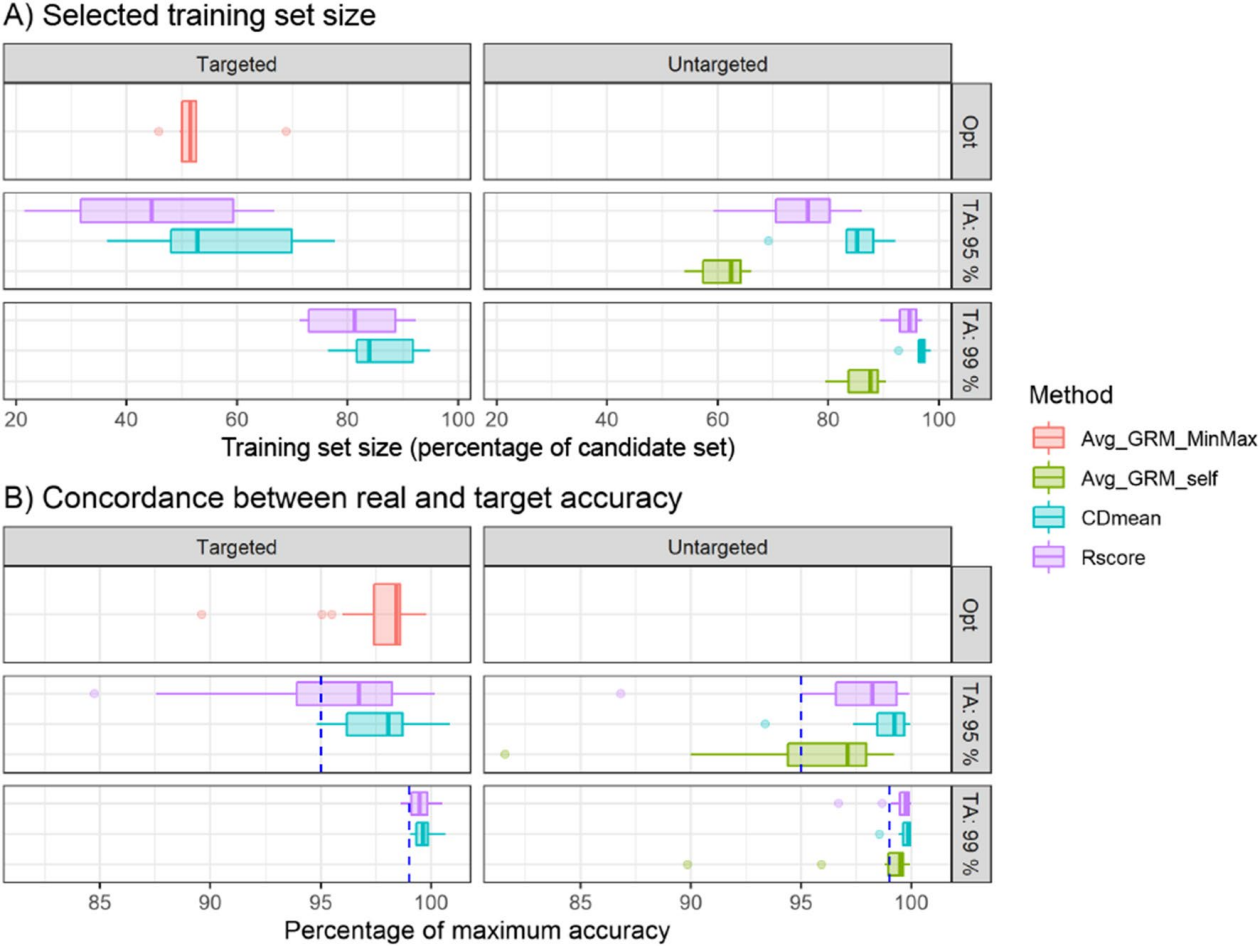
Boxplots showing (**A**) the optimal training set sizes selected by the different evaluation criteria in the tested datasets (all except soybean) and (**B**) the accuracy loss that would result from using the optimal training set size with GBLUP model in the assessed dataset-trait combinations (all non-simulated traits in all datasets except soybean). The training set size is expressed as a percentage of the candidate set and the accuracy is expressed as a percentage of the maximum accuracy obtained when the training set is the entire candidate set. The boxplots on the left correspond to targeted optimization and the ones on the right correspond to untargeted. The evaluation criteria were tested in 3 different scenarios: Optimal (Opt) which is only possible in Avg_GRM_MinMax as it is the only evaluation criteria that presents a maximum within the range of training set sizes tested; target accuracy (TA) = 95% and target accuracy = 99%, which correspond to the training set sizes that result in the evaluation metric being 95% and 99% of the value it reaches when the training set is the entire candidate set respectively. The dotted lines in (**B**) correspond to the target accuracy. The displayed accuracies are average values across iterations and are calculated by interpolation from the accuracy obtained in the tested training set sizes with the best training set optimization algorithm for each dataset (CDmean for targeted scenario and Rice and Spruce in untargeted; Avg_GRM_self in the rest)

Figure 5 shows the validation of the optimal training set sizes selected by the different evaluation criteria by interpolating the GBLUP accuracies obtained in the cross-validation. The interpolation was performed over the accuracies corresponding to the best method of optimization of the training set composition in each dataset (CDmean for targeted scenario and Rice and Spruce in untargeted; Avg_GRM_self in the rest). Our results showed that a larger reduction in the training set size is observed when using targeted vs. untargeted optimization without causing a substantial drop in accuracy (Fig. 5A). In most dataset-trait combinations, the training set size of 50–55% selected by targeted Avg_GRM_MinMax resulted in a small accuracy loss of less than 5%, with only one outlier reaching 10% (Fig. 5B). In the untargeted scenario training set sizes ranging between 65 and 85% are needed for similar drops in accuracies. All optimization criteria that rely on a target accuracy tended to select training set sizes larger than needed to reach the selected target accuracy of 95 or 99% of the maximum, with Avg_GRM_self selecting the smallest training sets and being the closest to the target, followed by Rscore and CDmean. For instance, (Fig. 5A) shows that the training set sizes selected by Avg_GRM_self for a target accuracy of 99% are similar to the ones obtained with CDmean for a target accuracy of 95%. However, the 3 criteria presented a large variation and for some dataset-trait combinations, the obtained accuracy was lower than the target. For example, when target accuracy was 95%, the actual accuracies resulting from the training set sizes selected by Avg_GRM_self ranged between 82% and 99%, with 68% of the dataset-trait combinations reaching an accuracy larger than the target accuracy of 95% (Fig. 5 B). In general, a larger variance was observed for evaluation metrics with a lower bias in the estimation of the target accuracy, with the variance being larger for smaller values of target accuracy.

## Discussion

The goal of our work was to develop guidelines and establish a benchmark of a wide range of training set optimization methods under different population structures, genetic architectures, and heritability values. According to the literature, an ideal training set optimization method should (i) be able to build a training set that maximizes the accuracy of the GS model, by maximizing the relationship between the training set and the test set, and by minimizing the relationship within the training set to capture as much genetic variance as possible. (Pszczola et al. 2012; Isidro y Sánchez and Akdemir 2021), (ii) reduce the computational burden and iii) be easy to implement. For instance, CDmean should be preferable over clustered CDmean if both of them perform equally. Our results, in concordance with many papers describing training set optimization (see Isidro y Sánchez and Akdemir (2021) review), showed that the building of the training set has a very significant impact to increase the accuracy of GS models relative to random sampling (Fig. 3).

### Optimization of the composition of the training set: untargeted scenario

Previous reports (Pszczola et al. 2012; Clark et al. 2012) showed that to optimally design a training set, we should minimize the relationship within the training set but maximize the relationship between training set and test set. The latter is not possible in the untargeted scenario as the test set is unknown. The algorithm Avg_GRM_self, which focused on minimizing the relationship within the training set, outperformed all the untargeted methods tested in most situations, including the previously suggested algorithm Avg_GRM (Fig. 3, Table 3). This indicates that maximizing the genetic variance in the training set seems to play a more important role than maximizing the relationship between the training set and the target population. As in untargeted optimization the target population is not the test set, Avg_GRM unsurprisingly yielded very poor results, generating a training set with low diversity and low relationship with the test set (Fig. 4), which lead to a poor sampling of the genetic space (Figures S2–S22), and consequently performed significantly worse than random sampling (Fig. 3, Table 3).

To balance both Avg_GRM_self and Avg_GRM approaches, we developed the Avg_GRM_MinMax criterion. This algorithm performed well but it never exceeded Avg_GRM_self in non-simulated traits. Our results strongly suggest that optimization that does not take into account the target population, such as Avg_GRM_self, is the best strategy under untargeted optimization scenarios. For instance, Avg_GRM_self usually outperformed CDmean for all datasets-traits combinations except for rice and spruce and it was more robust than CDmean to population structure (Fig. 3, Table 3). Figure 4 also supports the superiority of Avg_GRM_self, as it generates training sets that are more diverse and with a higher relationship with the test set than the other untargeted methods. The reason for the high relationship training set–test set achieved by Avg_GRM_self is probably due to the random selection of the test sets, which leads on average to an even coverage of the genetic space and, therefore, the highly diverse training sets selected by Avg_GRM_self are strongly related to them.

Our results also indicated that other methods (PAM, Rscore, and Stratified sampling) did not consistently outperform random sampling (Fig. 3, Table 3). In the literature, there have been contrasting results in this matter. For example, Guo et al. (2019) showed that PAM was usually better than targeted PEVmean and CDmean, while the opposite was observed in Rio et al. (2021b) and Isidro y Sánchez and Akdemir (2021). Our results are in line with the latter studies, as PAM was consistently outperformed by targeted CDmean and sometimes it was worse than random sampling (Fig. 3, Table 3). We claim that the contrasting search heuristic settings applied in both studies might be the reason for the different results. Guo et al. (2019) used 100 or 200 iterations of a genetic algorithm (depending on the dataset size) to implement CDmean, while we performed 200 iterations of a genetic algorithm and 2000 steps of simulated annealing (10 steps for each iteration of the genetic algorithm). Ou and Liao (2019) suggested that Rscore was a great alternative to previous criteria, but they only tested it in the targeted scenario and our results show that it is among the weakest methods in the untargeted scenario (Fig. 3, Table 3).

The trends explained above were mostly constant across the entire range of training set sizes, but as seen in Fig. 2, there are some exceptions that can be explained using Figures S2–S22. CDmean sometimes showed a drop in performance for a training set size of 40% of the candidate set, which coincides with the observed transition from sampling frequently from the edge of the genetic space to focusing on the center (see Supplementary Materials, Note 2). PAM usually showed great performance for the smallest training set size but fell off rapidly in datasets with intermediate or strong population structures. This is likely because, for intermediate or large training sets, this method tends to overrepresent isolated individuals, which are very common when population structure is strong, as clearly seen in Figure S18.

### Optimization of the composition of the training set: targeted scenario

As has been shown previously (Akdemir and Isidro-Sánchez 2019), targeted performed substantially better than untargeted optimization, as it allows to maximize the relationship training set–test set, which was not possible in the untargeted scenario (Fig. 4). Among optimization methods, those that use CDmean statistics showed the best results under targeted optimization, which is consistent with previous studies (Akdemir and Isidro-Sánchez 2019; Ou and Liao 2019). Atanda et al. (2021) indicated that the performance of Avg_GRM and CDmean was similar, although in our study (Fig. 3, Tables S32–S52), Avg_GRM consistently had the lowest performance. This criterion is based on finding the training set with the highest relationship with the test set, at cost of minimizing the diversity (Fig. 4). This trade-off (Pszczola et al. 2012) and the poor performance of Avg_GRM confirm that maximizing one at the expense of the other it is not a good strategy. We proposed Avg_GRM_MinMax to improve Avg_GRM by balancing both aspects of the trade-off (Fig. 4). This allowed Avg_GRM_MinMax to outperform Avg_GRM in all situations, being the best targeted method after CDmean and its clustered variants (Fig. 3, Table 3).

In our study, Rscore consistently performed worse than CDmean, which seemed to contrast with the results obtained by Ou and Liao (2019). However, Ou and Liao (2019) compared targeted Rscore with untargeted CDmean, which explains the difference between both studies. CDmean and Rscore are based on similar but not identical concepts, which may explain the observed difference in performance. CDmean is an estimation of the correlation between the GEBVs and the true breeding values (TBVs) (Laloë 1993), while Rscore is derived from the correlation between the GEBVs and the unknown phenotypes (Ou and Liao 2019). The phenotypic correlation is regularly used to calculate the accuracy of the GS models because the TBVs are unknown. Nevertheless, in training set optimization both the TBVs and the phenotypes are unknown and therefore there is no advantage in using the phenotypes over the TBVs. In this sense, the higher the heritability is, the lower the differences between the TBVs and the phenotypes. Therefore, we would expect Rscore and CDmean to be increasingly similar as heritability gets closer to 1. Figure S24 supports this claim, as heritability is positively correlated with Rscore performance but not with CDmean performance. Nevertheless, even for the trait with the highest heritability (HT in ricePopStr, $h^2 = 0.81$), CDmean outperformed Rscore in both targeted and untargeted scenarios (Fig. 3, Tables S50–S52), confirming that training set optimization on the correlation of GEBVs and TBVs is key for great performance. This claim is further supported by the results of Tsai et al. (2021), who found that Bayesian optimization based on predicted genotypic values (an estimator of the TBVs) performed better than a similar optimization based on predicted phenotypic values.

It is important to note that results from this study apply to the framework where training and test sets belong to the same population. Additional challenges that could not be addressed in this study may be present when the training and test sets are different populations. A common scenario would be using older training sets to predict a test set from a newer year, which would involve dealing with the year effect. For instance, Lemeunier et al. (2022) obtained different results from ours, with very good performance with Avg_GRM and similar methods. This is probably because their test set genetic space was smaller and contained within the genetic space of the candidate set. In this situation, for an appropriate training set size, Avg_GRM will select all lines within the candidate set that are in the overlapping part of the genetic space with the test set, but not lines with a low relationship with the test set, and therefore they obtained a higher performance using Avg_GRM. The role of the different training algorithms under different populations will be addressed in a future work

Regarding computational time, the best performing method (CDmean) was also one of the most computationally intensive (Table S53). In fact, in this work, we needed to perform a preselection in the soybean dataset due to the large sample size, as otherwise we would not have been able to perform the 40 repetitions in a reasonable time. It is important to note that in a breeding program the optimization process only needs to be run once, and therefore, the use of CDmean will not be as limited for large datasets. Nevertheless, if computational time is a major concern, Avg_GRM_MinMax would be the best alternative to CDmean, since although it did not reach the same level of performance as CDmean, it still achieved excellent results while being computationally faster.

### Training set size optimization

With the implementation of genomic-assisted breeding tools into the breeding programs, the evaluation of the germplasm has significantly changed. Breeders do not need to evaluate the entire progeny in the field but to optimally select a training set size that will improve the resource allocation within the breeding program. For instance, a reduction in training set size due to optimization implies that more alleles could be tested in the field or that more resources could be invested in more precise phenotyping. Consequently, it is important to optimize the training set size to minimize it without incurring a large loss of accuracy. However, the training set size optimization is computationally intensive as it relies on performing optimization repeatedly for several training set sizes and fitting a function that can predict the evolution of the evaluation criteria as the training set size varies (Figures S25–S30). Our results showed that Avg_GRM_MinMax was the most suitable criterion for the optimization of the training set size in the targeted scenario. This was because it was the only criterion that showed a relative maximum, which allowed us to accurately predict a small training set size with low accuracy loss (Fig. 5). In contrast, CDmean and Rscore did not present a maximum within the range of tested training set sizes, but they could be used to select an optimal size that would result in an expected accuracy loss (Fig. 5), which would be a good approach if they were good predictors of the accuracy. However, for a given target accuracy, CDmean tended to select larger training sets than needed, causing the real accuracies to be greater than the target and therefore being a biased predictor. Rscore had a lower bias than CDmean, but it showed a large variance. For instance, when the target accuracy was 95%, the real accuracies corresponding to the training set sizes selected by Rscore across dataset-trait combinations ranged between 85% and 100%. In addition, optimization with CDmean and Rscore was substantially more time-consuming than the optimization of Avg_GRM_MinMax, making the latter the best option for training set size optimization in the targeted scenario.

With regard to the untargeted scenario, none of the criteria presented a maximum within the range of tested training set sizes and therefore relied on a predefined target accuracy (Fig. 5). Our results showed that none of the criteria were reliable predictors of accuracy, as they presented high bias (CDmean), high variance (Avg_GRM_self) or a combination of the two (Rscore). Furthermore, in Fig. 5 it can be seen that Avg_GRM_self with a target accuracy of 99% yielded very similar results to CDmean with a target accuracy of 95%, suggesting that the three criteria would be similar with a proper tuning of the target accuracy parameter. As a result, we recommend using Avg_GRM_self, as it is markedly faster than the other criteria. However, as the large variance Avg_GRM_self for a target accuracy of 95% may result in outliers with an unacceptably low accuracy, a target accuracy larger than 95% is advised.

It has been shown previously that the larger the training set, the higher the prediction accuracies due to a reduction of bias and variance of marker effect estimates (Clark et al. 2012; Rincent et al. 2012; Isidro et al. 2015; Rincent et al. 2017; Guo et al. 2019; Akdemir and Isidro-Sánchez 2019). As seen in Fig. 5, for targeted optimization a training set size of around 50-55% of the candidate set usually generates accuracies in the range of 95-100% of the maximum, while for untargeted optimization a training set size of 65-85% is needed for similar results. The ability to consider the relationship between the training set and the test set in targeted optimization allows the reduction in training set size, while for untargeted optimization we must increase the sample size if we want to obtain a high likelihood of including in the training set all the lines relevant for making predictions over the test set. This shows that identifying and excluding irrelevant genotypes allows to reduce the training set size, but including them in the training set does not have a negative impact on prediction accuracy, as demonstrated by the fact that the largest accuracy was almost universally reached when the training set was the entire candidate set (Tables S3–S31). In this sense, our study does not follow the results of Lorenz and Smith (2015), who indicated that excluding distinct genotypes improved prediction. This discrepancy might be explained by the fact that the marker density of Lorenz and Smith (2015) was much lower than the one used in our study, with a high marker density being essential to decrease the likelihood of including individuals in the training set whose marker-QTL linkage disequilibrium phases are markedly different to the ones found in the test set (Hickey et al. 2014).

Lastly, we have calculated the correlation between the evaluation metric and accuracy across training set sizes (Figures S31–S36) and found that a higher correlation doesn’t necessarily imply a better ability of the evaluation metric to identify the best training set size. This is exemplified by targeted Avg_GRM_MinMax. This evaluation metric reached a maximum value for an intermediate training set size and then decreased for all datasets (Figures S25–S30), which caused it to be weakly correlated with the evolution of the actual accuracy of the genomic selection models. Nevertheless, we found that the training set size for which Avg_GRM_MinMax reached its maximum was a very good predictor of the actual optimal size (Fig. 5).

### Population structure, heritability and trait architecture

As would have been expected from previous studies (Isidro et al. 2015; Rincent et al. 2017; Ou and Liao 2019), population structure had a major impact on the performance of optimization methods (Table 3), although in this study it did not have the same degree of importance. Our results suggest that methods that generated a very diverse training set (Fig. 4) were usually very robust to variations in ﻿population structure (Figs. 3, S23). For instance, the methods most negatively affected by ﻿population structure were Avg_GRM and PAM (Figure S23), with the former selecting training sets with extremely low diversity (Fig. 4) and the latter over-representing individuals that are isolated in the genetic space, which are very common under high ﻿population structure (Figures S2–S22). Our results also show that including clustering information could improve the performance of an optimization method when ﻿population structure is high, although not by a large margin. In Figure S23 a weak positive correlation was observed between ﻿population structure and the performance of both Stratified sampling and untargeted WIClustCDmean, which is consistent with previous results (Isidro et al. 2015; Rincent et al. 2017; Ou and Liao 2019). However, under the targeted scenario, the inclusion of clustering information did not have any effect on the performance of CDmean, as the performance of all targeted CDmean variants was mostly uncorrelated with ﻿population structure (Figure S23). This difference between targeted vs. untargeted can be explained in Fig. 4. Adding cluster information to optimize the training set implies an increase of genetic diversity in the training set, which improved the performance of methods that count on ﻿population structure. Nevertheless, in the targeted scenario, this advantage is obtained at the expense of reducing its relationship with the test set, which offsets any gains that may have been obtained. Furthermore, Avg_GRM_self had a significantly better performance than any clustered methods in most situations regardless of ﻿population structure in the untargeted scenario (Fig. 3), which indicates that adding cluster information is not key for training set optimization, bypassing the problem of finding a clustering suitable for training set optimization. This is not a trivial issue, as evidenced by the fact that different training set optimization results were obtained with similar methods under the same datasets in studies that had used different clustering approaches (Isidro et al. 2015; Ou and Liao 2019). In addition, the superior performance of Avg_GRM_self suggests that making a diverse training set would be enough to make it robust to ﻿population structure, as no clusters are overrepresented or underrepresented in the training set (Figure S2–S22).

Trait architecture and heritability are important factors affecting the performance of training set optimization, which is consistent with the literature (Rincent et al. 2012; Isidro et al. 2015; Karaman et al. 2016; Olatoye et al. 2020; Heslot and Feoktistov 2020) and is evidenced in our results by the different average performance within a dataset for the different traits (Table 2). However, how they influence optimization is difficult to assess, as the effect of the trait was inconsistent across datasets, and trait architecture is difficult to quantify. It is important to note that the optimization performance in the simulated traits broadly followed the same patterns as in non-simulated traits, although in a much less consistent way (Tables S32–S52). This lack of consistency stems from the high standard error of the mean caused by the fact that a different trait was simulated for each iteration. Regarding the impact of heritability on the performance of the different methods (Figure S23), they tended to be positively correlated in the untargeted scenario while the opposite happened for targeted methods. These results suggest that high heritability traits enable to make a more non-specific diverse training set, while in low heritability traits the specific design of the training set for the desired test set had a key impact.

### Conclusions and final guidelines for training set optimization

In this work, we have been able to identify the most promising strategies for optimizing the training set size and composition and have obtained new insights into the factors that influence the optimization process. Our results suggest that, when heritability is low, the advantage of targeted over untargeted optimization increases (Figure S24), and that selecting a diverse training set is a good strategy to deal with structured populations, as evidenced by the good performance of Avg_GRM_self when ﻿population structure was high. Furthermore, the varying performance of the optimization methods for the different traits within a given dataset (Table 2) shows that there is an impact of trait architecture on training set optimization, although we were not able to identify the underlying mechanisms. Finally, we found that the GS model used did not have a noticeable effect on the performance of training set optimization. For modeling, we recommend testing at least one linear and one nonlinear model for each dataset.

The main benchmark guidelines from this study are:

- The choice of a GS model did not have an impact on prediction accuracies when compared to the variation in prediction accuracies caused by the different training populations.
- The relationship between individuals within the training set seems to play a more important role than the relationship between the training set and the test set on the optimization, i.e, minimizing the relationship between individuals in the training set was more reliable than maximizing the relationship between the training set and the test set, although ideally a balance of the two should be reached.
- *Optimization of training set size*:
  - The optimal training set size depends on the breeding resources budget, but a training set size that covers 50-55% of the total candidate population in the targeted scenario and 65-85% in untargeted will result in an accuracy loss of less than 5% in almost all situations.
  - If a more exact estimation of the optimal training set size is desired, we recommend using Avg_GRM_MinMax for targeted optimization and Avg_GRM_self for untargeted. For the latter, it is needed to set a target accuracy, and we recommend selecting more than 95%.
- *Optimization of training set composition*:
  - Avg_GRM_self is the recommended optimization method in the untargeted scenario as it consistently showed excellent performance (Fig. 3, Table 3), and it is computationally fast (Table S53) and escalates well for datasets with high dimensionality.
  - An ideal untargeted optimization method should be able to use the genomic information without the need to specify a target population. This is shown by the superior performance of Avg_GRM_self (Fig. 3), which is the only method that complies with these requirements.
  - If test set data is available, targeted optimization is advised. Within the targeted scenario, CDmean is the most powerful method under all circumstances (Fig. 3, Table 3).
  - The addition of clustering information to targeted CDmean to account for population structure (WIClustCDmean and OvClustCDmean) does not improve its performance.
  - The most important weakness of targeted CDmean is its low speed for large datasets (Table S53). If time is a limiting factor, Avg_GRM_MinMax can be used as an alternative to CDmean, as its time requirement is substantially lower at the expense of a moderate drop in performance.
  - In summary, CDmean and Avg_GRM_self are the chosen methods for training set optimization under most circumstances.

## Supplementary Information

Below is the link to the electronic supplementary material.

Supplementary file 1 (pdf 52771 KB)

## Data Availability

The datasets analyzed during the current study are available in GitHub, https://github.com/TheRocinante-lab/Publications/tree/main/2022/Fernandez-Gonzalez_et_al_2022_Comparison.
