# Supplementary material for: A comparison of methods for training population optimization in genomic selection
Source: Theor Appl Genet. 2023 Mar 9;136(3):30. doi: 10.1007/s00122-023-04265-6 (PMC9998580; doi:10.1007/s00122-023-04265-6)
Supplement: Supplementary file 1 — (pdf 52771 KB) [file 122_2023_4265_MOESM1_ESM.pdf]

# Supplementary Materials

## Note 1. Genomic selection models

In this study, we focused on genomic best linear unbiased prediction (GBLUP), Bayesian B (BayesB), and Reproducing Kernel Hilbert Space (RKHS), which will allow us to test additive and non-additive effects. As we had the BLUPs from each dataset, we performed a second-step type of genomic prediction. We will briefly explain them starting with the GBLUP linear mixed model (Karimi et al., 2019):

$$\boldsymbol{y} = \mathbf{1}\mu + Z\mathbf{u} + \boldsymbol{\epsilon}, \tag{4}$$

where the vector $\boldsymbol{y}$ contains the genotypic values, $\mathbf{1}$ is a vector of ones $\mu$ is the overall mean, $Z$ is a design matrix for the random effects (genotypic effects), and $\boldsymbol{\epsilon}$ is a vector of residuals. The GEBVs (contained in vector $\boldsymbol{u}$) and residuals follow a multivariate normal distribution of mean $\mathbf{0}$ and covariance $A\sigma_a^2$ and $I\sigma_e^2$ respectively, where $A$ is the genomic additive relationship matrix derived from the marker data, $\sigma_a^2$ is the additive variance captured by the markers, $I$ is the identity matrix and $\sigma_e^2$ is the residual variance. Both variances are estimated by restricted maximum likelihood (REML). The relationship matrix $A$ was calculated using the VanRaden method (VanRaden, 2008):

$$A = \frac{WW'}{2\sum_{j=1}^{n} p_j(1-p_j)}, \tag{5}$$

Where $W_{i,j} = M_{i,j} - 2p_j$, is $M$ is the marker matrix, and $p_j$ is the frequency of the second allele at locus $j$.

The RKHS model is similar to GBLUP substituting the additive relationship matrix ($A$) with a kernel matrix ($K$). The latter matrix contains pairwise distances among the individuals and was calculated from the marker data using a Gaussian kernel (Gianola and van Kaam, 2008):

$$K_{i,j} = e^{\frac{-h(x_i - x_j)^2}{2}}, \tag{6}$$

where $\boldsymbol{x_i}$ and $\boldsymbol{x_j}$ are the vectors containing the markers for the individuals i and j respectively and $h$ is a tuning parameter that controls the rate of decay of the relationships between individuals. $h$ was estimated using cross-validation. The usage of a kernel matrix instead of the additive relationship matrix allows RKHS to capture additive and non-additive effects (de los Campos et al., 2009).

Finally, we used BayesB, which has the following equation:

$$\boldsymbol{y} = \mathbf{1}\mu + X\boldsymbol{\beta} + \boldsymbol{\epsilon},$$

where $\boldsymbol{y}$ is a vector of genotypic values, $\mathbf{1}$ is a vector of ones $\mu$ is the overall mean, $X$ is the marker matrix, $\boldsymbol{\beta}$ is a vector of marker effects and $\boldsymbol{\epsilon}$ is a vector of residuals. More details about its mechanism can be found in Meuwissen et al. (2001).

**Table S1** Summary of the training set optimization methods used. It is important to note that TrainSel always maximizes the selected evaluation criterion and the parameters used were 200 iterations of the genetic algorithm, population size of 200, 5 elite solutions selected, 10 steps of simulated annealing per iteration of the genetic algorithm. $n_{set}$; number of instances present in the set indicated in the subindex. $A$; relationship matrix. $\lambda$; shrinkage parameter. $X$; marker matrix. $C$; contrast matrix. a,b; pondering parameters with any value $\geq 0$. $Tr[\cdot]$; trace of a matrix, $sumsqr[\cdot]$; sum of the squared elements of a matrix, $diag(\cdot)$; main diagonal of a matrix; $mean(\cdot)$; average of all elements of a vector or matrix, TRS; training set, TP; target population. $I$; identity matrix. If $I$ has a subindex, it indicates its dimensions. Otherwise, it has the dimensions needed for the operations. For all other matrices a subindex indicates that a subset is taken. For instance $X_{TRS;All}$ is the marker matrix whose rows are the individuals in the training set and with all the columns taken.

| Method | | Mechanism | Reference |
|---|---|---|---|
| StratSamp | | Random sampling forcing that the amount of individuals selected within each cluster for the training set is proportional to the total size of said cluster in the candidate set. | Isidro et al. (2015) |
| PAM | | 1. Select k initial random medoids (k = training set size).<br>2. Build a cluster around each medoid. All lines in the candidate set are assigned to the cluster with the closest medoid.<br>3. The total cost of the selected set of medoids (T) is calculated as the sum of the dissimilarities of all the elements to their closest medoid.<br>4. Select a random non-medoid individual to become a new medoid, replacing one of the old ones.<br>5. T is recalculated and, if it is smaller than it previously was, the new set of medoids is kept.<br>6. Steps 3-5 are repeated until convergence is reached. | Guo et al. (2019) |
| TrainSel | | 1. Select an initial population comprised of random training sets.<br>2. Evaluate each training set in the population using the desired criterion.<br>3. Select the best training sets to create a elite population.<br>4. Improve the elite solutions using simulated annealing.<br>5. Create new population:<br>(a) Crossover of random pairs of elite solutions.<br>(b) Apply mutation algorithm.<br>(c) Repeat until the desired number of individuals has been obtained.<br>6. Steps 2-5 are repeated until convergence or the maximum number of iterations is reached. | Akdemir et al. (2021) |
| Evaluation criteria for TrainSel | CDmean | $\text{CDmean} = mean(diag(CDmat_{TP;TP}))$<br>$CDmat = (A_{All;TP}(Vinv - (VinvVinv))/sum(VinvA_{TRS;All})/A$<br>$Vinv = (A_{TRS;TRS} + \lambda I)^{-1}$ | Rio et al. (2021a) |
| | CDMEAN2 | $\text{CDMEAN2} = -mean[diag(CX_{TP;All}(X'_{TRS;All}X_{TRS;All} + \lambda * I)^{-1}$<br>$X'_{TRS;All}X_{TRS;All}(X'_{TRS;All}X_{TRS;All} + \lambda * I)^{-1}$<br>$X'_{TP;All}C')/diag(CX_{TP;All}X'_{TP;All}C')]$ | Akdemir (2017) |
| | Rscore | $\text{Rscore} = q_{12}/\sqrt{q_1 q_2}$;<br>$q_{12} = Tr[X^T_{TP;All}IJX_{TP;All}AmatX_{TRS;All}]$;<br>$q_1 = (n_{TP} - 1) + sumsqr[X^T_{TP;All}IJX_{TP;All}]$;<br>$q_2 = sumsqr[Amat^T X^T_{TP;All}IJX_{TP;All}Amat]+$<br>$+sumsqr[X_{TP;All}Amat^T IJAmatX_{TRS;All}]$;<br>$Amat = X^T_{TRS;All}(X_{TRS;All}X^T_{TRS;All} + I(1/n_{markers}))^{-1}$;<br>$IJ = I_{n_{TP}} - I_{n_{TP}}(1/n_{TP})$ | Ou and Liao (2019) |
| | gAvg_GRM | $\text{gAvg\_GRM} = a \cdot mean(A_{TRS;TP}) - b \cdot mean(A_{TRS;TRS})$ | Derived from Atanda et al. (2021) |

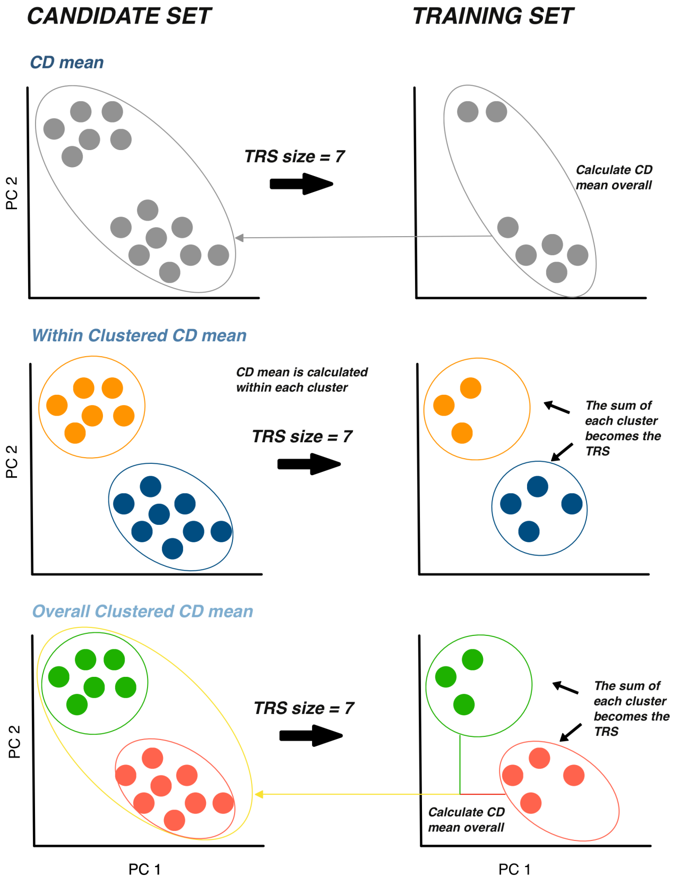


**Fig. S1** Overview of the different CDmean criteria displayed on a toy example with 2 clusters (C1 and C2) on the first two principal components (PC1 and PC2). We aim to use TrainSel to select an optimal training set in an untargeted scenario (the target population is the candidate set). The desired TRS consists on 7 individuals from a candidate set of 14 separated in two clusters of size 6 and 8. Both WIClustCDmean and OvClustCDmean sampled a proportional number of individuals from each cluster.

**Table S2** Average accuracy across training set optimization methods and training set sizes for all possible combinations of datasets, traits and models. The total average is the mean for all values for each model. HT; plant height, FT; flowering time, YLD; yield, FP; florets per panicle, PC; protein content, MO; moisture content, R8; the number of reproductive nodes and pods in the main stem during the reproductive stages R7–R8, DBH; diameter at breast height, DE; density, ST; standability, AN; anthesis date, Sim; simulated trait with heritability 0.5.

**Models Comparison**

| | | Average accuracy | | |
|---|---|---|---|---|
| Dataset | Trait | GBLUP | BayesB | RKHS |
| Maize | FT | 0.674 | 0.674 | 0.671 |
| | HT | 0.411 | 0.414 | 0.407 |
| | YLD | 0.482 | 0.487 | 0.480 |
| | Sim | 0.248 | 0.247 | 0.246 |
| Rice | FT | 0.432 | 0.484 | 0.446 |
| | HT | 0.354 | 0.360 | 0.369 |
| | YLD | 0.265 | 0.276 | 0.294 |
| | Sim | 0.381 | 0.382 | 0.379 |
| RicePopStr | FP | 0.569 | 0.586 | 0.580 |
| | FT | 0.572 | 0.582 | 0.589 |
| | HT | 0.736 | 0.730 | 0.734 |
| | PC | 0.428 | 0.443 | 0.461 |
| | Sim | 0.484 | 0.490 | 0.486 |
| Sorghum | HT | 0.536 | 0.535 | 0.538 |
| | MO | 0.510 | 0.511 | 0.512 |
| | YLD | 0.327 | 0.331 | 0.334 |
| | Sim | 0.404 | 0.406 | 0.403 |
| Soybean | HT | 0.285 | 0.294 | 0.292 |
| | R8 | 0.298 | 0.304 | 0.310 |
| | YLD | 0.528 | 0.531 | 0.530 |
| | Sim | 0.578 | 0.578 | 0.576 |
| Maize | FT | 0.674 | 0.674 | 0.671 |
| | HT | 0.411 | 0.414 | 0.407 |
| | YLD | 0.482 | 0.487 | 0.480 |
| | Sim | 0.248 | 0.247 | 0.246 |
| Switchgrass | AN | 0.808 | 0.800 | 0.808 |
| | HT | 0.591 | 0.593 | 0.591 |
| | ST | 0.659 | 0.658 | 0.659 |
| | Sim | 0.387 | 0.389 | 0.387 |
| Mean | Mean | 0.475 | 0.480 | 0.479 |

**Table S3** Average accuracy and its corresponding standard error of the mean (SEM) across the 40 iterations for all training set optimization methods, models and training set (TRS) sizes (expressed as percentage of the candidate set (CS)) for maize dataset and FT trait. If "targ" is added at the end of the name of a method, it corresponds to targeted optimization. Otherwise, untargeted optimization was performed.

**Maize trait FT**

| | | Average accuracy | | | | | | | | Average accuracy | | | | | |
|---|---|---|---|---|---|---|---|---|---|---|---|---|---|---|---|
| | | GBLUP | | BayesB | | RKHS | | | | GBLUP | | BayesB | | RKHS | |
| Optimization method | TRS size (% of CS) | Mean | SEM | Mean | SEM | Mean | SEM | Optimization method | TRS size (% of CS) | Mean | SEM | Mean | SEM | Mean | SEM |
| RAND | 10 | 0.493 | 0.0244 | 0.483 | 0.0255 | 0.487 | 0.0244 | Rscore | 10 | 0.404 | 0.0276 | 0.386 | 0.0283 | 0.395 | 0.0277 |
| | 20 | 0.590 | 0.0177 | 0.584 | 0.0180 | 0.586 | 0.0176 | | 20 | 0.540 | 0.0252 | 0.536 | 0.0254 | 0.535 | 0.0251 |
| | 40 | 0.676 | 0.0165 | 0.677 | 0.0169 | 0.673 | 0.0164 | | 40 | 0.684 | 0.0169 | 0.687 | 0.0172 | 0.682 | 0.0167 |
| | 60 | 0.715 | 0.0148 | 0.715 | 0.0151 | 0.714 | 0.0146 | | 60 | 0.713 | 0.0165 | 0.717 | 0.0164 | 0.712 | 0.0164 |
| | 80 | 0.747 | 0.0139 | 0.747 | 0.0144 | 0.746 | 0.0137 | | 80 | 0.760 | 0.0137 | 0.762 | 0.0141 | 0.759 | 0.0135 |
| | 100 | 0.775 | 0.0136 | 0.778 | 0.0137 | 0.775 | 0.0134 | | 100 | 0.775 | 0.0136 | 0.778 | 0.0137 | 0.775 | 0.0134 |
| StratSamp | 10 | 0.472 | 0.0220 | 0.466 | 0.0218 | 0.464 | 0.0221 | Rscoretarg | 10 | 0.523 | 0.0144 | 0.518 | 0.0150 | 0.516 | 0.0143 |
| | 20 | 0.533 | 0.0202 | 0.530 | 0.0198 | 0.529 | 0.0199 | | 20 | 0.610 | 0.0161 | 0.608 | 0.0164 | 0.604 | 0.0158 |
| | 40 | 0.660 | 0.0168 | 0.661 | 0.0169 | 0.657 | 0.0165 | | 40 | 0.711 | 0.0138 | 0.713 | 0.0137 | 0.707 | 0.0135 |
| | 60 | 0.725 | 0.0139 | 0.729 | 0.0139 | 0.724 | 0.0137 | | 60 | 0.749 | 0.0128 | 0.752 | 0.0130 | 0.747 | 0.0126 |
| | 80 | 0.749 | 0.0139 | 0.752 | 0.0142 | 0.748 | 0.0138 | | 80 | 0.769 | 0.0134 | 0.770 | 0.0138 | 0.768 | 0.0132 |
| | 100 | 0.775 | 0.0136 | 0.778 | 0.0137 | 0.775 | 0.0134 | | 100 | 0.775 | 0.0136 | 0.778 | 0.0137 | 0.775 | 0.0134 |
| CD | 10 | 0.418 | 0.0229 | 0.422 | 0.0228 | 0.411 | 0.0225 | Avg_GRM | 10 | 0.312 | 0.0270 | 0.299 | 0.0258 | 0.308 | 0.0269 |
| | 20 | 0.563 | 0.0200 | 0.567 | 0.0195 | 0.558 | 0.0195 | | 20 | 0.390 | 0.0319 | 0.373 | 0.0318 | 0.384 | 0.0321 |
| | 40 | 0.687 | 0.0166 | 0.691 | 0.0173 | 0.686 | 0.0164 | | 40 | 0.560 | 0.0246 | 0.551 | 0.0259 | 0.559 | 0.0251 |
| | 60 | 0.753 | 0.0126 | 0.755 | 0.0132 | 0.751 | 0.0126 | | 60 | 0.633 | 0.0199 | 0.634 | 0.0202 | 0.634 | 0.0200 |
| | 80 | 0.766 | 0.0135 | 0.770 | 0.0138 | 0.766 | 0.0134 | | 80 | 0.677 | 0.0184 | 0.681 | 0.0188 | 0.679 | 0.0185 |
| | 100 | 0.775 | 0.0136 | 0.778 | 0.0137 | 0.775 | 0.0134 | | 100 | 0.775 | 0.0136 | 0.778 | 0.0137 | 0.775 | 0.0134 |
| CDtarg | 10 | 0.558 | 0.0144 | 0.555 | 0.0144 | 0.544 | 0.0149 | Avg_GRMtarg | 10 | 0.459 | 0.0216 | 0.451 | 0.0216 | 0.447 | 0.0223 |
| | 20 | 0.643 | 0.0156 | 0.638 | 0.0158 | 0.635 | 0.0157 | | 20 | 0.557 | 0.0230 | 0.550 | 0.0234 | 0.548 | 0.0232 |
| | 40 | 0.727 | 0.0136 | 0.725 | 0.0140 | 0.723 | 0.0134 | | 40 | 0.677 | 0.0162 | 0.674 | 0.0169 | 0.672 | 0.0162 |
| | 60 | 0.759 | 0.0136 | 0.759 | 0.0139 | 0.756 | 0.0134 | | 60 | 0.749 | 0.0136 | 0.749 | 0.0143 | 0.747 | 0.0135 |
| | 80 | 0.772 | 0.0132 | 0.773 | 0.0135 | 0.770 | 0.0131 | | 80 | 0.769 | 0.0135 | 0.769 | 0.0140 | 0.768 | 0.0134 |
| | 100 | 0.775 | 0.0136 | 0.778 | 0.0137 | 0.775 | 0.0134 | | 100 | 0.775 | 0.0136 | 0.778 | 0.0137 | 0.775 | 0.0134 |
| OvClustCD | 10 | 0.385 | 0.0249 | 0.378 | 0.0253 | 0.378 | 0.0242 | Avg_GRM_MinMax | 10 | 0.477 | 0.0229 | 0.472 | 0.0233 | 0.470 | 0.0229 |
| | 20 | 0.507 | 0.0172 | 0.505 | 0.0171 | 0.494 | 0.0167 | | 20 | 0.605 | 0.0147 | 0.606 | 0.0152 | 0.599 | 0.0144 |
| | 40 | 0.690 | 0.0164 | 0.693 | 0.0170 | 0.687 | 0.0162 | | 40 | 0.716 | 0.0128 | 0.723 | 0.0133 | 0.714 | 0.0126 |
| | 60 | 0.759 | 0.0134 | 0.761 | 0.0138 | 0.757 | 0.0132 | | 60 | 0.754 | 0.0134 | 0.756 | 0.0137 | 0.752 | 0.0132 |
| | 80 | 0.779 | 0.0135 | 0.777 | 0.0141 | 0.778 | 0.0134 | | 80 | 0.771 | 0.0138 | 0.774 | 0.0141 | 0.770 | 0.0136 |
| | 100 | 0.775 | 0.0136 | 0.778 | 0.0137 | 0.775 | 0.0134 | | 100 | 0.775 | 0.0136 | 0.778 | 0.0137 | 0.775 | 0.0134 |
| OvClustCDtarg | 10 | 0.536 | 0.0160 | 0.537 | 0.0146 | 0.531 | 0.0157 | Avg_GRM_MinMaxtarg | 10 | 0.610 | 0.0131 | 0.604 | 0.0131 | 0.598 | 0.0137 |
| | 20 | 0.662 | 0.0133 | 0.662 | 0.0125 | 0.655 | 0.0128 | | 20 | 0.691 | 0.0143 | 0.688 | 0.0145 | 0.685 | 0.0142 |
| | 40 | 0.742 | 0.0129 | 0.740 | 0.0132 | 0.738 | 0.0127 | | 40 | 0.740 | 0.0126 | 0.740 | 0.0128 | 0.736 | 0.0124 |
| | 60 | 0.766 | 0.0129 | 0.767 | 0.0129 | 0.764 | 0.0127 | | 60 | 0.763 | 0.0137 | 0.763 | 0.0138 | 0.761 | 0.0135 |
| | 80 | 0.774 | 0.0131 | 0.776 | 0.0134 | 0.772 | 0.0130 | | 80 | 0.771 | 0.0137 | 0.775 | 0.0139 | 0.770 | 0.0136 |
| | 100 | 0.775 | 0.0136 | 0.778 | 0.0137 | 0.775 | 0.0134 | | 100 | 0.775 | 0.0136 | 0.778 | 0.0137 | 0.775 | 0.0134 |
| WIClustCD | 10 | 0.426 | 0.0178 | 0.413 | 0.0179 | 0.419 | 0.0179 | Avg_GRM_self | 10 | 0.525 | 0.0163 | 0.523 | 0.0163 | 0.515 | 0.0164 |
| | 20 | 0.507 | 0.0171 | 0.511 | 0.0169 | 0.500 | 0.0174 | | 20 | 0.631 | 0.0161 | 0.633 | 0.0169 | 0.626 | 0.0158 |
| | 40 | 0.697 | 0.0145 | 0.699 | 0.0147 | 0.693 | 0.0143 | | 40 | 0.725 | 0.0142 | 0.727 | 0.0145 | 0.723 | 0.0139 |
| | 60 | 0.756 | 0.0138 | 0.758 | 0.0143 | 0.754 | 0.0136 | | 60 | 0.754 | 0.0131 | 0.758 | 0.0135 | 0.753 | 0.0129 |
| | 80 | 0.775 | 0.0137 | 0.775 | 0.0138 | 0.773 | 0.0136 | | 80 | 0.771 | 0.0135 | 0.772 | 0.0140 | 0.770 | 0.0133 |
| | 100 | 0.775 | 0.0136 | 0.778 | 0.0137 | 0.775 | 0.0134 | | 100 | 0.775 | 0.0136 | 0.778 | 0.0137 | 0.775 | 0.0134 |
| WIClustCDtarg | 10 | 0.561 | 0.0179 | 0.558 | 0.0179 | 0.556 | 0.0173 | PAM | 10 | 0.481 | 0.0190 | 0.474 | 0.0193 | 0.472 | 0.0187 |
| | 20 | 0.680 | 0.0130 | 0.680 | 0.0125 | 0.673 | 0.0131 | | 20 | 0.572 | 0.0178 | 0.567 | 0.0171 | 0.567 | 0.0175 |
| | 40 | 0.742 | 0.0122 | 0.743 | 0.0122 | 0.739 | 0.0119 | | 40 | 0.690 | 0.0173 | 0.691 | 0.0178 | 0.688 | 0.0171 |
| | 60 | 0.763 | 0.0131 | 0.763 | 0.0135 | 0.761 | 0.0129 | | 60 | 0.752 | 0.0131 | 0.754 | 0.0133 | 0.751 | 0.0130 |
| | 80 | 0.771 | 0.0137 | 0.772 | 0.0139 | 0.770 | 0.0136 | | 80 | 0.765 | 0.0135 | 0.768 | 0.0141 | 0.765 | 0.0134 |
| | 100 | 0.775 | 0.0136 | 0.778 | 0.0137 | 0.775 | 0.0134 | | 100 | 0.775 | 0.0136 | 0.778 | 0.0137 | 0.775 | 0.0134 |

**Table S4** Average accuracy and its corresponding standard error of the mean (SEM) across the 40 iterations for all training set optimization methods, models and training set (TRS) sizes (expressed as percentage of the candidate set (CS)) for maize dataset and HT trait. If "targ" is added at the end of the name of a method, it corresponds to targeted optimization. Otherwise, untargeted optimization was performed.

**Maize trait HT**

| | | Average accuracy | | | | | | | | Average accuracy | | | | | |
|---|---|---|---|---|---|---|---|---|---|---|---|---|---|---|---|
| | | GBLUP | | BayesB | | RKHS | | | | GBLUP | | BayesB | | RKHS | |
| Optimization method | TRS size (% of CS) | Mean | SEM | Mean | SEM | Mean | SEM | Optimization method | TRS size (% of CS) | Mean | SEM | Mean | SEM | Mean | SEM |
| RAND | 10 | 0.289 | 0.0148 | 0.287 | 0.0163 | 0.278 | 0.0153 | Rscore | 10 | 0.260 | 0.0204 | 0.251 | 0.0199 | 0.253 | 0.0206 |
| | 20 | 0.368 | 0.0173 | 0.368 | 0.0173 | 0.361 | 0.0173 | | 20 | 0.319 | 0.0173 | 0.316 | 0.0185 | 0.313 | 0.0176 |
| | 40 | 0.405 | 0.0173 | 0.409 | 0.0169 | 0.403 | 0.0173 | | 40 | 0.421 | 0.0196 | 0.428 | 0.0189 | 0.419 | 0.0194 |
| | 60 | 0.424 | 0.0158 | 0.427 | 0.0151 | 0.423 | 0.0158 | | 60 | 0.435 | 0.0177 | 0.439 | 0.0172 | 0.432 | 0.0179 |
| | 80 | 0.452 | 0.0171 | 0.454 | 0.0170 | 0.450 | 0.0170 | | 80 | 0.460 | 0.0177 | 0.460 | 0.0175 | 0.459 | 0.0177 |
| | 100 | 0.473 | 0.0166 | 0.474 | 0.0160 | 0.471 | 0.0167 | | 100 | 0.473 | 0.0166 | 0.474 | 0.0160 | 0.471 | 0.0167 |
| StratSamp | 10 | 0.283 | 0.0191 | 0.280 | 0.0195 | 0.274 | 0.0196 | Rscoretarg | 10 | 0.266 | 0.0207 | 0.273 | 0.0214 | 0.260 | 0.0217 |
| | 20 | 0.347 | 0.0149 | 0.349 | 0.0140 | 0.345 | 0.0149 | | 20 | 0.350 | 0.0202 | 0.354 | 0.0194 | 0.343 | 0.0202 |
| | 40 | 0.411 | 0.0177 | 0.409 | 0.0178 | 0.405 | 0.0178 | | 40 | 0.415 | 0.0177 | 0.417 | 0.0178 | 0.410 | 0.0180 |
| | 60 | 0.438 | 0.0181 | 0.442 | 0.0174 | 0.437 | 0.0181 | | 60 | 0.445 | 0.0174 | 0.447 | 0.0169 | 0.441 | 0.0172 |
| | 80 | 0.458 | 0.0165 | 0.460 | 0.0164 | 0.457 | 0.0165 | | 80 | 0.463 | 0.0166 | 0.464 | 0.0160 | 0.461 | 0.0166 |
| | 100 | 0.473 | 0.0166 | 0.474 | 0.0160 | 0.471 | 0.0167 | | 100 | 0.473 | 0.0166 | 0.474 | 0.0160 | 0.471 | 0.0167 |
| CD | 10 | 0.308 | 0.0158 | 0.310 | 0.0155 | 0.302 | 0.0157 | Avg_GRM | 10 | 0.203 | 0.0217 | 0.204 | 0.0219 | 0.195 | 0.0223 |
| | 20 | 0.370 | 0.0168 | 0.370 | 0.0159 | 0.367 | 0.0164 | | 20 | 0.269 | 0.0181 | 0.272 | 0.0171 | 0.260 | 0.0185 |
| | 40 | 0.410 | 0.0188 | 0.421 | 0.0186 | 0.408 | 0.0186 | | 40 | 0.342 | 0.0196 | 0.357 | 0.0185 | 0.340 | 0.0198 |
| | 60 | 0.454 | 0.0181 | 0.470 | 0.0171 | 0.454 | 0.0180 | | 60 | 0.401 | 0.0164 | 0.407 | 0.0157 | 0.397 | 0.0172 |
| | 80 | 0.461 | 0.0170 | 0.468 | 0.0162 | 0.459 | 0.0170 | | 80 | 0.433 | 0.0153 | 0.438 | 0.0152 | 0.432 | 0.0154 |
| | 100 | 0.473 | 0.0166 | 0.474 | 0.0160 | 0.471 | 0.0167 | | 100 | 0.473 | 0.0166 | 0.474 | 0.0160 | 0.471 | 0.0167 |
| CDtarg | 10 | 0.340 | 0.0192 | 0.340 | 0.0186 | 0.330 | 0.0190 | Avg_GRMtarg | 10 | 0.243 | 0.0265 | 0.252 | 0.0270 | 0.243 | 0.0260 |
| | 20 | 0.373 | 0.0181 | 0.374 | 0.0184 | 0.369 | 0.0180 | | 20 | 0.292 | 0.0261 | 0.304 | 0.0246 | 0.288 | 0.0252 |
| | 40 | 0.433 | 0.0175 | 0.434 | 0.0171 | 0.427 | 0.0178 | | 40 | 0.383 | 0.0223 | 0.399 | 0.0217 | 0.377 | 0.0229 |
| | 60 | 0.461 | 0.0165 | 0.458 | 0.0170 | 0.456 | 0.0166 | | 60 | 0.437 | 0.0183 | 0.445 | 0.0183 | 0.433 | 0.0191 |
| | 80 | 0.470 | 0.0166 | 0.469 | 0.0163 | 0.468 | 0.0166 | | 80 | 0.459 | 0.0171 | 0.462 | 0.0163 | 0.458 | 0.0171 |
| | 100 | 0.473 | 0.0166 | 0.474 | 0.0160 | 0.471 | 0.0167 | | 100 | 0.473 | 0.0166 | 0.474 | 0.0160 | 0.471 | 0.0167 |
| OvClustCD | 10 | 0.304 | 0.0163 | 0.304 | 0.0163 | 0.299 | 0.0161 | Avg_GRM_MinMax | 10 | 0.304 | 0.0158 | 0.305 | 0.0150 | 0.299 | 0.0159 |
| | 20 | 0.362 | 0.0173 | 0.355 | 0.0181 | 0.355 | 0.0174 | | 20 | 0.381 | 0.0154 | 0.379 | 0.0157 | 0.371 | 0.0160 |
| | 40 | 0.423 | 0.0175 | 0.431 | 0.0170 | 0.420 | 0.0175 | | 40 | 0.420 | 0.0188 | 0.426 | 0.0184 | 0.419 | 0.0188 |
| | 60 | 0.444 | 0.0193 | 0.455 | 0.0186 | 0.443 | 0.0191 | | 60 | 0.463 | 0.0181 | 0.467 | 0.0179 | 0.461 | 0.0182 |
| | 80 | 0.448 | 0.0188 | 0.457 | 0.0177 | 0.446 | 0.0189 | | 80 | 0.463 | 0.0159 | 0.465 | 0.0157 | 0.462 | 0.0160 |
| | 100 | 0.473 | 0.0166 | 0.474 | 0.0160 | 0.471 | 0.0167 | | 100 | 0.473 | 0.0166 | 0.474 | 0.0160 | 0.471 | 0.0167 |
| OvClustCDtarg | 10 | 0.331 | 0.0200 | 0.329 | 0.0197 | 0.321 | 0.0201 | Avg_GRM_MinMaxtarg | 10 | 0.332 | 0.0227 | 0.333 | 0.0220 | 0.326 | 0.0226 |
| | 20 | 0.387 | 0.0180 | 0.389 | 0.0180 | 0.379 | 0.0184 | | 20 | 0.385 | 0.0193 | 0.389 | 0.0185 | 0.383 | 0.0193 |
| | 40 | 0.443 | 0.0175 | 0.446 | 0.0174 | 0.440 | 0.0176 | | 40 | 0.438 | 0.0158 | 0.443 | 0.0152 | 0.435 | 0.0160 |
| | 60 | 0.459 | 0.0172 | 0.460 | 0.0169 | 0.455 | 0.0174 | | 60 | 0.454 | 0.0164 | 0.458 | 0.0168 | 0.452 | 0.0164 |
| | 80 | 0.466 | 0.0166 | 0.467 | 0.0161 | 0.463 | 0.0169 | | 80 | 0.462 | 0.0163 | 0.463 | 0.0166 | 0.462 | 0.0164 |
| | 100 | 0.473 | 0.0166 | 0.474 | 0.0160 | 0.471 | 0.0167 | | 100 | 0.473 | 0.0166 | 0.474 | 0.0160 | 0.471 | 0.0167 |
| WIClustCD | 10 | 0.293 | 0.0163 | 0.294 | 0.0158 | 0.285 | 0.0168 | Avg_GRM_self | 10 | 0.332 | 0.0198 | 0.332 | 0.0194 | 0.327 | 0.0200 |
| | 20 | 0.358 | 0.0166 | 0.356 | 0.0166 | 0.348 | 0.0174 | | 20 | 0.382 | 0.0178 | 0.385 | 0.0183 | 0.378 | 0.0177 |
| | 40 | 0.397 | 0.0190 | 0.407 | 0.0178 | 0.394 | 0.0188 | | 40 | 0.429 | 0.0206 | 0.434 | 0.0200 | 0.426 | 0.0206 |
| | 60 | 0.432 | 0.0212 | 0.444 | 0.0195 | 0.430 | 0.0211 | | 60 | 0.456 | 0.0158 | 0.462 | 0.0158 | 0.454 | 0.0161 |
| | 80 | 0.452 | 0.0171 | 0.460 | 0.0164 | 0.450 | 0.0172 | | 80 | 0.463 | 0.0158 | 0.463 | 0.0161 | 0.461 | 0.0158 |
| | 100 | 0.473 | 0.0166 | 0.474 | 0.0160 | 0.471 | 0.0167 | | 100 | 0.473 | 0.0166 | 0.474 | 0.0160 | 0.471 | 0.0167 |
| WIClustCDtarg | 10 | 0.333 | 0.0205 | 0.330 | 0.0202 | 0.325 | 0.0207 | PAM | 10 | 0.322 | 0.0138 | 0.322 | 0.0143 | 0.316 | 0.0136 |
| | 20 | 0.399 | 0.0190 | 0.404 | 0.0189 | 0.387 | 0.0187 | | 20 | 0.375 | 0.0139 | 0.375 | 0.0144 | 0.371 | 0.0137 |
| | 40 | 0.444 | 0.0175 | 0.449 | 0.0170 | 0.441 | 0.0175 | | 40 | 0.422 | 0.0162 | 0.430 | 0.0152 | 0.419 | 0.0160 |
| | 60 | 0.464 | 0.0170 | 0.466 | 0.0165 | 0.461 | 0.0171 | | 60 | 0.461 | 0.0162 | 0.467 | 0.0157 | 0.461 | 0.0161 |
| | 80 | 0.470 | 0.0170 | 0.471 | 0.0166 | 0.468 | 0.0172 | | 80 | 0.455 | 0.0168 | 0.461 | 0.0164 | 0.452 | 0.0167 |
| | 100 | 0.473 | 0.0166 | 0.474 | 0.0160 | 0.471 | 0.0167 | | 100 | 0.473 | 0.0166 | 0.474 | 0.0160 | 0.471 | 0.0167 |

**Table S5** Average accuracy and its corresponding standard error of the mean (SEM) across the 40 iterations for all training set optimization methods, models and training set (TRS) sizes (expressed as percentage of the candidate set (CS)) for maize dataset and YLD trait. If ”targ” is added at the end of the name of a method, it corresponds to targeted optimization. Otherwise, untargeted optimization was performed.

**Maize trait YLD**

| Optimization method | TRS size (% of CS) | Average accuracy | | | | | | Optimization method | TRS size (% of CS) | Average accuracy | | | | | |
|---|---|---|---|---|---|---|---|---|---|---|---|---|---|---|---|
| | | GBLUP | | BayesB | | RKHS | | | | GBLUP | | BayesB | | RKHS | |
| | | Mean | SEM | Mean | SEM | Mean | SEM | | | Mean | SEM | Mean | SEM | Mean | SEM |
| RAND | 10 | 0.387 | 0.01906 | 0.383 | 0.01885 | 0.384 | 0.01900 | Rscore | 10 | 0.341 | 0.01739 | 0.336 | 0.01726 | 0.330 | 0.01809 |
| | 20 | 0.433 | 0.01242 | 0.439 | 0.01201 | 0.431 | 0.01230 | | 20 | 0.417 | 0.01278 | 0.423 | 0.01282 | 0.414 | 0.01287 |
| | 40 | 0.493 | 0.00962 | 0.499 | 0.00967 | 0.493 | 0.00966 | | 40 | 0.492 | 0.01205 | 0.502 | 0.01182 | 0.491 | 0.01202 |
| | 60 | 0.511 | 0.01034 | 0.520 | 0.01037 | 0.510 | 0.01027 | | 60 | 0.499 | 0.01320 | 0.509 | 0.01284 | 0.498 | 0.01331 |
| | 80 | 0.515 | 0.01242 | 0.523 | 0.01220 | 0.515 | 0.01223 | | 80 | 0.525 | 0.01282 | 0.530 | 0.01266 | 0.525 | 0.01281 |
| | 100 | 0.536 | 0.01104 | 0.540 | 0.01083 | 0.535 | 0.01096 | | 100 | 0.536 | 0.01104 | 0.540 | 0.01083 | 0.535 | 0.01096 |
| StratSamp | 10 | 0.364 | 0.01565 | 0.362 | 0.01574 | 0.358 | 0.01617 | Rscoretarg | 10 | 0.346 | 0.01833 | 0.342 | 0.01952 | 0.337 | 0.01911 |
| | 20 | 0.430 | 0.01311 | 0.430 | 0.01284 | 0.429 | 0.01315 | | 20 | 0.447 | 0.01264 | 0.450 | 0.01323 | 0.443 | 0.01273 |
| | 40 | 0.477 | 0.01242 | 0.484 | 0.01236 | 0.476 | 0.01224 | | 40 | 0.500 | 0.01178 | 0.509 | 0.01157 | 0.498 | 0.01164 |
| | 60 | 0.515 | 0.01175 | 0.521 | 0.01204 | 0.515 | 0.01174 | | 60 | 0.522 | 0.01214 | 0.525 | 0.01219 | 0.520 | 0.01213 |
| | 80 | 0.523 | 0.01113 | 0.529 | 0.01119 | 0.522 | 0.01106 | | 80 | 0.535 | 0.01166 | 0.539 | 0.01140 | 0.534 | 0.01155 |
| | 100 | 0.536 | 0.01104 | 0.540 | 0.01083 | 0.535 | 0.01096 | | 100 | 0.536 | 0.01104 | 0.540 | 0.01083 | 0.535 | 0.01096 |
| CD | 10 | 0.352 | 0.01575 | 0.350 | 0.01639 | 0.347 | 0.01582 | Avg_GRM | 10 | 0.288 | 0.02042 | 0.293 | 0.02148 | 0.279 | 0.02114 |
| | 20 | 0.431 | 0.01398 | 0.433 | 0.01385 | 0.427 | 0.01399 | | 20 | 0.358 | 0.01632 | 0.362 | 0.01535 | 0.348 | 0.01666 |
| | 40 | 0.484 | 0.01236 | 0.495 | 0.01252 | 0.484 | 0.01231 | | 40 | 0.424 | 0.01595 | 0.425 | 0.01613 | 0.422 | 0.01591 |
| | 60 | 0.507 | 0.01392 | 0.521 | 0.01356 | 0.508 | 0.01391 | | 60 | 0.473 | 0.01406 | 0.482 | 0.01388 | 0.474 | 0.01401 |
| | 80 | 0.526 | 0.01298 | 0.541 | 0.01303 | 0.528 | 0.01294 | | 80 | 0.511 | 0.01202 | 0.518 | 0.01176 | 0.512 | 0.01197 |
| | 100 | 0.536 | 0.01104 | 0.540 | 0.01083 | 0.535 | 0.01096 | | 100 | 0.536 | 0.01104 | 0.540 | 0.01083 | 0.535 | 0.01096 |
| CDtarg | 10 | 0.397 | 0.01564 | 0.398 | 0.01576 | 0.391 | 0.01627 | Avg_GRMtarg | 10 | 0.321 | 0.02172 | 0.330 | 0.02159 | 0.313 | 0.02272 |
| | 20 | 0.458 | 0.01300 | 0.459 | 0.01283 | 0.455 | 0.01303 | | 20 | 0.395 | 0.01859 | 0.396 | 0.01855 | 0.386 | 0.01909 |
| | 40 | 0.513 | 0.01136 | 0.514 | 0.01127 | 0.512 | 0.01127 | | 40 | 0.479 | 0.01399 | 0.483 | 0.01407 | 0.471 | 0.01410 |
| | 60 | 0.534 | 0.01116 | 0.538 | 0.01100 | 0.533 | 0.01102 | | 60 | 0.526 | 0.01188 | 0.531 | 0.01207 | 0.522 | 0.01151 |
| | 80 | 0.535 | 0.01107 | 0.537 | 0.01106 | 0.534 | 0.01094 | | 80 | 0.534 | 0.01101 | 0.539 | 0.01098 | 0.533 | 0.01093 |
| | 100 | 0.536 | 0.01104 | 0.540 | 0.01083 | 0.535 | 0.01096 | | 100 | 0.536 | 0.01104 | 0.540 | 0.01083 | 0.535 | 0.01096 |
| OvClustCD | 10 | 0.359 | 0.01496 | 0.362 | 0.01581 | 0.351 | 0.01499 | Avg_GRM_MinMax | 10 | 0.384 | 0.01828 | 0.390 | 0.01818 | 0.379 | 0.01834 |
| | 20 | 0.419 | 0.01425 | 0.421 | 0.01498 | 0.415 | 0.01424 | | 20 | 0.448 | 0.01364 | 0.447 | 0.01400 | 0.445 | 0.01359 |
| | 40 | 0.486 | 0.01294 | 0.497 | 0.01332 | 0.485 | 0.01296 | | 40 | 0.501 | 0.01206 | 0.507 | 0.01140 | 0.499 | 0.01187 |
| | 60 | 0.515 | 0.01211 | 0.527 | 0.01183 | 0.517 | 0.01201 | | 60 | 0.515 | 0.01214 | 0.521 | 0.01168 | 0.515 | 0.01207 |
| | 80 | 0.536 | 0.01117 | 0.547 | 0.01089 | 0.537 | 0.01120 | | 80 | 0.535 | 0.01054 | 0.541 | 0.01055 | 0.534 | 0.01042 |
| | 100 | 0.536 | 0.01104 | 0.540 | 0.01083 | 0.535 | 0.01096 | | 100 | 0.536 | 0.01104 | 0.540 | 0.01083 | 0.535 | 0.01096 |
| OvClustCDtarg | 10 | 0.396 | 0.01795 | 0.398 | 0.01761 | 0.389 | 0.01817 | Avg_GRM_MinMaxtarg | 10 | 0.405 | 0.01438 | 0.406 | 0.01449 | 0.401 | 0.01432 |
| | 20 | 0.476 | 0.01310 | 0.476 | 0.01254 | 0.473 | 0.01300 | | 20 | 0.477 | 0.01242 | 0.481 | 0.01243 | 0.475 | 0.01231 |
| | 40 | 0.522 | 0.01101 | 0.528 | 0.01126 | 0.521 | 0.01100 | | 40 | 0.522 | 0.01021 | 0.526 | 0.01040 | 0.520 | 0.01003 |
| | 60 | 0.534 | 0.01086 | 0.538 | 0.01085 | 0.534 | 0.01077 | | 60 | 0.534 | 0.01050 | 0.541 | 0.01046 | 0.533 | 0.01049 |
| | 80 | 0.537 | 0.01108 | 0.541 | 0.01101 | 0.536 | 0.01094 | | 80 | 0.541 | 0.01068 | 0.546 | 0.01036 | 0.540 | 0.01061 |
| | 100 | 0.536 | 0.01104 | 0.540 | 0.01083 | 0.535 | 0.01096 | | 100 | 0.536 | 0.01104 | 0.540 | 0.01083 | 0.535 | 0.01096 |
| WIClustCD | 10 | 0.354 | 0.01547 | 0.351 | 0.01540 | 0.343 | 0.01556 | Avg_GRM_self | 10 | 0.395 | 0.01581 | 0.401 | 0.01530 | 0.390 | 0.01583 |
| | 20 | 0.429 | 0.01137 | 0.434 | 0.01130 | 0.425 | 0.01150 | | 20 | 0.459 | 0.01166 | 0.464 | 0.01164 | 0.456 | 0.01161 |
| | 40 | 0.473 | 0.01299 | 0.483 | 0.01276 | 0.473 | 0.01289 | | 40 | 0.501 | 0.01250 | 0.512 | 0.01171 | 0.500 | 0.01234 |
| | 60 | 0.507 | 0.01277 | 0.519 | 0.01219 | 0.507 | 0.01248 | | 60 | 0.523 | 0.01082 | 0.533 | 0.01081 | 0.522 | 0.01075 |
| | 80 | 0.539 | 0.01134 | 0.548 | 0.01135 | 0.540 | 0.01132 | | 80 | 0.535 | 0.01137 | 0.542 | 0.01114 | 0.535 | 0.01131 |
| | 100 | 0.536 | 0.01104 | 0.540 | 0.01083 | 0.535 | 0.01096 | | 100 | 0.536 | 0.01104 | 0.540 | 0.01083 | 0.535 | 0.01096 |
| WIClustCDtarg | 10 | 0.397 | 0.01584 | 0.395 | 0.01611 | 0.389 | 0.01603 | PAM | 10 | 0.393 | 0.01441 | 0.397 | 0.01519 | 0.388 | 0.01443 |
| | 20 | 0.481 | 0.01287 | 0.482 | 0.01292 | 0.478 | 0.01280 | | 20 | 0.422 | 0.01266 | 0.435 | 0.01224 | 0.420 | 0.01266 |
| | 40 | 0.525 | 0.01063 | 0.529 | 0.01078 | 0.523 | 0.01056 | | 40 | 0.473 | 0.01222 | 0.486 | 0.01224 | 0.473 | 0.01219 |
| | 60 | 0.535 | 0.01059 | 0.541 | 0.01051 | 0.535 | 0.01048 | | 60 | 0.517 | 0.01261 | 0.530 | 0.01276 | 0.518 | 0.01257 |
| | 80 | 0.533 | 0.01082 | 0.539 | 0.01073 | 0.533 | 0.01067 | | 80 | 0.515 | 0.01190 | 0.531 | 0.01167 | 0.515 | 0.01173 |
| | 100 | 0.536 | 0.01104 | 0.540 | 0.01083 | 0.535 | 0.01096 | | 100 | 0.536 | 0.01104 | 0.540 | 0.01083 | 0.535 | 0.01096 |

**Table S6** Average accuracy and its corresponding standard error of the mean (SEM) across the 40 iterations for all training set optimization methods, models and training set (TRS) sizes (expressed as percentage of the candidate set (CS)) for maize dataset and the simulated trait. If "targ" is added at the end of the name of a method, it corresponds to targeted optimization. Otherwise, untargeted optimization was performed.

**Maize trait simulated1**

| | | Average accuracy | | | | | | | | Average accuracy | | | | | |
|---|---|---|---|---|---|---|---|---|---|---|---|---|---|---|---|
| | | GBLUP | | BayesB | | RKHS | | | | GBLUP | | BayesB | | RKHS | |
| Optimization method | TRS size (% of CS) | Mean | SEM | Mean | SEM | Mean | SEM | Optimization method | TRS size (% of CS) | Mean | SEM | Mean | SEM | Mean | SEM |
| RAND | 10 | 0.1518 | 0.0238 | 0.1556 | 0.0239 | 0.1523 | 0.0242 | Rscore | 10 | 0.1103 | 0.0242 | 0.1065 | 0.0244 | 0.1066 | 0.0237 |
| | 20 | 0.1694 | 0.0236 | 0.1654 | 0.0239 | 0.1700 | 0.0240 | | 20 | 0.1874 | 0.0218 | 0.1865 | 0.0211 | 0.1816 | 0.0210 |
| | 40 | 0.2367 | 0.0233 | 0.2337 | 0.0245 | 0.2339 | 0.0236 | | 40 | 0.2428 | 0.0209 | 0.2455 | 0.0206 | 0.2413 | 0.0215 |
| | 60 | 0.2730 | 0.0209 | 0.2755 | 0.0205 | 0.2747 | 0.0212 | | 60 | 0.2918 | 0.0211 | 0.2964 | 0.0213 | 0.2860 | 0.0210 |
| | 80 | 0.2869 | 0.0232 | 0.2905 | 0.0231 | 0.2847 | 0.0233 | | 80 | 0.3074 | 0.0187 | 0.3095 | 0.0188 | 0.3003 | 0.0194 |
| | 100 | 0.3054 | 0.0206 | 0.3024 | 0.0205 | 0.3030 | 0.0210 | | 100 | 0.3054 | 0.0206 | 0.3024 | 0.0205 | 0.3030 | 0.0210 |
| StratSamp | 10 | 0.1332 | 0.0235 | 0.1258 | 0.0247 | 0.1345 | 0.0233 | Rscoretarg | 10 | 0.1703 | 0.0235 | 0.1746 | 0.0227 | 0.1772 | 0.0240 |
| | 20 | 0.1945 | 0.0163 | 0.1948 | 0.0170 | 0.1973 | 0.0156 | | 20 | 0.2210 | 0.0186 | 0.2242 | 0.0172 | 0.2196 | 0.0186 |
| | 40 | 0.2528 | 0.0182 | 0.2594 | 0.0172 | 0.2503 | 0.0178 | | 40 | 0.2495 | 0.0213 | 0.2494 | 0.0201 | 0.2496 | 0.0214 |
| | 60 | 0.2815 | 0.0187 | 0.2845 | 0.0182 | 0.2838 | 0.0181 | | 60 | 0.2732 | 0.0219 | 0.2690 | 0.0221 | 0.2719 | 0.0222 |
| | 80 | 0.3007 | 0.0161 | 0.3007 | 0.0160 | 0.2983 | 0.0161 | | 80 | 0.2858 | 0.0194 | 0.2828 | 0.0192 | 0.2833 | 0.0199 |
| | 100 | 0.3054 | 0.0206 | 0.3024 | 0.0205 | 0.3030 | 0.0210 | | 100 | 0.3054 | 0.0206 | 0.3024 | 0.0205 | 0.3030 | 0.0210 |
| CD | 10 | 0.2014 | 0.0189 | 0.1992 | 0.0175 | 0.1998 | 0.0186 | Avg_GRM | 10 | 0.0935 | 0.0189 | 0.0880 | 0.0184 | 0.0915 | 0.0187 |
| | 20 | 0.2255 | 0.0165 | 0.2294 | 0.0164 | 0.2184 | 0.0166 | | 20 | 0.1033 | 0.0210 | 0.0978 | 0.0217 | 0.1055 | 0.0210 |
| | 40 | 0.2723 | 0.0161 | 0.2689 | 0.0166 | 0.2718 | 0.0159 | | 40 | 0.1420 | 0.0222 | 0.1423 | 0.0228 | 0.1410 | 0.0233 |
| | 60 | 0.2591 | 0.0172 | 0.2621 | 0.0165 | 0.2548 | 0.0163 | | 60 | 0.1883 | 0.0204 | 0.1902 | 0.0208 | 0.1912 | 0.0206 |
| | 80 | 0.2993 | 0.0181 | 0.3034 | 0.0185 | 0.2985 | 0.0186 | | 80 | 0.2185 | 0.0196 | 0.2180 | 0.0194 | 0.2213 | 0.0198 |
| | 100 | 0.3054 | 0.0206 | 0.3024 | 0.0205 | 0.3030 | 0.0210 | | 100 | 0.3054 | 0.0206 | 0.3024 | 0.0205 | 0.3030 | 0.0210 |
| CDtarg | 10 | 0.2575 | 0.0216 | 0.2588 | 0.0221 | 0.2543 | 0.0214 | Avg_GRMtarg | 10 | 0.1833 | 0.0203 | 0.1743 | 0.0197 | 0.1824 | 0.0197 |
| | 20 | 0.2884 | 0.0201 | 0.2817 | 0.0202 | 0.2859 | 0.0199 | | 20 | 0.2173 | 0.0227 | 0.2119 | 0.0208 | 0.2141 | 0.0214 |
| | 40 | 0.3138 | 0.0180 | 0.3079 | 0.0193 | 0.3062 | 0.0183 | | 40 | 0.2404 | 0.0201 | 0.2360 | 0.0195 | 0.2329 | 0.0199 |
| | 60 | 0.3188 | 0.0179 | 0.3152 | 0.0179 | 0.3143 | 0.0180 | | 60 | 0.2561 | 0.0184 | 0.2540 | 0.0178 | 0.2518 | 0.0184 |
| | 80 | 0.3229 | 0.0182 | 0.3208 | 0.0180 | 0.3164 | 0.0184 | | 80 | 0.2683 | 0.0184 | 0.2665 | 0.0180 | 0.2623 | 0.0184 |
| | 100 | 0.3054 | 0.0206 | 0.3024 | 0.0205 | 0.3030 | 0.0210 | | 100 | 0.3054 | 0.0206 | 0.3024 | 0.0205 | 0.3030 | 0.0210 |
| OvClustCD | 10 | 0.1823 | 0.0207 | 0.1861 | 0.0217 | 0.1786 | 0.0214 | Avg_GRM_MinMax | 10 | 0.1089 | 0.0257 | 0.1149 | 0.0251 | 0.1088 | 0.0254 |
| | 20 | 0.2269 | 0.0186 | 0.2284 | 0.0188 | 0.2246 | 0.0190 | | 20 | 0.1797 | 0.0237 | 0.1840 | 0.0226 | 0.1845 | 0.0223 |
| | 40 | 0.2546 | 0.0169 | 0.2507 | 0.0166 | 0.2489 | 0.0164 | | 40 | 0.2233 | 0.0172 | 0.2205 | 0.0166 | 0.2237 | 0.0163 |
| | 60 | 0.2892 | 0.0161 | 0.2894 | 0.0157 | 0.2858 | 0.0157 | | 60 | 0.2447 | 0.0193 | 0.2402 | 0.0192 | 0.2410 | 0.0191 |
| | 80 | 0.3056 | 0.0195 | 0.3066 | 0.0191 | 0.3032 | 0.0194 | | 80 | 0.2664 | 0.0188 | 0.2676 | 0.0187 | 0.2617 | 0.0182 |
| | 100 | 0.3054 | 0.0206 | 0.3024 | 0.0205 | 0.3030 | 0.0210 | | 100 | 0.3054 | 0.0206 | 0.3024 | 0.0205 | 0.3030 | 0.0210 |
| OvClustCDtarg | 10 | 0.2349 | 0.0168 | 0.2318 | 0.0161 | 0.2362 | 0.0166 | Avg_GRM_MinMaxtarg | 10 | 0.2082 | 0.0190 | 0.2051 | 0.0196 | 0.2083 | 0.0190 |
| | 20 | 0.2602 | 0.0179 | 0.2548 | 0.0174 | 0.2601 | 0.0181 | | 20 | 0.2304 | 0.0170 | 0.2292 | 0.0168 | 0.2271 | 0.0171 |
| | 40 | 0.2823 | 0.0204 | 0.2820 | 0.0200 | 0.2799 | 0.0199 | | 40 | 0.2524 | 0.0176 | 0.2469 | 0.0174 | 0.2463 | 0.0177 |
| | 60 | 0.2802 | 0.0200 | 0.2852 | 0.0196 | 0.2812 | 0.0196 | | 60 | 0.2686 | 0.0173 | 0.2656 | 0.0173 | 0.2635 | 0.0172 |
| | 80 | 0.2847 | 0.0196 | 0.2882 | 0.0199 | 0.2836 | 0.0188 | | 80 | 0.2719 | 0.0184 | 0.2703 | 0.0176 | 0.2687 | 0.0181 |
| | 100 | 0.3054 | 0.0206 | 0.3024 | 0.0205 | 0.3030 | 0.0210 | | 100 | 0.3054 | 0.0206 | 0.3024 | 0.0205 | 0.3030 | 0.0210 |
| WIClustCD | 10 | 0.1652 | 0.0202 | 0.1711 | 0.0191 | 0.1653 | 0.0198 | Avg_GRM_self | 10 | 0.1715 | 0.0206 | 0.1706 | 0.0200 | 0.1710 | 0.0207 |
| | 20 | 0.1944 | 0.0207 | 0.1944 | 0.0200 | 0.1975 | 0.0205 | | 20 | 0.2044 | 0.0219 | 0.2132 | 0.0206 | 0.2039 | 0.0219 |
| | 40 | 0.2272 | 0.0206 | 0.2245 | 0.0204 | 0.2260 | 0.0206 | | 40 | 0.2492 | 0.0204 | 0.2434 | 0.0200 | 0.2485 | 0.0198 |
| | 60 | 0.2277 | 0.0184 | 0.2250 | 0.0179 | 0.2274 | 0.0182 | | 60 | 0.2554 | 0.0183 | 0.2532 | 0.0175 | 0.2530 | 0.0181 |
| | 80 | 0.2653 | 0.0172 | 0.2641 | 0.0168 | 0.2634 | 0.0166 | | 80 | 0.2775 | 0.0182 | 0.2742 | 0.0183 | 0.2716 | 0.0178 |
| | 100 | 0.3054 | 0.0206 | 0.3024 | 0.0205 | 0.3030 | 0.0210 | | 100 | 0.3054 | 0.0206 | 0.3024 | 0.0205 | 0.3030 | 0.0210 |
| WIClustCDtarg | 10 | 0.2105 | 0.0214 | 0.2111 | 0.0214 | 0.2124 | 0.0205 | PAM | 10 | 0.1725 | 0.0236 | 0.1768 | 0.0243 | 0.1730 | 0.0235 |
| | 20 | 0.2297 | 0.0200 | 0.2321 | 0.0193 | 0.2320 | 0.0198 | | 20 | 0.2266 | 0.0229 | 0.2365 | 0.0224 | 0.2245 | 0.0227 |
| | 40 | 0.2492 | 0.0182 | 0.2533 | 0.0175 | 0.2474 | 0.0182 | | 40 | 0.2502 | 0.0212 | 0.2582 | 0.0196 | 0.2440 | 0.0204 |
| | 60 | 0.2623 | 0.0178 | 0.2638 | 0.0177 | 0.2600 | 0.0179 | | 60 | 0.2857 | 0.0201 | 0.2862 | 0.0194 | 0.2811 | 0.0190 |
| | 80 | 0.2680 | 0.0179 | 0.2658 | 0.0175 | 0.2638 | 0.0180 | | 80 | 0.3172 | 0.0215 | 0.3164 | 0.0210 | 0.3102 | 0.0211 |
| | 100 | 0.3054 | 0.0206 | 0.3024 | 0.0205 | 0.3030 | 0.0210 | | 100 | 0.3054 | 0.0206 | 0.3024 | 0.0205 | 0.3030 | 0.0210 |

**Table S7** Average accuracy and its corresponding standard error of the mean (SEM) across the 40 iterations for all training set optimization methods, models and training set (TRS) sizes (expressed as percentage of the candidate set (CS)) for rice dataset and FT trait. If "targ" is added at the end of the name of a method, it corresponds to targeted optimization. Otherwise, untargeted optimization was performed.

**Rice trait FT**

| Optimization method | TRS size (% of CS) | Average accuracy | | | | | | Optimization method | TRS size (% of CS) | Average accuracy | | | | | |
|---|---|---|---|---|---|---|---|---|---|---|---|---|---|---|---|
| | | GBLUP | | BayesB | | RKHS | | | | GBLUP | | BayesB | | RKHS | |
| | | Mean | SEM | Mean | SEM | Mean | SEM | | | Mean | SEM | Mean | SEM | Mean | SEM |
| RAND | 30 | 0.213 | 0.0229 | 0.224 | 0.0225 | 0.219 | 0.0228 | Rscore | 30 | 0.200 | 0.0238 | 0.207 | 0.0250 | 0.208 | 0.0240 |
| | 55 | 0.287 | 0.0231 | 0.289 | 0.0235 | 0.299 | 0.0232 | | 55 | 0.294 | 0.0249 | 0.294 | 0.0243 | 0.300 | 0.0235 |
| | 110 | 0.419 | 0.0162 | 0.429 | 0.0170 | 0.427 | 0.0163 | | 110 | 0.403 | 0.0192 | 0.416 | 0.0190 | 0.414 | 0.0190 |
| | 165 | 0.477 | 0.0199 | 0.556 | 0.0173 | 0.494 | 0.0199 | | 165 | 0.494 | 0.0201 | 0.557 | 0.0197 | 0.501 | 0.0203 |
| | 220 | 0.505 | 0.0187 | 0.619 | 0.0145 | 0.517 | 0.0189 | | 220 | 0.515 | 0.0164 | 0.618 | 0.0149 | 0.527 | 0.0166 |
| | 277 | 0.533 | 0.0166 | 0.641 | 0.0130 | 0.545 | 0.0166 | | 277 | 0.533 | 0.0166 | 0.641 | 0.0130 | 0.545 | 0.0166 |
| StratSamp | 30 | 0.234 | 0.0204 | 0.227 | 0.0206 | 0.233 | 0.0193 | Rscoretarg | 30 | 0.295 | 0.0257 | 0.302 | 0.0249 | 0.303 | 0.0257 |
| | 55 | 0.308 | 0.0225 | 0.315 | 0.0214 | 0.320 | 0.0219 | | 55 | 0.360 | 0.0219 | 0.362 | 0.0218 | 0.374 | 0.0214 |
| | 110 | 0.396 | 0.0166 | 0.409 | 0.0168 | 0.419 | 0.0171 | | 110 | 0.454 | 0.0204 | 0.478 | 0.0190 | 0.471 | 0.0200 |
| | 165 | 0.467 | 0.0205 | 0.518 | 0.0218 | 0.474 | 0.0200 | | 165 | 0.502 | 0.0193 | 0.560 | 0.0187 | 0.512 | 0.0195 |
| | 220 | 0.502 | 0.0160 | 0.610 | 0.0126 | 0.511 | 0.0166 | | 220 | 0.520 | 0.0183 | 0.619 | 0.0164 | 0.539 | 0.0185 |
| | 277 | 0.533 | 0.0166 | 0.641 | 0.0130 | 0.545 | 0.0166 | | 277 | 0.533 | 0.0166 | 0.641 | 0.0130 | 0.545 | 0.0166 |
| CD | 30 | 0.242 | 0.0207 | 0.249 | 0.0218 | 0.257 | 0.0194 | Avg_GRM | 30 | 0.132 | 0.0193 | 0.130 | 0.0200 | 0.137 | 0.0192 |
| | 55 | 0.382 | 0.0212 | 0.384 | 0.0217 | 0.404 | 0.0219 | | 55 | 0.192 | 0.0232 | 0.187 | 0.0229 | 0.198 | 0.0224 |
| | 110 | 0.448 | 0.0188 | 0.463 | 0.0190 | 0.465 | 0.0192 | | 110 | 0.323 | 0.0212 | 0.329 | 0.0207 | 0.329 | 0.0199 |
| | 165 | 0.486 | 0.0156 | 0.580 | 0.0140 | 0.495 | 0.0161 | | 165 | 0.384 | 0.0164 | 0.459 | 0.0157 | 0.391 | 0.0155 |
| | 220 | 0.529 | 0.0168 | 0.631 | 0.0130 | 0.539 | 0.0164 | | 220 | 0.467 | 0.0161 | 0.583 | 0.0143 | 0.479 | 0.0159 |
| | 277 | 0.533 | 0.0166 | 0.641 | 0.0130 | 0.545 | 0.0166 | | 277 | 0.533 | 0.0166 | 0.641 | 0.0130 | 0.545 | 0.0166 |
| CDtarg | 30 | 0.404 | 0.0227 | 0.408 | 0.0212 | 0.416 | 0.0222 | Avg_GRMtarg | 30 | 0.253 | 0.0212 | 0.269 | 0.0211 | 0.262 | 0.0211 |
| | 55 | 0.424 | 0.0196 | 0.429 | 0.0200 | 0.436 | 0.0198 | | 55 | 0.348 | 0.0222 | 0.358 | 0.0218 | 0.361 | 0.0213 |
| | 110 | 0.493 | 0.0179 | 0.510 | 0.0169 | 0.511 | 0.0173 | | 110 | 0.467 | 0.0190 | 0.496 | 0.0176 | 0.472 | 0.0188 |
| | 165 | 0.526 | 0.0168 | 0.584 | 0.0169 | 0.537 | 0.0167 | | 165 | 0.502 | 0.0166 | 0.568 | 0.0161 | 0.509 | 0.0165 |
| | 220 | 0.536 | 0.0164 | 0.641 | 0.0134 | 0.547 | 0.0170 | | 220 | 0.526 | 0.0176 | 0.632 | 0.0152 | 0.537 | 0.0178 |
| | 277 | 0.533 | 0.0166 | 0.641 | 0.0130 | 0.545 | 0.0166 | | 277 | 0.533 | 0.0166 | 0.641 | 0.0130 | 0.545 | 0.0166 |
| OvClustCD | 30 | 0.276 | 0.0161 | 0.282 | 0.0181 | 0.296 | 0.0174 | Avg_GRM_MinMax | 30 | 0.244 | 0.0252 | 0.257 | 0.0251 | 0.256 | 0.0248 |
| | 55 | 0.382 | 0.0203 | 0.394 | 0.0194 | 0.412 | 0.0201 | | 55 | 0.354 | 0.0235 | 0.357 | 0.0224 | 0.367 | 0.0237 |
| | 110 | 0.438 | 0.0177 | 0.445 | 0.0166 | 0.459 | 0.0182 | | 110 | 0.418 | 0.0168 | 0.429 | 0.0165 | 0.434 | 0.0168 |
| | 165 | 0.481 | 0.0175 | 0.575 | 0.0133 | 0.488 | 0.0175 | | 165 | 0.471 | 0.0201 | 0.523 | 0.0172 | 0.488 | 0.0198 |
| | 220 | 0.530 | 0.0179 | 0.632 | 0.0136 | 0.541 | 0.0179 | | 220 | 0.530 | 0.0169 | 0.625 | 0.0131 | 0.543 | 0.0166 |
| | 277 | 0.533 | 0.0166 | 0.641 | 0.0130 | 0.545 | 0.0166 | | 277 | 0.533 | 0.0166 | 0.641 | 0.0130 | 0.545 | 0.0166 |
| OvClustCDtarg | 30 | 0.386 | 0.0236 | 0.398 | 0.0216 | 0.404 | 0.0228 | Avg_GRM_MinMaxtarg | 30 | 0.346 | 0.0208 | 0.349 | 0.0209 | 0.358 | 0.0208 |
| | 55 | 0.423 | 0.0187 | 0.429 | 0.0189 | 0.442 | 0.0188 | | 55 | 0.375 | 0.0231 | 0.385 | 0.0219 | 0.387 | 0.0231 |
| | 110 | 0.493 | 0.0187 | 0.501 | 0.0177 | 0.505 | 0.0182 | | 110 | 0.475 | 0.0171 | 0.488 | 0.0176 | 0.487 | 0.0185 |
| | 165 | 0.528 | 0.0171 | 0.589 | 0.0169 | 0.536 | 0.0175 | | 165 | 0.498 | 0.0193 | 0.562 | 0.0183 | 0.512 | 0.0189 |
| | 220 | 0.539 | 0.0166 | 0.639 | 0.0143 | 0.550 | 0.0173 | | 220 | 0.518 | 0.0181 | 0.618 | 0.0152 | 0.528 | 0.0184 |
| | 277 | 0.533 | 0.0166 | 0.641 | 0.0130 | 0.545 | 0.0166 | | 277 | 0.533 | 0.0166 | 0.641 | 0.0130 | 0.545 | 0.0166 |
| WIClustCD | 30 | 0.226 | 0.0231 | 0.232 | 0.0227 | 0.239 | 0.0231 | Avg_GRM_self | 30 | 0.239 | 0.0217 | 0.251 | 0.0213 | 0.255 | 0.0215 |
| | 55 | 0.360 | 0.0189 | 0.369 | 0.0182 | 0.379 | 0.0182 | | 55 | 0.347 | 0.0213 | 0.361 | 0.0209 | 0.365 | 0.0211 |
| | 110 | 0.432 | 0.0210 | 0.441 | 0.0202 | 0.454 | 0.0205 | | 110 | 0.453 | 0.0200 | 0.463 | 0.0203 | 0.472 | 0.0190 |
| | 165 | 0.476 | 0.0183 | 0.562 | 0.0144 | 0.485 | 0.0178 | | 165 | 0.499 | 0.0161 | 0.538 | 0.0166 | 0.513 | 0.0163 |
| | 220 | 0.526 | 0.0173 | 0.624 | 0.0139 | 0.536 | 0.0170 | | 220 | 0.516 | 0.0176 | 0.620 | 0.0145 | 0.528 | 0.0177 |
| | 277 | 0.533 | 0.0166 | 0.641 | 0.0130 | 0.545 | 0.0166 | | 277 | 0.533 | 0.0166 | 0.641 | 0.0130 | 0.545 | 0.0166 |
| WIClustCDtarg | 30 | 0.372 | 0.0222 | 0.389 | 0.0219 | 0.390 | 0.0219 | PAM | 30 | 0.188 | 0.0251 | 0.192 | 0.0250 | 0.215 | 0.0252 |
| | 55 | 0.421 | 0.0194 | 0.432 | 0.0191 | 0.443 | 0.0195 | | 55 | 0.321 | 0.0199 | 0.328 | 0.0206 | 0.354 | 0.0199 |
| | 110 | 0.488 | 0.0183 | 0.500 | 0.0172 | 0.500 | 0.0179 | | 110 | 0.392 | 0.0186 | 0.405 | 0.0184 | 0.421 | 0.0189 |
| | 165 | 0.525 | 0.0165 | 0.593 | 0.0165 | 0.532 | 0.0168 | | 165 | 0.492 | 0.0168 | 0.511 | 0.0157 | 0.510 | 0.0177 |
| | 220 | 0.538 | 0.0181 | 0.641 | 0.0132 | 0.547 | 0.0185 | | 220 | 0.523 | 0.0181 | 0.626 | 0.0142 | 0.540 | 0.0183 |
| | 277 | 0.533 | 0.0166 | 0.641 | 0.0130 | 0.545 | 0.0166 | | 277 | 0.533 | 0.0166 | 0.641 | 0.0130 | 0.545 | 0.0166 |

**Table S8** Average accuracy and its corresponding standard error of the mean (SEM) across the 40 iterations for all training set optimization methods, models and training set (TRS) sizes (expressed as percentage of the candidate set (CS)) for rice dataset and HT trait. If "targ" is added at the end of the name of a method, it corresponds to targeted optimization. Otherwise, untargeted optimization was performed.

**Rice trait HT**

| | | Average accuracy | | | | | | | | Average accuracy | | | | | |
|---|---|---|---|---|---|---|---|---|---|---|---|---|---|---|---|
| | | GBLUP | | BayesB | | RKHS | | | | GBLUP | | BayesB | | RKHS | |
| Optimization method | TRS size (% of CS) | Mean | SEM | Mean | SEM | Mean | SEM | Optimization method | TRS size (% of CS) | Mean | SEM | Mean | SEM | Mean | SEM |
| RAND | 30 | 0.1927 | 0.0237 | 0.1971 | 0.0238 | 0.1997 | 0.0239 | Rscore | 30 | 0.1922 | 0.0266 | 0.2042 | 0.0260 | 0.1996 | 0.0268 |
| | 55 | 0.2458 | 0.0226 | 0.2510 | 0.0218 | 0.2515 | 0.0217 | | 55 | 0.2562 | 0.0232 | 0.2640 | 0.0224 | 0.2655 | 0.0237 |
| | 110 | 0.3157 | 0.0200 | 0.3212 | 0.0204 | 0.3260 | 0.0206 | | 110 | 0.3271 | 0.0227 | 0.3330 | 0.0237 | 0.3429 | 0.0233 |
| | 165 | 0.3646 | 0.0192 | 0.3717 | 0.0192 | 0.3783 | 0.0201 | | 165 | 0.3665 | 0.0187 | 0.3726 | 0.0190 | 0.3847 | 0.0196 |
| | 220 | 0.3972 | 0.0179 | 0.4023 | 0.0179 | 0.4126 | 0.0179 | | 220 | 0.3953 | 0.0195 | 0.3997 | 0.0191 | 0.4197 | 0.0197 |
| | 277 | 0.4243 | 0.0186 | 0.4309 | 0.0187 | 0.4440 | 0.0191 | | 277 | 0.4243 | 0.0186 | 0.4309 | 0.0187 | 0.4440 | 0.0191 |
| StratSamp | 30 | 0.1897 | 0.0214 | 0.1840 | 0.0225 | 0.1995 | 0.0215 | Rscoretarg | 30 | 0.3234 | 0.0182 | 0.3344 | 0.0167 | 0.3321 | 0.0182 |
| | 55 | 0.2687 | 0.0226 | 0.2771 | 0.0229 | 0.2767 | 0.0222 | | 55 | 0.3432 | 0.0204 | 0.3477 | 0.0199 | 0.3573 | 0.0201 |
| | 110 | 0.3130 | 0.0202 | 0.3282 | 0.0213 | 0.3290 | 0.0200 | | 110 | 0.3805 | 0.0200 | 0.3852 | 0.0200 | 0.3982 | 0.0198 |
| | 165 | 0.3533 | 0.0210 | 0.3569 | 0.0226 | 0.3682 | 0.0218 | | 165 | 0.4047 | 0.0202 | 0.4155 | 0.0199 | 0.4292 | 0.0200 |
| | 220 | 0.3921 | 0.0200 | 0.3975 | 0.0198 | 0.4106 | 0.0204 | | 220 | 0.4092 | 0.0196 | 0.4160 | 0.0194 | 0.4337 | 0.0198 |
| | 277 | 0.4243 | 0.0186 | 0.4309 | 0.0187 | 0.4440 | 0.0191 | | 277 | 0.4243 | 0.0186 | 0.4309 | 0.0187 | 0.4440 | 0.0191 |
| CD | 30 | 0.2789 | 0.0232 | 0.2732 | 0.0239 | 0.2881 | 0.0236 | Avg_GRM | 30 | 0.0777 | 0.0228 | 0.0808 | 0.0222 | 0.0846 | 0.0223 |
| | 55 | 0.3190 | 0.0213 | 0.3280 | 0.0203 | 0.3372 | 0.0204 | | 55 | 0.1363 | 0.0217 | 0.1365 | 0.0217 | 0.1432 | 0.0208 |
| | 110 | 0.3357 | 0.0219 | 0.3466 | 0.0223 | 0.3501 | 0.0218 | | 110 | 0.2280 | 0.0200 | 0.2368 | 0.0220 | 0.2279 | 0.0197 |
| | 165 | 0.3534 | 0.0220 | 0.3704 | 0.0222 | 0.3627 | 0.0224 | | 165 | 0.2843 | 0.0201 | 0.2936 | 0.0215 | 0.2885 | 0.0213 |
| | 220 | 0.3822 | 0.0207 | 0.3961 | 0.0206 | 0.4039 | 0.0209 | | 220 | 0.3398 | 0.0209 | 0.3471 | 0.0210 | 0.3451 | 0.0217 |
| | 277 | 0.4243 | 0.0186 | 0.4309 | 0.0187 | 0.4440 | 0.0191 | | 277 | 0.4243 | 0.0186 | 0.4309 | 0.0187 | 0.4440 | 0.0191 |
| CDtarg | 30 | 0.4063 | 0.0162 | 0.4104 | 0.0169 | 0.4214 | 0.0169 | Avg_GRMtarg | 30 | 0.2754 | 0.0238 | 0.2788 | 0.0229 | 0.2844 | 0.0238 |
| | 55 | 0.4385 | 0.0158 | 0.4366 | 0.0160 | 0.4529 | 0.0163 | | 55 | 0.2909 | 0.0247 | 0.2976 | 0.0240 | 0.3009 | 0.0239 |
| | 110 | 0.4195 | 0.0184 | 0.4222 | 0.0184 | 0.4407 | 0.0185 | | 110 | 0.3610 | 0.0207 | 0.3647 | 0.0210 | 0.3743 | 0.0201 |
| | 165 | 0.4232 | 0.0192 | 0.4301 | 0.0187 | 0.4418 | 0.0194 | | 165 | 0.3862 | 0.0199 | 0.3961 | 0.0203 | 0.4058 | 0.0194 |
| | 220 | 0.4154 | 0.0190 | 0.4214 | 0.0190 | 0.4383 | 0.0192 | | 220 | 0.4116 | 0.0206 | 0.4202 | 0.0203 | 0.4294 | 0.0203 |
| | 277 | 0.4243 | 0.0186 | 0.4309 | 0.0187 | 0.4440 | 0.0191 | | 277 | 0.4243 | 0.0186 | 0.4309 | 0.0187 | 0.4440 | 0.0191 |
| OvClustCD | 30 | 0.2938 | 0.0223 | 0.2936 | 0.0227 | 0.3025 | 0.0221 | Avg_GRM_MinMax | 30 | 0.2219 | 0.0210 | 0.2243 | 0.0212 | 0.2289 | 0.0213 |
| | 55 | 0.3291 | 0.0195 | 0.3325 | 0.0185 | 0.3425 | 0.0199 | | 55 | 0.2687 | 0.0170 | 0.2737 | 0.0165 | 0.2852 | 0.0183 |
| | 110 | 0.3407 | 0.0205 | 0.3489 | 0.0206 | 0.3573 | 0.0211 | | 110 | 0.3096 | 0.0161 | 0.3098 | 0.0162 | 0.3245 | 0.0164 |
| | 165 | 0.3541 | 0.0223 | 0.3674 | 0.0227 | 0.3648 | 0.0220 | | 165 | 0.3668 | 0.0189 | 0.3708 | 0.0188 | 0.3835 | 0.0183 |
| | 220 | 0.3801 | 0.0199 | 0.3944 | 0.0206 | 0.4027 | 0.0199 | | 220 | 0.4060 | 0.0182 | 0.4108 | 0.0179 | 0.4239 | 0.0183 |
| | 277 | 0.4243 | 0.0186 | 0.4309 | 0.0187 | 0.4440 | 0.0191 | | 277 | 0.4243 | 0.0186 | 0.4309 | 0.0187 | 0.4440 | 0.0191 |
| OvClustCDtarg | 30 | 0.4057 | 0.0176 | 0.4086 | 0.0171 | 0.4163 | 0.0171 | Avg_GRM_MinMaxtarg | 30 | 0.2449 | 0.0234 | 0.2475 | 0.0246 | 0.2514 | 0.0243 |
| | 55 | 0.4315 | 0.0162 | 0.4332 | 0.0168 | 0.4507 | 0.0164 | | 55 | 0.3225 | 0.0205 | 0.3226 | 0.0218 | 0.3389 | 0.0214 |
| | 110 | 0.4218 | 0.0168 | 0.4259 | 0.0167 | 0.4422 | 0.0172 | | 110 | 0.3792 | 0.0212 | 0.3807 | 0.0211 | 0.3982 | 0.0204 |
| | 165 | 0.4268 | 0.0177 | 0.4298 | 0.0183 | 0.4437 | 0.0184 | | 165 | 0.4007 | 0.0204 | 0.4056 | 0.0207 | 0.4197 | 0.0205 |
| | 220 | 0.4181 | 0.0182 | 0.4239 | 0.0181 | 0.4420 | 0.0186 | | 220 | 0.4209 | 0.0197 | 0.4294 | 0.0199 | 0.4385 | 0.0201 |
| | 277 | 0.4243 | 0.0186 | 0.4309 | 0.0187 | 0.4440 | 0.0191 | | 277 | 0.4243 | 0.0186 | 0.4309 | 0.0187 | 0.4440 | 0.0191 |
| WIClustCD | 30 | 0.2741 | 0.0211 | 0.2768 | 0.0203 | 0.2829 | 0.0216 | Avg_GRM_self | 30 | 0.2240 | 0.0225 | 0.2193 | 0.0219 | 0.2277 | 0.0228 |
| | 55 | 0.3305 | 0.0193 | 0.3386 | 0.0178 | 0.3407 | 0.0195 | | 55 | 0.3099 | 0.0231 | 0.3154 | 0.0236 | 0.3170 | 0.0229 |
| | 110 | 0.3130 | 0.0229 | 0.3268 | 0.0225 | 0.3262 | 0.0235 | | 110 | 0.3511 | 0.0179 | 0.3560 | 0.0176 | 0.3705 | 0.0194 |
| | 165 | 0.3490 | 0.0225 | 0.3603 | 0.0223 | 0.3576 | 0.0222 | | 165 | 0.3913 | 0.0173 | 0.3964 | 0.0171 | 0.4080 | 0.0187 |
| | 220 | 0.3799 | 0.0214 | 0.3946 | 0.0216 | 0.4011 | 0.0215 | | 220 | 0.4068 | 0.0190 | 0.4136 | 0.0190 | 0.4281 | 0.0190 |
| | 277 | 0.4243 | 0.0186 | 0.4309 | 0.0187 | 0.4440 | 0.0191 | | 277 | 0.4243 | 0.0186 | 0.4309 | 0.0187 | 0.4440 | 0.0191 |
| WIClustCDtarg | 30 | 0.3961 | 0.0168 | 0.3998 | 0.0166 | 0.4085 | 0.0164 | PAM | 30 | 0.2153 | 0.0228 | 0.2211 | 0.0210 | 0.2275 | 0.0220 |
| | 55 | 0.4178 | 0.0150 | 0.4201 | 0.0160 | 0.4354 | 0.0157 | | 55 | 0.2683 | 0.0203 | 0.2795 | 0.0200 | 0.2931 | 0.0212 |
| | 110 | 0.4175 | 0.0170 | 0.4174 | 0.0175 | 0.4352 | 0.0171 | | 110 | 0.3289 | 0.0197 | 0.3431 | 0.0198 | 0.3505 | 0.0196 |
| | 165 | 0.4206 | 0.0169 | 0.4230 | 0.0167 | 0.4386 | 0.0176 | | 165 | 0.3529 | 0.0179 | 0.3685 | 0.0193 | 0.3668 | 0.0185 |
| | 220 | 0.4208 | 0.0184 | 0.4273 | 0.0185 | 0.4419 | 0.0188 | | 220 | 0.3870 | 0.0193 | 0.3955 | 0.0196 | 0.3979 | 0.0199 |
| | 277 | 0.4243 | 0.0186 | 0.4309 | 0.0187 | 0.4440 | 0.0191 | | 277 | 0.4243 | 0.0186 | 0.4309 | 0.0187 | 0.4440 | 0.0191 |

**Table S9** Average accuracy and its corresponding standard error of the mean (SEM) across the 40 iterations for all training set optimization methods, models and training set (TRS) sizes (expressed as percentage of the candidate set (CS)) for rice dataset and YLD trait. If "targ" is added at the end of the name of a method, it corresponds to targeted optimization. Otherwise, untargeted optimization was performed.

**Rice trait YLD**

| | | Average accuracy | | | | | | | | Average accuracy | | | | | |
|---|---|---|---|---|---|---|---|---|---|---|---|---|---|---|---|
| | | GBLUP | | BayesB | | RKHS | | | | GBLUP | | BayesB | | RKHS | |
| Optimization method | TRS size (% of CS) | Mean | SEM | Mean | SEM | Mean | SEM | Optimization method | TRS size (% of CS) | Mean | SEM | Mean | SEM | Mean | SEM |
| RAND | 30 | 0.0576 | 0.0224 | 0.0705 | 0.0223 | 0.0662 | 0.0224 | Rscore | 30 | 0.0852 | 0.0265 | 0.1065 | 0.0266 | 0.0966 | 0.0260 |
| | 55 | 0.1224 | 0.0231 | 0.1395 | 0.0223 | 0.1397 | 0.0235 | | 55 | 0.1672 | 0.0229 | 0.1829 | 0.0218 | 0.1852 | 0.0239 |
| | 110 | 0.2087 | 0.0231 | 0.2210 | 0.0231 | 0.2397 | 0.0217 | | 110 | 0.1950 | 0.0206 | 0.2296 | 0.0201 | 0.2247 | 0.0211 |
| | 165 | 0.2689 | 0.0226 | 0.2866 | 0.0228 | 0.3028 | 0.0239 | | 165 | 0.2999 | 0.0186 | 0.3134 | 0.0191 | 0.3336 | 0.0194 |
| | 220 | 0.3299 | 0.0224 | 0.3362 | 0.0233 | 0.3627 | 0.0230 | | 220 | 0.3214 | 0.0213 | 0.3315 | 0.0217 | 0.3543 | 0.0218 |
| | 277 | 0.3606 | 0.0208 | 0.3634 | 0.0212 | 0.3947 | 0.0205 | | 277 | 0.3606 | 0.0208 | 0.3634 | 0.0212 | 0.3947 | 0.0205 |
| StratSamp | 30 | 0.0709 | 0.0184 | 0.0714 | 0.0195 | 0.0853 | 0.0188 | Rscoretarg | 30 | 0.1670 | 0.0256 | 0.1759 | 0.0256 | 0.1749 | 0.0255 |
| | 55 | 0.1350 | 0.0220 | 0.1483 | 0.0223 | 0.1461 | 0.0219 | | 55 | 0.2117 | 0.0242 | 0.2255 | 0.0242 | 0.2301 | 0.0248 |
| | 110 | 0.1935 | 0.0248 | 0.2200 | 0.0237 | 0.2351 | 0.0232 | | 110 | 0.2599 | 0.0230 | 0.2766 | 0.0224 | 0.2888 | 0.0237 |
| | 165 | 0.2827 | 0.0214 | 0.2988 | 0.0214 | 0.3159 | 0.0222 | | 165 | 0.2776 | 0.0251 | 0.2932 | 0.0249 | 0.3140 | 0.0245 |
| | 220 | 0.3221 | 0.0175 | 0.3251 | 0.0184 | 0.3537 | 0.0182 | | 220 | 0.3222 | 0.0196 | 0.3329 | 0.0205 | 0.3626 | 0.0193 |
| | 277 | 0.3606 | 0.0208 | 0.3634 | 0.0212 | 0.3947 | 0.0205 | | 277 | 0.3606 | 0.0208 | 0.3634 | 0.0212 | 0.3947 | 0.0205 |
| CD | 30 | 0.1074 | 0.0238 | 0.1320 | 0.0242 | 0.1191 | 0.0240 | Avg_GRM | 30 | 0.0421 | 0.0252 | 0.0338 | 0.0261 | 0.0518 | 0.0255 |
| | 55 | 0.2170 | 0.0268 | 0.2393 | 0.0274 | 0.2536 | 0.0265 | | 55 | 0.0852 | 0.0240 | 0.0792 | 0.0240 | 0.1015 | 0.0239 |
| | 110 | 0.2774 | 0.0238 | 0.2920 | 0.0235 | 0.3229 | 0.0246 | | 110 | 0.1446 | 0.0232 | 0.1508 | 0.0229 | 0.1648 | 0.0232 |
| | 165 | 0.3255 | 0.0191 | 0.3370 | 0.0191 | 0.3487 | 0.0188 | | 165 | 0.1752 | 0.0203 | 0.1822 | 0.0202 | 0.1916 | 0.0207 |
| | 220 | 0.3301 | 0.0185 | 0.3409 | 0.0197 | 0.3544 | 0.0190 | | 220 | 0.2649 | 0.0191 | 0.2721 | 0.0201 | 0.2914 | 0.0206 |
| | 277 | 0.3606 | 0.0208 | 0.3634 | 0.0212 | 0.3947 | 0.0205 | | 277 | 0.3606 | 0.0208 | 0.3634 | 0.0212 | 0.3947 | 0.0205 |
| CDtarg | 30 | 0.2483 | 0.0271 | 0.2499 | 0.0270 | 0.2638 | 0.0261 | Avg_GRMtarg | 30 | 0.1459 | 0.0219 | 0.1518 | 0.0229 | 0.1568 | 0.0218 |
| | 55 | 0.2779 | 0.0254 | 0.2920 | 0.0256 | 0.3144 | 0.0235 | | 55 | 0.2001 | 0.0263 | 0.2093 | 0.0268 | 0.2122 | 0.0255 |
| | 110 | 0.3133 | 0.0237 | 0.3216 | 0.0234 | 0.3453 | 0.0218 | | 110 | 0.2902 | 0.0212 | 0.3036 | 0.0210 | 0.3088 | 0.0218 |
| | 165 | 0.3457 | 0.0215 | 0.3547 | 0.0217 | 0.3839 | 0.0205 | | 165 | 0.3114 | 0.0233 | 0.3253 | 0.0237 | 0.3368 | 0.0226 |
| | 220 | 0.3555 | 0.0213 | 0.3632 | 0.0216 | 0.3923 | 0.0206 | | 220 | 0.3391 | 0.0228 | 0.3473 | 0.0231 | 0.3653 | 0.0226 |
| | 277 | 0.3606 | 0.0208 | 0.3634 | 0.0212 | 0.3947 | 0.0205 | | 277 | 0.3606 | 0.0208 | 0.3634 | 0.0212 | 0.3947 | 0.0205 |
| OvClustCD | 30 | 0.1238 | 0.0228 | 0.1429 | 0.0220 | 0.1376 | 0.0229 | Avg_GRM_MinMax | 30 | 0.1010 | 0.0221 | 0.1148 | 0.0207 | 0.1115 | 0.0229 |
| | 55 | 0.2315 | 0.0216 | 0.2503 | 0.0223 | 0.2649 | 0.0221 | | 55 | 0.1592 | 0.0237 | 0.1874 | 0.0232 | 0.1923 | 0.0231 |
| | 110 | 0.2746 | 0.0222 | 0.2854 | 0.0231 | 0.3098 | 0.0228 | | 110 | 0.2116 | 0.0263 | 0.2487 | 0.0267 | 0.2498 | 0.0265 |
| | 165 | 0.3095 | 0.0202 | 0.3280 | 0.0214 | 0.3328 | 0.0214 | | 165 | 0.2937 | 0.0219 | 0.3199 | 0.0221 | 0.3402 | 0.0220 |
| | 220 | 0.3198 | 0.0180 | 0.3312 | 0.0194 | 0.3427 | 0.0179 | | 220 | 0.3370 | 0.0217 | 0.3419 | 0.0219 | 0.3724 | 0.0211 |
| | 277 | 0.3606 | 0.0208 | 0.3634 | 0.0212 | 0.3947 | 0.0205 | | 277 | 0.3606 | 0.0208 | 0.3634 | 0.0212 | 0.3947 | 0.0205 |
| OvClustCDtarg | 30 | 0.2526 | 0.0264 | 0.2587 | 0.0263 | 0.2651 | 0.0254 | Avg_GRM_MinMaxtarg | 30 | 0.1692 | 0.0223 | 0.1726 | 0.0228 | 0.1830 | 0.0226 |
| | 55 | 0.2814 | 0.0241 | 0.2933 | 0.0261 | 0.3159 | 0.0234 | | 55 | 0.2316 | 0.0270 | 0.2479 | 0.0249 | 0.2499 | 0.0263 |
| | 110 | 0.3172 | 0.0244 | 0.3265 | 0.0236 | 0.3512 | 0.0220 | | 110 | 0.2802 | 0.0230 | 0.2977 | 0.0230 | 0.3110 | 0.0226 |
| | 165 | 0.3559 | 0.0214 | 0.3658 | 0.0213 | 0.3895 | 0.0204 | | 165 | 0.3143 | 0.0230 | 0.3311 | 0.0232 | 0.3454 | 0.0228 |
| | 220 | 0.3531 | 0.0213 | 0.3616 | 0.0217 | 0.3933 | 0.0206 | | 220 | 0.3392 | 0.0217 | 0.3451 | 0.0222 | 0.3739 | 0.0207 |
| | 277 | 0.3606 | 0.0208 | 0.3634 | 0.0212 | 0.3947 | 0.0205 | | 277 | 0.3606 | 0.0208 | 0.3634 | 0.0212 | 0.3947 | 0.0205 |
| WIClustCD | 30 | 0.1036 | 0.0219 | 0.1326 | 0.0220 | 0.1197 | 0.0224 | Avg_GRM_self | 30 | 0.1485 | 0.0256 | 0.1662 | 0.0254 | 0.1635 | 0.0264 |
| | 55 | 0.1863 | 0.0267 | 0.2149 | 0.0254 | 0.2242 | 0.0257 | | 55 | 0.2050 | 0.0242 | 0.2299 | 0.0247 | 0.2202 | 0.0249 |
| | 110 | 0.2815 | 0.0218 | 0.3047 | 0.0228 | 0.3262 | 0.0211 | | 110 | 0.2302 | 0.0249 | 0.2540 | 0.0242 | 0.2752 | 0.0257 |
| | 165 | 0.3011 | 0.0194 | 0.3254 | 0.0210 | 0.3285 | 0.0200 | | 165 | 0.3056 | 0.0220 | 0.3188 | 0.0220 | 0.3423 | 0.0202 |
| | 220 | 0.3321 | 0.0196 | 0.3459 | 0.0212 | 0.3580 | 0.0192 | | 220 | 0.3334 | 0.0208 | 0.3400 | 0.0215 | 0.3730 | 0.0204 |
| | 277 | 0.3606 | 0.0208 | 0.3634 | 0.0212 | 0.3947 | 0.0205 | | 277 | 0.3606 | 0.0208 | 0.3634 | 0.0212 | 0.3947 | 0.0205 |
| WIClustCDtarg | 30 | 0.2375 | 0.0276 | 0.2371 | 0.0281 | 0.2505 | 0.0260 | PAM | 30 | 0.1426 | 0.0212 | 0.1525 | 0.0211 | 0.1513 | 0.0217 |
| | 55 | 0.2827 | 0.0254 | 0.2900 | 0.0255 | 0.3125 | 0.0234 | | 55 | 0.2032 | 0.0194 | 0.2131 | 0.0199 | 0.2285 | 0.0210 |
| | 110 | 0.3026 | 0.0231 | 0.3173 | 0.0226 | 0.3424 | 0.0220 | | 110 | 0.2287 | 0.0181 | 0.2536 | 0.0205 | 0.2824 | 0.0201 |
| | 165 | 0.3315 | 0.0216 | 0.3418 | 0.0223 | 0.3685 | 0.0208 | | 165 | 0.2859 | 0.0200 | 0.3159 | 0.0211 | 0.3446 | 0.0205 |
| | 220 | 0.3486 | 0.0217 | 0.3560 | 0.0222 | 0.3853 | 0.0213 | | 220 | 0.3294 | 0.0209 | 0.3452 | 0.0226 | 0.3841 | 0.0208 |
| | 277 | 0.3606 | 0.0208 | 0.3634 | 0.0212 | 0.3947 | 0.0205 | | 277 | 0.3606 | 0.0208 | 0.3634 | 0.0212 | 0.3947 | 0.0205 |

**Table S10** Average accuracy and its corresponding standard error of the mean (SEM) across the 40 iterations for all training set optimization methods, models and training set (TRS) sizes (expressed as percentage of the candidate set (CS)) for rice dataset and the simulated trait. If "targ" is added at the end of the name of a method, it corresponds to targeted optimization. Otherwise, untargeted optimization was performed.

**Rice trait simulated1**

| Optimization method | TRS size (% of CS) | Average accuracy GBLUP Mean | GBLUP SEM | BayesB Mean | BayesB SEM | RKHS Mean | RKHS SEM | Optimization method | TRS size (% of CS) | Average accuracy GBLUP Mean | GBLUP SEM | BayesB Mean | BayesB SEM | RKHS Mean | RKHS SEM |
|---|---|---|---|---|---|---|---|---|---|---|---|---|---|---|---|
| RAND | 30 | 0.239 | 0.0273 | 0.248 | 0.0273 | 0.242 | 0.0266 | Rscore | 30 | 0.228 | 0.0242 | 0.227 | 0.0228 | 0.231 | 0.0236 |
| | 55 | 0.287 | 0.0188 | 0.278 | 0.0207 | 0.280 | 0.0195 | | 55 | 0.312 | 0.0262 | 0.308 | 0.0275 | 0.309 | 0.0265 |
| | 110 | 0.343 | 0.0239 | 0.343 | 0.0238 | 0.341 | 0.0238 | | 110 | 0.372 | 0.0191 | 0.369 | 0.0196 | 0.365 | 0.0192 |
| | 165 | 0.403 | 0.0183 | 0.406 | 0.0179 | 0.400 | 0.0175 | | 165 | 0.410 | 0.0226 | 0.413 | 0.0225 | 0.406 | 0.0229 |
| | 220 | 0.421 | 0.0208 | 0.422 | 0.0210 | 0.419 | 0.0210 | | 220 | 0.426 | 0.0210 | 0.427 | 0.0210 | 0.421 | 0.0203 |
| | 277 | 0.486 | 0.0213 | 0.486 | 0.0211 | 0.484 | 0.0213 | | 277 | 0.486 | 0.0213 | 0.486 | 0.0211 | 0.484 | 0.0213 |
| StratSamp | 30 | 0.236 | 0.0242 | 0.242 | 0.0235 | 0.240 | 0.0241 | Rscoretarg | 30 | 0.315 | 0.0245 | 0.322 | 0.0247 | 0.306 | 0.0239 |
| | 55 | 0.263 | 0.0241 | 0.273 | 0.0215 | 0.261 | 0.0241 | | 55 | 0.345 | 0.0257 | 0.359 | 0.0247 | 0.344 | 0.0250 |
| | 110 | 0.327 | 0.0226 | 0.327 | 0.0234 | 0.328 | 0.0227 | | 110 | 0.418 | 0.0193 | 0.415 | 0.0187 | 0.416 | 0.0193 |
| | 165 | 0.369 | 0.0178 | 0.366 | 0.0181 | 0.365 | 0.0188 | | 165 | 0.434 | 0.0214 | 0.432 | 0.0214 | 0.430 | 0.0217 |
| | 220 | 0.401 | 0.0199 | 0.401 | 0.0202 | 0.400 | 0.0205 | | 220 | 0.461 | 0.0200 | 0.457 | 0.0197 | 0.458 | 0.0198 |
| | 277 | 0.486 | 0.0213 | 0.486 | 0.0211 | 0.484 | 0.0213 | | 277 | 0.486 | 0.0213 | 0.486 | 0.0211 | 0.484 | 0.0213 |
| CD | 30 | 0.244 | 0.0258 | 0.245 | 0.0268 | 0.240 | 0.0259 | Avg_GRM | 30 | 0.184 | 0.0275 | 0.177 | 0.0288 | 0.179 | 0.0279 |
| | 55 | 0.331 | 0.0192 | 0.330 | 0.0180 | 0.329 | 0.0183 | | 55 | 0.245 | 0.0268 | 0.235 | 0.0273 | 0.241 | 0.0273 |
| | 110 | 0.348 | 0.0248 | 0.354 | 0.0241 | 0.344 | 0.0238 | | 110 | 0.329 | 0.0224 | 0.327 | 0.0222 | 0.325 | 0.0232 |
| | 165 | 0.378 | 0.0256 | 0.382 | 0.0248 | 0.376 | 0.0245 | | 165 | 0.368 | 0.0213 | 0.367 | 0.0210 | 0.366 | 0.0221 |
| | 220 | 0.433 | 0.0198 | 0.437 | 0.0195 | 0.429 | 0.0196 | | 220 | 0.397 | 0.0231 | 0.397 | 0.0230 | 0.398 | 0.0235 |
| | 277 | 0.486 | 0.0213 | 0.486 | 0.0211 | 0.484 | 0.0213 | | 277 | 0.486 | 0.0213 | 0.486 | 0.0211 | 0.484 | 0.0213 |
| CDtarg | 30 | 0.313 | 0.0262 | 0.319 | 0.0240 | 0.312 | 0.0257 | Avg_GRMtarg | 30 | 0.218 | 0.0249 | 0.222 | 0.0238 | 0.216 | 0.0250 |
| | 55 | 0.346 | 0.0217 | 0.353 | 0.0217 | 0.350 | 0.0213 | | 55 | 0.297 | 0.0192 | 0.300 | 0.0178 | 0.289 | 0.0201 |
| | 110 | 0.391 | 0.0174 | 0.390 | 0.0177 | 0.394 | 0.0172 | | 110 | 0.356 | 0.0248 | 0.360 | 0.0238 | 0.351 | 0.0255 |
| | 165 | 0.427 | 0.0175 | 0.423 | 0.0171 | 0.426 | 0.0171 | | 165 | 0.402 | 0.0231 | 0.402 | 0.0220 | 0.399 | 0.0233 |
| | 220 | 0.439 | 0.0183 | 0.436 | 0.0184 | 0.438 | 0.0180 | | 220 | 0.428 | 0.0213 | 0.430 | 0.0205 | 0.427 | 0.0211 |
| | 277 | 0.486 | 0.0213 | 0.486 | 0.0211 | 0.484 | 0.0213 | | 277 | 0.486 | 0.0213 | 0.486 | 0.0211 | 0.484 | 0.0213 |
| OvClustCD | 30 | 0.249 | 0.0250 | 0.255 | 0.0254 | 0.254 | 0.0244 | Avg_GRM_MinMax | 30 | 0.241 | 0.0262 | 0.245 | 0.0260 | 0.241 | 0.0262 |
| | 55 | 0.307 | 0.0260 | 0.309 | 0.0246 | 0.304 | 0.0251 | | 55 | 0.296 | 0.0205 | 0.297 | 0.0203 | 0.292 | 0.0201 |
| | 110 | 0.376 | 0.0238 | 0.375 | 0.0237 | 0.377 | 0.0237 | | 110 | 0.342 | 0.0254 | 0.345 | 0.0233 | 0.342 | 0.0255 |
| | 165 | 0.413 | 0.0227 | 0.414 | 0.0226 | 0.408 | 0.0228 | | 165 | 0.411 | 0.0198 | 0.411 | 0.0193 | 0.407 | 0.0198 |
| | 220 | 0.423 | 0.0227 | 0.421 | 0.0223 | 0.414 | 0.0220 | | 220 | 0.430 | 0.0207 | 0.431 | 0.0204 | 0.430 | 0.0202 |
| | 277 | 0.486 | 0.0213 | 0.486 | 0.0211 | 0.484 | 0.0213 | | 277 | 0.486 | 0.0213 | 0.486 | 0.0211 | 0.484 | 0.0213 |
| OvClustCDtarg | 30 | 0.362 | 0.0227 | 0.365 | 0.0229 | 0.364 | 0.0220 | Avg_GRM_MinMaxtarg | 30 | 0.252 | 0.0271 | 0.251 | 0.0281 | 0.253 | 0.0269 |
| | 55 | 0.388 | 0.0224 | 0.394 | 0.0231 | 0.386 | 0.0220 | | 55 | 0.323 | 0.0233 | 0.322 | 0.0227 | 0.328 | 0.0228 |
| | 110 | 0.447 | 0.0203 | 0.453 | 0.0208 | 0.443 | 0.0205 | | 110 | 0.375 | 0.0229 | 0.376 | 0.0227 | 0.373 | 0.0221 |
| | 165 | 0.468 | 0.0195 | 0.472 | 0.0196 | 0.467 | 0.0198 | | 165 | 0.409 | 0.0211 | 0.411 | 0.0206 | 0.405 | 0.0205 |
| | 220 | 0.486 | 0.0193 | 0.488 | 0.0194 | 0.482 | 0.0195 | | 220 | 0.432 | 0.0198 | 0.433 | 0.0197 | 0.430 | 0.0197 |
| | 277 | 0.486 | 0.0213 | 0.486 | 0.0211 | 0.484 | 0.0213 | | 277 | 0.486 | 0.0213 | 0.486 | 0.0211 | 0.484 | 0.0213 |
| WIClustCD | 30 | 0.268 | 0.0287 | 0.271 | 0.0290 | 0.265 | 0.0286 | Avg_GRM_self | 30 | 0.260 | 0.0286 | 0.273 | 0.0287 | 0.262 | 0.0281 |
| | 55 | 0.358 | 0.0230 | 0.357 | 0.0220 | 0.353 | 0.0233 | | 55 | 0.272 | 0.0295 | 0.283 | 0.0287 | 0.277 | 0.0286 |
| | 110 | 0.383 | 0.0239 | 0.382 | 0.0237 | 0.382 | 0.0239 | | 110 | 0.366 | 0.0227 | 0.373 | 0.0212 | 0.364 | 0.0228 |
| | 165 | 0.408 | 0.0216 | 0.408 | 0.0210 | 0.406 | 0.0215 | | 165 | 0.388 | 0.0224 | 0.394 | 0.0217 | 0.388 | 0.0225 |
| | 220 | 0.422 | 0.0199 | 0.424 | 0.0188 | 0.423 | 0.0194 | | 220 | 0.432 | 0.0214 | 0.432 | 0.0208 | 0.429 | 0.0208 |
| | 277 | 0.486 | 0.0213 | 0.486 | 0.0211 | 0.484 | 0.0213 | | 277 | 0.486 | 0.0213 | 0.486 | 0.0211 | 0.484 | 0.0213 |
| WIClustCDtarg | 30 | 0.290 | 0.0297 | 0.295 | 0.0295 | 0.293 | 0.0299 | PAM | 30 | 0.254 | 0.0269 | 0.255 | 0.0271 | 0.249 | 0.0265 |
| | 55 | 0.329 | 0.0250 | 0.337 | 0.0240 | 0.333 | 0.0242 | | 55 | 0.341 | 0.0210 | 0.344 | 0.0215 | 0.341 | 0.0208 |
| | 110 | 0.377 | 0.0240 | 0.379 | 0.0229 | 0.378 | 0.0235 | | 110 | 0.397 | 0.0225 | 0.398 | 0.0231 | 0.396 | 0.0224 |
| | 165 | 0.410 | 0.0228 | 0.411 | 0.0225 | 0.411 | 0.0222 | | 165 | 0.424 | 0.0227 | 0.425 | 0.0232 | 0.421 | 0.0222 |
| | 220 | 0.428 | 0.0211 | 0.431 | 0.0202 | 0.428 | 0.0206 | | 220 | 0.451 | 0.0222 | 0.447 | 0.0224 | 0.447 | 0.0225 |
| | 277 | 0.486 | 0.0213 | 0.486 | 0.0211 | 0.484 | 0.0213 | | 277 | 0.486 | 0.0213 | 0.486 | 0.0211 | 0.484 | 0.0213 |

**Table S11** Average accuracy and its corresponding standard error of the mean (SEM) across the 40 iterations for all training set optimization methods, models and training set (TRS) sizes (expressed as percentage of the candidate set (CS)) for ricePopStr dataset and FP trait. If "targ" is added at the end of the name of a method, it corresponds to targeted optimization. Otherwise, untargeted optimization was performed.

**Rice_high_PS trait FP**

| | | Average accuracy | | | | | | | | Average accuracy | | | | | |
|---|---|---|---|---|---|---|---|---|---|---|---|---|---|---|---|
| | | GBLUP | | BayesB | | RKHS | | | | GBLUP | | BayesB | | RKHS | |
| Optimization method | TRS size (% of CS) | Mean | SEM | Mean | SEM | Mean | SEM | Optimization method | TRS size (% of CS) | Mean | SEM | Mean | SEM | Mean | SEM |
| RAND | 10 | 0.315 | 0.02279 | 0.403 | 0.01464 | 0.365 | 0.0218 | Rscore | 10 | 0.341 | 0.02147 | 0.453 | 0.01629 | 0.382 | 0.0197 |
| | 20 | 0.496 | 0.01945 | 0.508 | 0.01714 | 0.507 | 0.0197 | | 20 | 0.472 | 0.01860 | 0.513 | 0.01680 | 0.515 | 0.0178 |
| | 40 | 0.612 | 0.01167 | 0.607 | 0.01225 | 0.606 | 0.0117 | | 40 | 0.583 | 0.01562 | 0.581 | 0.01525 | 0.588 | 0.0155 |
| | 60 | 0.640 | 0.01177 | 0.638 | 0.01159 | 0.639 | 0.0122 | | 60 | 0.632 | 0.01234 | 0.629 | 0.01264 | 0.634 | 0.0124 |
| | 80 | 0.659 | 0.01016 | 0.659 | 0.01025 | 0.657 | 0.0103 | | 80 | 0.663 | 0.01053 | 0.660 | 0.01075 | 0.663 | 0.0109 |
| | 100 | 0.672 | 0.00987 | 0.671 | 0.01009 | 0.671 | 0.0102 | | 100 | 0.672 | 0.00987 | 0.671 | 0.01009 | 0.671 | 0.0102 |
| StratSamp | 10 | 0.353 | 0.02435 | 0.386 | 0.02305 | 0.380 | 0.0248 | Rscoretarg | 10 | 0.399 | 0.02336 | 0.488 | 0.01531 | 0.454 | 0.0199 |
| | 20 | 0.486 | 0.01963 | 0.514 | 0.01686 | 0.518 | 0.0178 | | 20 | 0.546 | 0.02171 | 0.576 | 0.01371 | 0.576 | 0.0153 |
| | 40 | 0.590 | 0.01315 | 0.588 | 0.01277 | 0.590 | 0.0140 | | 40 | 0.631 | 0.01167 | 0.627 | 0.01196 | 0.628 | 0.0124 |
| | 60 | 0.648 | 0.01351 | 0.645 | 0.01363 | 0.642 | 0.0139 | | 60 | 0.648 | 0.01145 | 0.648 | 0.01174 | 0.648 | 0.0117 |
| | 80 | 0.657 | 0.01034 | 0.656 | 0.01075 | 0.655 | 0.0110 | | 80 | 0.664 | 0.01042 | 0.663 | 0.01034 | 0.663 | 0.0109 |
| | 100 | 0.672 | 0.00987 | 0.671 | 0.01009 | 0.671 | 0.0102 | | 100 | 0.672 | 0.00987 | 0.671 | 0.01009 | 0.671 | 0.0102 |
| CD | 10 | 0.297 | 0.02604 | 0.419 | 0.01785 | 0.348 | 0.0271 | Avg_GRM | 10 | 0.142 | 0.03014 | 0.138 | 0.02972 | 0.145 | 0.0292 |
| | 20 | 0.541 | 0.01734 | 0.552 | 0.01340 | 0.541 | 0.0181 | | 20 | 0.215 | 0.02943 | 0.201 | 0.02878 | 0.218 | 0.0283 |
| | 40 | 0.613 | 0.01171 | 0.608 | 0.01212 | 0.618 | 0.0115 | | 40 | 0.365 | 0.01537 | 0.367 | 0.01576 | 0.371 | 0.0179 |
| | 60 | 0.639 | 0.01056 | 0.634 | 0.01085 | 0.641 | 0.0106 | | 60 | 0.479 | 0.02117 | 0.485 | 0.02002 | 0.480 | 0.0186 |
| | 80 | 0.667 | 0.00941 | 0.665 | 0.01005 | 0.669 | 0.0096 | | 80 | 0.602 | 0.01129 | 0.602 | 0.01130 | 0.599 | 0.0118 |
| | 100 | 0.672 | 0.00987 | 0.671 | 0.01009 | 0.671 | 0.0102 | | 100 | 0.672 | 0.00987 | 0.671 | 0.01009 | 0.671 | 0.0102 |
| CDtarg | 10 | 0.376 | 0.02752 | 0.515 | 0.01744 | 0.443 | 0.0271 | Avg_GRMtarg | 10 | 0.251 | 0.03255 | 0.246 | 0.03125 | 0.256 | 0.0318 |
| | 20 | 0.584 | 0.01658 | 0.606 | 0.01457 | 0.603 | 0.0149 | | 20 | 0.327 | 0.02553 | 0.326 | 0.02615 | 0.336 | 0.0248 |
| | 40 | 0.651 | 0.01145 | 0.650 | 0.01182 | 0.647 | 0.0117 | | 40 | 0.469 | 0.01636 | 0.473 | 0.01608 | 0.476 | 0.0168 |
| | 60 | 0.660 | 0.01004 | 0.660 | 0.01035 | 0.657 | 0.0107 | | 60 | 0.569 | 0.01150 | 0.572 | 0.01110 | 0.584 | 0.0114 |
| | 80 | 0.668 | 0.00960 | 0.667 | 0.00984 | 0.666 | 0.0101 | | 80 | 0.649 | 0.00934 | 0.650 | 0.00971 | 0.650 | 0.0095 |
| | 100 | 0.672 | 0.00987 | 0.671 | 0.01009 | 0.671 | 0.0102 | | 100 | 0.672 | 0.00987 | 0.671 | 0.01009 | 0.671 | 0.0102 |
| OvClustCD | 10 | 0.231 | 0.02663 | 0.395 | 0.02004 | 0.273 | 0.0259 | Avg_GRM_MinMax | 10 | 0.393 | 0.02282 | 0.513 | 0.01653 | 0.484 | 0.0208 |
| | 20 | 0.528 | 0.01675 | 0.549 | 0.01386 | 0.546 | 0.0154 | | 20 | 0.540 | 0.01800 | 0.558 | 0.01593 | 0.560 | 0.0173 |
| | 40 | 0.600 | 0.01219 | 0.594 | 0.01282 | 0.605 | 0.0124 | | 40 | 0.618 | 0.01366 | 0.614 | 0.01413 | 0.617 | 0.0135 |
| | 60 | 0.639 | 0.01030 | 0.633 | 0.01071 | 0.645 | 0.0102 | | 60 | 0.652 | 0.00966 | 0.648 | 0.01018 | 0.650 | 0.0103 |
| | 80 | 0.663 | 0.00999 | 0.659 | 0.01074 | 0.663 | 0.0101 | | 80 | 0.664 | 0.01069 | 0.661 | 0.01084 | 0.664 | 0.0111 |
| | 100 | 0.672 | 0.00987 | 0.671 | 0.01009 | 0.671 | 0.0102 | | 100 | 0.672 | 0.00987 | 0.671 | 0.01009 | 0.671 | 0.0102 |
| OvClustCDtarg | 10 | 0.396 | 0.03157 | 0.517 | 0.01921 | 0.457 | 0.0290 | Avg_GRM_MinMaxtarg | 10 | 0.427 | 0.02704 | 0.527 | 0.01422 | 0.499 | 0.0235 |
| | 20 | 0.577 | 0.02166 | 0.609 | 0.01509 | 0.603 | 0.0166 | | 20 | 0.571 | 0.02025 | 0.589 | 0.01608 | 0.591 | 0.0160 |
| | 40 | 0.652 | 0.01129 | 0.649 | 0.01137 | 0.648 | 0.0119 | | 40 | 0.637 | 0.01282 | 0.634 | 0.01323 | 0.639 | 0.0133 |
| | 60 | 0.662 | 0.01020 | 0.663 | 0.01060 | 0.659 | 0.0106 | | 60 | 0.653 | 0.01021 | 0.653 | 0.01039 | 0.653 | 0.0110 |
| | 80 | 0.666 | 0.00976 | 0.666 | 0.01009 | 0.665 | 0.0102 | | 80 | 0.667 | 0.00959 | 0.665 | 0.00986 | 0.667 | 0.0100 |
| | 100 | 0.672 | 0.00987 | 0.671 | 0.01009 | 0.671 | 0.0102 | | 100 | 0.672 | 0.00987 | 0.671 | 0.01009 | 0.671 | 0.0102 |
| WIClustCD | 10 | 0.402 | 0.02074 | 0.509 | 0.01716 | 0.485 | 0.0177 | Avg_GRM_self | 10 | 0.408 | 0.02139 | 0.508 | 0.01796 | 0.477 | 0.0185 |
| | 20 | 0.575 | 0.01573 | 0.569 | 0.01271 | 0.582 | 0.0147 | | 20 | 0.562 | 0.02014 | 0.575 | 0.01579 | 0.588 | 0.0147 |
| | 40 | 0.620 | 0.01089 | 0.612 | 0.01149 | 0.624 | 0.0115 | | 40 | 0.620 | 0.01089 | 0.616 | 0.01128 | 0.623 | 0.0111 |
| | 60 | 0.637 | 0.01005 | 0.631 | 0.01078 | 0.643 | 0.0104 | | 60 | 0.655 | 0.01108 | 0.653 | 0.01146 | 0.653 | 0.0115 |
| | 80 | 0.665 | 0.01020 | 0.660 | 0.01083 | 0.664 | 0.0104 | | 80 | 0.664 | 0.00980 | 0.663 | 0.00984 | 0.662 | 0.0101 |
| | 100 | 0.672 | 0.00987 | 0.671 | 0.01009 | 0.671 | 0.0102 | | 100 | 0.672 | 0.00987 | 0.671 | 0.01009 | 0.671 | 0.0102 |
| WIClustCDtarg | 10 | 0.422 | 0.02844 | 0.529 | 0.01816 | 0.477 | 0.0240 | PAM | 10 | 0.376 | 0.02085 | 0.528 | 0.01629 | 0.418 | 0.0211 |
| | 20 | 0.596 | 0.01773 | 0.606 | 0.01493 | 0.601 | 0.0159 | | 20 | 0.469 | 0.02622 | 0.551 | 0.01451 | 0.506 | 0.0250 |
| | 40 | 0.650 | 0.01168 | 0.650 | 0.01198 | 0.644 | 0.0127 | | 40 | 0.585 | 0.01200 | 0.583 | 0.01138 | 0.606 | 0.0127 |
| | 60 | 0.662 | 0.01045 | 0.661 | 0.01078 | 0.660 | 0.0110 | | 60 | 0.636 | 0.01072 | 0.635 | 0.01091 | 0.641 | 0.0111 |
| | 80 | 0.667 | 0.01008 | 0.666 | 0.01027 | 0.665 | 0.0104 | | 80 | 0.666 | 0.00971 | 0.663 | 0.01015 | 0.668 | 0.0100 |
| | 100 | 0.672 | 0.00987 | 0.671 | 0.01009 | 0.671 | 0.0102 | | 100 | 0.672 | 0.00987 | 0.671 | 0.01009 | 0.671 | 0.0102 |

**Table S12** Average accuracy and its corresponding standard error of the mean (SEM) across the 40 iterations for all training set optimization methods, models and training set (TRS) sizes (expressed as percentage of the candidate set (CS)) for ricePopStr dataset and FT trait. If "targ" is added at the end of the name of a method, it corresponds to targeted optimization. Otherwise, untargeted optimization was performed.

**Rice_high_PS trait FT**

| | | Average accuracy | | | | | | | | Average accuracy | | | | | |
|---|---|---|---|---|---|---|---|---|---|---|---|---|---|---|---|
| | | GBLUP | | BayesB | | RKHS | | | | GBLUP | | BayesB | | RKHS | |
| Optimization method | TRS size (% of CS) | Mean | SEM | Mean | SEM | Mean | SEM | Optimization method | TRS size (% of CS) | Mean | SEM | Mean | SEM | Mean | SEM |
| RAND | 10 | 0.4149 | 0.0219 | 0.4612 | 0.0174 | 0.455 | 0.0194 | Rscore | 10 | 0.3762 | 0.0197 | 0.4420 | 0.0247 | 0.405 | 0.0250 |
| | 20 | 0.5247 | 0.0164 | 0.5279 | 0.0147 | 0.539 | 0.0162 | | 20 | 0.4724 | 0.0174 | 0.5059 | 0.0164 | 0.498 | 0.0184 |
| | 40 | 0.6004 | 0.0146 | 0.5973 | 0.0143 | 0.608 | 0.0152 | | 40 | 0.5794 | 0.0153 | 0.5771 | 0.0163 | 0.597 | 0.0153 |
| | 60 | 0.6335 | 0.0144 | 0.6297 | 0.0148 | 0.642 | 0.0145 | | 60 | 0.6261 | 0.0154 | 0.6221 | 0.0153 | 0.638 | 0.0154 |
| | 80 | 0.6567 | 0.0143 | 0.6546 | 0.0145 | 0.662 | 0.0142 | | 80 | 0.6649 | 0.0132 | 0.6585 | 0.0135 | 0.678 | 0.0132 |
| | 100 | 0.6763 | 0.0134 | 0.6741 | 0.0137 | 0.682 | 0.0133 | | 100 | 0.6763 | 0.0134 | 0.6741 | 0.0137 | 0.682 | 0.0133 |
| StratSamp | 10 | 0.4118 | 0.0207 | 0.4441 | 0.0216 | 0.442 | 0.0216 | Rscoretarg | 10 | 0.4268 | 0.0285 | 0.4942 | 0.0230 | 0.477 | 0.0258 |
| | 20 | 0.5072 | 0.0177 | 0.5235 | 0.0176 | 0.540 | 0.0184 | | 20 | 0.5552 | 0.0229 | 0.5709 | 0.0196 | 0.583 | 0.0196 |
| | 40 | 0.5828 | 0.0171 | 0.5835 | 0.0174 | 0.595 | 0.0168 | | 40 | 0.6292 | 0.0165 | 0.6283 | 0.0166 | 0.637 | 0.0162 |
| | 60 | 0.6332 | 0.0151 | 0.6280 | 0.0153 | 0.640 | 0.0155 | | 60 | 0.6585 | 0.0144 | 0.6537 | 0.0149 | 0.671 | 0.0142 |
| | 80 | 0.6680 | 0.0137 | 0.6658 | 0.0139 | 0.673 | 0.0143 | | 80 | 0.6714 | 0.0136 | 0.6685 | 0.0138 | 0.678 | 0.0136 |
| | 100 | 0.6763 | 0.0134 | 0.6741 | 0.0137 | 0.682 | 0.0133 | | 100 | 0.6763 | 0.0134 | 0.6741 | 0.0137 | 0.682 | 0.0133 |
| CD | 10 | 0.3847 | 0.0181 | 0.4606 | 0.0192 | 0.411 | 0.0197 | Avg_GRM | 10 | 0.0877 | 0.0369 | 0.0588 | 0.0351 | 0.101 | 0.0376 |
| | 20 | 0.4969 | 0.0179 | 0.5320 | 0.0168 | 0.538 | 0.0185 | | 20 | 0.1719 | 0.0381 | 0.1414 | 0.0383 | 0.194 | 0.0369 |
| | 40 | 0.5827 | 0.0164 | 0.5766 | 0.0160 | 0.607 | 0.0157 | | 40 | 0.3520 | 0.0336 | 0.3577 | 0.0336 | 0.384 | 0.0315 |
| | 60 | 0.6081 | 0.0166 | 0.6014 | 0.0168 | 0.626 | 0.0166 | | 60 | 0.4659 | 0.0255 | 0.4782 | 0.0226 | 0.509 | 0.0202 |
| | 80 | 0.6555 | 0.0147 | 0.6501 | 0.0153 | 0.672 | 0.0143 | | 80 | 0.6076 | 0.0161 | 0.6061 | 0.0162 | 0.618 | 0.0153 |
| | 100 | 0.6763 | 0.0134 | 0.6741 | 0.0137 | 0.682 | 0.0133 | | 100 | 0.6763 | 0.0134 | 0.6741 | 0.0137 | 0.682 | 0.0133 |
| CDtarg | 10 | 0.4469 | 0.0232 | 0.5520 | 0.0174 | 0.486 | 0.0252 | Avg_GRMtarg | 10 | 0.0926 | 0.0404 | 0.1274 | 0.0379 | 0.120 | 0.0408 |
| | 20 | 0.5661 | 0.0182 | 0.6100 | 0.0136 | 0.605 | 0.0150 | | 20 | 0.2753 | 0.0414 | 0.2540 | 0.0408 | 0.296 | 0.0392 |
| | 40 | 0.6567 | 0.0142 | 0.6534 | 0.0140 | 0.662 | 0.0139 | | 40 | 0.4465 | 0.0266 | 0.4597 | 0.0250 | 0.467 | 0.0268 |
| | 60 | 0.6677 | 0.0133 | 0.6637 | 0.0133 | 0.672 | 0.0131 | | 60 | 0.5565 | 0.0224 | 0.5597 | 0.0215 | 0.576 | 0.0204 |
| | 80 | 0.6702 | 0.0134 | 0.6684 | 0.0135 | 0.677 | 0.0132 | | 80 | 0.6351 | 0.0172 | 0.6318 | 0.0173 | 0.640 | 0.0172 |
| | 100 | 0.6763 | 0.0134 | 0.6741 | 0.0137 | 0.682 | 0.0133 | | 100 | 0.6763 | 0.0134 | 0.6741 | 0.0137 | 0.682 | 0.0133 |
| OvClustCD | 10 | 0.3994 | 0.0183 | 0.4519 | 0.0176 | 0.416 | 0.0196 | Avg_GRM_MinMax | 10 | 0.4625 | 0.0220 | 0.4953 | 0.0187 | 0.490 | 0.0226 |
| | 20 | 0.4572 | 0.0173 | 0.4833 | 0.0161 | 0.476 | 0.0179 | | 20 | 0.5191 | 0.0173 | 0.5408 | 0.0154 | 0.546 | 0.0168 |
| | 40 | 0.5419 | 0.0162 | 0.5484 | 0.0166 | 0.576 | 0.0165 | | 40 | 0.6129 | 0.0146 | 0.6134 | 0.0159 | 0.628 | 0.0142 |
| | 60 | 0.6316 | 0.0150 | 0.6225 | 0.0159 | 0.650 | 0.0149 | | 60 | 0.6508 | 0.0149 | 0.6473 | 0.0156 | 0.658 | 0.0147 |
| | 80 | 0.6626 | 0.0148 | 0.6571 | 0.0150 | 0.679 | 0.0140 | | 80 | 0.6663 | 0.0136 | 0.6634 | 0.0138 | 0.673 | 0.0137 |
| | 100 | 0.6763 | 0.0134 | 0.6741 | 0.0137 | 0.682 | 0.0133 | | 100 | 0.6763 | 0.0134 | 0.6741 | 0.0137 | 0.682 | 0.0133 |
| OvClustCDtarg | 10 | 0.4693 | 0.0226 | 0.5485 | 0.0168 | 0.514 | 0.0239 | Avg_GRM_MinMaxtarg | 10 | 0.4865 | 0.0210 | 0.5366 | 0.0180 | 0.529 | 0.0203 |
| | 20 | 0.5786 | 0.0184 | 0.6162 | 0.0144 | 0.612 | 0.0161 | | 20 | 0.5584 | 0.0191 | 0.5902 | 0.0143 | 0.589 | 0.0168 |
| | 40 | 0.6553 | 0.0146 | 0.6590 | 0.0140 | 0.659 | 0.0140 | | 40 | 0.6416 | 0.0142 | 0.6402 | 0.0144 | 0.649 | 0.0138 |
| | 60 | 0.6700 | 0.0130 | 0.6674 | 0.0127 | 0.675 | 0.0132 | | 60 | 0.6626 | 0.0139 | 0.6604 | 0.0142 | 0.669 | 0.0144 |
| | 80 | 0.6717 | 0.0131 | 0.6698 | 0.0134 | 0.678 | 0.0131 | | 80 | 0.6709 | 0.0137 | 0.6687 | 0.0138 | 0.677 | 0.0137 |
| | 100 | 0.6763 | 0.0134 | 0.6741 | 0.0137 | 0.682 | 0.0133 | | 100 | 0.6763 | 0.0134 | 0.6741 | 0.0137 | 0.682 | 0.0133 |
| WIClustCD | 10 | 0.4468 | 0.0149 | 0.4928 | 0.0160 | 0.498 | 0.0167 | Avg_GRM_self | 10 | 0.4645 | 0.0200 | 0.4971 | 0.0194 | 0.495 | 0.0196 |
| | 20 | 0.5176 | 0.0183 | 0.5413 | 0.0164 | 0.556 | 0.0186 | | 20 | 0.5511 | 0.0200 | 0.5623 | 0.0176 | 0.576 | 0.0189 |
| | 40 | 0.5647 | 0.0150 | 0.5677 | 0.0158 | 0.583 | 0.0155 | | 40 | 0.6205 | 0.0163 | 0.6264 | 0.0152 | 0.632 | 0.0154 |
| | 60 | 0.6304 | 0.0146 | 0.6286 | 0.0149 | 0.650 | 0.0146 | | 60 | 0.6540 | 0.0134 | 0.6510 | 0.0137 | 0.660 | 0.0137 |
| | 80 | 0.6704 | 0.0144 | 0.6650 | 0.0147 | 0.688 | 0.0137 | | 80 | 0.6684 | 0.0131 | 0.6682 | 0.0130 | 0.674 | 0.0130 |
| | 100 | 0.6763 | 0.0134 | 0.6741 | 0.0137 | 0.682 | 0.0133 | | 100 | 0.6763 | 0.0134 | 0.6741 | 0.0137 | 0.682 | 0.0133 |
| WIClustCDtarg | 10 | 0.4716 | 0.0202 | 0.5417 | 0.0152 | 0.504 | 0.0196 | PAM | 10 | 0.4504 | 0.0183 | 0.4811 | 0.0165 | 0.494 | 0.0186 |
| | 20 | 0.5651 | 0.0187 | 0.6052 | 0.0151 | 0.602 | 0.0168 | | 20 | 0.5264 | 0.0170 | 0.5431 | 0.0156 | 0.559 | 0.0164 |
| | 40 | 0.6438 | 0.0138 | 0.6452 | 0.0138 | 0.652 | 0.0138 | | 40 | 0.5695 | 0.0159 | 0.5696 | 0.0157 | 0.594 | 0.0159 |
| | 60 | 0.6625 | 0.0130 | 0.6609 | 0.0128 | 0.669 | 0.0134 | | 60 | 0.6283 | 0.0145 | 0.6192 | 0.0150 | 0.642 | 0.0144 |
| | 80 | 0.6710 | 0.0135 | 0.6696 | 0.0135 | 0.676 | 0.0136 | | 80 | 0.6613 | 0.0139 | 0.6566 | 0.0141 | 0.669 | 0.0139 |
| | 100 | 0.6763 | 0.0134 | 0.6741 | 0.0137 | 0.682 | 0.0133 | | 100 | 0.6763 | 0.0134 | 0.6741 | 0.0137 | 0.682 | 0.0133 |

**Table S13** Average accuracy and its corresponding standard error of the mean (SEM) across the 40 iterations for all training set optimization methods, models and training set (TRS) sizes (expressed as percentage of the candidate set (CS)) for ricePopStr dataset and HT trait. If "targ" is added at the end of the name of a method, it corresponds to targeted optimization. Otherwise, untargeted optimization was performed.

**Rice_high_PS trait HT**

| Optimization method | TRS size (% of CS) | Average accuracy | | | | | | Optimization method | TRS size (% of CS) | Average accuracy | | | | | |
|---|---|---|---|---|---|---|---|---|---|---|---|---|---|---|---|
| | | GBLUP | | BayesB | | RKHS | | | | GBLUP | | BayesB | | RKHS | |
| | | Mean | SEM | Mean | SEM | Mean | SEM | | | Mean | SEM | Mean | SEM | Mean | SEM |
| RAND | 10 | 0.617 | 0.01455 | 0.613 | 0.01636 | 0.609 | 0.01499 | Rscore | 10 | 0.648 | 0.01422 | 0.639 | 0.01450 | 0.644 | 0.01469 |
| | 20 | 0.683 | 0.01128 | 0.672 | 0.01181 | 0.681 | 0.01180 | | 20 | 0.711 | 0.01036 | 0.700 | 0.01070 | 0.710 | 0.01069 |
| | 40 | 0.745 | 0.01053 | 0.739 | 0.01098 | 0.743 | 0.01091 | | 40 | 0.768 | 0.00865 | 0.760 | 0.00881 | 0.768 | 0.00883 |
| | 60 | 0.781 | 0.00834 | 0.778 | 0.00814 | 0.780 | 0.00850 | | 60 | 0.790 | 0.00736 | 0.787 | 0.00750 | 0.790 | 0.00761 |
| | 80 | 0.798 | 0.00774 | 0.797 | 0.00756 | 0.797 | 0.00786 | | 80 | 0.807 | 0.00779 | 0.805 | 0.00815 | 0.808 | 0.00785 |
| | 100 | 0.812 | 0.00717 | 0.810 | 0.00728 | 0.812 | 0.00731 | | 100 | 0.812 | 0.00717 | 0.810 | 0.00728 | 0.812 | 0.00731 |
| StratSamp | 10 | 0.605 | 0.01488 | 0.601 | 0.01403 | 0.600 | 0.01566 | Rscoretarg | 10 | 0.681 | 0.01310 | 0.666 | 0.01255 | 0.674 | 0.01336 |
| | 20 | 0.681 | 0.01120 | 0.675 | 0.01163 | 0.678 | 0.01147 | | 20 | 0.744 | 0.01037 | 0.734 | 0.01048 | 0.742 | 0.01057 |
| | 40 | 0.737 | 0.00951 | 0.733 | 0.00980 | 0.736 | 0.00960 | | 40 | 0.786 | 0.00758 | 0.779 | 0.00758 | 0.785 | 0.00775 |
| | 60 | 0.780 | 0.00761 | 0.776 | 0.00780 | 0.780 | 0.00770 | | 60 | 0.807 | 0.00693 | 0.801 | 0.00700 | 0.806 | 0.00713 |
| | 80 | 0.798 | 0.00759 | 0.797 | 0.00789 | 0.798 | 0.00770 | | 80 | 0.811 | 0.00726 | 0.807 | 0.00743 | 0.811 | 0.00741 |
| | 100 | 0.812 | 0.00717 | 0.810 | 0.00728 | 0.812 | 0.00731 | | 100 | 0.812 | 0.00717 | 0.810 | 0.00728 | 0.812 | 0.00731 |
| CD | 10 | 0.662 | 0.01260 | 0.653 | 0.01272 | 0.659 | 0.01278 | Avg_GRM | 10 | 0.214 | 0.04435 | 0.176 | 0.05272 | 0.209 | 0.04305 |
| | 20 | 0.732 | 0.00937 | 0.717 | 0.00992 | 0.731 | 0.00950 | | 20 | 0.258 | 0.04764 | 0.256 | 0.05042 | 0.229 | 0.04417 |
| | 40 | 0.767 | 0.00906 | 0.757 | 0.00952 | 0.768 | 0.00940 | | 40 | 0.478 | 0.03741 | 0.454 | 0.03605 | 0.467 | 0.03705 |
| | 60 | 0.791 | 0.00819 | 0.785 | 0.00822 | 0.791 | 0.00840 | | 60 | 0.578 | 0.03937 | 0.576 | 0.03910 | 0.582 | 0.03823 |
| | 80 | 0.808 | 0.00748 | 0.806 | 0.00727 | 0.808 | 0.00787 | | 80 | 0.737 | 0.01169 | 0.737 | 0.01153 | 0.738 | 0.01166 |
| | 100 | 0.812 | 0.00717 | 0.810 | 0.00728 | 0.812 | 0.00731 | | 100 | 0.812 | 0.00717 | 0.810 | 0.00728 | 0.812 | 0.00731 |
| CDtarg | 10 | 0.717 | 0.01061 | 0.715 | 0.01030 | 0.713 | 0.01078 | Avg_GRMtarg | 10 | 0.233 | 0.05333 | 0.214 | 0.05119 | 0.244 | 0.05044 |
| | 20 | 0.768 | 0.00932 | 0.764 | 0.00900 | 0.765 | 0.00950 | | 20 | 0.454 | 0.03614 | 0.424 | 0.03781 | 0.439 | 0.03423 |
| | 40 | 0.802 | 0.00774 | 0.797 | 0.00755 | 0.801 | 0.00786 | | 40 | 0.551 | 0.03864 | 0.554 | 0.03744 | 0.553 | 0.03678 |
| | 60 | 0.809 | 0.00734 | 0.806 | 0.00714 | 0.808 | 0.00751 | | 60 | 0.678 | 0.02669 | 0.680 | 0.02550 | 0.686 | 0.02483 |
| | 80 | 0.812 | 0.00724 | 0.812 | 0.00717 | 0.812 | 0.00737 | | 80 | 0.798 | 0.00914 | 0.796 | 0.00904 | 0.799 | 0.00918 |
| | 100 | 0.812 | 0.00717 | 0.810 | 0.00728 | 0.812 | 0.00731 | | 100 | 0.812 | 0.00717 | 0.810 | 0.00728 | 0.812 | 0.00731 |
| OvClustCD | 10 | 0.646 | 0.01329 | 0.642 | 0.01307 | 0.646 | 0.01367 | Avg_GRM_MinMax | 10 | 0.659 | 0.01178 | 0.647 | 0.01265 | 0.653 | 0.01189 |
| | 20 | 0.695 | 0.01134 | 0.683 | 0.01196 | 0.694 | 0.01151 | | 20 | 0.714 | 0.01033 | 0.704 | 0.01120 | 0.710 | 0.01031 |
| | 40 | 0.740 | 0.01037 | 0.732 | 0.01036 | 0.740 | 0.01054 | | 40 | 0.773 | 0.00785 | 0.766 | 0.00822 | 0.772 | 0.00804 |
| | 60 | 0.781 | 0.00809 | 0.776 | 0.00784 | 0.781 | 0.00828 | | 60 | 0.795 | 0.00765 | 0.790 | 0.00781 | 0.794 | 0.00776 |
| | 80 | 0.804 | 0.00734 | 0.799 | 0.00736 | 0.803 | 0.00756 | | 80 | 0.807 | 0.00733 | 0.805 | 0.00758 | 0.807 | 0.00750 |
| | 100 | 0.812 | 0.00717 | 0.810 | 0.00728 | 0.812 | 0.00731 | | 100 | 0.812 | 0.00717 | 0.810 | 0.00728 | 0.812 | 0.00731 |
| OvClustCDtarg | 10 | 0.710 | 0.01211 | 0.711 | 0.01202 | 0.708 | 0.01227 | Avg_GRM_MinMaxtarg | 10 | 0.698 | 0.00977 | 0.694 | 0.00923 | 0.693 | 0.00980 |
| | 20 | 0.762 | 0.01013 | 0.757 | 0.00971 | 0.759 | 0.01022 | | 20 | 0.748 | 0.00889 | 0.738 | 0.00909 | 0.745 | 0.00908 |
| | 40 | 0.802 | 0.00738 | 0.797 | 0.00734 | 0.800 | 0.00755 | | 40 | 0.792 | 0.00798 | 0.786 | 0.00794 | 0.791 | 0.00812 |
| | 60 | 0.810 | 0.00711 | 0.809 | 0.00709 | 0.809 | 0.00729 | | 60 | 0.804 | 0.00760 | 0.801 | 0.00761 | 0.804 | 0.00770 |
| | 80 | 0.814 | 0.00697 | 0.813 | 0.00704 | 0.813 | 0.00714 | | 80 | 0.811 | 0.00719 | 0.811 | 0.00711 | 0.811 | 0.00732 |
| | 100 | 0.812 | 0.00717 | 0.810 | 0.00728 | 0.812 | 0.00731 | | 100 | 0.812 | 0.00717 | 0.810 | 0.00728 | 0.812 | 0.00731 |
| WIClustCD | 10 | 0.622 | 0.01379 | 0.616 | 0.01342 | 0.614 | 0.01471 | Avg_GRM_self | 10 | 0.661 | 0.01268 | 0.666 | 0.01172 | 0.654 | 0.01220 |
| | 20 | 0.691 | 0.01106 | 0.681 | 0.01165 | 0.689 | 0.01104 | | 20 | 0.735 | 0.01093 | 0.720 | 0.01037 | 0.731 | 0.01106 |
| | 40 | 0.748 | 0.00890 | 0.740 | 0.00894 | 0.747 | 0.00948 | | 40 | 0.776 | 0.00806 | 0.770 | 0.00764 | 0.774 | 0.00823 |
| | 60 | 0.782 | 0.00835 | 0.779 | 0.00783 | 0.783 | 0.00843 | | 60 | 0.798 | 0.00765 | 0.794 | 0.00789 | 0.797 | 0.00779 |
| | 80 | 0.803 | 0.00769 | 0.800 | 0.00753 | 0.803 | 0.00797 | | 80 | 0.808 | 0.00717 | 0.806 | 0.00731 | 0.808 | 0.00732 |
| | 100 | 0.812 | 0.00717 | 0.810 | 0.00728 | 0.812 | 0.00731 | | 100 | 0.812 | 0.00717 | 0.810 | 0.00728 | 0.812 | 0.00731 |
| WIClustCDtarg | 10 | 0.660 | 0.01149 | 0.666 | 0.01098 | 0.656 | 0.01225 | PAM | 10 | 0.663 | 0.01153 | 0.642 | 0.01158 | 0.662 | 0.01173 |
| | 20 | 0.736 | 0.01047 | 0.730 | 0.01059 | 0.735 | 0.01061 | | 20 | 0.725 | 0.00865 | 0.704 | 0.00957 | 0.727 | 0.00869 |
| | 40 | 0.797 | 0.00776 | 0.792 | 0.00783 | 0.796 | 0.00794 | | 40 | 0.776 | 0.00745 | 0.763 | 0.00791 | 0.775 | 0.00766 |
| | 60 | 0.809 | 0.00718 | 0.807 | 0.00719 | 0.808 | 0.00737 | | 60 | 0.794 | 0.00693 | 0.789 | 0.00667 | 0.794 | 0.00705 |
| | 80 | 0.811 | 0.00697 | 0.811 | 0.00657 | 0.811 | 0.00713 | | 80 | 0.807 | 0.00676 | 0.807 | 0.00721 | 0.808 | 0.00694 |
| | 100 | 0.812 | 0.00717 | 0.810 | 0.00728 | 0.812 | 0.00731 | | 100 | 0.812 | 0.00717 | 0.810 | 0.00728 | 0.812 | 0.00731 |

**Table S14** Average accuracy and its corresponding standard error of the mean (SEM) across the 40 iterations for all training set optimization methods, models and training set (TRS) sizes (expressed as percentage of the candidate set (CS)) for ricePopStr dataset and PC trait. If "targ" is added at the end of the name of a method, it corresponds to targeted optimization. Otherwise, untargeted optimization was performed.

**Rice_high_PS trait PC**

| Optimization method | TRS size (% of CS) | Average accuracy | | | | | | Optimization method | TRS size (% of CS) | Average accuracy | | | | | |
|---|---|---|---|---|---|---|---|---|---|---|---|---|---|---|---|
| | | GBLUP | | BayesB | | RKHS | | | | GBLUP | | BayesB | | RKHS | |
| | | Mean | SEM | Mean | SEM | Mean | SEM | | | Mean | SEM | Mean | SEM | Mean | SEM |
| RAND | 10 | 0.3140 | 0.0165 | 0.3351 | 0.0205 | 0.327 | 0.0163 | Rscore | 10 | 0.3303 | 0.0167 | 0.3605 | 0.0206 | 0.337 | 0.0174 |
| | 20 | 0.3765 | 0.0177 | 0.4177 | 0.0184 | 0.416 | 0.0186 | | 20 | 0.3557 | 0.0140 | 0.3878 | 0.0140 | 0.381 | 0.0149 |
| | 40 | 0.4615 | 0.0154 | 0.4709 | 0.0155 | 0.497 | 0.0156 | | 40 | 0.3981 | 0.0164 | 0.4233 | 0.0164 | 0.441 | 0.0168 |
| | 60 | 0.4890 | 0.0153 | 0.4896 | 0.0151 | 0.513 | 0.0155 | | 60 | 0.4513 | 0.0157 | 0.4619 | 0.0162 | 0.493 | 0.0160 |
| | 80 | 0.5042 | 0.0160 | 0.5064 | 0.0158 | 0.526 | 0.0158 | | 80 | 0.4793 | 0.0166 | 0.4809 | 0.0166 | 0.510 | 0.0159 |
| | 100 | 0.5022 | 0.0162 | 0.5011 | 0.0161 | 0.525 | 0.0160 | | 100 | 0.5022 | 0.0162 | 0.5011 | 0.0161 | 0.525 | 0.0160 |
| StratSamp | 10 | 0.3049 | 0.0224 | 0.3340 | 0.0218 | 0.332 | 0.0215 | Rscoretarg | 10 | 0.3451 | 0.0144 | 0.3962 | 0.0183 | 0.363 | 0.0166 |
| | 20 | 0.3638 | 0.0172 | 0.3980 | 0.0161 | 0.411 | 0.0184 | | 20 | 0.3958 | 0.0237 | 0.4385 | 0.0179 | 0.448 | 0.0170 |
| | 40 | 0.4642 | 0.0150 | 0.4727 | 0.0149 | 0.494 | 0.0149 | | 40 | 0.4691 | 0.0161 | 0.4741 | 0.0158 | 0.495 | 0.0156 |
| | 60 | 0.4781 | 0.0165 | 0.4806 | 0.0162 | 0.503 | 0.0161 | | 60 | 0.4930 | 0.0157 | 0.4965 | 0.0158 | 0.514 | 0.0162 |
| | 80 | 0.4957 | 0.0158 | 0.4977 | 0.0159 | 0.517 | 0.0157 | | 80 | 0.5019 | 0.0159 | 0.5027 | 0.0157 | 0.526 | 0.0155 |
| | 100 | 0.5022 | 0.0162 | 0.5011 | 0.0161 | 0.525 | 0.0160 | | 100 | 0.5022 | 0.0162 | 0.5011 | 0.0161 | 0.525 | 0.0160 |
| CD | 10 | 0.3643 | 0.0150 | 0.4319 | 0.0151 | 0.410 | 0.0165 | Avg_GRM | 10 | 0.0638 | 0.0343 | 0.0857 | 0.0349 | 0.096 | 0.0327 |
| | 20 | 0.3894 | 0.0134 | 0.4449 | 0.0153 | 0.467 | 0.0170 | | 20 | 0.1202 | 0.0307 | 0.1179 | 0.0295 | 0.154 | 0.0312 |
| | 40 | 0.4044 | 0.0144 | 0.4352 | 0.0165 | 0.435 | 0.0149 | | 40 | 0.2054 | 0.0315 | 0.2008 | 0.0320 | 0.292 | 0.0237 |
| | 60 | 0.3945 | 0.0136 | 0.4289 | 0.0158 | 0.441 | 0.0158 | | 60 | 0.2542 | 0.0366 | 0.2589 | 0.0360 | 0.327 | 0.0273 |
| | 80 | 0.4684 | 0.0160 | 0.4724 | 0.0162 | 0.503 | 0.0158 | | 80 | 0.4459 | 0.0183 | 0.4470 | 0.0184 | 0.475 | 0.0170 |
| | 100 | 0.5022 | 0.0162 | 0.5011 | 0.0161 | 0.525 | 0.0160 | | 100 | 0.5022 | 0.0162 | 0.5011 | 0.0161 | 0.525 | 0.0160 |
| CDtarg | 10 | 0.3691 | 0.0216 | 0.4235 | 0.0203 | 0.424 | 0.0244 | Avg_GRMtarg | 10 | 0.0914 | 0.0302 | 0.1091 | 0.0338 | 0.137 | 0.0298 |
| | 20 | 0.4636 | 0.0195 | 0.4704 | 0.0187 | 0.486 | 0.0182 | | 20 | 0.1557 | 0.0299 | 0.1569 | 0.0295 | 0.188 | 0.0263 |
| | 40 | 0.4892 | 0.0172 | 0.4909 | 0.0173 | 0.507 | 0.0164 | | 40 | 0.2329 | 0.0332 | 0.2419 | 0.0312 | 0.330 | 0.0228 |
| | 60 | 0.4989 | 0.0166 | 0.5012 | 0.0165 | 0.518 | 0.0164 | | 60 | 0.2907 | 0.0305 | 0.2905 | 0.0308 | 0.366 | 0.0218 |
| | 80 | 0.5056 | 0.0160 | 0.5054 | 0.0161 | 0.526 | 0.0158 | | 80 | 0.4600 | 0.0184 | 0.4652 | 0.0181 | 0.483 | 0.0179 |
| | 100 | 0.5022 | 0.0162 | 0.5011 | 0.0161 | 0.525 | 0.0160 | | 100 | 0.5022 | 0.0162 | 0.5011 | 0.0161 | 0.525 | 0.0160 |
| OvClustCD | 10 | 0.3775 | 0.0145 | 0.4467 | 0.0136 | 0.450 | 0.0176 | Avg_GRM_MinMax | 10 | 0.3523 | 0.0145 | 0.4232 | 0.0148 | 0.381 | 0.0165 |
| | 20 | 0.4755 | 0.0163 | 0.4766 | 0.0157 | 0.511 | 0.0161 | | 20 | 0.4015 | 0.0155 | 0.4454 | 0.0145 | 0.438 | 0.0146 |
| | 40 | 0.3895 | 0.0129 | 0.4125 | 0.0136 | 0.426 | 0.0139 | | 40 | 0.4822 | 0.0148 | 0.4879 | 0.0149 | 0.505 | 0.0152 |
| | 60 | 0.4570 | 0.0148 | 0.4712 | 0.0159 | 0.505 | 0.0155 | | 60 | 0.4884 | 0.0159 | 0.4886 | 0.0158 | 0.510 | 0.0157 |
| | 80 | 0.4910 | 0.0158 | 0.4944 | 0.0158 | 0.517 | 0.0160 | | 80 | 0.4937 | 0.0159 | 0.4952 | 0.0158 | 0.520 | 0.0160 |
| | 100 | 0.5022 | 0.0162 | 0.5011 | 0.0161 | 0.525 | 0.0160 | | 100 | 0.5022 | 0.0162 | 0.5011 | 0.0161 | 0.525 | 0.0160 |
| OvClustCDtarg | 10 | 0.3441 | 0.0230 | 0.4218 | 0.0209 | 0.428 | 0.0228 | Avg_GRM_MinMaxtarg | 10 | 0.3401 | 0.0237 | 0.3914 | 0.0209 | 0.391 | 0.0269 |
| | 20 | 0.4687 | 0.0181 | 0.4846 | 0.0167 | 0.498 | 0.0158 | | 20 | 0.4090 | 0.0150 | 0.4437 | 0.0158 | 0.455 | 0.0158 |
| | 40 | 0.4941 | 0.0162 | 0.4938 | 0.0162 | 0.511 | 0.0158 | | 40 | 0.4761 | 0.0170 | 0.4840 | 0.0167 | 0.500 | 0.0165 |
| | 60 | 0.4963 | 0.0163 | 0.4985 | 0.0163 | 0.516 | 0.0163 | | 60 | 0.4911 | 0.0163 | 0.4929 | 0.0163 | 0.514 | 0.0161 |
| | 80 | 0.5031 | 0.0159 | 0.5053 | 0.0156 | 0.524 | 0.0159 | | 80 | 0.4982 | 0.0163 | 0.5001 | 0.0161 | 0.522 | 0.0160 |
| | 100 | 0.5022 | 0.0162 | 0.5011 | 0.0161 | 0.525 | 0.0160 | | 100 | 0.5022 | 0.0162 | 0.5011 | 0.0161 | 0.525 | 0.0160 |
| WIClustCD | 10 | 0.3690 | 0.0147 | 0.4213 | 0.0180 | 0.407 | 0.0189 | Avg_GRM_self | 10 | 0.3505 | 0.0141 | 0.4191 | 0.0155 | 0.392 | 0.0185 |
| | 20 | 0.4670 | 0.0155 | 0.4844 | 0.0140 | 0.503 | 0.0144 | | 20 | 0.4269 | 0.0178 | 0.4609 | 0.0159 | 0.484 | 0.0178 |
| | 40 | 0.4065 | 0.0143 | 0.4297 | 0.0154 | 0.451 | 0.0154 | | 40 | 0.4695 | 0.0143 | 0.4760 | 0.0150 | 0.496 | 0.0141 |
| | 60 | 0.4689 | 0.0157 | 0.4767 | 0.0162 | 0.505 | 0.0158 | | 60 | 0.4938 | 0.0163 | 0.4932 | 0.0163 | 0.515 | 0.0163 |
| | 80 | 0.4914 | 0.0160 | 0.4932 | 0.0162 | 0.515 | 0.0160 | | 80 | 0.4962 | 0.0159 | 0.4981 | 0.0160 | 0.520 | 0.0157 |
| | 100 | 0.5022 | 0.0162 | 0.5011 | 0.0161 | 0.525 | 0.0160 | | 100 | 0.5022 | 0.0162 | 0.5011 | 0.0161 | 0.525 | 0.0160 |
| WIClustCDtarg | 10 | 0.3921 | 0.0194 | 0.4229 | 0.0183 | 0.435 | 0.0186 | PAM | 10 | 0.3488 | 0.0168 | 0.3879 | 0.0188 | 0.361 | 0.0168 |
| | 20 | 0.4753 | 0.0162 | 0.4805 | 0.0160 | 0.492 | 0.0157 | | 20 | 0.3618 | 0.0160 | 0.4238 | 0.0182 | 0.402 | 0.0186 |
| | 40 | 0.4933 | 0.0165 | 0.4954 | 0.0165 | 0.510 | 0.0159 | | 40 | 0.3624 | 0.0138 | 0.3999 | 0.0149 | 0.404 | 0.0149 |
| | 60 | 0.4995 | 0.0168 | 0.5022 | 0.0167 | 0.520 | 0.0167 | | 60 | 0.3926 | 0.0140 | 0.4340 | 0.0157 | 0.467 | 0.0157 |
| | 80 | 0.5060 | 0.0157 | 0.5085 | 0.0157 | 0.525 | 0.0160 | | 80 | 0.4548 | 0.0147 | 0.4704 | 0.0158 | 0.503 | 0.0156 |
| | 100 | 0.5022 | 0.0162 | 0.5011 | 0.0161 | 0.525 | 0.0160 | | 100 | 0.5022 | 0.0162 | 0.5011 | 0.0161 | 0.525 | 0.0160 |

**Table S15** Average accuracy and its corresponding standard error of the mean (SEM) across the 40 iterations for all training set optimization methods, models and training set (TRS) sizes (expressed as percentage of the candidate set) for ricePopStr dataset and the simulated trait. If "targ" is added at the end of the name of a method, it corresponds to targeted optimization. Otherwise, untargeted optimization was performed.

**Rice_high_PS trait simulated1**

| Optimization method | TRS size (% of CS) | Average accuracy | | | | | | Optimization method | TRS size (% of CS) | Average accuracy | | | | | |
|---|---|---|---|---|---|---|---|---|---|---|---|---|---|---|---|
| | | GBLUP | | BayesB | | RKHS | | | | GBLUP | | BayesB | | RKHS | |
| | | Mean | SEM | Mean | SEM | Mean | SEM | | | Mean | SEM | Mean | SEM | Mean | SEM |
| RAND | 10 | 0.4232 | 0.0274 | 0.424 | 0.0250 | 0.417 | 0.0269 | Rscore | 10 | 0.4096 | 0.0308 | 0.434 | 0.0260 | 0.432 | 0.0280 |
| | 20 | 0.4705 | 0.0256 | 0.467 | 0.0254 | 0.465 | 0.0257 | | 20 | 0.4589 | 0.0273 | 0.480 | 0.0209 | 0.459 | 0.0265 |
| | 40 | 0.4987 | 0.0237 | 0.506 | 0.0210 | 0.506 | 0.0209 | | 40 | 0.4948 | 0.0247 | 0.506 | 0.0224 | 0.502 | 0.0231 |
| | 60 | 0.5160 | 0.0209 | 0.521 | 0.0199 | 0.521 | 0.0197 | | 60 | 0.5151 | 0.0214 | 0.523 | 0.0206 | 0.518 | 0.0206 |
| | 80 | 0.5242 | 0.0199 | 0.523 | 0.0196 | 0.526 | 0.0194 | | 80 | 0.5211 | 0.0212 | 0.524 | 0.0207 | 0.525 | 0.0204 |
| | 100 | 0.5335 | 0.0193 | 0.535 | 0.0191 | 0.535 | 0.0192 | | 100 | 0.5335 | 0.0193 | 0.535 | 0.0191 | 0.535 | 0.0192 |
| StratSamp | 10 | 0.4200 | 0.0305 | 0.419 | 0.0286 | 0.420 | 0.0301 | Rscoretarg | 10 | 0.4641 | 0.0232 | 0.473 | 0.0215 | 0.463 | 0.0226 |
| | 20 | 0.4600 | 0.0262 | 0.469 | 0.0227 | 0.463 | 0.0248 | | 20 | 0.4882 | 0.0230 | 0.498 | 0.0197 | 0.493 | 0.0209 |
| | 40 | 0.5029 | 0.0222 | 0.512 | 0.0208 | 0.511 | 0.0212 | | 40 | 0.5055 | 0.0239 | 0.512 | 0.0184 | 0.509 | 0.0235 |
| | 60 | 0.5150 | 0.0204 | 0.516 | 0.0203 | 0.515 | 0.0200 | | 60 | 0.5181 | 0.0214 | 0.522 | 0.0211 | 0.521 | 0.0208 |
| | 80 | 0.5265 | 0.0199 | 0.528 | 0.0193 | 0.529 | 0.0196 | | 80 | 0.5300 | 0.0191 | 0.529 | 0.0187 | 0.532 | 0.0190 |
| | 100 | 0.5335 | 0.0193 | 0.535 | 0.0191 | 0.535 | 0.0192 | | 100 | 0.5335 | 0.0193 | 0.535 | 0.0191 | 0.535 | 0.0192 |
| CD | 10 | 0.4725 | 0.0261 | 0.476 | 0.0257 | 0.469 | 0.0259 | Avg_GRM | 10 | 0.0228 | 0.0494 | 0.066 | 0.0543 | 0.019 | 0.0486 |
| | 20 | 0.4892 | 0.0234 | 0.501 | 0.0224 | 0.488 | 0.0239 | | 20 | 0.1232 | 0.0448 | 0.147 | 0.0395 | 0.125 | 0.0409 |
| | 40 | 0.4976 | 0.0233 | 0.499 | 0.0239 | 0.496 | 0.0230 | | 40 | 0.3225 | 0.0394 | 0.348 | 0.0383 | 0.315 | 0.0375 |
| | 60 | 0.5039 | 0.0216 | 0.502 | 0.0219 | 0.503 | 0.0211 | | 60 | 0.3854 | 0.0374 | 0.381 | 0.0388 | 0.372 | 0.0354 |
| | 80 | 0.5253 | 0.0205 | 0.524 | 0.0206 | 0.527 | 0.0204 | | 80 | 0.4934 | 0.0220 | 0.499 | 0.0198 | 0.500 | 0.0201 |
| | 100 | 0.5335 | 0.0193 | 0.535 | 0.0191 | 0.535 | 0.0192 | | 100 | 0.5335 | 0.0193 | 0.535 | 0.0191 | 0.535 | 0.0192 |
| CDtarg | 10 | 0.4665 | 0.0253 | 0.475 | 0.0215 | 0.469 | 0.0238 | Avg_GRMtarg | 10 | 0.1949 | 0.0316 | 0.204 | 0.0353 | 0.190 | 0.0280 |
| | 20 | 0.4991 | 0.0226 | 0.505 | 0.0203 | 0.505 | 0.0202 | | 20 | 0.2228 | 0.0393 | 0.224 | 0.0439 | 0.234 | 0.0342 |
| | 40 | 0.5195 | 0.0205 | 0.524 | 0.0192 | 0.523 | 0.0199 | | 40 | 0.3883 | 0.0307 | 0.393 | 0.0297 | 0.393 | 0.0292 |
| | 60 | 0.5309 | 0.0193 | 0.529 | 0.0191 | 0.532 | 0.0194 | | 60 | 0.4474 | 0.0286 | 0.451 | 0.0288 | 0.448 | 0.0281 |
| | 80 | 0.5306 | 0.0193 | 0.530 | 0.0192 | 0.531 | 0.0192 | | 80 | 0.5173 | 0.0208 | 0.518 | 0.0206 | 0.516 | 0.0211 |
| | 100 | 0.5335 | 0.0193 | 0.535 | 0.0191 | 0.535 | 0.0192 | | 100 | 0.5335 | 0.0193 | 0.535 | 0.0191 | 0.535 | 0.0192 |
| OvClustCD | 10 | 0.4662 | 0.0272 | 0.473 | 0.0242 | 0.466 | 0.0275 | Avg_GRM_MinMax | 10 | 0.4469 | 0.0293 | 0.447 | 0.0263 | 0.449 | 0.0288 |
| | 20 | 0.4763 | 0.0260 | 0.497 | 0.0219 | 0.475 | 0.0249 | | 20 | 0.4753 | 0.0229 | 0.488 | 0.0213 | 0.476 | 0.0225 |
| | 40 | 0.4924 | 0.0246 | 0.502 | 0.0215 | 0.494 | 0.0226 | | 40 | 0.5023 | 0.0208 | 0.509 | 0.0197 | 0.503 | 0.0211 |
| | 60 | 0.5064 | 0.0248 | 0.518 | 0.0208 | 0.511 | 0.0214 | | 60 | 0.5222 | 0.0203 | 0.525 | 0.0198 | 0.526 | 0.0195 |
| | 80 | 0.5254 | 0.0204 | 0.526 | 0.0205 | 0.523 | 0.0204 | | 80 | 0.5331 | 0.0200 | 0.533 | 0.0201 | 0.533 | 0.0200 |
| | 100 | 0.5335 | 0.0193 | 0.535 | 0.0191 | 0.535 | 0.0192 | | 100 | 0.5335 | 0.0193 | 0.535 | 0.0191 | 0.535 | 0.0192 |
| OvClustCDtarg | 10 | 0.4700 | 0.0232 | 0.476 | 0.0215 | 0.468 | 0.0234 | Avg_GRM_MinMaxtarg | 10 | 0.4358 | 0.0253 | 0.452 | 0.0222 | 0.444 | 0.0244 |
| | 20 | 0.5024 | 0.0206 | 0.502 | 0.0198 | 0.500 | 0.0207 | | 20 | 0.4789 | 0.0233 | 0.481 | 0.0218 | 0.481 | 0.0214 |
| | 40 | 0.5228 | 0.0193 | 0.521 | 0.0190 | 0.521 | 0.0196 | | 40 | 0.5081 | 0.0221 | 0.515 | 0.0186 | 0.515 | 0.0192 |
| | 60 | 0.5309 | 0.0189 | 0.529 | 0.0185 | 0.531 | 0.0189 | | 60 | 0.5243 | 0.0194 | 0.522 | 0.0193 | 0.525 | 0.0193 |
| | 80 | 0.5323 | 0.0192 | 0.532 | 0.0192 | 0.533 | 0.0190 | | 80 | 0.5317 | 0.0195 | 0.531 | 0.0192 | 0.534 | 0.0195 |
| | 100 | 0.5335 | 0.0193 | 0.535 | 0.0191 | 0.535 | 0.0192 | | 100 | 0.5335 | 0.0193 | 0.535 | 0.0191 | 0.535 | 0.0192 |
| WIClustCD | 10 | 0.4250 | 0.0262 | 0.440 | 0.0243 | 0.426 | 0.0264 | Avg_GRM_self | 10 | 0.4605 | 0.0265 | 0.468 | 0.0243 | 0.458 | 0.0252 |
| | 20 | 0.4761 | 0.0271 | 0.500 | 0.0223 | 0.478 | 0.0268 | | 20 | 0.4962 | 0.0225 | 0.499 | 0.0218 | 0.495 | 0.0224 |
| | 40 | 0.4841 | 0.0254 | 0.488 | 0.0231 | 0.484 | 0.0236 | | 40 | 0.5115 | 0.0228 | 0.516 | 0.0206 | 0.519 | 0.0206 |
| | 60 | 0.5146 | 0.0217 | 0.514 | 0.0216 | 0.515 | 0.0213 | | 60 | 0.5175 | 0.0199 | 0.518 | 0.0191 | 0.521 | 0.0191 |
| | 80 | 0.5209 | 0.0203 | 0.519 | 0.0207 | 0.518 | 0.0201 | | 80 | 0.5298 | 0.0193 | 0.530 | 0.0191 | 0.530 | 0.0191 |
| | 100 | 0.5335 | 0.0193 | 0.535 | 0.0191 | 0.535 | 0.0192 | | 100 | 0.5335 | 0.0193 | 0.535 | 0.0191 | 0.535 | 0.0192 |
| WIClustCDtarg | 10 | 0.4470 | 0.0261 | 0.463 | 0.0224 | 0.457 | 0.0241 | PAM | 10 | 0.4459 | 0.0277 | 0.464 | 0.0245 | 0.443 | 0.0275 |
| | 20 | 0.4913 | 0.0238 | 0.502 | 0.0200 | 0.500 | 0.0206 | | 20 | 0.4789 | 0.0259 | 0.484 | 0.0233 | 0.477 | 0.0253 |
| | 40 | 0.5206 | 0.0196 | 0.521 | 0.0191 | 0.518 | 0.0199 | | 40 | 0.4832 | 0.0287 | 0.502 | 0.0218 | 0.490 | 0.0263 |
| | 60 | 0.5320 | 0.0183 | 0.532 | 0.0182 | 0.531 | 0.0184 | | 60 | 0.5029 | 0.0232 | 0.511 | 0.0198 | 0.508 | 0.0196 |
| | 80 | 0.5306 | 0.0188 | 0.531 | 0.0187 | 0.531 | 0.0186 | | 80 | 0.5136 | 0.0216 | 0.521 | 0.0194 | 0.524 | 0.0192 |
| | 100 | 0.5335 | 0.0193 | 0.535 | 0.0191 | 0.535 | 0.0192 | | 100 | 0.5335 | 0.0193 | 0.535 | 0.0191 | 0.535 | 0.0192 |

**Table S16** Average accuracy and its corresponding standard error of the mean (SEM) across the 40 iterations for all training set optimization methods, models and training set (TRS) sizes (expressed as percentage of the candidate set (CS)) for sorghum dataset and HT trait. If "targ" is added at the end of the name of a method, it corresponds to targeted optimization. Otherwise, untargeted optimization was performed.

**Sorghum trait HT**

| Optimization method | TRS size (% of CS) | Average accuracy | | | | | | Optimization method | TRS size (% of CS) | Average accuracy | | | | | |
|---|---|---|---|---|---|---|---|---|---|---|---|---|---|---|---|
| | | GBLUP | | BayesB | | RKHS | | | | GBLUP | | BayesB | | RKHS | |
| | | Mean | SEM | Mean | SEM | Mean | SEM | | | Mean | SEM | Mean | SEM | Mean | SEM |
| RAND | 10 | 0.408 | 0.0166 | 0.417 | 0.0160 | 0.413 | 0.0160 | Rscore | 10 | 0.428 | 0.0160 | 0.432 | 0.0152 | 0.428 | 0.0158 |
| | 20 | 0.484 | 0.0160 | 0.485 | 0.0157 | 0.488 | 0.0165 | | 20 | 0.474 | 0.0142 | 0.475 | 0.0145 | 0.473 | 0.0137 |
| | 40 | 0.526 | 0.0154 | 0.525 | 0.0151 | 0.530 | 0.0152 | | 40 | 0.530 | 0.0131 | 0.529 | 0.0132 | 0.531 | 0.0130 |
| | 60 | 0.562 | 0.0119 | 0.560 | 0.0122 | 0.560 | 0.0117 | | 60 | 0.561 | 0.0142 | 0.558 | 0.0141 | 0.563 | 0.0141 |
| | 80 | 0.584 | 0.0134 | 0.583 | 0.0130 | 0.584 | 0.0128 | | 80 | 0.591 | 0.0119 | 0.587 | 0.0119 | 0.595 | 0.0117 |
| | 100 | 0.601 | 0.0127 | 0.600 | 0.0126 | 0.602 | 0.0125 | | 100 | 0.601 | 0.0127 | 0.600 | 0.0126 | 0.602 | 0.0125 |
| StratSamp | 10 | 0.418 | 0.0146 | 0.415 | 0.0140 | 0.417 | 0.0144 | Rscoretarg | 10 | 0.483 | 0.0153 | 0.488 | 0.0163 | 0.488 | 0.0151 |
| | 20 | 0.471 | 0.0145 | 0.468 | 0.0141 | 0.472 | 0.0143 | | 20 | 0.522 | 0.0131 | 0.528 | 0.0132 | 0.529 | 0.0132 |
| | 40 | 0.536 | 0.0121 | 0.533 | 0.0123 | 0.534 | 0.0124 | | 40 | 0.561 | 0.0132 | 0.562 | 0.0129 | 0.569 | 0.0126 |
| | 60 | 0.559 | 0.0121 | 0.559 | 0.0124 | 0.561 | 0.0119 | | 60 | 0.583 | 0.0130 | 0.580 | 0.0133 | 0.586 | 0.0127 |
| | 80 | 0.581 | 0.0127 | 0.580 | 0.0124 | 0.581 | 0.0127 | | 80 | 0.602 | 0.0124 | 0.600 | 0.0125 | 0.602 | 0.0124 |
| | 100 | 0.601 | 0.0127 | 0.600 | 0.0126 | 0.602 | 0.0125 | | 100 | 0.601 | 0.0127 | 0.600 | 0.0126 | 0.602 | 0.0125 |
| CD | 10 | 0.457 | 0.0150 | 0.463 | 0.0143 | 0.461 | 0.0146 | Avg_GRM | 10 | 0.128 | 0.0306 | 0.117 | 0.0328 | 0.138 | 0.0273 |
| | 20 | 0.504 | 0.0133 | 0.510 | 0.0135 | 0.511 | 0.0130 | | 20 | 0.216 | 0.0271 | 0.221 | 0.0249 | 0.218 | 0.0264 |
| | 40 | 0.521 | 0.0147 | 0.524 | 0.0148 | 0.525 | 0.0147 | | 40 | 0.356 | 0.0276 | 0.359 | 0.0274 | 0.365 | 0.0258 |
| | 60 | 0.562 | 0.0143 | 0.560 | 0.0142 | 0.565 | 0.0141 | | 60 | 0.482 | 0.0175 | 0.483 | 0.0177 | 0.487 | 0.0178 |
| | 80 | 0.584 | 0.0124 | 0.581 | 0.0128 | 0.591 | 0.0123 | | 80 | 0.538 | 0.0145 | 0.537 | 0.0141 | 0.542 | 0.0140 |
| | 100 | 0.601 | 0.0127 | 0.600 | 0.0126 | 0.602 | 0.0125 | | 100 | 0.601 | 0.0127 | 0.600 | 0.0126 | 0.602 | 0.0125 |
| CDtarg | 10 | 0.543 | 0.0135 | 0.543 | 0.0136 | 0.550 | 0.0135 | Avg_GRMtarg | 10 | 0.272 | 0.0252 | 0.270 | 0.0280 | 0.274 | 0.0232 |
| | 20 | 0.566 | 0.0135 | 0.563 | 0.0130 | 0.567 | 0.0134 | | 20 | 0.342 | 0.0243 | 0.342 | 0.0244 | 0.348 | 0.0221 |
| | 40 | 0.588 | 0.0129 | 0.586 | 0.0131 | 0.588 | 0.0122 | | 40 | 0.479 | 0.0187 | 0.478 | 0.0191 | 0.475 | 0.0183 |
| | 60 | 0.595 | 0.0122 | 0.594 | 0.0127 | 0.594 | 0.0121 | | 60 | 0.548 | 0.0155 | 0.549 | 0.0153 | 0.548 | 0.0155 |
| | 80 | 0.597 | 0.0129 | 0.596 | 0.0125 | 0.597 | 0.0127 | | 80 | 0.587 | 0.0130 | 0.586 | 0.0129 | 0.588 | 0.0126 |
| | 100 | 0.601 | 0.0127 | 0.600 | 0.0126 | 0.602 | 0.0125 | | 100 | 0.601 | 0.0127 | 0.600 | 0.0126 | 0.602 | 0.0125 |
| OvClustCD | 10 | 0.467 | 0.0141 | 0.477 | 0.0145 | 0.469 | 0.0140 | Avg_GRM_MinMax | 10 | 0.445 | 0.0156 | 0.451 | 0.0160 | 0.444 | 0.0155 |
| | 20 | 0.500 | 0.0127 | 0.503 | 0.0128 | 0.503 | 0.0124 | | 20 | 0.497 | 0.0140 | 0.499 | 0.0144 | 0.500 | 0.0140 |
| | 40 | 0.521 | 0.0136 | 0.519 | 0.0134 | 0.522 | 0.0137 | | 40 | 0.551 | 0.0127 | 0.552 | 0.0127 | 0.555 | 0.0127 |
| | 60 | 0.563 | 0.0127 | 0.558 | 0.0129 | 0.568 | 0.0128 | | 60 | 0.576 | 0.0138 | 0.572 | 0.0140 | 0.579 | 0.0136 |
| | 80 | 0.587 | 0.0133 | 0.582 | 0.0134 | 0.592 | 0.0131 | | 80 | 0.596 | 0.0129 | 0.594 | 0.0129 | 0.598 | 0.0125 |
| | 100 | 0.601 | 0.0127 | 0.600 | 0.0126 | 0.602 | 0.0125 | | 100 | 0.601 | 0.0127 | 0.600 | 0.0126 | 0.602 | 0.0125 |
| OvClustCDtarg | 10 | 0.534 | 0.0160 | 0.537 | 0.0154 | 0.545 | 0.0154 | Avg_GRM_MinMaxtarg | 10 | 0.491 | 0.0134 | 0.503 | 0.0135 | 0.493 | 0.0131 |
| | 20 | 0.569 | 0.0125 | 0.567 | 0.0125 | 0.571 | 0.0121 | | 20 | 0.539 | 0.0125 | 0.539 | 0.0127 | 0.544 | 0.0121 |
| | 40 | 0.590 | 0.0127 | 0.587 | 0.0124 | 0.589 | 0.0120 | | 40 | 0.582 | 0.0125 | 0.581 | 0.0134 | 0.585 | 0.0122 |
| | 60 | 0.597 | 0.0124 | 0.595 | 0.0123 | 0.597 | 0.0121 | | 60 | 0.590 | 0.0136 | 0.589 | 0.0136 | 0.593 | 0.0132 |
| | 80 | 0.598 | 0.0125 | 0.597 | 0.0123 | 0.598 | 0.0125 | | 80 | 0.596 | 0.0126 | 0.594 | 0.0126 | 0.599 | 0.0124 |
| | 100 | 0.601 | 0.0127 | 0.600 | 0.0126 | 0.602 | 0.0125 | | 100 | 0.601 | 0.0127 | 0.600 | 0.0126 | 0.602 | 0.0125 |
| WIClustCD | 10 | 0.480 | 0.0126 | 0.487 | 0.0128 | 0.483 | 0.0125 | Avg_GRM_self | 10 | 0.475 | 0.0144 | 0.482 | 0.0144 | 0.477 | 0.0137 |
| | 20 | 0.491 | 0.0136 | 0.498 | 0.0134 | 0.493 | 0.0134 | | 20 | 0.490 | 0.0153 | 0.499 | 0.0152 | 0.497 | 0.0152 |
| | 40 | 0.531 | 0.0152 | 0.530 | 0.0145 | 0.530 | 0.0149 | | 40 | 0.564 | 0.0132 | 0.562 | 0.0129 | 0.570 | 0.0131 |
| | 60 | 0.558 | 0.0139 | 0.557 | 0.0142 | 0.564 | 0.0139 | | 60 | 0.587 | 0.0119 | 0.583 | 0.0121 | 0.589 | 0.0120 |
| | 80 | 0.585 | 0.0127 | 0.584 | 0.0130 | 0.590 | 0.0125 | | 80 | 0.597 | 0.0128 | 0.595 | 0.0127 | 0.600 | 0.0126 |
| | 100 | 0.601 | 0.0127 | 0.600 | 0.0126 | 0.602 | 0.0125 | | 100 | 0.601 | 0.0127 | 0.600 | 0.0126 | 0.602 | 0.0125 |
| WIClustCDtarg | 10 | 0.530 | 0.0156 | 0.534 | 0.0153 | 0.538 | 0.0154 | PAM | 10 | 0.448 | 0.0136 | 0.452 | 0.0144 | 0.451 | 0.0132 |
| | 20 | 0.569 | 0.0143 | 0.568 | 0.0139 | 0.572 | 0.0126 | | 20 | 0.480 | 0.0141 | 0.481 | 0.0144 | 0.480 | 0.0139 |
| | 40 | 0.586 | 0.0129 | 0.586 | 0.0129 | 0.586 | 0.0124 | | 40 | 0.518 | 0.0150 | 0.518 | 0.0150 | 0.524 | 0.0151 |
| | 60 | 0.596 | 0.0128 | 0.596 | 0.0126 | 0.596 | 0.0125 | | 60 | 0.556 | 0.0135 | 0.559 | 0.0139 | 0.563 | 0.0134 |
| | 80 | 0.601 | 0.0130 | 0.599 | 0.0128 | 0.601 | 0.0128 | | 80 | 0.583 | 0.0127 | 0.582 | 0.0128 | 0.589 | 0.0127 |
| | 100 | 0.601 | 0.0127 | 0.600 | 0.0126 | 0.602 | 0.0125 | | 100 | 0.601 | 0.0127 | 0.600 | 0.0126 | 0.602 | 0.0125 |

**Table S17** Average accuracy and its corresponding standard error of the mean (SEM) across the 40 iterations for all training set optimization methods, models and training set (TRS) sizes (expressed as percentage of the candidate set (CS)) for sorghum dataset and MO trait. If ”targ” is added at the end of the name of a method, it corresponds to targeted optimization. Otherwise, untargeted optimization was performed.

**Sorghum trait MO**

| Optimization method | TRS size (% of CS) | Average accuracy | | | | | | Optimization method | TRS size (% of CS) | Average accuracy | | | | | |
|---|---|---|---|---|---|---|---|---|---|---|---|---|---|---|---|
| | | GBLUP | | BayesB | | RKHS | | | | GBLUP | | BayesB | | RKHS | |
| | | Mean | SEM | Mean | SEM | Mean | SEM | | | Mean | SEM | Mean | SEM | Mean | SEM |
| RAND | 10 | 0.396 | 0.0167 | 0.3961 | 0.0174 | 0.3993 | 0.0178 | Rscore | 10 | 0.378 | 0.0174 | 0.3880 | 0.0160 | 0.3847 | 0.0172 |
| | 20 | 0.446 | 0.0135 | 0.4479 | 0.0138 | 0.4512 | 0.0134 | | 20 | 0.452 | 0.0166 | 0.4551 | 0.0175 | 0.4506 | 0.0171 |
| | 40 | 0.510 | 0.0124 | 0.5130 | 0.0126 | 0.5114 | 0.0125 | | 40 | 0.514 | 0.0133 | 0.5167 | 0.0133 | 0.5150 | 0.0138 |
| | 60 | 0.549 | 0.0143 | 0.5504 | 0.0140 | 0.5495 | 0.0143 | | 60 | 0.546 | 0.0126 | 0.5452 | 0.0128 | 0.5442 | 0.0125 |
| | 80 | 0.568 | 0.0132 | 0.5678 | 0.0132 | 0.5689 | 0.0131 | | 80 | 0.565 | 0.0116 | 0.5648 | 0.0115 | 0.5665 | 0.0114 |
| | 100 | 0.576 | 0.0121 | 0.5769 | 0.0118 | 0.5778 | 0.0120 | | 100 | 0.576 | 0.0121 | 0.5769 | 0.0118 | 0.5778 | 0.0120 |
| StratSamp | 10 | 0.394 | 0.0165 | 0.3949 | 0.0168 | 0.3953 | 0.0168 | Rscoretarg | 10 | 0.433 | 0.0160 | 0.4380 | 0.0159 | 0.4375 | 0.0154 |
| | 20 | 0.460 | 0.0167 | 0.4663 | 0.0160 | 0.4632 | 0.0163 | | 20 | 0.498 | 0.0159 | 0.4973 | 0.0158 | 0.4947 | 0.0165 |
| | 40 | 0.514 | 0.0136 | 0.5175 | 0.0132 | 0.5153 | 0.0138 | | 40 | 0.555 | 0.0130 | 0.5550 | 0.0131 | 0.5545 | 0.0132 |
| | 60 | 0.544 | 0.0123 | 0.5447 | 0.0125 | 0.5457 | 0.0124 | | 60 | 0.564 | 0.0128 | 0.5641 | 0.0127 | 0.5655 | 0.0127 |
| | 80 | 0.565 | 0.0123 | 0.5656 | 0.0121 | 0.5662 | 0.0122 | | 80 | 0.571 | 0.0129 | 0.5711 | 0.0129 | 0.5724 | 0.0129 |
| | 100 | 0.576 | 0.0121 | 0.5769 | 0.0118 | 0.5778 | 0.0120 | | 100 | 0.576 | 0.0121 | 0.5769 | 0.0118 | 0.5778 | 0.0120 |
| CD | 10 | 0.401 | 0.0143 | 0.3956 | 0.0147 | 0.4019 | 0.0148 | Avg_GRM | 10 | 0.074 | 0.0241 | 0.0741 | 0.0241 | 0.0996 | 0.0219 |
| | 20 | 0.462 | 0.0134 | 0.4702 | 0.0129 | 0.4613 | 0.0137 | | 20 | 0.215 | 0.0208 | 0.2181 | 0.0207 | 0.2193 | 0.0204 |
| | 40 | 0.519 | 0.0155 | 0.5246 | 0.0149 | 0.5195 | 0.0155 | | 40 | 0.344 | 0.0259 | 0.3385 | 0.0264 | 0.3524 | 0.0247 |
| | 60 | 0.552 | 0.0130 | 0.5539 | 0.0131 | 0.5517 | 0.0130 | | 60 | 0.438 | 0.0202 | 0.4404 | 0.0205 | 0.4396 | 0.0198 |
| | 80 | 0.567 | 0.0126 | 0.5686 | 0.0127 | 0.5687 | 0.0130 | | 80 | 0.512 | 0.0142 | 0.5120 | 0.0144 | 0.5133 | 0.0145 |
| | 100 | 0.576 | 0.0121 | 0.5769 | 0.0118 | 0.5778 | 0.0120 | | 100 | 0.576 | 0.0121 | 0.5769 | 0.0118 | 0.5778 | 0.0120 |
| CDtarg | 10 | 0.469 | 0.0161 | 0.4707 | 0.0168 | 0.4720 | 0.0159 | Avg_GRMtarg | 10 | 0.206 | 0.0302 | 0.2136 | 0.0289 | 0.2283 | 0.0284 |
| | 20 | 0.523 | 0.0122 | 0.5233 | 0.0124 | 0.5245 | 0.0121 | | 20 | 0.335 | 0.0222 | 0.3392 | 0.0230 | 0.3450 | 0.0213 |
| | 40 | 0.551 | 0.0126 | 0.5507 | 0.0122 | 0.5526 | 0.0126 | | 40 | 0.448 | 0.0160 | 0.4475 | 0.0163 | 0.4549 | 0.0154 |
| | 60 | 0.567 | 0.0124 | 0.5667 | 0.0122 | 0.5695 | 0.0123 | | 60 | 0.507 | 0.0133 | 0.5063 | 0.0137 | 0.5096 | 0.0130 |
| | 80 | 0.571 | 0.0122 | 0.5706 | 0.0121 | 0.5732 | 0.0122 | | 80 | 0.557 | 0.0115 | 0.5585 | 0.0118 | 0.5593 | 0.0112 |
| | 100 | 0.576 | 0.0121 | 0.5769 | 0.0118 | 0.5778 | 0.0120 | | 100 | 0.576 | 0.0121 | 0.5769 | 0.0118 | 0.5778 | 0.0120 |
| OvClustCD | 10 | 0.413 | 0.0145 | 0.4095 | 0.0148 | 0.4223 | 0.0147 | Avg_GRM_MinMax | 10 | 0.415 | 0.0131 | 0.4187 | 0.0144 | 0.4149 | 0.0138 |
| | 20 | 0.455 | 0.0130 | 0.4574 | 0.0122 | 0.4519 | 0.0132 | | 20 | 0.476 | 0.0152 | 0.4790 | 0.0151 | 0.4810 | 0.0148 |
| | 40 | 0.504 | 0.0133 | 0.5100 | 0.0129 | 0.5044 | 0.0132 | | 40 | 0.522 | 0.0127 | 0.5238 | 0.0129 | 0.5246 | 0.0128 |
| | 60 | 0.541 | 0.0113 | 0.5492 | 0.0107 | 0.5420 | 0.0115 | | 60 | 0.545 | 0.0127 | 0.5462 | 0.0126 | 0.5480 | 0.0125 |
| | 80 | 0.558 | 0.0110 | 0.5619 | 0.0109 | 0.5577 | 0.0111 | | 80 | 0.572 | 0.0120 | 0.5707 | 0.0121 | 0.5727 | 0.0122 |
| | 100 | 0.576 | 0.0121 | 0.5769 | 0.0118 | 0.5778 | 0.0120 | | 100 | 0.576 | 0.0121 | 0.5769 | 0.0118 | 0.5778 | 0.0120 |
| OvClustCDtarg | 10 | 0.475 | 0.0146 | 0.4799 | 0.0148 | 0.4788 | 0.0143 | Avg_GRM_MinMaxtarg | 10 | 0.469 | 0.0129 | 0.4696 | 0.0127 | 0.4699 | 0.0129 |
| | 20 | 0.519 | 0.0113 | 0.5203 | 0.0115 | 0.5221 | 0.0112 | | 20 | 0.519 | 0.0142 | 0.5200 | 0.0141 | 0.5174 | 0.0144 |
| | 40 | 0.551 | 0.0128 | 0.5510 | 0.0128 | 0.5538 | 0.0128 | | 40 | 0.544 | 0.0131 | 0.5456 | 0.0129 | 0.5469 | 0.0131 |
| | 60 | 0.565 | 0.0125 | 0.5644 | 0.0127 | 0.5681 | 0.0125 | | 60 | 0.567 | 0.0123 | 0.5693 | 0.0119 | 0.5690 | 0.0122 |
| | 80 | 0.571 | 0.0123 | 0.5723 | 0.0121 | 0.5730 | 0.0123 | | 80 | 0.574 | 0.0117 | 0.5744 | 0.0117 | 0.5760 | 0.0116 |
| | 100 | 0.576 | 0.0121 | 0.5769 | 0.0118 | 0.5778 | 0.0120 | | 100 | 0.576 | 0.0121 | 0.5769 | 0.0118 | 0.5778 | 0.0120 |
| WIClustCD | 10 | 0.386 | 0.0144 | 0.3925 | 0.0160 | 0.3893 | 0.0142 | Avg_GRM_self | 10 | 0.428 | 0.0139 | 0.4264 | 0.0147 | 0.4261 | 0.0148 |
| | 20 | 0.448 | 0.0162 | 0.4524 | 0.0164 | 0.4455 | 0.0157 | | 20 | 0.497 | 0.0136 | 0.4941 | 0.0132 | 0.4972 | 0.0133 |
| | 40 | 0.512 | 0.0151 | 0.5195 | 0.0143 | 0.5126 | 0.0148 | | 40 | 0.544 | 0.0128 | 0.5466 | 0.0126 | 0.5457 | 0.0130 |
| | 60 | 0.538 | 0.0117 | 0.5482 | 0.0113 | 0.5385 | 0.0114 | | 60 | 0.559 | 0.0139 | 0.5602 | 0.0135 | 0.5610 | 0.0136 |
| | 80 | 0.556 | 0.0120 | 0.5612 | 0.0117 | 0.5556 | 0.0119 | | 80 | 0.572 | 0.0124 | 0.5726 | 0.0121 | 0.5735 | 0.0122 |
| | 100 | 0.576 | 0.0121 | 0.5769 | 0.0118 | 0.5778 | 0.0120 | | 100 | 0.576 | 0.0121 | 0.5769 | 0.0118 | 0.5778 | 0.0120 |
| WIClustCDtarg | 10 | 0.470 | 0.0158 | 0.4786 | 0.0161 | 0.4758 | 0.0158 | PAM | 10 | 0.445 | 0.0186 | 0.4619 | 0.0160 | 0.4520 | 0.0174 |
| | 20 | 0.513 | 0.0121 | 0.5130 | 0.0124 | 0.5162 | 0.0121 | | 20 | 0.508 | 0.0122 | 0.5016 | 0.0122 | 0.5097 | 0.0121 |
| | 40 | 0.554 | 0.0124 | 0.5540 | 0.0125 | 0.5573 | 0.0125 | | 40 | 0.551 | 0.0130 | 0.5468 | 0.0127 | 0.5524 | 0.0130 |
| | 60 | 0.566 | 0.0124 | 0.5664 | 0.0123 | 0.5683 | 0.0124 | | 60 | 0.563 | 0.0119 | 0.5602 | 0.0118 | 0.5668 | 0.0118 |
| | 80 | 0.576 | 0.0121 | 0.5755 | 0.0122 | 0.5774 | 0.0122 | | 80 | 0.566 | 0.0121 | 0.5667 | 0.0121 | 0.5681 | 0.0121 |
| | 100 | 0.576 | 0.0121 | 0.5769 | 0.0118 | 0.5778 | 0.0120 | | 100 | 0.576 | 0.0121 | 0.5769 | 0.0118 | 0.5778 | 0.0120 |

**Table S18** Average accuracy and its corresponding standard error of the mean (SEM) across the 40 iterations for all training set optimization methods, models and training set (TRS) sizes (expressed as percentage of the candidate set (CS)) for sorghum dataset and YLD trait. If "targ" is added at the end of the name of a method, it corresponds to targeted optimization. Otherwise, untargeted optimization was performed.

**Sorghum trait YLD**

| Optimization method | TRS size (% of CS) | Average accuracy | | | | | | Optimization method | TRS size (% of CS) | Average accuracy | | | | | |
|---|---|---|---|---|---|---|---|---|---|---|---|---|---|---|---|
| | | GBLUP | | BayesB | | RKHS | | | | GBLUP | | BayesB | | RKHS | |
| | | Mean | SEM | Mean | SEM | Mean | SEM | | | Mean | SEM | Mean | SEM | Mean | SEM |
| RAND | 10 | 0.1979 | 0.0212 | 0.2065 | 0.0221 | 0.201 | 0.0206 | Rscore | 10 | 0.2429 | 0.0184 | 0.2508 | 0.0186 | 0.247 | 0.0187 |
| | 20 | 0.2764 | 0.0189 | 0.2830 | 0.0196 | 0.280 | 0.0193 | | 20 | 0.2760 | 0.0209 | 0.2766 | 0.0196 | 0.278 | 0.0197 |
| | 40 | 0.3206 | 0.0179 | 0.3209 | 0.0178 | 0.328 | 0.0179 | | 40 | 0.3071 | 0.0180 | 0.3060 | 0.0182 | 0.313 | 0.0182 |
| | 60 | 0.3482 | 0.0181 | 0.3553 | 0.0178 | 0.357 | 0.0177 | | 60 | 0.3572 | 0.0184 | 0.3649 | 0.0182 | 0.364 | 0.0188 |
| | 80 | 0.3770 | 0.0163 | 0.3806 | 0.0159 | 0.385 | 0.0167 | | 80 | 0.3765 | 0.0172 | 0.3805 | 0.0173 | 0.386 | 0.0171 |
| | 100 | 0.3899 | 0.0172 | 0.3923 | 0.0167 | 0.399 | 0.0172 | | 100 | 0.3899 | 0.0172 | 0.3923 | 0.0167 | 0.399 | 0.0172 |
| StratSamp | 10 | 0.2402 | 0.0195 | 0.2434 | 0.0196 | 0.243 | 0.0195 | Rscoretarg | 10 | 0.2697 | 0.0213 | 0.2756 | 0.0207 | 0.277 | 0.0209 |
| | 20 | 0.2827 | 0.0201 | 0.2877 | 0.0186 | 0.287 | 0.0193 | | 20 | 0.3024 | 0.0216 | 0.3073 | 0.0219 | 0.309 | 0.0223 |
| | 40 | 0.3388 | 0.0187 | 0.3437 | 0.0189 | 0.342 | 0.0185 | | 40 | 0.3583 | 0.0170 | 0.3590 | 0.0160 | 0.366 | 0.0165 |
| | 60 | 0.3611 | 0.0160 | 0.3659 | 0.0160 | 0.369 | 0.0164 | | 60 | 0.3667 | 0.0173 | 0.3695 | 0.0168 | 0.372 | 0.0166 |
| | 80 | 0.3787 | 0.0163 | 0.3834 | 0.0159 | 0.387 | 0.0166 | | 80 | 0.3803 | 0.0175 | 0.3848 | 0.0171 | 0.387 | 0.0180 |
| | 100 | 0.3899 | 0.0172 | 0.3923 | 0.0167 | 0.399 | 0.0172 | | 100 | 0.3899 | 0.0172 | 0.3923 | 0.0167 | 0.399 | 0.0172 |
| CD | 10 | 0.2467 | 0.0214 | 0.2549 | 0.0198 | 0.259 | 0.0200 | Avg_GRM | 10 | 0.0498 | 0.0232 | 0.0533 | 0.0237 | 0.064 | 0.0205 |
| | 20 | 0.3075 | 0.0188 | 0.3049 | 0.0197 | 0.312 | 0.0190 | | 20 | 0.0987 | 0.0231 | 0.0880 | 0.0225 | 0.103 | 0.0223 |
| | 40 | 0.2913 | 0.0190 | 0.3025 | 0.0185 | 0.297 | 0.0192 | | 40 | 0.1614 | 0.0289 | 0.1579 | 0.0292 | 0.179 | 0.0277 |
| | 60 | 0.3419 | 0.0173 | 0.3542 | 0.0175 | 0.355 | 0.0178 | | 60 | 0.2358 | 0.0236 | 0.2305 | 0.0231 | 0.252 | 0.0218 |
| | 80 | 0.3630 | 0.0189 | 0.3640 | 0.0185 | 0.368 | 0.0189 | | 80 | 0.3197 | 0.0168 | 0.3210 | 0.0171 | 0.332 | 0.0157 |
| | 100 | 0.3899 | 0.0172 | 0.3923 | 0.0167 | 0.399 | 0.0172 | | 100 | 0.3899 | 0.0172 | 0.3923 | 0.0167 | 0.399 | 0.0172 |
| CDtarg | 10 | 0.3108 | 0.0196 | 0.3270 | 0.0165 | 0.316 | 0.0191 | Avg_GRMtarg | 10 | 0.1752 | 0.0253 | 0.1805 | 0.0257 | 0.179 | 0.0231 |
| | 20 | 0.3376 | 0.0188 | 0.3470 | 0.0183 | 0.341 | 0.0193 | | 20 | 0.2061 | 0.0270 | 0.2031 | 0.0273 | 0.212 | 0.0253 |
| | 40 | 0.3682 | 0.0180 | 0.3692 | 0.0176 | 0.371 | 0.0184 | | 40 | 0.2562 | 0.0262 | 0.2729 | 0.0227 | 0.273 | 0.0256 |
| | 60 | 0.3804 | 0.0177 | 0.3815 | 0.0173 | 0.384 | 0.0182 | | 60 | 0.3031 | 0.0222 | 0.3040 | 0.0215 | 0.320 | 0.0214 |
| | 80 | 0.3872 | 0.0173 | 0.3881 | 0.0170 | 0.392 | 0.0176 | | 80 | 0.3728 | 0.0191 | 0.3760 | 0.0187 | 0.384 | 0.0191 |
| | 100 | 0.3899 | 0.0172 | 0.3923 | 0.0167 | 0.399 | 0.0172 | | 100 | 0.3899 | 0.0172 | 0.3923 | 0.0167 | 0.399 | 0.0172 |
| OvClustCD | 10 | 0.2410 | 0.0190 | 0.2568 | 0.0195 | 0.247 | 0.0187 | Avg_GRM_MinMax | 10 | 0.2287 | 0.0202 | 0.2397 | 0.0183 | 0.232 | 0.0198 |
| | 20 | 0.2869 | 0.0196 | 0.2965 | 0.0189 | 0.297 | 0.0197 | | 20 | 0.2984 | 0.0203 | 0.3078 | 0.0198 | 0.304 | 0.0199 |
| | 40 | 0.3027 | 0.0184 | 0.3097 | 0.0184 | 0.307 | 0.0183 | | 40 | 0.3427 | 0.0174 | 0.3461 | 0.0176 | 0.347 | 0.0184 |
| | 60 | 0.3446 | 0.0172 | 0.3476 | 0.0172 | 0.350 | 0.0170 | | 60 | 0.3689 | 0.0178 | 0.3745 | 0.0171 | 0.377 | 0.0179 |
| | 80 | 0.3726 | 0.0180 | 0.3755 | 0.0176 | 0.383 | 0.0176 | | 80 | 0.3785 | 0.0180 | 0.3834 | 0.0177 | 0.388 | 0.0180 |
| | 100 | 0.3899 | 0.0172 | 0.3923 | 0.0167 | 0.399 | 0.0172 | | 100 | 0.3899 | 0.0172 | 0.3923 | 0.0167 | 0.399 | 0.0172 |
| OvClustCDtarg | 10 | 0.2795 | 0.0194 | 0.3058 | 0.0178 | 0.291 | 0.0185 | Avg_GRM_MinMaxtarg | 10 | 0.2741 | 0.0198 | 0.2891 | 0.0175 | 0.279 | 0.0195 |
| | 20 | 0.3339 | 0.0187 | 0.3460 | 0.0181 | 0.340 | 0.0188 | | 20 | 0.3209 | 0.0163 | 0.3311 | 0.0157 | 0.330 | 0.0167 |
| | 40 | 0.3699 | 0.0176 | 0.3738 | 0.0171 | 0.373 | 0.0181 | | 40 | 0.3602 | 0.0182 | 0.3613 | 0.0175 | 0.364 | 0.0185 |
| | 60 | 0.3784 | 0.0180 | 0.3830 | 0.0177 | 0.382 | 0.0183 | | 60 | 0.3799 | 0.0184 | 0.3847 | 0.0176 | 0.384 | 0.0190 |
| | 80 | 0.3875 | 0.0176 | 0.3895 | 0.0173 | 0.392 | 0.0178 | | 80 | 0.3806 | 0.0176 | 0.3850 | 0.0175 | 0.388 | 0.0178 |
| | 100 | 0.3899 | 0.0172 | 0.3923 | 0.0167 | 0.399 | 0.0172 | | 100 | 0.3899 | 0.0172 | 0.3923 | 0.0167 | 0.399 | 0.0172 |
| WIClustCD | 10 | 0.2427 | 0.0180 | 0.2674 | 0.0195 | 0.251 | 0.0177 | Avg_GRM_self | 10 | 0.2572 | 0.0250 | 0.2644 | 0.0252 | 0.257 | 0.0253 |
| | 20 | 0.2896 | 0.0190 | 0.2937 | 0.0184 | 0.297 | 0.0184 | | 20 | 0.2960 | 0.0195 | 0.2996 | 0.0187 | 0.302 | 0.0197 |
| | 40 | 0.3205 | 0.0171 | 0.3257 | 0.0174 | 0.339 | 0.0174 | | 40 | 0.3422 | 0.0199 | 0.3494 | 0.0192 | 0.352 | 0.0192 |
| | 60 | 0.3414 | 0.0177 | 0.3407 | 0.0172 | 0.349 | 0.0171 | | 60 | 0.3608 | 0.0167 | 0.3675 | 0.0161 | 0.367 | 0.0164 |
| | 80 | 0.3739 | 0.0188 | 0.3775 | 0.0184 | 0.382 | 0.0188 | | 80 | 0.3852 | 0.0176 | 0.3900 | 0.0175 | 0.392 | 0.0178 |
| | 100 | 0.3899 | 0.0172 | 0.3923 | 0.0167 | 0.399 | 0.0172 | | 100 | 0.3899 | 0.0172 | 0.3923 | 0.0167 | 0.399 | 0.0172 |
| WIClustCDtarg | 10 | 0.2775 | 0.0245 | 0.3092 | 0.0216 | 0.290 | 0.0232 | PAM | 10 | 0.2474 | 0.0233 | 0.2381 | 0.0234 | 0.247 | 0.0230 |
| | 20 | 0.3305 | 0.0187 | 0.3417 | 0.0177 | 0.339 | 0.0180 | | 20 | 0.2457 | 0.0218 | 0.2472 | 0.0212 | 0.249 | 0.0223 |
| | 40 | 0.3620 | 0.0170 | 0.3645 | 0.0170 | 0.366 | 0.0179 | | 40 | 0.2878 | 0.0216 | 0.2833 | 0.0213 | 0.293 | 0.0217 |
| | 60 | 0.3771 | 0.0176 | 0.3802 | 0.0169 | 0.384 | 0.0180 | | 60 | 0.3504 | 0.0199 | 0.3518 | 0.0199 | 0.358 | 0.0204 |
| | 80 | 0.3835 | 0.0175 | 0.3876 | 0.0170 | 0.391 | 0.0176 | | 80 | 0.3689 | 0.0193 | 0.3729 | 0.0189 | 0.378 | 0.0191 |
| | 100 | 0.3899 | 0.0172 | 0.3923 | 0.0167 | 0.399 | 0.0172 | | 100 | 0.3899 | 0.0172 | 0.3923 | 0.0167 | 0.399 | 0.0172 |

**Table S19** Average accuracy and its corresponding standard error of the mean (SEM) across the 40 iterations for all training set optimization methods, models and training set (TRS) sizes (expressed as percentage of the candidate set (CS)) for sorghum dataset and the simulated trait. If "targ" is added at the end of the name of a method, it corresponds to targeted optimization. Otherwise, untargeted optimization was performed.

**Sorghum trait simulated1**

| Optimization method | TRS size (% of CS) | Average accuracy | | | | | | Optimization method | TRS size (% of CS) | Average accuracy | | | | | |
|---|---|---|---|---|---|---|---|---|---|---|---|---|---|---|---|
| | | GBLUP | | BayesB | | RKHS | | | | GBLUP | | BayesB | | RKHS | |
| | | Mean | SEM | Mean | SEM | Mean | SEM | | | Mean | SEM | Mean | SEM | Mean | SEM |
| RAND | 10 | 0.342 | 0.0301 | 0.341 | 0.0293 | 0.343 | 0.0301 | Rscore | 10 | 0.345 | 0.0221 | 0.346 | 0.0207 | 0.345 | 0.0220 |
| | 20 | 0.386 | 0.0266 | 0.393 | 0.0251 | 0.383 | 0.0267 | | 20 | 0.390 | 0.0203 | 0.396 | 0.0201 | 0.390 | 0.0203 |
| | 40 | 0.414 | 0.0260 | 0.420 | 0.0252 | 0.412 | 0.0262 | | 40 | 0.416 | 0.0188 | 0.421 | 0.0189 | 0.416 | 0.0187 |
| | 60 | 0.441 | 0.0256 | 0.442 | 0.0254 | 0.440 | 0.0254 | | 60 | 0.433 | 0.0183 | 0.433 | 0.0185 | 0.432 | 0.0183 |
| | 80 | 0.451 | 0.0242 | 0.451 | 0.0245 | 0.452 | 0.0240 | | 80 | 0.447 | 0.0174 | 0.448 | 0.0176 | 0.446 | 0.0178 |
| | 100 | 0.438 | 0.0183 | 0.439 | 0.0180 | 0.436 | 0.0187 | | 100 | 0.438 | 0.0183 | 0.439 | 0.0180 | 0.436 | 0.0187 |
| StratSamp | 10 | 0.348 | 0.0269 | 0.347 | 0.0265 | 0.347 | 0.0268 | Rscoretarg | 10 | 0.369 | 0.0248 | 0.376 | 0.0235 | 0.370 | 0.0240 |
| | 20 | 0.403 | 0.0194 | 0.407 | 0.0188 | 0.404 | 0.0188 | | 20 | 0.411 | 0.0220 | 0.410 | 0.0215 | 0.412 | 0.0214 |
| | 40 | 0.422 | 0.0226 | 0.423 | 0.0224 | 0.420 | 0.0221 | | 40 | 0.427 | 0.0230 | 0.432 | 0.0221 | 0.424 | 0.0226 |
| | 60 | 0.459 | 0.0198 | 0.456 | 0.0199 | 0.458 | 0.0197 | | 60 | 0.451 | 0.0210 | 0.453 | 0.0207 | 0.451 | 0.0209 |
| | 80 | 0.475 | 0.0191 | 0.474 | 0.0191 | 0.474 | 0.0192 | | 80 | 0.461 | 0.0209 | 0.462 | 0.0207 | 0.460 | 0.0209 |
| | 100 | 0.438 | 0.0183 | 0.439 | 0.0180 | 0.436 | 0.0187 | | 100 | 0.438 | 0.0183 | 0.439 | 0.0180 | 0.436 | 0.0187 |
| CD | 10 | 0.360 | 0.0273 | 0.372 | 0.0250 | 0.359 | 0.0270 | Avg_GRM | 10 | 0.101 | 0.0322 | 0.104 | 0.0320 | 0.106 | 0.0310 |
| | 20 | 0.370 | 0.0283 | 0.375 | 0.0263 | 0.369 | 0.0281 | | 20 | 0.174 | 0.0279 | 0.188 | 0.0280 | 0.177 | 0.0262 |
| | 40 | 0.414 | 0.0248 | 0.414 | 0.0244 | 0.413 | 0.0241 | | 40 | 0.276 | 0.0239 | 0.285 | 0.0222 | 0.277 | 0.0232 |
| | 60 | 0.454 | 0.0216 | 0.455 | 0.0209 | 0.454 | 0.0203 | | 60 | 0.335 | 0.0217 | 0.337 | 0.0215 | 0.337 | 0.0213 |
| | 80 | 0.468 | 0.0212 | 0.467 | 0.0206 | 0.468 | 0.0204 | | 80 | 0.385 | 0.0206 | 0.385 | 0.0199 | 0.389 | 0.0199 |
| | 100 | 0.438 | 0.0183 | 0.439 | 0.0180 | 0.436 | 0.0187 | | 100 | 0.438 | 0.0183 | 0.439 | 0.0180 | 0.436 | 0.0187 |
| CDtarg | 10 | 0.377 | 0.0231 | 0.387 | 0.0197 | 0.373 | 0.0226 | Avg_GRMtarg | 10 | 0.171 | 0.0273 | 0.180 | 0.0290 | 0.172 | 0.0271 |
| | 20 | 0.412 | 0.0201 | 0.411 | 0.0192 | 0.410 | 0.0199 | | 20 | 0.248 | 0.0285 | 0.261 | 0.0275 | 0.255 | 0.0272 |
| | 40 | 0.440 | 0.0174 | 0.441 | 0.0172 | 0.441 | 0.0176 | | 40 | 0.348 | 0.0262 | 0.356 | 0.0248 | 0.355 | 0.0243 |
| | 60 | 0.450 | 0.0172 | 0.453 | 0.0169 | 0.450 | 0.0173 | | 60 | 0.406 | 0.0245 | 0.412 | 0.0226 | 0.405 | 0.0243 |
| | 80 | 0.456 | 0.0163 | 0.455 | 0.0162 | 0.455 | 0.0166 | | 80 | 0.444 | 0.0193 | 0.445 | 0.0186 | 0.445 | 0.0193 |
| | 100 | 0.438 | 0.0183 | 0.439 | 0.0180 | 0.436 | 0.0187 | | 100 | 0.438 | 0.0183 | 0.439 | 0.0180 | 0.436 | 0.0187 |
| OvClustCD | 10 | 0.348 | 0.0185 | 0.353 | 0.0184 | 0.346 | 0.0184 | Avg_GRM_MinMax | 10 | 0.337 | 0.0242 | 0.347 | 0.0235 | 0.340 | 0.0241 |
| | 20 | 0.376 | 0.0182 | 0.384 | 0.0173 | 0.373 | 0.0185 | | 20 | 0.365 | 0.0208 | 0.370 | 0.0206 | 0.368 | 0.0203 |
| | 40 | 0.401 | 0.0183 | 0.405 | 0.0176 | 0.399 | 0.0179 | | 40 | 0.401 | 0.0209 | 0.408 | 0.0205 | 0.402 | 0.0205 |
| | 60 | 0.423 | 0.0169 | 0.423 | 0.0168 | 0.416 | 0.0175 | | 60 | 0.431 | 0.0191 | 0.433 | 0.0187 | 0.432 | 0.0193 |
| | 80 | 0.438 | 0.0164 | 0.438 | 0.0161 | 0.434 | 0.0170 | | 80 | 0.441 | 0.0190 | 0.440 | 0.0192 | 0.443 | 0.0191 |
| | 100 | 0.438 | 0.0183 | 0.439 | 0.0180 | 0.436 | 0.0187 | | 100 | 0.438 | 0.0183 | 0.439 | 0.0180 | 0.436 | 0.0187 |
| OvClustCDtarg | 10 | 0.372 | 0.0196 | 0.373 | 0.0190 | 0.366 | 0.0191 | Avg_GRM_MinMaxtarg | 10 | 0.351 | 0.0230 | 0.359 | 0.0219 | 0.352 | 0.0226 |
| | 20 | 0.403 | 0.0191 | 0.401 | 0.0191 | 0.400 | 0.0192 | | 20 | 0.390 | 0.0215 | 0.400 | 0.0204 | 0.387 | 0.0207 |
| | 40 | 0.434 | 0.0177 | 0.434 | 0.0176 | 0.432 | 0.0175 | | 40 | 0.422 | 0.0190 | 0.425 | 0.0186 | 0.423 | 0.0190 |
| | 60 | 0.442 | 0.0167 | 0.441 | 0.0167 | 0.441 | 0.0166 | | 60 | 0.441 | 0.0190 | 0.444 | 0.0183 | 0.444 | 0.0185 |
| | 80 | 0.453 | 0.0169 | 0.451 | 0.0171 | 0.451 | 0.0167 | | 80 | 0.443 | 0.0191 | 0.443 | 0.0188 | 0.444 | 0.0188 |
| | 100 | 0.438 | 0.0183 | 0.439 | 0.0180 | 0.436 | 0.0187 | | 100 | 0.438 | 0.0183 | 0.439 | 0.0180 | 0.436 | 0.0187 |
| WIClustCD | 10 | 0.349 | 0.0232 | 0.362 | 0.0220 | 0.352 | 0.0226 | Avg_GRM_self | 10 | 0.356 | 0.0222 | 0.365 | 0.0203 | 0.355 | 0.0219 |
| | 20 | 0.387 | 0.0195 | 0.390 | 0.0197 | 0.387 | 0.0190 | | 20 | 0.360 | 0.0225 | 0.364 | 0.0218 | 0.361 | 0.0219 |
| | 40 | 0.395 | 0.0197 | 0.398 | 0.0194 | 0.395 | 0.0197 | | 40 | 0.407 | 0.0208 | 0.414 | 0.0200 | 0.411 | 0.0204 |
| | 60 | 0.411 | 0.0204 | 0.416 | 0.0199 | 0.412 | 0.0199 | | 60 | 0.435 | 0.0181 | 0.435 | 0.0182 | 0.438 | 0.0180 |
| | 80 | 0.432 | 0.0198 | 0.433 | 0.0199 | 0.431 | 0.0197 | | 80 | 0.443 | 0.0193 | 0.442 | 0.0192 | 0.444 | 0.0191 |
| | 100 | 0.438 | 0.0183 | 0.439 | 0.0180 | 0.436 | 0.0187 | | 100 | 0.438 | 0.0183 | 0.439 | 0.0180 | 0.436 | 0.0187 |
| WIClustCDtarg | 10 | 0.375 | 0.0208 | 0.378 | 0.0210 | 0.380 | 0.0203 | PAM | 10 | 0.357 | 0.0229 | 0.357 | 0.0235 | 0.353 | 0.0230 |
| | 20 | 0.400 | 0.0192 | 0.407 | 0.0184 | 0.404 | 0.0191 | | 20 | 0.392 | 0.0200 | 0.393 | 0.0194 | 0.386 | 0.0201 |
| | 40 | 0.431 | 0.0191 | 0.434 | 0.0183 | 0.431 | 0.0189 | | 40 | 0.425 | 0.0179 | 0.424 | 0.0180 | 0.421 | 0.0178 |
| | 60 | 0.440 | 0.0190 | 0.441 | 0.0182 | 0.439 | 0.0187 | | 60 | 0.442 | 0.0190 | 0.443 | 0.0186 | 0.438 | 0.0185 |
| | 80 | 0.447 | 0.0187 | 0.448 | 0.0183 | 0.448 | 0.0186 | | 80 | 0.465 | 0.0175 | 0.462 | 0.0169 | 0.461 | 0.0172 |
| | 100 | 0.438 | 0.0183 | 0.439 | 0.0180 | 0.436 | 0.0187 | | 100 | 0.438 | 0.0183 | 0.439 | 0.0180 | 0.436 | 0.0187 |

**Table S20** Average accuracy and its corresponding standard error of the mean (SEM) across the 40 iterations for all training set optimization methods, models and training set (TRS) sizes (expressed as percentage of the candidate set (CS)) for soybean dataset and HT trait. If "targ" is added at the end of the name of a method, it corresponds to targeted optimization. Otherwise, untargeted optimization was performed.

**Soy trait HT**

| Optimization method | TRS size (% of CS) | Average accuracy | | | | | | Optimization method | TRS size (% of CS) | Average accuracy | | | | | |
|---|---|---|---|---|---|---|---|---|---|---|---|---|---|---|---|
| | | GBLUP | | BayesB | | RKHS | | | | GBLUP | | BayesB | | RKHS | |
| | | Mean | SEM | Mean | SEM | Mean | SEM | | | Mean | SEM | Mean | SEM | Mean | SEM |
| RAND | 10 | 0.123 | 0.00681 | 0.142 | 0.00635 | 0.135 | 0.00689 | Rscore | 10 | 0.128 | 0.00871 | 0.145 | 0.00953 | 0.154 | 0.00883 |
| | 20 | 0.186 | 0.00599 | 0.206 | 0.00565 | 0.202 | 0.00578 | | 20 | 0.190 | 0.00888 | 0.207 | 0.00858 | 0.219 | 0.00871 |
| | 40 | 0.270 | 0.00704 | 0.278 | 0.00657 | 0.278 | 0.00695 | | 40 | 0.267 | 0.00611 | 0.278 | 0.00577 | 0.280 | 0.00545 |
| | 60 | 0.334 | 0.00583 | 0.340 | 0.00578 | 0.338 | 0.00601 | | 60 | 0.325 | 0.00541 | 0.331 | 0.00554 | 0.325 | 0.00592 |
| | 80 | 0.364 | 0.00521 | 0.366 | 0.00489 | 0.366 | 0.00558 | | 80 | 0.363 | 0.00451 | 0.365 | 0.00451 | 0.354 | 0.00474 |
| | 100 | 0.392 | 0.00360 | 0.393 | 0.00352 | 0.388 | 0.00398 | | 100 | 0.392 | 0.00360 | 0.393 | 0.00352 | 0.388 | 0.00398 |
| StratSamp | 10 | 0.117 | 0.00654 | 0.129 | 0.00723 | 0.136 | 0.00711 | Rscoretarg | 10 | 0.148 | 0.00763 | 0.160 | 0.00784 | 0.166 | 0.00815 |
| | 20 | 0.189 | 0.00711 | 0.208 | 0.00683 | 0.213 | 0.00746 | | 20 | 0.201 | 0.00807 | 0.213 | 0.00782 | 0.220 | 0.00802 |
| | 40 | 0.288 | 0.00540 | 0.298 | 0.00547 | 0.302 | 0.00536 | | 40 | 0.290 | 0.00558 | 0.296 | 0.00548 | 0.294 | 0.00548 |
| | 60 | 0.336 | 0.00447 | 0.341 | 0.00458 | 0.333 | 0.00479 | | 60 | 0.335 | 0.00552 | 0.340 | 0.00507 | 0.328 | 0.00568 |
| | 80 | 0.368 | 0.00415 | 0.370 | 0.00393 | 0.363 | 0.00473 | | 80 | 0.369 | 0.00449 | 0.371 | 0.00450 | 0.357 | 0.00576 |
| | 100 | 0.392 | 0.00360 | 0.393 | 0.00352 | 0.388 | 0.00398 | | 100 | 0.392 | 0.00360 | 0.393 | 0.00352 | 0.388 | 0.00398 |
| CD | 10 | 0.123 | 0.00745 | 0.143 | 0.00799 | 0.144 | 0.00749 | Avg_GRM | 10 | 0.116 | 0.00741 | 0.120 | 0.00771 | 0.125 | 0.00753 |
| | 20 | 0.186 | 0.00623 | 0.209 | 0.00670 | 0.216 | 0.00668 | | 20 | 0.164 | 0.00608 | 0.174 | 0.00612 | 0.180 | 0.00625 |
| | 40 | 0.281 | 0.00738 | 0.292 | 0.00729 | 0.291 | 0.00746 | | 40 | 0.264 | 0.00629 | 0.274 | 0.00629 | 0.283 | 0.00615 |
| | 60 | 0.328 | 0.00545 | 0.335 | 0.00533 | 0.322 | 0.00565 | | 60 | 0.334 | 0.00597 | 0.338 | 0.00576 | 0.345 | 0.00605 |
| | 80 | 0.366 | 0.00465 | 0.369 | 0.00443 | 0.356 | 0.00562 | | 80 | 0.368 | 0.00496 | 0.371 | 0.00495 | 0.368 | 0.00496 |
| | 100 | 0.392 | 0.00360 | 0.393 | 0.00352 | 0.388 | 0.00398 | | 100 | 0.392 | 0.00360 | 0.393 | 0.00352 | 0.388 | 0.00398 |
| CDtarg | 10 | 0.125 | 0.00738 | 0.142 | 0.00815 | 0.142 | 0.00778 | Avg_GRMtarg | 10 | 0.122 | 0.00776 | 0.134 | 0.00741 | 0.134 | 0.00820 |
| | 20 | 0.194 | 0.00698 | 0.214 | 0.00717 | 0.221 | 0.00703 | | 20 | 0.189 | 0.00745 | 0.203 | 0.00718 | 0.207 | 0.00783 |
| | 40 | 0.290 | 0.00585 | 0.301 | 0.00531 | 0.300 | 0.00629 | | 40 | 0.278 | 0.00587 | 0.289 | 0.00559 | 0.288 | 0.00588 |
| | 60 | 0.341 | 0.00400 | 0.346 | 0.00381 | 0.344 | 0.00501 | | 60 | 0.333 | 0.00507 | 0.339 | 0.00494 | 0.334 | 0.00548 |
| | 80 | 0.372 | 0.00381 | 0.374 | 0.00375 | 0.364 | 0.00513 | | 80 | 0.370 | 0.00404 | 0.373 | 0.00393 | 0.368 | 0.00405 |
| | 100 | 0.392 | 0.00360 | 0.393 | 0.00352 | 0.388 | 0.00398 | | 100 | 0.392 | 0.00360 | 0.393 | 0.00352 | 0.388 | 0.00398 |
| OvClustCD | 10 | 0.124 | 0.00629 | 0.142 | 0.00659 | 0.148 | 0.00680 | Avg_GRM_MinMax | 10 | 0.123 | 0.00933 | 0.142 | 0.00930 | 0.143 | 0.00888 |
| | 20 | 0.196 | 0.00717 | 0.218 | 0.00770 | 0.227 | 0.00715 | | 20 | 0.189 | 0.00850 | 0.208 | 0.00860 | 0.211 | 0.00794 |
| | 40 | 0.273 | 0.00652 | 0.283 | 0.00631 | 0.282 | 0.00612 | | 40 | 0.274 | 0.00594 | 0.288 | 0.00519 | 0.281 | 0.00581 |
| | 60 | 0.325 | 0.00497 | 0.330 | 0.00508 | 0.324 | 0.00597 | | 60 | 0.337 | 0.00526 | 0.343 | 0.00511 | 0.337 | 0.00578 |
| | 80 | 0.365 | 0.00487 | 0.368 | 0.00476 | 0.356 | 0.00587 | | 80 | 0.373 | 0.00430 | 0.375 | 0.00418 | 0.369 | 0.00508 |
| | 100 | 0.392 | 0.00360 | 0.393 | 0.00352 | 0.388 | 0.00398 | | 100 | 0.392 | 0.00360 | 0.393 | 0.00352 | 0.388 | 0.00398 |
| OvClustCDtarg | 10 | 0.139 | 0.00586 | 0.155 | 0.00594 | 0.164 | 0.00583 | Avg_GRM_MinMaxtarg | 10 | 0.118 | 0.00793 | 0.139 | 0.00796 | 0.132 | 0.00788 |
| | 20 | 0.191 | 0.00820 | 0.209 | 0.00778 | 0.217 | 0.00813 | | 20 | 0.179 | 0.00701 | 0.200 | 0.00633 | 0.203 | 0.00683 |
| | 40 | 0.285 | 0.00605 | 0.293 | 0.00561 | 0.290 | 0.00608 | | 40 | 0.275 | 0.00605 | 0.285 | 0.00592 | 0.284 | 0.00554 |
| | 60 | 0.346 | 0.00500 | 0.350 | 0.00484 | 0.340 | 0.00614 | | 60 | 0.341 | 0.00457 | 0.347 | 0.00466 | 0.337 | 0.00488 |
| | 80 | 0.374 | 0.00329 | 0.376 | 0.00325 | 0.365 | 0.00491 | | 80 | 0.374 | 0.00308 | 0.377 | 0.00321 | 0.367 | 0.00390 |
| | 100 | 0.392 | 0.00360 | 0.393 | 0.00352 | 0.388 | 0.00398 | | 100 | 0.392 | 0.00360 | 0.393 | 0.00352 | 0.388 | 0.00398 |
| WIClustCD | 10 | 0.142 | 0.00674 | 0.160 | 0.00743 | 0.163 | 0.00716 | Avg_GRM_self | 10 | 0.122 | 0.00864 | 0.140 | 0.00856 | 0.136 | 0.00915 |
| | 20 | 0.190 | 0.00882 | 0.209 | 0.00829 | 0.218 | 0.00894 | | 20 | 0.185 | 0.00746 | 0.205 | 0.00701 | 0.209 | 0.00725 |
| | 40 | 0.277 | 0.00597 | 0.288 | 0.00544 | 0.292 | 0.00587 | | 40 | 0.288 | 0.00519 | 0.298 | 0.00519 | 0.296 | 0.00596 |
| | 60 | 0.326 | 0.00562 | 0.330 | 0.00549 | 0.323 | 0.00553 | | 60 | 0.338 | 0.00504 | 0.343 | 0.00473 | 0.329 | 0.00558 |
| | 80 | 0.366 | 0.00401 | 0.368 | 0.00406 | 0.351 | 0.00501 | | 80 | 0.375 | 0.00364 | 0.378 | 0.00349 | 0.368 | 0.00371 |
| | 100 | 0.392 | 0.00360 | 0.393 | 0.00352 | 0.388 | 0.00398 | | 100 | 0.392 | 0.00360 | 0.393 | 0.00352 | 0.388 | 0.00398 |
| WIClustCDtarg | 10 | 0.124 | 0.00769 | 0.144 | 0.00791 | 0.141 | 0.00732 | PAM | 10 | 0.124 | 0.00639 | 0.139 | 0.00691 | 0.146 | 0.00685 |
| | 20 | 0.212 | 0.00600 | 0.231 | 0.00597 | 0.234 | 0.00643 | | 20 | 0.194 | 0.00718 | 0.213 | 0.00687 | 0.216 | 0.00726 |
| | 40 | 0.291 | 0.00494 | 0.298 | 0.00481 | 0.298 | 0.00515 | | 40 | 0.284 | 0.00511 | 0.292 | 0.00550 | 0.293 | 0.00510 |
| | 60 | 0.347 | 0.00418 | 0.352 | 0.00418 | 0.342 | 0.00534 | | 60 | 0.340 | 0.00448 | 0.344 | 0.00438 | 0.335 | 0.00540 |
| | 80 | 0.374 | 0.00395 | 0.375 | 0.00395 | 0.362 | 0.00479 | | 80 | 0.371 | 0.00425 | 0.373 | 0.00428 | 0.363 | 0.00501 |
| | 100 | 0.392 | 0.00360 | 0.393 | 0.00352 | 0.388 | 0.00398 | | 100 | 0.392 | 0.00360 | 0.393 | 0.00352 | 0.388 | 0.00398 |

**Table S21** Average accuracy and its corresponding standard error of the mean (SEM) across the 40 iterations for all training set optimization methods, models and training set (TRS) sizes (expressed as percentage of the candidate set (CS)) for soybean dataset and R8 trait. If "targ" is added at the end of the name of a method, it corresponds to targeted optimization. Otherwise, untargeted optimization was performed.

**Soy trait R8**

| Optimization method | TRS size (% of CS) | Average accuracy | | | | | | Optimization method | TRS size (% of CS) | Average accuracy | | | | | |
|---|---|---|---|---|---|---|---|---|---|---|---|---|---|---|---|
| | | GBLUP | | BayesB | | RKHS | | | | GBLUP | | BayesB | | RKHS | |
| | | Mean | SEM | Mean | SEM | Mean | SEM | | | Mean | SEM | Mean | SEM | Mean | SEM |
| RAND | 10 | 0.128 | 0.00856 | 0.142 | 0.00859 | 0.140 | 0.00948 | Rscore | 10 | 0.154 | 0.00723 | 0.169 | 0.00754 | 0.175 | 0.00820 |
| | 20 | 0.209 | 0.00636 | 0.223 | 0.00647 | 0.233 | 0.00684 | | 20 | 0.206 | 0.00720 | 0.216 | 0.00717 | 0.235 | 0.00767 |
| | 40 | 0.294 | 0.00720 | 0.303 | 0.00702 | 0.307 | 0.00627 | | 40 | 0.286 | 0.00746 | 0.292 | 0.00733 | 0.303 | 0.00671 |
| | 60 | 0.342 | 0.00566 | 0.349 | 0.00548 | 0.350 | 0.00553 | | 60 | 0.342 | 0.00517 | 0.346 | 0.00505 | 0.352 | 0.00571 |
| | 80 | 0.381 | 0.00569 | 0.385 | 0.00551 | 0.383 | 0.00537 | | 80 | 0.377 | 0.00537 | 0.380 | 0.00533 | 0.375 | 0.00601 |
| | 100 | 0.401 | 0.00532 | 0.402 | 0.00527 | 0.401 | 0.00581 | | 100 | 0.401 | 0.00532 | 0.402 | 0.00527 | 0.401 | 0.00581 |
| StratSamp | 10 | 0.140 | 0.00900 | 0.149 | 0.00887 | 0.161 | 0.00949 | Rscoretarg | 10 | 0.151 | 0.00852 | 0.164 | 0.00866 | 0.176 | 0.00864 |
| | 20 | 0.202 | 0.00730 | 0.216 | 0.00769 | 0.230 | 0.00774 | | 20 | 0.220 | 0.00745 | 0.229 | 0.00755 | 0.243 | 0.00778 |
| | 40 | 0.302 | 0.00631 | 0.308 | 0.00621 | 0.322 | 0.00616 | | 40 | 0.295 | 0.00606 | 0.301 | 0.00580 | 0.310 | 0.00616 |
| | 60 | 0.345 | 0.00530 | 0.350 | 0.00549 | 0.352 | 0.00554 | | 60 | 0.347 | 0.00586 | 0.351 | 0.00589 | 0.350 | 0.00656 |
| | 80 | 0.375 | 0.00488 | 0.378 | 0.00486 | 0.380 | 0.00548 | | 80 | 0.381 | 0.00555 | 0.384 | 0.00544 | 0.378 | 0.00627 |
| | 100 | 0.401 | 0.00532 | 0.402 | 0.00527 | 0.401 | 0.00581 | | 100 | 0.401 | 0.00532 | 0.402 | 0.00527 | 0.401 | 0.00581 |
| CD | 10 | 0.139 | 0.00705 | 0.154 | 0.00691 | 0.163 | 0.00732 | Avg_GRM | 10 | 0.103 | 0.00789 | 0.106 | 0.00823 | 0.114 | 0.00797 |
| | 20 | 0.204 | 0.00682 | 0.219 | 0.00680 | 0.237 | 0.00720 | | 20 | 0.154 | 0.00685 | 0.160 | 0.00706 | 0.172 | 0.00711 |
| | 40 | 0.302 | 0.00626 | 0.308 | 0.00620 | 0.321 | 0.00697 | | 40 | 0.266 | 0.00564 | 0.273 | 0.00540 | 0.287 | 0.00537 |
| | 60 | 0.345 | 0.00605 | 0.351 | 0.00594 | 0.356 | 0.00660 | | 60 | 0.335 | 0.00687 | 0.339 | 0.00679 | 0.349 | 0.00710 |
| | 80 | 0.378 | 0.00560 | 0.381 | 0.00559 | 0.377 | 0.00626 | | 80 | 0.375 | 0.00547 | 0.377 | 0.00541 | 0.381 | 0.00575 |
| | 100 | 0.401 | 0.00532 | 0.402 | 0.00527 | 0.401 | 0.00581 | | 100 | 0.401 | 0.00532 | 0.402 | 0.00527 | 0.401 | 0.00581 |
| CDtarg | 10 | 0.135 | 0.00891 | 0.154 | 0.00955 | 0.163 | 0.00939 | Avg_GRMtarg | 10 | 0.148 | 0.00833 | 0.155 | 0.00823 | 0.169 | 0.00801 |
| | 20 | 0.217 | 0.00670 | 0.232 | 0.00609 | 0.246 | 0.00662 | | 20 | 0.208 | 0.00760 | 0.218 | 0.00737 | 0.234 | 0.00720 |
| | 40 | 0.308 | 0.00589 | 0.312 | 0.00592 | 0.324 | 0.00706 | | 40 | 0.292 | 0.00650 | 0.299 | 0.00657 | 0.312 | 0.00692 |
| | 60 | 0.353 | 0.00655 | 0.358 | 0.00667 | 0.363 | 0.00654 | | 60 | 0.339 | 0.00612 | 0.344 | 0.00610 | 0.350 | 0.00626 |
| | 80 | 0.380 | 0.00549 | 0.383 | 0.00538 | 0.383 | 0.00635 | | 80 | 0.378 | 0.00523 | 0.381 | 0.00519 | 0.381 | 0.00584 |
| | 100 | 0.401 | 0.00532 | 0.402 | 0.00527 | 0.401 | 0.00581 | | 100 | 0.401 | 0.00532 | 0.402 | 0.00527 | 0.401 | 0.00581 |
| OvClustCD | 10 | 0.152 | 0.00769 | 0.168 | 0.00725 | 0.181 | 0.00760 | Avg_GRM_MinMax | 10 | 0.144 | 0.00854 | 0.159 | 0.00779 | 0.168 | 0.00785 |
| | 20 | 0.227 | 0.00668 | 0.241 | 0.00622 | 0.255 | 0.00628 | | 20 | 0.210 | 0.00670 | 0.223 | 0.00647 | 0.241 | 0.00568 |
| | 40 | 0.301 | 0.00681 | 0.308 | 0.00704 | 0.323 | 0.00645 | | 40 | 0.288 | 0.00658 | 0.296 | 0.00657 | 0.312 | 0.00671 |
| | 60 | 0.344 | 0.00575 | 0.348 | 0.00570 | 0.353 | 0.00669 | | 60 | 0.350 | 0.00601 | 0.354 | 0.00613 | 0.356 | 0.00690 |
| | 80 | 0.376 | 0.00536 | 0.380 | 0.00543 | 0.377 | 0.00631 | | 80 | 0.382 | 0.00591 | 0.384 | 0.00579 | 0.381 | 0.00663 |
| | 100 | 0.401 | 0.00532 | 0.402 | 0.00527 | 0.401 | 0.00581 | | 100 | 0.401 | 0.00532 | 0.402 | 0.00527 | 0.401 | 0.00581 |
| OvClustCDtarg | 10 | 0.140 | 0.00828 | 0.157 | 0.00821 | 0.173 | 0.00802 | Avg_GRM_MinMaxtarg | 10 | 0.144 | 0.00909 | 0.156 | 0.00947 | 0.164 | 0.00906 |
| | 20 | 0.217 | 0.00706 | 0.231 | 0.00634 | 0.247 | 0.00790 | | 20 | 0.207 | 0.00764 | 0.225 | 0.00786 | 0.233 | 0.00804 |
| | 40 | 0.304 | 0.00615 | 0.309 | 0.00604 | 0.318 | 0.00699 | | 40 | 0.295 | 0.00664 | 0.303 | 0.00649 | 0.313 | 0.00637 |
| | 60 | 0.354 | 0.00560 | 0.358 | 0.00560 | 0.360 | 0.00640 | | 60 | 0.355 | 0.00583 | 0.360 | 0.00591 | 0.366 | 0.00613 |
| | 80 | 0.380 | 0.00525 | 0.383 | 0.00526 | 0.381 | 0.00611 | | 80 | 0.385 | 0.00513 | 0.389 | 0.00519 | 0.385 | 0.00568 |
| | 100 | 0.401 | 0.00532 | 0.402 | 0.00527 | 0.401 | 0.00581 | | 100 | 0.401 | 0.00532 | 0.402 | 0.00527 | 0.401 | 0.00581 |
| WIClustCD | 10 | 0.159 | 0.00882 | 0.177 | 0.00859 | 0.185 | 0.00913 | Avg_GRM_self | 10 | 0.142 | 0.00794 | 0.159 | 0.00812 | 0.159 | 0.00784 |
| | 20 | 0.218 | 0.00619 | 0.235 | 0.00605 | 0.251 | 0.00602 | | 20 | 0.220 | 0.00797 | 0.236 | 0.00752 | 0.249 | 0.00789 |
| | 40 | 0.292 | 0.00551 | 0.300 | 0.00526 | 0.314 | 0.00598 | | 40 | 0.306 | 0.00588 | 0.315 | 0.00585 | 0.323 | 0.00590 |
| | 60 | 0.343 | 0.00557 | 0.347 | 0.00556 | 0.355 | 0.00625 | | 60 | 0.348 | 0.00653 | 0.352 | 0.00651 | 0.352 | 0.00693 |
| | 80 | 0.377 | 0.00514 | 0.380 | 0.00508 | 0.375 | 0.00571 | | 80 | 0.384 | 0.00571 | 0.386 | 0.00577 | 0.385 | 0.00606 |
| | 100 | 0.401 | 0.00532 | 0.402 | 0.00527 | 0.401 | 0.00581 | | 100 | 0.401 | 0.00532 | 0.402 | 0.00527 | 0.401 | 0.00581 |
| WIClustCDtarg | 10 | 0.151 | 0.00833 | 0.170 | 0.00861 | 0.172 | 0.00848 | PAM | 10 | 0.139 | 0.00620 | 0.152 | 0.00571 | 0.166 | 0.00603 |
| | 20 | 0.230 | 0.00654 | 0.242 | 0.00576 | 0.259 | 0.00677 | | 20 | 0.215 | 0.00671 | 0.229 | 0.00664 | 0.242 | 0.00682 |
| | 40 | 0.309 | 0.00640 | 0.316 | 0.00637 | 0.324 | 0.00703 | | 40 | 0.292 | 0.00652 | 0.299 | 0.00649 | 0.312 | 0.00678 |
| | 60 | 0.357 | 0.00532 | 0.361 | 0.00533 | 0.364 | 0.00603 | | 60 | 0.346 | 0.00585 | 0.350 | 0.00591 | 0.351 | 0.00654 |
| | 80 | 0.382 | 0.00517 | 0.384 | 0.00500 | 0.378 | 0.00594 | | 80 | 0.381 | 0.00545 | 0.384 | 0.00549 | 0.380 | 0.00624 |
| | 100 | 0.401 | 0.00532 | 0.402 | 0.00527 | 0.401 | 0.00581 | | 100 | 0.401 | 0.00532 | 0.402 | 0.00527 | 0.401 | 0.00581 |

**Table S22** Average accuracy and its corresponding standard error of the mean (SEM) across the 40 iterations for all training set optimization methods, models and training set (TRS) sizes (expressed as percentage of the candidate set (CS)) for soybean dataset and YLD trait. If "targ" is added at the end of the name of a method, it corresponds to targeted optimization. Otherwise, untargeted optimization was performed.

**Soy trait YLD**

| Optimization method | TRS size (% of CS) | Average accuracy | | | | | | Optimization method | TRS size (% of CS) | Average accuracy | | | | | |
|---|---|---|---|---|---|---|---|---|---|---|---|---|---|---|---|
| | | GBLUP | | BayesB | | RKHS | | | | GBLUP | | BayesB | | RKHS | |
| | | Mean | SEM | Mean | SEM | Mean | SEM | | | Mean | SEM | Mean | SEM | Mean | SEM |
| RAND | 10 | 0.347 | 0.00789 | 0.353 | 0.00745 | 0.348 | 0.00780 | Rscore | 10 | 0.364 | 0.00820 | 0.368 | 0.00805 | 0.368 | 0.00806 |
| | 20 | 0.450 | 0.00558 | 0.454 | 0.00574 | 0.452 | 0.00553 | | 20 | 0.463 | 0.00515 | 0.467 | 0.00496 | 0.466 | 0.00538 |
| | 40 | 0.539 | 0.00539 | 0.542 | 0.00542 | 0.539 | 0.00553 | | 40 | 0.539 | 0.00434 | 0.542 | 0.00427 | 0.538 | 0.00424 |
| | 60 | 0.566 | 0.00436 | 0.569 | 0.00430 | 0.567 | 0.00455 | | 60 | 0.574 | 0.00406 | 0.577 | 0.00412 | 0.575 | 0.00420 |
| | 80 | 0.591 | 0.00386 | 0.592 | 0.00380 | 0.592 | 0.00381 | | 80 | 0.591 | 0.00361 | 0.593 | 0.00355 | 0.593 | 0.00375 |
| | 100 | 0.605 | 0.00339 | 0.605 | 0.00343 | 0.606 | 0.00346 | | 100 | 0.605 | 0.00339 | 0.605 | 0.00343 | 0.606 | 0.00346 |
| StratSamp | 10 | 0.358 | 0.00927 | 0.364 | 0.00900 | 0.364 | 0.00888 | Rscoretarg | 10 | 0.354 | 0.00849 | 0.362 | 0.00832 | 0.358 | 0.00835 |
| | 20 | 0.468 | 0.00536 | 0.471 | 0.00516 | 0.471 | 0.00543 | | 20 | 0.452 | 0.00664 | 0.457 | 0.00671 | 0.455 | 0.00694 |
| | 40 | 0.542 | 0.00475 | 0.545 | 0.00485 | 0.543 | 0.00473 | | 40 | 0.535 | 0.00498 | 0.539 | 0.00497 | 0.535 | 0.00525 |
| | 60 | 0.574 | 0.00411 | 0.576 | 0.00403 | 0.575 | 0.00415 | | 60 | 0.569 | 0.00377 | 0.571 | 0.00363 | 0.569 | 0.00371 |
| | 80 | 0.594 | 0.00348 | 0.596 | 0.00333 | 0.595 | 0.00365 | | 80 | 0.592 | 0.00384 | 0.593 | 0.00373 | 0.593 | 0.00394 |
| | 100 | 0.605 | 0.00339 | 0.605 | 0.00343 | 0.606 | 0.00346 | | 100 | 0.605 | 0.00339 | 0.605 | 0.00343 | 0.606 | 0.00346 |
| CD | 10 | 0.382 | 0.00691 | 0.385 | 0.00699 | 0.386 | 0.00689 | Avg_GRM | 10 | 0.378 | 0.00769 | 0.381 | 0.00716 | 0.386 | 0.00784 |
| | 20 | 0.474 | 0.00603 | 0.478 | 0.00589 | 0.478 | 0.00602 | | 20 | 0.449 | 0.00681 | 0.452 | 0.00698 | 0.451 | 0.00680 |
| | 40 | 0.545 | 0.00491 | 0.549 | 0.00489 | 0.545 | 0.00503 | | 40 | 0.536 | 0.00473 | 0.540 | 0.00467 | 0.536 | 0.00474 |
| | 60 | 0.575 | 0.00366 | 0.578 | 0.00350 | 0.577 | 0.00387 | | 60 | 0.573 | 0.00372 | 0.575 | 0.00375 | 0.574 | 0.00389 |
| | 80 | 0.594 | 0.00314 | 0.596 | 0.00310 | 0.595 | 0.00349 | | 80 | 0.591 | 0.00345 | 0.592 | 0.00339 | 0.593 | 0.00346 |
| | 100 | 0.605 | 0.00339 | 0.605 | 0.00343 | 0.606 | 0.00346 | | 100 | 0.605 | 0.00339 | 0.605 | 0.00343 | 0.606 | 0.00346 |
| CDtarg | 10 | 0.388 | 0.00875 | 0.393 | 0.00839 | 0.395 | 0.00847 | Avg_GRMtarg | 10 | 0.350 | 0.00836 | 0.354 | 0.00813 | 0.355 | 0.00823 |
| | 20 | 0.480 | 0.00470 | 0.483 | 0.00469 | 0.484 | 0.00466 | | 20 | 0.446 | 0.00764 | 0.450 | 0.00766 | 0.446 | 0.00744 |
| | 40 | 0.547 | 0.00461 | 0.550 | 0.00464 | 0.548 | 0.00438 | | 40 | 0.534 | 0.00489 | 0.536 | 0.00466 | 0.533 | 0.00489 |
| | 60 | 0.579 | 0.00398 | 0.581 | 0.00399 | 0.580 | 0.00392 | | 60 | 0.572 | 0.00426 | 0.574 | 0.00414 | 0.573 | 0.00439 |
| | 80 | 0.596 | 0.00354 | 0.597 | 0.00348 | 0.597 | 0.00360 | | 80 | 0.592 | 0.00376 | 0.594 | 0.00369 | 0.594 | 0.00384 |
| | 100 | 0.605 | 0.00339 | 0.605 | 0.00343 | 0.606 | 0.00346 | | 100 | 0.605 | 0.00339 | 0.605 | 0.00343 | 0.606 | 0.00346 |
| OvClustCD | 10 | 0.398 | 0.00568 | 0.403 | 0.00567 | 0.406 | 0.00546 | Avg_GRM_MinMax | 10 | 0.367 | 0.00702 | 0.373 | 0.00700 | 0.372 | 0.00697 |
| | 20 | 0.473 | 0.00562 | 0.477 | 0.00554 | 0.477 | 0.00560 | | 20 | 0.462 | 0.00522 | 0.466 | 0.00511 | 0.465 | 0.00505 |
| | 40 | 0.547 | 0.00394 | 0.551 | 0.00391 | 0.549 | 0.00420 | | 40 | 0.535 | 0.00512 | 0.538 | 0.00517 | 0.535 | 0.00523 |
| | 60 | 0.576 | 0.00371 | 0.578 | 0.00361 | 0.578 | 0.00370 | | 60 | 0.575 | 0.00379 | 0.576 | 0.00368 | 0.574 | 0.00422 |
| | 80 | 0.592 | 0.00326 | 0.593 | 0.00313 | 0.593 | 0.00314 | | 80 | 0.594 | 0.00351 | 0.595 | 0.00350 | 0.595 | 0.00371 |
| | 100 | 0.605 | 0.00339 | 0.605 | 0.00343 | 0.606 | 0.00346 | | 100 | 0.605 | 0.00339 | 0.605 | 0.00343 | 0.606 | 0.00346 |
| OvClustCDtarg | 10 | 0.392 | 0.00684 | 0.395 | 0.00691 | 0.399 | 0.00692 | Avg_GRM_MinMaxtarg | 10 | 0.362 | 0.00951 | 0.372 | 0.00835 | 0.367 | 0.00935 |
| | 20 | 0.481 | 0.00545 | 0.484 | 0.00550 | 0.483 | 0.00564 | | 20 | 0.461 | 0.00637 | 0.466 | 0.00633 | 0.462 | 0.00639 |
| | 40 | 0.546 | 0.00386 | 0.549 | 0.00375 | 0.548 | 0.00396 | | 40 | 0.540 | 0.00478 | 0.543 | 0.00466 | 0.539 | 0.00522 |
| | 60 | 0.576 | 0.00370 | 0.578 | 0.00363 | 0.577 | 0.00369 | | 60 | 0.578 | 0.00405 | 0.580 | 0.00393 | 0.580 | 0.00404 |
| | 80 | 0.594 | 0.00357 | 0.595 | 0.00355 | 0.595 | 0.00378 | | 80 | 0.595 | 0.00324 | 0.597 | 0.00316 | 0.596 | 0.00329 |
| | 100 | 0.605 | 0.00339 | 0.605 | 0.00343 | 0.606 | 0.00346 | | 100 | 0.605 | 0.00339 | 0.605 | 0.00343 | 0.606 | 0.00346 |
| WIClustCD | 10 | 0.378 | 0.00866 | 0.383 | 0.00875 | 0.382 | 0.00874 | Avg_GRM_self | 10 | 0.366 | 0.00687 | 0.376 | 0.00710 | 0.372 | 0.00707 |
| | 20 | 0.473 | 0.00619 | 0.478 | 0.00616 | 0.477 | 0.00615 | | 20 | 0.462 | 0.00637 | 0.467 | 0.00657 | 0.465 | 0.00633 |
| | 40 | 0.547 | 0.00438 | 0.551 | 0.00437 | 0.549 | 0.00470 | | 40 | 0.544 | 0.00406 | 0.548 | 0.00409 | 0.544 | 0.00420 |
| | 60 | 0.575 | 0.00372 | 0.577 | 0.00354 | 0.576 | 0.00396 | | 60 | 0.575 | 0.00368 | 0.577 | 0.00352 | 0.576 | 0.00388 |
| | 80 | 0.593 | 0.00365 | 0.594 | 0.00358 | 0.595 | 0.00369 | | 80 | 0.593 | 0.00351 | 0.595 | 0.00354 | 0.595 | 0.00364 |
| | 100 | 0.605 | 0.00339 | 0.605 | 0.00343 | 0.606 | 0.00346 | | 100 | 0.605 | 0.00339 | 0.605 | 0.00343 | 0.606 | 0.00346 |
| WIClustCDtarg | 10 | 0.392 | 0.00837 | 0.395 | 0.00795 | 0.400 | 0.00801 | PAM | 10 | 0.361 | 0.00683 | 0.365 | 0.00672 | 0.366 | 0.00696 |
| | 20 | 0.480 | 0.00538 | 0.485 | 0.00537 | 0.485 | 0.00529 | | 20 | 0.463 | 0.00653 | 0.466 | 0.00675 | 0.465 | 0.00669 |
| | 40 | 0.554 | 0.00414 | 0.558 | 0.00419 | 0.555 | 0.00385 | | 40 | 0.543 | 0.00472 | 0.547 | 0.00465 | 0.545 | 0.00468 |
| | 60 | 0.579 | 0.00400 | 0.580 | 0.00386 | 0.579 | 0.00380 | | 60 | 0.575 | 0.00401 | 0.577 | 0.00395 | 0.577 | 0.00406 |
| | 80 | 0.596 | 0.00356 | 0.597 | 0.00354 | 0.597 | 0.00362 | | 80 | 0.594 | 0.00364 | 0.594 | 0.00344 | 0.596 | 0.00368 |
| | 100 | 0.605 | 0.00339 | 0.605 | 0.00343 | 0.606 | 0.00346 | | 100 | 0.605 | 0.00339 | 0.605 | 0.00343 | 0.606 | 0.00346 |

**Table S23** Average accuracy and its corresponding standard error of the mean (SEM) across the 40 iterations for all training set optimization methods, models and training set (TRS) sizes (expressed as percentage of the candidate set (CS)) for soybean dataset and the simulated trait. If "targ" is added at the end of the name of a method, it corresponds to targeted optimization. Otherwise, untargeted optimization was performed.

**Soy trait simulated1**

| Optimization method | TRS size (% of CS) | Average accuracy | | | | | | Optimization method | TRS size (% of CS) | Average accuracy | | | | | |
|---|---|---|---|---|---|---|---|---|---|---|---|---|---|---|---|
| | | GBLUP | | BayesB | | RKHS | | | | GBLUP | | BayesB | | RKHS | |
| | | Mean | SEM | Mean | SEM | Mean | SEM | | | Mean | SEM | Mean | SEM | Mean | SEM |
| RAND | 10 | 0.446 | 0.00806 | 0.446 | 0.00799 | 0.442 | 0.00783 | Rscore | 10 | 0.455 | 0.00884 | 0.455 | 0.00875 | 0.453 | 0.00871 |
| | 20 | 0.528 | 0.00578 | 0.527 | 0.00595 | 0.524 | 0.00574 | | 20 | 0.526 | 0.00550 | 0.525 | 0.00548 | 0.523 | 0.00522 |
| | 40 | 0.580 | 0.00389 | 0.579 | 0.00387 | 0.578 | 0.00384 | | 40 | 0.586 | 0.00392 | 0.585 | 0.00397 | 0.583 | 0.00395 |
| | 60 | 0.609 | 0.00403 | 0.608 | 0.00403 | 0.607 | 0.00405 | | 60 | 0.613 | 0.00354 | 0.612 | 0.00352 | 0.611 | 0.00352 |
| | 80 | 0.624 | 0.00314 | 0.624 | 0.00308 | 0.622 | 0.00306 | | 80 | 0.629 | 0.00343 | 0.628 | 0.00338 | 0.627 | 0.00353 |
| | 100 | 0.638 | 0.00313 | 0.637 | 0.00317 | 0.635 | 0.00316 | | 100 | 0.638 | 0.00313 | 0.637 | 0.00317 | 0.635 | 0.00316 |
| StratSamp | 10 | 0.453 | 0.00806 | 0.454 | 0.00778 | 0.451 | 0.00809 | Rscoretarg | 10 | 0.471 | 0.00669 | 0.471 | 0.00664 | 0.467 | 0.00702 |
| | 20 | 0.528 | 0.00648 | 0.528 | 0.00640 | 0.525 | 0.00617 | | 20 | 0.531 | 0.00606 | 0.530 | 0.00618 | 0.526 | 0.00624 |
| | 40 | 0.592 | 0.00432 | 0.591 | 0.00445 | 0.590 | 0.00431 | | 40 | 0.591 | 0.00390 | 0.590 | 0.00399 | 0.588 | 0.00379 |
| | 60 | 0.614 | 0.00323 | 0.612 | 0.00333 | 0.611 | 0.00326 | | 60 | 0.615 | 0.00381 | 0.614 | 0.00388 | 0.613 | 0.00380 |
| | 80 | 0.627 | 0.00324 | 0.626 | 0.00328 | 0.625 | 0.00327 | | 80 | 0.628 | 0.00313 | 0.627 | 0.00312 | 0.626 | 0.00310 |
| | 100 | 0.638 | 0.00313 | 0.637 | 0.00317 | 0.635 | 0.00316 | | 100 | 0.638 | 0.00313 | 0.637 | 0.00317 | 0.635 | 0.00316 |
| CD | 10 | 0.469 | 0.00800 | 0.471 | 0.00785 | 0.465 | 0.00830 | Avg_GRM | 10 | 0.425 | 0.00921 | 0.426 | 0.00906 | 0.423 | 0.00951 |
| | 20 | 0.540 | 0.00497 | 0.540 | 0.00484 | 0.538 | 0.00487 | | 20 | 0.513 | 0.00808 | 0.512 | 0.00807 | 0.511 | 0.00793 |
| | 40 | 0.590 | 0.00337 | 0.589 | 0.00339 | 0.589 | 0.00330 | | 40 | 0.581 | 0.00421 | 0.580 | 0.00426 | 0.579 | 0.00432 |
| | 60 | 0.614 | 0.00351 | 0.614 | 0.00348 | 0.612 | 0.00353 | | 60 | 0.612 | 0.00350 | 0.611 | 0.00355 | 0.609 | 0.00357 |
| | 80 | 0.629 | 0.00355 | 0.628 | 0.00352 | 0.626 | 0.00358 | | 80 | 0.628 | 0.00328 | 0.627 | 0.00332 | 0.626 | 0.00327 |
| | 100 | 0.638 | 0.00313 | 0.637 | 0.00317 | 0.635 | 0.00316 | | 100 | 0.638 | 0.00313 | 0.637 | 0.00317 | 0.635 | 0.00316 |
| CDtarg | 10 | 0.477 | 0.00858 | 0.477 | 0.00842 | 0.475 | 0.00872 | Avg_GRMtarg | 10 | 0.441 | 0.00979 | 0.440 | 0.00998 | 0.439 | 0.00984 |
| | 20 | 0.543 | 0.00523 | 0.543 | 0.00527 | 0.540 | 0.00551 | | 20 | 0.521 | 0.00678 | 0.521 | 0.00665 | 0.519 | 0.00664 |
| | 40 | 0.594 | 0.00379 | 0.593 | 0.00391 | 0.592 | 0.00371 | | 40 | 0.586 | 0.00463 | 0.585 | 0.00464 | 0.585 | 0.00476 |
| | 60 | 0.618 | 0.00326 | 0.617 | 0.00326 | 0.616 | 0.00333 | | 60 | 0.614 | 0.00358 | 0.613 | 0.00366 | 0.613 | 0.00365 |
| | 80 | 0.630 | 0.00322 | 0.628 | 0.00327 | 0.627 | 0.00326 | | 80 | 0.628 | 0.00329 | 0.627 | 0.00333 | 0.626 | 0.00330 |
| | 100 | 0.638 | 0.00313 | 0.637 | 0.00317 | 0.635 | 0.00316 | | 100 | 0.638 | 0.00313 | 0.637 | 0.00317 | 0.635 | 0.00316 |
| OvClustCD | 10 | 0.471 | 0.00740 | 0.472 | 0.00729 | 0.468 | 0.00731 | Avg_GRM_MinMax | 10 | 0.461 | 0.01004 | 0.461 | 0.01002 | 0.459 | 0.01033 |
| | 20 | 0.537 | 0.00554 | 0.535 | 0.00557 | 0.531 | 0.00570 | | 20 | 0.530 | 0.00500 | 0.530 | 0.00491 | 0.527 | 0.00484 |
| | 40 | 0.593 | 0.00414 | 0.593 | 0.00417 | 0.591 | 0.00417 | | 40 | 0.592 | 0.00421 | 0.592 | 0.00421 | 0.591 | 0.00430 |
| | 60 | 0.617 | 0.00349 | 0.616 | 0.00351 | 0.616 | 0.00346 | | 60 | 0.615 | 0.00312 | 0.614 | 0.00313 | 0.613 | 0.00313 |
| | 80 | 0.630 | 0.00316 | 0.629 | 0.00321 | 0.628 | 0.00315 | | 80 | 0.630 | 0.00327 | 0.629 | 0.00330 | 0.628 | 0.00335 |
| | 100 | 0.638 | 0.00313 | 0.637 | 0.00317 | 0.635 | 0.00316 | | 100 | 0.638 | 0.00313 | 0.637 | 0.00317 | 0.635 | 0.00316 |
| OvClustCDtarg | 10 | 0.462 | 0.00821 | 0.463 | 0.00785 | 0.459 | 0.00835 | Avg_GRM_MinMaxtarg | 10 | 0.437 | 0.01160 | 0.439 | 0.01055 | 0.436 | 0.01167 |
| | 20 | 0.545 | 0.00479 | 0.545 | 0.00477 | 0.542 | 0.00470 | | 20 | 0.534 | 0.00573 | 0.535 | 0.00570 | 0.532 | 0.00593 |
| | 40 | 0.595 | 0.00411 | 0.594 | 0.00418 | 0.592 | 0.00418 | | 40 | 0.587 | 0.00431 | 0.586 | 0.00426 | 0.585 | 0.00433 |
| | 60 | 0.615 | 0.00353 | 0.614 | 0.00357 | 0.613 | 0.00353 | | 60 | 0.614 | 0.00367 | 0.613 | 0.00365 | 0.612 | 0.00373 |
| | 80 | 0.630 | 0.00326 | 0.630 | 0.00324 | 0.628 | 0.00329 | | 80 | 0.629 | 0.00345 | 0.628 | 0.00345 | 0.627 | 0.00353 |
| | 100 | 0.638 | 0.00313 | 0.637 | 0.00317 | 0.635 | 0.00316 | | 100 | 0.638 | 0.00313 | 0.637 | 0.00317 | 0.635 | 0.00316 |
| WIClustCD | 10 | 0.466 | 0.00741 | 0.465 | 0.00742 | 0.462 | 0.00751 | Avg_GRM_self | 10 | 0.450 | 0.00776 | 0.450 | 0.00777 | 0.449 | 0.00774 |
| | 20 | 0.528 | 0.00466 | 0.528 | 0.00468 | 0.525 | 0.00472 | | 20 | 0.528 | 0.00692 | 0.528 | 0.00682 | 0.526 | 0.00690 |
| | 40 | 0.590 | 0.00359 | 0.590 | 0.00361 | 0.588 | 0.00372 | | 40 | 0.588 | 0.00406 | 0.587 | 0.00406 | 0.585 | 0.00404 |
| | 60 | 0.615 | 0.00342 | 0.614 | 0.00341 | 0.613 | 0.00340 | | 60 | 0.614 | 0.00343 | 0.614 | 0.00351 | 0.612 | 0.00351 |
| | 80 | 0.628 | 0.00326 | 0.627 | 0.00330 | 0.626 | 0.00323 | | 80 | 0.629 | 0.00326 | 0.628 | 0.00327 | 0.627 | 0.00327 |
| | 100 | 0.638 | 0.00313 | 0.637 | 0.00317 | 0.635 | 0.00316 | | 100 | 0.638 | 0.00313 | 0.637 | 0.00317 | 0.635 | 0.00316 |
| WIClustCDtarg | 10 | 0.470 | 0.00780 | 0.470 | 0.00773 | 0.466 | 0.00787 | PAM | 10 | 0.447 | 0.00668 | 0.448 | 0.00679 | 0.443 | 0.00712 |
| | 20 | 0.538 | 0.00414 | 0.538 | 0.00412 | 0.535 | 0.00406 | | 20 | 0.531 | 0.00492 | 0.530 | 0.00483 | 0.529 | 0.00491 |
| | 40 | 0.592 | 0.00318 | 0.591 | 0.00325 | 0.588 | 0.00338 | | 40 | 0.590 | 0.00399 | 0.589 | 0.00394 | 0.588 | 0.00394 |
| | 60 | 0.615 | 0.00323 | 0.614 | 0.00330 | 0.612 | 0.00336 | | 60 | 0.613 | 0.00343 | 0.612 | 0.00340 | 0.611 | 0.00340 |
| | 80 | 0.629 | 0.00303 | 0.627 | 0.00306 | 0.626 | 0.00308 | | 80 | 0.628 | 0.00322 | 0.627 | 0.00331 | 0.625 | 0.00321 |
| | 100 | 0.638 | 0.00313 | 0.637 | 0.00317 | 0.635 | 0.00316 | | 100 | 0.638 | 0.00313 | 0.637 | 0.00317 | 0.635 | 0.00316 |

**Table S24** Average accuracy and its corresponding standard error of the mean (SEM) across the 40 iterations for all training set optimization methods, models and training set (TRS) sizes (expressed as percentage of the candidate set (CS)) for spruce dataset and DBH trait. If "targ" is added at the end of the name of a method, it corresponds to targeted optimization. Otherwise, untargeted optimization was performed.

**Spruce trait DBH**

| | | Average accuracy | | | | | | | | Average accuracy | | | | | |
|---|---|---|---|---|---|---|---|---|---|---|---|---|---|---|---|
| | | GBLUP | | BayesB | | RKHS | | | | GBLUP | | BayesB | | RKHS | |
| Optimization method | TRS size (% of CS) | Mean | SEM | Mean | SEM | Mean | SEM | Optimization method | TRS size (% of CS) | Mean | SEM | Mean | SEM | Mean | SEM |
| RAND | 10 | 0.1285 | 0.00952 | 0.1290 | 0.00892 | 0.135 | 0.00947 | Rscore | 10 | 0.1380 | 0.01085 | 0.1380 | 0.01080 | 0.144 | 0.01089 |
| | 20 | 0.1752 | 0.00938 | 0.1751 | 0.00933 | 0.186 | 0.00975 | | 20 | 0.1646 | 0.00793 | 0.1671 | 0.00846 | 0.177 | 0.00802 |
| | 40 | 0.2092 | 0.00634 | 0.2084 | 0.00643 | 0.225 | 0.00612 | | 40 | 0.2033 | 0.00790 | 0.2000 | 0.00780 | 0.218 | 0.00796 |
| | 60 | 0.2309 | 0.00742 | 0.2289 | 0.00730 | 0.249 | 0.00753 | | 60 | 0.2300 | 0.00833 | 0.2270 | 0.00849 | 0.247 | 0.00871 |
| | 80 | 0.2398 | 0.00810 | 0.2374 | 0.00819 | 0.258 | 0.00804 | | 80 | 0.2358 | 0.00752 | 0.2324 | 0.00746 | 0.254 | 0.00769 |
| | 100 | 0.2443 | 0.00756 | 0.2422 | 0.00752 | 0.264 | 0.00758 | | 100 | 0.2443 | 0.00756 | 0.2422 | 0.00752 | 0.264 | 0.00758 |
| StratSamp | 10 | 0.1281 | 0.00865 | 0.1311 | 0.00847 | 0.136 | 0.00885 | Rscoretarg | 10 | 0.1187 | 0.01093 | 0.1257 | 0.01038 | 0.126 | 0.01078 |
| | 20 | 0.1759 | 0.00828 | 0.1793 | 0.00830 | 0.186 | 0.00800 | | 20 | 0.1616 | 0.00942 | 0.1618 | 0.00932 | 0.174 | 0.00925 |
| | 40 | 0.2131 | 0.00796 | 0.2120 | 0.00784 | 0.228 | 0.00820 | | 40 | 0.2067 | 0.00820 | 0.2055 | 0.00824 | 0.221 | 0.00850 |
| | 60 | 0.2256 | 0.00697 | 0.2226 | 0.00689 | 0.243 | 0.00685 | | 60 | 0.2288 | 0.00803 | 0.2262 | 0.00809 | 0.246 | 0.00820 |
| | 80 | 0.2370 | 0.00727 | 0.2354 | 0.00748 | 0.255 | 0.00730 | | 80 | 0.2397 | 0.00805 | 0.2376 | 0.00815 | 0.258 | 0.00806 |
| | 100 | 0.2443 | 0.00756 | 0.2422 | 0.00752 | 0.264 | 0.00758 | | 100 | 0.2443 | 0.00756 | 0.2422 | 0.00752 | 0.264 | 0.00758 |
| CD | 10 | 0.1327 | 0.00837 | 0.1350 | 0.00813 | 0.141 | 0.00842 | Avg_GRM | 10 | 0.0542 | 0.00886 | 0.0472 | 0.00910 | 0.062 | 0.00859 |
| | 20 | 0.1908 | 0.00959 | 0.1943 | 0.00871 | 0.206 | 0.00949 | | 20 | 0.0940 | 0.00983 | 0.0936 | 0.01031 | 0.106 | 0.01010 |
| | 40 | 0.2072 | 0.00923 | 0.2056 | 0.00930 | 0.221 | 0.00949 | | 40 | 0.1769 | 0.00871 | 0.1760 | 0.00852 | 0.195 | 0.00871 |
| | 60 | 0.2234 | 0.00771 | 0.2215 | 0.00776 | 0.239 | 0.00793 | | 60 | 0.1994 | 0.00782 | 0.1983 | 0.00773 | 0.218 | 0.00778 |
| | 80 | 0.2407 | 0.00701 | 0.2382 | 0.00689 | 0.258 | 0.00699 | | 80 | 0.2367 | 0.00758 | 0.2329 | 0.00757 | 0.256 | 0.00778 |
| | 100 | 0.2443 | 0.00756 | 0.2422 | 0.00752 | 0.264 | 0.00758 | | 100 | 0.2443 | 0.00756 | 0.2422 | 0.00752 | 0.264 | 0.00758 |
| CDtarg | 10 | 0.1342 | 0.01091 | 0.1371 | 0.01086 | 0.143 | 0.01066 | Avg_GRMtarg | 10 | 0.1078 | 0.00887 | 0.0956 | 0.00973 | 0.114 | 0.00872 |
| | 20 | 0.1985 | 0.00710 | 0.1984 | 0.00680 | 0.209 | 0.00695 | | 20 | 0.1522 | 0.00852 | 0.1489 | 0.00844 | 0.163 | 0.00848 |
| | 40 | 0.2336 | 0.00874 | 0.2325 | 0.00880 | 0.247 | 0.00864 | | 40 | 0.2124 | 0.00760 | 0.2080 | 0.00761 | 0.225 | 0.00746 |
| | 60 | 0.2452 | 0.00846 | 0.2435 | 0.00843 | 0.262 | 0.00858 | | 60 | 0.2244 | 0.00818 | 0.2217 | 0.00807 | 0.241 | 0.00809 |
| | 80 | 0.2424 | 0.00743 | 0.2402 | 0.00733 | 0.261 | 0.00762 | | 80 | 0.2379 | 0.00793 | 0.2356 | 0.00809 | 0.256 | 0.00789 |
| | 100 | 0.2443 | 0.00756 | 0.2422 | 0.00752 | 0.264 | 0.00758 | | 100 | 0.2443 | 0.00756 | 0.2422 | 0.00752 | 0.264 | 0.00758 |
| OvClustCD | 10 | 0.1506 | 0.00955 | 0.1532 | 0.00971 | 0.159 | 0.00966 | Avg_GRM_MinMax | 10 | 0.1263 | 0.01018 | 0.1321 | 0.01060 | 0.135 | 0.01024 |
| | 20 | 0.1723 | 0.00826 | 0.1745 | 0.00767 | 0.185 | 0.00827 | | 20 | 0.1850 | 0.01086 | 0.1841 | 0.01049 | 0.196 | 0.01087 |
| | 40 | 0.2122 | 0.00851 | 0.2101 | 0.00865 | 0.226 | 0.00851 | | 40 | 0.2166 | 0.00920 | 0.2151 | 0.00919 | 0.232 | 0.00896 |
| | 60 | 0.2296 | 0.00690 | 0.2282 | 0.00704 | 0.244 | 0.00689 | | 60 | 0.2293 | 0.00779 | 0.2273 | 0.00793 | 0.245 | 0.00777 |
| | 80 | 0.2379 | 0.00706 | 0.2359 | 0.00703 | 0.255 | 0.00700 | | 80 | 0.2340 | 0.00748 | 0.2310 | 0.00741 | 0.253 | 0.00760 |
| | 100 | 0.2443 | 0.00756 | 0.2422 | 0.00752 | 0.264 | 0.00758 | | 100 | 0.2443 | 0.00756 | 0.2422 | 0.00752 | 0.264 | 0.00758 |
| OvClustCDtarg | 10 | 0.1526 | 0.00986 | 0.1583 | 0.00996 | 0.161 | 0.01002 | Avg_GRM_MinMaxtarg | 10 | 0.1297 | 0.01022 | 0.1364 | 0.01006 | 0.138 | 0.01015 |
| | 20 | 0.1950 | 0.00824 | 0.1953 | 0.00858 | 0.206 | 0.00833 | | 20 | 0.1890 | 0.01071 | 0.1910 | 0.01080 | 0.199 | 0.01077 |
| | 40 | 0.2263 | 0.00849 | 0.2241 | 0.00846 | 0.241 | 0.00850 | | 40 | 0.2163 | 0.00760 | 0.2147 | 0.00775 | 0.231 | 0.00765 |
| | 60 | 0.2353 | 0.00764 | 0.2325 | 0.00741 | 0.252 | 0.00764 | | 60 | 0.2339 | 0.00877 | 0.2311 | 0.00861 | 0.250 | 0.00873 |
| | 80 | 0.2367 | 0.00773 | 0.2344 | 0.00785 | 0.255 | 0.00758 | | 80 | 0.2376 | 0.00784 | 0.2348 | 0.00807 | 0.256 | 0.00798 |
| | 100 | 0.2443 | 0.00756 | 0.2422 | 0.00752 | 0.264 | 0.00758 | | 100 | 0.2443 | 0.00756 | 0.2422 | 0.00752 | 0.264 | 0.00758 |
| WIClustCD | 10 | 0.1398 | 0.00998 | 0.1525 | 0.00960 | 0.150 | 0.01004 | Avg_GRM_self | 10 | 0.1289 | 0.01191 | 0.1341 | 0.01230 | 0.138 | 0.01213 |
| | 20 | 0.1705 | 0.00997 | 0.1757 | 0.00931 | 0.186 | 0.01007 | | 20 | 0.1744 | 0.00862 | 0.1762 | 0.00875 | 0.185 | 0.00874 |
| | 40 | 0.2097 | 0.00834 | 0.2061 | 0.00860 | 0.223 | 0.00855 | | 40 | 0.2132 | 0.00856 | 0.2115 | 0.00866 | 0.228 | 0.00869 |
| | 60 | 0.2299 | 0.00706 | 0.2268 | 0.00694 | 0.246 | 0.00710 | | 60 | 0.2320 | 0.00827 | 0.2309 | 0.00820 | 0.249 | 0.00831 |
| | 80 | 0.2407 | 0.00780 | 0.2398 | 0.00779 | 0.257 | 0.00787 | | 80 | 0.2406 | 0.00739 | 0.2389 | 0.00751 | 0.259 | 0.00736 |
| | 100 | 0.2443 | 0.00756 | 0.2422 | 0.00752 | 0.264 | 0.00758 | | 100 | 0.2443 | 0.00756 | 0.2422 | 0.00752 | 0.264 | 0.00758 |
| WIClustCDtarg | 10 | 0.1525 | 0.00986 | 0.1577 | 0.01017 | 0.162 | 0.00976 | PAM | 10 | 0.1283 | 0.01085 | 0.1383 | 0.01053 | 0.139 | 0.01066 |
| | 20 | 0.1993 | 0.00772 | 0.2008 | 0.00772 | 0.213 | 0.00783 | | 20 | 0.1753 | 0.00762 | 0.1805 | 0.00751 | 0.188 | 0.00757 |
| | 40 | 0.2329 | 0.00720 | 0.2325 | 0.00715 | 0.248 | 0.00732 | | 40 | 0.2025 | 0.00780 | 0.2014 | 0.00780 | 0.220 | 0.00757 |
| | 60 | 0.2418 | 0.00755 | 0.2400 | 0.00739 | 0.258 | 0.00752 | | 60 | 0.2311 | 0.00793 | 0.2290 | 0.00778 | 0.250 | 0.00790 |
| | 80 | 0.2444 | 0.00729 | 0.2413 | 0.00720 | 0.263 | 0.00731 | | 80 | 0.2411 | 0.00781 | 0.2388 | 0.00789 | 0.262 | 0.00778 |
| | 100 | 0.2443 | 0.00756 | 0.2422 | 0.00752 | 0.264 | 0.00758 | | 100 | 0.2443 | 0.00756 | 0.2422 | 0.00752 | 0.264 | 0.00758 |

**Table S25** Average accuracy and its corresponding standard error of the mean (SEM) across the 40 iterations for all training set optimization methods, models and training set (TRS) sizes (expressed as percentage of the candidate set (CS)) for spruce dataset and DE trait. If "targ" is added at the end of the name of a method, it corresponds to targeted optimization. Otherwise, untargeted optimization was performed.

**Spruce trait DE**

| Optimization method | TRS size (% of CS) | Average accuracy | | | | | | Optimization method | TRS size (% of CS) | Average accuracy | | | | | |
|---|---|---|---|---|---|---|---|---|---|---|---|---|---|---|---|
| | | GBLUP | | BayesB | | RKHS | | | | GBLUP | | BayesB | | RKHS | |
| | | Mean | SEM | Mean | SEM | Mean | SEM | | | Mean | SEM | Mean | SEM | Mean | SEM |
| RAND | 10 | 0.282 | 0.00735 | 0.282 | 0.00747 | 0.283 | 0.00741 | Rscore | 10 | 0.301 | 0.00910 | 0.300 | 0.00927 | 0.302 | 0.00902 |
| | 20 | 0.337 | 0.00806 | 0.336 | 0.00805 | 0.339 | 0.00807 | | 20 | 0.342 | 0.00721 | 0.339 | 0.00707 | 0.343 | 0.00720 |
| | 40 | 0.378 | 0.00668 | 0.377 | 0.00683 | 0.380 | 0.00644 | | 40 | 0.382 | 0.00698 | 0.381 | 0.00701 | 0.384 | 0.00701 |
| | 60 | 0.398 | 0.00692 | 0.398 | 0.00694 | 0.402 | 0.00673 | | 60 | 0.396 | 0.00632 | 0.395 | 0.00636 | 0.399 | 0.00627 |
| | 80 | 0.405 | 0.00636 | 0.405 | 0.00630 | 0.411 | 0.00610 | | 80 | 0.405 | 0.00667 | 0.405 | 0.00675 | 0.410 | 0.00636 |
| | 100 | 0.416 | 0.00625 | 0.416 | 0.00620 | 0.423 | 0.00596 | | 100 | 0.416 | 0.00625 | 0.416 | 0.00620 | 0.423 | 0.00596 |
| StratSamp | 10 | 0.293 | 0.01094 | 0.293 | 0.01080 | 0.293 | 0.01092 | Rscoretarg | 10 | 0.301 | 0.00903 | 0.300 | 0.00955 | 0.300 | 0.00912 |
| | 20 | 0.331 | 0.00817 | 0.330 | 0.00849 | 0.332 | 0.00808 | | 20 | 0.337 | 0.00846 | 0.336 | 0.00858 | 0.337 | 0.00832 |
| | 40 | 0.366 | 0.00708 | 0.365 | 0.00715 | 0.368 | 0.00687 | | 40 | 0.382 | 0.00728 | 0.381 | 0.00727 | 0.384 | 0.00708 |
| | 60 | 0.392 | 0.00701 | 0.391 | 0.00702 | 0.397 | 0.00675 | | 60 | 0.400 | 0.00652 | 0.398 | 0.00638 | 0.404 | 0.00631 |
| | 80 | 0.408 | 0.00640 | 0.406 | 0.00647 | 0.413 | 0.00632 | | 80 | 0.412 | 0.00585 | 0.411 | 0.00591 | 0.418 | 0.00569 |
| | 100 | 0.416 | 0.00625 | 0.416 | 0.00620 | 0.423 | 0.00596 | | 100 | 0.416 | 0.00625 | 0.416 | 0.00620 | 0.423 | 0.00596 |
| CD | 10 | 0.296 | 0.00872 | 0.297 | 0.00869 | 0.297 | 0.00864 | Avg_GRM | 10 | 0.162 | 0.01321 | 0.157 | 0.01275 | 0.162 | 0.01322 |
| | 20 | 0.356 | 0.00758 | 0.354 | 0.00789 | 0.358 | 0.00755 | | 20 | 0.254 | 0.01032 | 0.250 | 0.01087 | 0.255 | 0.01040 |
| | 40 | 0.379 | 0.00746 | 0.379 | 0.00759 | 0.381 | 0.00731 | | 40 | 0.340 | 0.00767 | 0.337 | 0.00793 | 0.342 | 0.00743 |
| | 60 | 0.397 | 0.00623 | 0.397 | 0.00647 | 0.401 | 0.00604 | | 60 | 0.377 | 0.00747 | 0.374 | 0.00755 | 0.380 | 0.00723 |
| | 80 | 0.405 | 0.00666 | 0.405 | 0.00678 | 0.411 | 0.00643 | | 80 | 0.401 | 0.00677 | 0.401 | 0.00676 | 0.407 | 0.00651 |
| | 100 | 0.416 | 0.00625 | 0.416 | 0.00620 | 0.423 | 0.00596 | | 100 | 0.416 | 0.00625 | 0.416 | 0.00620 | 0.423 | 0.00596 |
| CDtarg | 10 | 0.311 | 0.00870 | 0.309 | 0.00886 | 0.311 | 0.00864 | Avg_GRMtarg | 10 | 0.207 | 0.01109 | 0.207 | 0.01009 | 0.209 | 0.01064 |
| | 20 | 0.357 | 0.00749 | 0.355 | 0.00721 | 0.357 | 0.00730 | | 20 | 0.298 | 0.00871 | 0.298 | 0.00851 | 0.300 | 0.00859 |
| | 40 | 0.389 | 0.00567 | 0.389 | 0.00565 | 0.391 | 0.00565 | | 40 | 0.368 | 0.00800 | 0.368 | 0.00813 | 0.371 | 0.00808 |
| | 60 | 0.405 | 0.00649 | 0.404 | 0.00670 | 0.410 | 0.00636 | | 60 | 0.395 | 0.00618 | 0.394 | 0.00626 | 0.399 | 0.00607 |
| | 80 | 0.410 | 0.00624 | 0.409 | 0.00625 | 0.416 | 0.00609 | | 80 | 0.409 | 0.00596 | 0.409 | 0.00602 | 0.415 | 0.00577 |
| | 100 | 0.416 | 0.00625 | 0.416 | 0.00620 | 0.423 | 0.00596 | | 100 | 0.416 | 0.00625 | 0.416 | 0.00620 | 0.423 | 0.00596 |
| OvClustCD | 10 | 0.306 | 0.00920 | 0.303 | 0.00934 | 0.308 | 0.00933 | Avg_GRM_MinMax | 10 | 0.309 | 0.00809 | 0.310 | 0.00807 | 0.310 | 0.00808 |
| | 20 | 0.346 | 0.00700 | 0.343 | 0.00699 | 0.347 | 0.00684 | | 20 | 0.342 | 0.00641 | 0.341 | 0.00617 | 0.343 | 0.00648 |
| | 40 | 0.374 | 0.00545 | 0.372 | 0.00561 | 0.376 | 0.00533 | | 40 | 0.375 | 0.00591 | 0.374 | 0.00596 | 0.378 | 0.00588 |
| | 60 | 0.395 | 0.00705 | 0.394 | 0.00710 | 0.399 | 0.00689 | | 60 | 0.396 | 0.00646 | 0.395 | 0.00640 | 0.399 | 0.00637 |
| | 80 | 0.407 | 0.00613 | 0.407 | 0.00627 | 0.413 | 0.00581 | | 80 | 0.406 | 0.00592 | 0.405 | 0.00588 | 0.412 | 0.00573 |
| | 100 | 0.416 | 0.00625 | 0.416 | 0.00620 | 0.423 | 0.00596 | | 100 | 0.416 | 0.00625 | 0.416 | 0.00620 | 0.423 | 0.00596 |
| OvClustCDtarg | 10 | 0.316 | 0.00798 | 0.315 | 0.00784 | 0.316 | 0.00805 | Avg_GRM_MinMaxtarg | 10 | 0.307 | 0.00883 | 0.306 | 0.00880 | 0.307 | 0.00874 |
| | 20 | 0.351 | 0.00650 | 0.351 | 0.00641 | 0.352 | 0.00633 | | 20 | 0.344 | 0.00622 | 0.342 | 0.00631 | 0.345 | 0.00622 |
| | 40 | 0.391 | 0.00648 | 0.390 | 0.00643 | 0.393 | 0.00636 | | 40 | 0.382 | 0.00647 | 0.382 | 0.00622 | 0.384 | 0.00641 |
| | 60 | 0.404 | 0.00596 | 0.405 | 0.00608 | 0.409 | 0.00584 | | 60 | 0.401 | 0.00675 | 0.401 | 0.00664 | 0.405 | 0.00663 |
| | 80 | 0.412 | 0.00623 | 0.412 | 0.00615 | 0.418 | 0.00598 | | 80 | 0.409 | 0.00614 | 0.408 | 0.00613 | 0.414 | 0.00588 |
| | 100 | 0.416 | 0.00625 | 0.416 | 0.00620 | 0.423 | 0.00596 | | 100 | 0.416 | 0.00625 | 0.416 | 0.00620 | 0.423 | 0.00596 |
| WIClustCD | 10 | 0.310 | 0.00944 | 0.309 | 0.00938 | 0.313 | 0.00923 | Avg_GRM_self | 10 | 0.303 | 0.00921 | 0.303 | 0.00926 | 0.303 | 0.00919 |
| | 20 | 0.353 | 0.00902 | 0.353 | 0.00883 | 0.356 | 0.00871 | | 20 | 0.337 | 0.00928 | 0.335 | 0.00925 | 0.337 | 0.00928 |
| | 40 | 0.385 | 0.00678 | 0.383 | 0.00676 | 0.386 | 0.00652 | | 40 | 0.376 | 0.00662 | 0.374 | 0.00672 | 0.378 | 0.00657 |
| | 60 | 0.400 | 0.00684 | 0.399 | 0.00694 | 0.404 | 0.00658 | | 60 | 0.396 | 0.00640 | 0.395 | 0.00645 | 0.399 | 0.00615 |
| | 80 | 0.408 | 0.00622 | 0.408 | 0.00624 | 0.413 | 0.00614 | | 80 | 0.404 | 0.00650 | 0.404 | 0.00649 | 0.410 | 0.00625 |
| | 100 | 0.416 | 0.00625 | 0.416 | 0.00620 | 0.423 | 0.00596 | | 100 | 0.416 | 0.00625 | 0.416 | 0.00620 | 0.423 | 0.00596 |
| WIClustCDtarg | 10 | 0.323 | 0.00730 | 0.322 | 0.00728 | 0.324 | 0.00722 | PAM | 10 | 0.301 | 0.01039 | 0.299 | 0.01066 | 0.300 | 0.01034 |
| | 20 | 0.363 | 0.00657 | 0.363 | 0.00647 | 0.363 | 0.00653 | | 20 | 0.326 | 0.00860 | 0.323 | 0.00832 | 0.326 | 0.00832 |
| | 40 | 0.397 | 0.00644 | 0.396 | 0.00634 | 0.399 | 0.00621 | | 40 | 0.370 | 0.00661 | 0.369 | 0.00674 | 0.374 | 0.00641 |
| | 60 | 0.406 | 0.00641 | 0.406 | 0.00632 | 0.410 | 0.00618 | | 60 | 0.398 | 0.00711 | 0.398 | 0.00705 | 0.404 | 0.00685 |
| | 80 | 0.413 | 0.00595 | 0.413 | 0.00600 | 0.419 | 0.00572 | | 80 | 0.409 | 0.00681 | 0.409 | 0.00703 | 0.416 | 0.00655 |
| | 100 | 0.416 | 0.00625 | 0.416 | 0.00620 | 0.423 | 0.00596 | | 100 | 0.416 | 0.00625 | 0.416 | 0.00620 | 0.423 | 0.00596 |

**Table S26** Average accuracy and its corresponding standard error of the mean (SEM) across the 40 iterations for all training set optimization methods, models and training set (TRS) sizes (expressed as percentage of the candidate set (CS)) for spruce dataset and HT trait. If "targ" is added at the end of the name of a method, it corresponds to targeted optimization. Otherwise, untargeted optimization was performed.

**Spruce trait HT**

| Optimization method | TRS size (% of CS) | Average accuracy GBLUP | | BayesB | | RKHS | | Optimization method | TRS size (% of CS) | Average accuracy GBLUP | | BayesB | | RKHS | |
|---|---|---|---|---|---|---|---|---|---|---|---|---|---|---|---|
| | | Mean | SEM | Mean | SEM | Mean | SEM | | | Mean | SEM | Mean | SEM | Mean | SEM |
| RAND | 10 | 0.2051 | 0.00987 | 0.2074 | 0.00929 | 0.2134 | 0.01012 | Rscore | 10 | 0.1984 | 0.00949 | 0.2010 | 0.00899 | 0.2097 | 0.00929 |
| | 20 | 0.2373 | 0.00844 | 0.2356 | 0.00847 | 0.2493 | 0.00865 | | 20 | 0.2343 | 0.00703 | 0.2352 | 0.00708 | 0.2486 | 0.00705 |
| | 40 | 0.2817 | 0.00542 | 0.2784 | 0.00531 | 0.2988 | 0.00543 | | 40 | 0.2768 | 0.00789 | 0.2756 | 0.00803 | 0.2923 | 0.00785 |
| | 60 | 0.3072 | 0.00788 | 0.3039 | 0.00773 | 0.3247 | 0.00802 | | 60 | 0.3040 | 0.00663 | 0.3016 | 0.00665 | 0.3209 | 0.00710 |
| | 80 | 0.3096 | 0.00688 | 0.3074 | 0.00688 | 0.3288 | 0.00710 | | 80 | 0.3106 | 0.00702 | 0.3089 | 0.00692 | 0.3289 | 0.00725 |
| | 100 | 0.3184 | 0.00655 | 0.3163 | 0.00660 | 0.3390 | 0.00686 | | 100 | 0.3184 | 0.00655 | 0.3163 | 0.00660 | 0.3390 | 0.00686 |
| StratSamp | 10 | 0.2062 | 0.01011 | 0.2068 | 0.00975 | 0.2172 | 0.00982 | Rscoretarg | 10 | 0.1971 | 0.00955 | 0.2001 | 0.00950 | 0.2084 | 0.00956 |
| | 20 | 0.2447 | 0.00780 | 0.2442 | 0.00771 | 0.2561 | 0.00758 | | 20 | 0.2404 | 0.00929 | 0.2400 | 0.00924 | 0.2531 | 0.00925 |
| | 40 | 0.2906 | 0.00734 | 0.2885 | 0.00729 | 0.3059 | 0.00731 | | 40 | 0.2823 | 0.00720 | 0.2798 | 0.00705 | 0.2974 | 0.00736 |
| | 60 | 0.3009 | 0.00693 | 0.2985 | 0.00682 | 0.3184 | 0.00692 | | 60 | 0.3039 | 0.00722 | 0.3018 | 0.00718 | 0.3219 | 0.00741 |
| | 80 | 0.3099 | 0.00638 | 0.3085 | 0.00648 | 0.3293 | 0.00649 | | 80 | 0.3114 | 0.00672 | 0.3103 | 0.00664 | 0.3303 | 0.00692 |
| | 100 | 0.3184 | 0.00655 | 0.3163 | 0.00660 | 0.3390 | 0.00686 | | 100 | 0.3184 | 0.00655 | 0.3163 | 0.00660 | 0.3390 | 0.00686 |
| CD | 10 | 0.2028 | 0.00868 | 0.2040 | 0.00923 | 0.2143 | 0.00865 | Avg_GRM | 10 | 0.0872 | 0.01006 | 0.0854 | 0.01001 | 0.0983 | 0.00985 |
| | 20 | 0.2615 | 0.00890 | 0.2624 | 0.00848 | 0.2768 | 0.00885 | | 20 | 0.1569 | 0.00797 | 0.1507 | 0.00818 | 0.1710 | 0.00809 |
| | 40 | 0.2815 | 0.00724 | 0.2791 | 0.00690 | 0.2956 | 0.00735 | | 40 | 0.2435 | 0.00916 | 0.2428 | 0.00908 | 0.2623 | 0.00927 |
| | 60 | 0.2967 | 0.00721 | 0.2945 | 0.00709 | 0.3121 | 0.00750 | | 60 | 0.2755 | 0.00719 | 0.2737 | 0.00725 | 0.2942 | 0.00734 |
| | 80 | 0.3141 | 0.00630 | 0.3117 | 0.00640 | 0.3326 | 0.00640 | | 80 | 0.3085 | 0.00688 | 0.3067 | 0.00677 | 0.3278 | 0.00713 |
| | 100 | 0.3184 | 0.00655 | 0.3163 | 0.00660 | 0.3390 | 0.00686 | | 100 | 0.3184 | 0.00655 | 0.3163 | 0.00660 | 0.3390 | 0.00686 |
| CDtarg | 10 | 0.2029 | 0.00742 | 0.2063 | 0.00700 | 0.2141 | 0.00736 | Avg_GRMtarg | 10 | 0.1415 | 0.01169 | 0.1386 | 0.01137 | 0.1492 | 0.01166 |
| | 20 | 0.2620 | 0.00642 | 0.2608 | 0.00591 | 0.2751 | 0.00657 | | 20 | 0.1996 | 0.01067 | 0.1969 | 0.01034 | 0.2106 | 0.01088 |
| | 40 | 0.3048 | 0.00721 | 0.3029 | 0.00737 | 0.3191 | 0.00719 | | 40 | 0.2795 | 0.00666 | 0.2771 | 0.00691 | 0.2945 | 0.00684 |
| | 60 | 0.3156 | 0.00699 | 0.3135 | 0.00695 | 0.3333 | 0.00731 | | 60 | 0.2958 | 0.00735 | 0.2936 | 0.00725 | 0.3131 | 0.00737 |
| | 80 | 0.3140 | 0.00678 | 0.3130 | 0.00674 | 0.3328 | 0.00707 | | 80 | 0.3146 | 0.00683 | 0.3132 | 0.00693 | 0.3333 | 0.00698 |
| | 100 | 0.3184 | 0.00655 | 0.3163 | 0.00660 | 0.3390 | 0.00686 | | 100 | 0.3184 | 0.00655 | 0.3163 | 0.00660 | 0.3390 | 0.00686 |
| OvClustCD | 10 | 0.1975 | 0.00969 | 0.1979 | 0.00926 | 0.2084 | 0.01015 | Avg_GRM_MinMax | 10 | 0.1951 | 0.01135 | 0.2004 | 0.01155 | 0.2061 | 0.01127 |
| | 20 | 0.2495 | 0.00822 | 0.2495 | 0.00807 | 0.2641 | 0.00818 | | 20 | 0.2487 | 0.00892 | 0.2466 | 0.00883 | 0.2617 | 0.00896 |
| | 40 | 0.2857 | 0.00729 | 0.2834 | 0.00738 | 0.2996 | 0.00755 | | 40 | 0.2955 | 0.00749 | 0.2960 | 0.00729 | 0.3123 | 0.00749 |
| | 60 | 0.2981 | 0.00632 | 0.2960 | 0.00638 | 0.3127 | 0.00642 | | 60 | 0.3001 | 0.00706 | 0.2976 | 0.00709 | 0.3166 | 0.00728 |
| | 80 | 0.3072 | 0.00684 | 0.3054 | 0.00672 | 0.3246 | 0.00696 | | 80 | 0.3051 | 0.00711 | 0.3036 | 0.00714 | 0.3247 | 0.00735 |
| | 100 | 0.3184 | 0.00655 | 0.3163 | 0.00660 | 0.3390 | 0.00686 | | 100 | 0.3184 | 0.00655 | 0.3163 | 0.00660 | 0.3390 | 0.00686 |
| OvClustCDtarg | 10 | 0.2148 | 0.01030 | 0.2165 | 0.00989 | 0.2260 | 0.01024 | Avg_GRM_MinMaxtarg | 10 | 0.2078 | 0.00912 | 0.2070 | 0.00941 | 0.2156 | 0.00916 |
| | 20 | 0.2692 | 0.00831 | 0.2690 | 0.00832 | 0.2823 | 0.00842 | | 20 | 0.2621 | 0.00813 | 0.2619 | 0.00808 | 0.2735 | 0.00824 |
| | 40 | 0.2952 | 0.00740 | 0.2929 | 0.00733 | 0.3106 | 0.00755 | | 40 | 0.2862 | 0.00632 | 0.2848 | 0.00613 | 0.3021 | 0.00629 |
| | 60 | 0.3072 | 0.00696 | 0.3047 | 0.00711 | 0.3247 | 0.00720 | | 60 | 0.3064 | 0.00750 | 0.3044 | 0.00752 | 0.3236 | 0.00764 |
| | 80 | 0.3140 | 0.00673 | 0.3127 | 0.00682 | 0.3332 | 0.00683 | | 80 | 0.3116 | 0.00669 | 0.3092 | 0.00671 | 0.3306 | 0.00690 |
| | 100 | 0.3184 | 0.00655 | 0.3163 | 0.00660 | 0.3390 | 0.00686 | | 100 | 0.3184 | 0.00655 | 0.3163 | 0.00660 | 0.3390 | 0.00686 |
| WIClustCD | 10 | 0.2052 | 0.00881 | 0.2129 | 0.00831 | 0.2192 | 0.00882 | Avg_GRM_self | 10 | 0.2004 | 0.01134 | 0.2040 | 0.01121 | 0.2106 | 0.01159 |
| | 20 | 0.2413 | 0.00973 | 0.2414 | 0.00988 | 0.2569 | 0.00978 | | 20 | 0.2482 | 0.00896 | 0.2487 | 0.00903 | 0.2615 | 0.00913 |
| | 40 | 0.2837 | 0.00714 | 0.2812 | 0.00738 | 0.2982 | 0.00712 | | 40 | 0.2831 | 0.00745 | 0.2804 | 0.00737 | 0.2977 | 0.00750 |
| | 60 | 0.2980 | 0.00656 | 0.2962 | 0.00666 | 0.3124 | 0.00662 | | 60 | 0.3056 | 0.00665 | 0.3039 | 0.00684 | 0.3239 | 0.00689 |
| | 80 | 0.3120 | 0.00649 | 0.3102 | 0.00643 | 0.3287 | 0.00657 | | 80 | 0.3133 | 0.00635 | 0.3109 | 0.00633 | 0.3322 | 0.00656 |
| | 100 | 0.3184 | 0.00655 | 0.3163 | 0.00660 | 0.3390 | 0.00686 | | 100 | 0.3184 | 0.00655 | 0.3163 | 0.00660 | 0.3390 | 0.00686 |
| WIClustCDtarg | 10 | 0.2154 | 0.00804 | 0.2175 | 0.00831 | 0.2286 | 0.00823 | PAM | 10 | 0.1632 | 0.00977 | 0.1734 | 0.00921 | 0.1783 | 0.00968 |
| | 20 | 0.2639 | 0.00761 | 0.2643 | 0.00702 | 0.2788 | 0.00758 | | 20 | 0.2431 | 0.00816 | 0.2452 | 0.00844 | 0.2560 | 0.00842 |
| | 40 | 0.3050 | 0.00628 | 0.3044 | 0.00625 | 0.3202 | 0.00650 | | 40 | 0.2718 | 0.00654 | 0.2707 | 0.00628 | 0.2896 | 0.00640 |
| | 60 | 0.3118 | 0.00680 | 0.3094 | 0.00689 | 0.3286 | 0.00701 | | 60 | 0.3042 | 0.00675 | 0.3024 | 0.00665 | 0.3225 | 0.00703 |
| | 80 | 0.3175 | 0.00634 | 0.3152 | 0.00640 | 0.3363 | 0.00661 | | 80 | 0.3098 | 0.00670 | 0.3081 | 0.00670 | 0.3298 | 0.00700 |
| | 100 | 0.3184 | 0.00655 | 0.3163 | 0.00660 | 0.3390 | 0.00686 | | 100 | 0.3184 | 0.00655 | 0.3163 | 0.00660 | 0.3390 | 0.00686 |

**Table S27** Average accuracy and its corresponding standard error of the mean (SEM) across the 40 iterations for all training set optimization methods, models and training set (TRS) sizes (expressed as percentage of the candidate set (CS)) for spruce dataset and the simulated trait. If ”targ” is added at the end of the name of a method, it corresponds to targeted optimization. Otherwise, untargeted optimization was performed.

**Spruce trait simulated1**

| | | Average accuracy | | | | | | | | Average accuracy | | | | | |
|---|---|---|---|---|---|---|---|---|---|---|---|---|---|---|---|
| | | GBLUP | | BayesB | | RKHS | | | | GBLUP | | BayesB | | RKHS | |
| Optimization method | TRS size (% of CS) | Mean | SEM | Mean | SEM | Mean | SEM | Optimization method | TRS size (% of CS) | Mean | SEM | Mean | SEM | Mean | SEM |
| RAND | 10 | 0.343 | 0.01204 | 0.343 | 0.01202 | 0.343 | 0.01220 | Rscore | 10 | 0.399 | 0.01050 | 0.397 | 0.01062 | 0.399 | 0.01053 |
| | 20 | 0.411 | 0.01231 | 0.409 | 0.01226 | 0.410 | 0.01232 | | 20 | 0.441 | 0.01098 | 0.441 | 0.01084 | 0.441 | 0.01097 |
| | 40 | 0.456 | 0.01078 | 0.456 | 0.01085 | 0.455 | 0.01094 | | 40 | 0.478 | 0.00836 | 0.477 | 0.00809 | 0.478 | 0.00838 |
| | 60 | 0.478 | 0.00904 | 0.476 | 0.00903 | 0.478 | 0.00906 | | 60 | 0.508 | 0.00918 | 0.507 | 0.00907 | 0.508 | 0.00918 |
| | 80 | 0.497 | 0.00941 | 0.494 | 0.00949 | 0.496 | 0.00948 | | 80 | 0.528 | 0.00813 | 0.526 | 0.00805 | 0.528 | 0.00812 |
| | 100 | 0.508 | 0.00863 | 0.505 | 0.00877 | 0.507 | 0.00854 | | 100 | 0.508 | 0.00863 | 0.505 | 0.00877 | 0.507 | 0.00854 |
| StratSamp | 10 | 0.372 | 0.00991 | 0.372 | 0.01012 | 0.370 | 0.00989 | Rscoretarg | 10 | 0.383 | 0.01279 | 0.386 | 0.01227 | 0.382 | 0.01239 |
| | 20 | 0.432 | 0.00719 | 0.432 | 0.00748 | 0.432 | 0.00708 | | 20 | 0.428 | 0.01064 | 0.428 | 0.01089 | 0.427 | 0.01055 |
| | 40 | 0.468 | 0.00700 | 0.466 | 0.00709 | 0.467 | 0.00702 | | 40 | 0.481 | 0.00989 | 0.481 | 0.00988 | 0.481 | 0.00995 |
| | 60 | 0.491 | 0.00638 | 0.489 | 0.00664 | 0.491 | 0.00623 | | 60 | 0.501 | 0.00922 | 0.500 | 0.00926 | 0.501 | 0.00921 |
| | 80 | 0.513 | 0.00677 | 0.512 | 0.00691 | 0.513 | 0.00676 | | 80 | 0.520 | 0.00893 | 0.518 | 0.00885 | 0.520 | 0.00893 |
| | 100 | 0.508 | 0.00863 | 0.505 | 0.00877 | 0.507 | 0.00854 | | 100 | 0.508 | 0.00863 | 0.505 | 0.00877 | 0.507 | 0.00854 |
| CD | 10 | 0.368 | 0.01271 | 0.370 | 0.01269 | 0.368 | 0.01253 | Avg_GRM | 10 | 0.181 | 0.01376 | 0.180 | 0.01404 | 0.181 | 0.01373 |
| | 20 | 0.417 | 0.00874 | 0.416 | 0.00879 | 0.415 | 0.00869 | | 20 | 0.295 | 0.01229 | 0.298 | 0.01221 | 0.294 | 0.01221 |
| | 40 | 0.450 | 0.01010 | 0.449 | 0.01010 | 0.449 | 0.01002 | | 40 | 0.412 | 0.01201 | 0.411 | 0.01195 | 0.411 | 0.01201 |
| | 60 | 0.485 | 0.00938 | 0.483 | 0.00960 | 0.484 | 0.00954 | | 60 | 0.472 | 0.00951 | 0.471 | 0.00937 | 0.472 | 0.00960 |
| | 80 | 0.498 | 0.00905 | 0.497 | 0.00923 | 0.497 | 0.00918 | | 80 | 0.494 | 0.00874 | 0.493 | 0.00840 | 0.494 | 0.00880 |
| | 100 | 0.508 | 0.00863 | 0.505 | 0.00877 | 0.507 | 0.00854 | | 100 | 0.508 | 0.00863 | 0.505 | 0.00877 | 0.507 | 0.00854 |
| CDtarg | 10 | 0.376 | 0.01077 | 0.377 | 0.01057 | 0.376 | 0.01079 | Avg_GRMtarg | 10 | 0.274 | 0.01449 | 0.276 | 0.01395 | 0.275 | 0.01440 |
| | 20 | 0.425 | 0.01034 | 0.425 | 0.01013 | 0.425 | 0.01028 | | 20 | 0.376 | 0.00991 | 0.375 | 0.00992 | 0.375 | 0.00990 |
| | 40 | 0.470 | 0.00899 | 0.468 | 0.00881 | 0.469 | 0.00916 | | 40 | 0.445 | 0.00931 | 0.445 | 0.00917 | 0.446 | 0.00938 |
| | 60 | 0.483 | 0.00835 | 0.482 | 0.00839 | 0.482 | 0.00842 | | 60 | 0.481 | 0.00808 | 0.479 | 0.00818 | 0.481 | 0.00816 |
| | 80 | 0.499 | 0.00780 | 0.498 | 0.00780 | 0.498 | 0.00782 | | 80 | 0.501 | 0.00860 | 0.499 | 0.00868 | 0.502 | 0.00863 |
| | 100 | 0.508 | 0.00863 | 0.505 | 0.00877 | 0.507 | 0.00854 | | 100 | 0.508 | 0.00863 | 0.505 | 0.00877 | 0.507 | 0.00854 |
| OvClustCD | 10 | 0.376 | 0.01179 | 0.375 | 0.01175 | 0.374 | 0.01175 | Avg_GRM_MinMax | 10 | 0.360 | 0.00816 | 0.359 | 0.00842 | 0.360 | 0.00811 |
| | 20 | 0.413 | 0.00951 | 0.412 | 0.00932 | 0.411 | 0.00946 | | 20 | 0.411 | 0.01024 | 0.411 | 0.01001 | 0.411 | 0.01041 |
| | 40 | 0.459 | 0.00957 | 0.457 | 0.00949 | 0.458 | 0.00963 | | 40 | 0.448 | 0.00977 | 0.447 | 0.00966 | 0.447 | 0.00979 |
| | 60 | 0.483 | 0.00846 | 0.480 | 0.00860 | 0.482 | 0.00847 | | 60 | 0.486 | 0.00862 | 0.484 | 0.00854 | 0.485 | 0.00878 |
| | 80 | 0.502 | 0.00921 | 0.501 | 0.00913 | 0.502 | 0.00917 | | 80 | 0.500 | 0.00826 | 0.499 | 0.00825 | 0.500 | 0.00827 |
| | 100 | 0.508 | 0.00863 | 0.505 | 0.00877 | 0.507 | 0.00854 | | 100 | 0.508 | 0.00863 | 0.505 | 0.00877 | 0.507 | 0.00854 |
| OvClustCDtarg | 10 | 0.387 | 0.01173 | 0.388 | 0.01142 | 0.387 | 0.01159 | Avg_GRM_MinMaxtarg | 10 | 0.365 | 0.00972 | 0.364 | 0.01006 | 0.365 | 0.00976 |
| | 20 | 0.441 | 0.00868 | 0.441 | 0.00888 | 0.440 | 0.00868 | | 20 | 0.418 | 0.00805 | 0.418 | 0.00796 | 0.417 | 0.00821 |
| | 40 | 0.481 | 0.00819 | 0.480 | 0.00830 | 0.480 | 0.00816 | | 40 | 0.460 | 0.00852 | 0.460 | 0.00854 | 0.459 | 0.00863 |
| | 60 | 0.495 | 0.00870 | 0.494 | 0.00873 | 0.495 | 0.00872 | | 60 | 0.486 | 0.00813 | 0.485 | 0.00801 | 0.486 | 0.00819 |
| | 80 | 0.505 | 0.00816 | 0.504 | 0.00812 | 0.505 | 0.00810 | | 80 | 0.500 | 0.00818 | 0.498 | 0.00826 | 0.500 | 0.00826 |
| | 100 | 0.508 | 0.00863 | 0.505 | 0.00877 | 0.507 | 0.00854 | | 100 | 0.508 | 0.00863 | 0.505 | 0.00877 | 0.507 | 0.00854 |
| WIClustCD | 10 | 0.366 | 0.01172 | 0.368 | 0.01128 | 0.366 | 0.01176 | Avg_GRM_self | 10 | 0.352 | 0.01077 | 0.355 | 0.01050 | 0.352 | 0.01079 |
| | 20 | 0.412 | 0.00937 | 0.413 | 0.00930 | 0.412 | 0.00933 | | 20 | 0.415 | 0.00901 | 0.415 | 0.00897 | 0.415 | 0.00903 |
| | 40 | 0.456 | 0.00926 | 0.454 | 0.00914 | 0.455 | 0.00922 | | 40 | 0.458 | 0.00918 | 0.456 | 0.00935 | 0.457 | 0.00932 |
| | 60 | 0.480 | 0.00935 | 0.478 | 0.00918 | 0.479 | 0.00947 | | 60 | 0.483 | 0.00889 | 0.481 | 0.00906 | 0.483 | 0.00891 |
| | 80 | 0.498 | 0.00864 | 0.495 | 0.00866 | 0.498 | 0.00867 | | 80 | 0.504 | 0.00840 | 0.502 | 0.00824 | 0.504 | 0.00848 |
| | 100 | 0.508 | 0.00863 | 0.505 | 0.00877 | 0.507 | 0.00854 | | 100 | 0.508 | 0.00863 | 0.505 | 0.00877 | 0.507 | 0.00854 |
| WIClustCDtarg | 10 | 0.382 | 0.00885 | 0.381 | 0.00865 | 0.382 | 0.00888 | PAM | 10 | 0.382 | 0.01212 | 0.383 | 0.01192 | 0.380 | 0.01195 |
| | 20 | 0.431 | 0.00864 | 0.432 | 0.00861 | 0.430 | 0.00864 | | 20 | 0.427 | 0.01169 | 0.427 | 0.01172 | 0.426 | 0.01167 |
| | 40 | 0.470 | 0.00842 | 0.469 | 0.00851 | 0.470 | 0.00841 | | 40 | 0.474 | 0.00897 | 0.472 | 0.00918 | 0.473 | 0.00890 |
| | 60 | 0.492 | 0.00799 | 0.491 | 0.00804 | 0.491 | 0.00807 | | 60 | 0.495 | 0.00853 | 0.493 | 0.00892 | 0.495 | 0.00861 |
| | 80 | 0.506 | 0.00843 | 0.504 | 0.00852 | 0.506 | 0.00850 | | 80 | 0.509 | 0.00867 | 0.508 | 0.00870 | 0.510 | 0.00872 |
| | 100 | 0.508 | 0.00863 | 0.505 | 0.00877 | 0.507 | 0.00854 | | 100 | 0.508 | 0.00863 | 0.505 | 0.00877 | 0.507 | 0.00854 |

**Table S28** Average accuracy and its corresponding standard error of the mean (SEM) across the 40 iterations for all training set optimization methods, models and training set (TRS) sizes (expressed as percentage of the candidate set (CS)) for switchgrass dataset and AN trait. If "targ" is added at the end of the name of a method, it corresponds to targeted optimization. Otherwise, untargeted optimization was performed.

**Switchgrass trait AN**

| | | Average accuracy | | | | | | | | Average accuracy | | | | | |
|---|---|---|---|---|---|---|---|---|---|---|---|---|---|---|---|
| | | GBLUP | | BayesB | | RKHS | | | | GBLUP | | BayesB | | RKHS | |
| Optimization method | TRS size (% of CS) | Mean | SEM | Mean | SEM | Mean | SEM | Optimization method | TRS size (% of CS) | Mean | SEM | Mean | SEM | Mean | SEM |
| RAND | 10 | 0.741 | 0.01366 | 0.728 | 0.01334 | 0.741 | 0.01364 | Rscore | 10 | 0.739 | 0.01207 | 0.722 | 0.01219 | 0.739 | 0.01207 |
| | 20 | 0.785 | 0.01375 | 0.770 | 0.01356 | 0.785 | 0.01374 | | 20 | 0.796 | 0.00984 | 0.777 | 0.01055 | 0.796 | 0.00985 |
| | 40 | 0.828 | 0.01103 | 0.816 | 0.01137 | 0.828 | 0.01105 | | 40 | 0.833 | 0.01059 | 0.822 | 0.01082 | 0.833 | 0.01059 |
| | 60 | 0.856 | 0.00913 | 0.849 | 0.00917 | 0.856 | 0.00914 | | 60 | 0.851 | 0.00971 | 0.843 | 0.01004 | 0.851 | 0.00973 |
| | 80 | 0.866 | 0.00916 | 0.862 | 0.00928 | 0.866 | 0.00918 | | 80 | 0.868 | 0.00901 | 0.863 | 0.00920 | 0.868 | 0.00903 |
| | 100 | 0.871 | 0.00887 | 0.868 | 0.00920 | 0.871 | 0.00888 | | 100 | 0.871 | 0.00887 | 0.868 | 0.00920 | 0.871 | 0.00888 |
| StratSamp | 10 | 0.742 | 0.01222 | 0.723 | 0.01255 | 0.741 | 0.01226 | Rscoretarg | 10 | 0.756 | 0.01080 | 0.742 | 0.01067 | 0.756 | 0.01079 |
| | 20 | 0.789 | 0.01225 | 0.774 | 0.01231 | 0.789 | 0.01224 | | 20 | 0.797 | 0.01193 | 0.788 | 0.01189 | 0.797 | 0.01193 |
| | 40 | 0.828 | 0.01057 | 0.816 | 0.01075 | 0.828 | 0.01058 | | 40 | 0.847 | 0.01034 | 0.839 | 0.01024 | 0.846 | 0.01034 |
| | 60 | 0.856 | 0.00987 | 0.848 | 0.01004 | 0.856 | 0.00987 | | 60 | 0.861 | 0.00906 | 0.855 | 0.00911 | 0.861 | 0.00906 |
| | 80 | 0.865 | 0.00873 | 0.860 | 0.00895 | 0.865 | 0.00875 | | 80 | 0.871 | 0.00896 | 0.867 | 0.00896 | 0.871 | 0.00897 |
| | 100 | 0.871 | 0.00887 | 0.868 | 0.00920 | 0.871 | 0.00888 | | 100 | 0.871 | 0.00887 | 0.868 | 0.00920 | 0.871 | 0.00888 |
| CD | 10 | 0.744 | 0.01245 | 0.731 | 0.01268 | 0.744 | 0.01246 | Avg_GRM | 10 | 0.238 | 0.03782 | 0.246 | 0.03707 | 0.237 | 0.03791 |
| | 20 | 0.802 | 0.01176 | 0.785 | 0.01203 | 0.801 | 0.01175 | | 20 | 0.312 | 0.03298 | 0.317 | 0.03318 | 0.312 | 0.03301 |
| | 40 | 0.852 | 0.00997 | 0.843 | 0.01004 | 0.852 | 0.00998 | | 40 | 0.510 | 0.02451 | 0.514 | 0.02380 | 0.511 | 0.02442 |
| | 60 | 0.854 | 0.00932 | 0.848 | 0.00935 | 0.854 | 0.00933 | | 60 | 0.651 | 0.02092 | 0.652 | 0.01966 | 0.651 | 0.02078 |
| | 80 | 0.862 | 0.00918 | 0.861 | 0.00902 | 0.862 | 0.00919 | | 80 | 0.808 | 0.01341 | 0.802 | 0.01353 | 0.808 | 0.01335 |
| | 100 | 0.871 | 0.00887 | 0.868 | 0.00920 | 0.871 | 0.00888 | | 100 | 0.871 | 0.00887 | 0.868 | 0.00920 | 0.871 | 0.00888 |
| CDtarg | 10 | 0.726 | 0.01300 | 0.722 | 0.01295 | 0.726 | 0.01300 | Avg_GRMtarg | 10 | 0.408 | 0.02652 | 0.419 | 0.02632 | 0.408 | 0.02662 |
| | 20 | 0.777 | 0.00983 | 0.773 | 0.01001 | 0.777 | 0.00984 | | 20 | 0.544 | 0.02511 | 0.544 | 0.02401 | 0.544 | 0.02490 |
| | 40 | 0.844 | 0.00913 | 0.836 | 0.00933 | 0.844 | 0.00914 | | 40 | 0.679 | 0.02064 | 0.683 | 0.01970 | 0.679 | 0.02056 |
| | 60 | 0.866 | 0.00858 | 0.859 | 0.00886 | 0.865 | 0.00859 | | 60 | 0.798 | 0.01523 | 0.795 | 0.01440 | 0.798 | 0.01513 |
| | 80 | 0.870 | 0.00867 | 0.865 | 0.00890 | 0.870 | 0.00868 | | 80 | 0.866 | 0.00910 | 0.863 | 0.00903 | 0.866 | 0.00911 |
| | 100 | 0.871 | 0.00887 | 0.868 | 0.00920 | 0.871 | 0.00888 | | 100 | 0.871 | 0.00887 | 0.868 | 0.00920 | 0.871 | 0.00888 |
| OvClustCD | 10 | 0.740 | 0.01239 | 0.732 | 0.01264 | 0.740 | 0.01240 | Avg_GRM_MinMax | 10 | 0.771 | 0.01093 | 0.752 | 0.01100 | 0.771 | 0.01093 |
| | 20 | 0.803 | 0.01154 | 0.788 | 0.01146 | 0.803 | 0.01154 | | 20 | 0.810 | 0.00943 | 0.793 | 0.00986 | 0.809 | 0.00944 |
| | 40 | 0.845 | 0.01004 | 0.835 | 0.01033 | 0.844 | 0.01006 | | 40 | 0.848 | 0.00960 | 0.837 | 0.00964 | 0.848 | 0.00962 |
| | 60 | 0.863 | 0.00920 | 0.857 | 0.00943 | 0.863 | 0.00922 | | 60 | 0.861 | 0.00880 | 0.855 | 0.00889 | 0.861 | 0.00883 |
| | 80 | 0.870 | 0.00906 | 0.866 | 0.00929 | 0.870 | 0.00908 | | 80 | 0.869 | 0.00882 | 0.865 | 0.00887 | 0.869 | 0.00883 |
| | 100 | 0.871 | 0.00887 | 0.868 | 0.00920 | 0.871 | 0.00888 | | 100 | 0.871 | 0.00887 | 0.868 | 0.00920 | 0.871 | 0.00888 |
| OvClustCDtarg | 10 | 0.750 | 0.01024 | 0.745 | 0.01032 | 0.750 | 0.01023 | Avg_GRM_MinMaxtarg | 10 | 0.794 | 0.01059 | 0.773 | 0.01095 | 0.793 | 0.01060 |
| | 20 | 0.806 | 0.00870 | 0.800 | 0.00885 | 0.806 | 0.00870 | | 20 | 0.837 | 0.00912 | 0.820 | 0.00949 | 0.836 | 0.00913 |
| | 40 | 0.855 | 0.00878 | 0.847 | 0.00907 | 0.855 | 0.00878 | | 40 | 0.858 | 0.00919 | 0.849 | 0.00936 | 0.858 | 0.00921 |
| | 60 | 0.867 | 0.00867 | 0.863 | 0.00858 | 0.867 | 0.00867 | | 60 | 0.869 | 0.00895 | 0.864 | 0.00912 | 0.869 | 0.00897 |
| | 80 | 0.871 | 0.00871 | 0.868 | 0.00872 | 0.871 | 0.00872 | | 80 | 0.871 | 0.00880 | 0.866 | 0.00890 | 0.871 | 0.00882 |
| | 100 | 0.871 | 0.00887 | 0.868 | 0.00920 | 0.871 | 0.00888 | | 100 | 0.871 | 0.00887 | 0.868 | 0.00920 | 0.871 | 0.00888 |
| WIClustCD | 10 | 0.737 | 0.01195 | 0.726 | 0.01224 | 0.737 | 0.01196 | Avg_GRM_self | 10 | 0.779 | 0.01335 | 0.760 | 0.01320 | 0.778 | 0.01334 |
| | 20 | 0.816 | 0.01035 | 0.796 | 0.01071 | 0.816 | 0.01036 | | 20 | 0.813 | 0.01012 | 0.795 | 0.01033 | 0.812 | 0.01013 |
| | 40 | 0.848 | 0.00993 | 0.838 | 0.01013 | 0.848 | 0.00995 | | 40 | 0.845 | 0.00871 | 0.835 | 0.00888 | 0.845 | 0.00873 |
| | 60 | 0.863 | 0.00956 | 0.856 | 0.00944 | 0.863 | 0.00958 | | 60 | 0.864 | 0.00906 | 0.858 | 0.00924 | 0.864 | 0.00908 |
| | 80 | 0.869 | 0.00918 | 0.865 | 0.00930 | 0.869 | 0.00920 | | 80 | 0.870 | 0.00893 | 0.866 | 0.00907 | 0.870 | 0.00895 |
| | 100 | 0.871 | 0.00887 | 0.868 | 0.00920 | 0.871 | 0.00888 | | 100 | 0.871 | 0.00887 | 0.868 | 0.00920 | 0.871 | 0.00888 |
| WIClustCDtarg | 10 | 0.761 | 0.01087 | 0.755 | 0.01117 | 0.761 | 0.01087 | PAM | 10 | 0.778 | 0.01279 | 0.745 | 0.01278 | 0.777 | 0.01280 |
| | 20 | 0.814 | 0.00907 | 0.807 | 0.00921 | 0.814 | 0.00906 | | 20 | 0.816 | 0.01022 | 0.786 | 0.01098 | 0.816 | 0.01025 |
| | 40 | 0.861 | 0.00867 | 0.854 | 0.00885 | 0.860 | 0.00868 | | 40 | 0.827 | 0.00947 | 0.803 | 0.01014 | 0.826 | 0.00951 |
| | 60 | 0.869 | 0.00854 | 0.864 | 0.00871 | 0.868 | 0.00855 | | 60 | 0.843 | 0.00912 | 0.824 | 0.00983 | 0.843 | 0.00915 |
| | 80 | 0.872 | 0.00863 | 0.868 | 0.00898 | 0.872 | 0.00864 | | 80 | 0.868 | 0.00890 | 0.861 | 0.00915 | 0.868 | 0.00894 |
| | 100 | 0.871 | 0.00887 | 0.868 | 0.00920 | 0.871 | 0.00888 | | 100 | 0.871 | 0.00887 | 0.868 | 0.00920 | 0.871 | 0.00888 |

**Table S29** Average accuracy and its corresponding standard error of the mean (SEM) across the 40 iterations for all training set optimization methods, models and training set (TRS) sizes (expressed as percentage of the candidate set (CS)) for switchgrass dataset and HT trait. If "targ" is added at the end of the name of a method, it corresponds to targeted optimization. Otherwise, untargeted optimization was performed.

**Switchgrass trait HT**

| Optimization method | TRS size (% of CS) | Average accuracy | | | | | | Optimization method | TRS size (% of CS) | Average accuracy | | | | | |
|---|---|---|---|---|---|---|---|---|---|---|---|---|---|---|---|
| | | GBLUP | | BayesB | | RKHS | | | | GBLUP | | BayesB | | RKHS | |
| | | Mean | SEM | Mean | SEM | Mean | SEM | | | Mean | SEM | Mean | SEM | Mean | SEM |
| RAND | 10 | 0.546 | 0.0133 | 0.546 | 0.01187 | 0.546 | 0.0133 | Rscore | 10 | 0.550 | 0.0140 | 0.549 | 0.01241 | 0.550 | 0.0140 |
| | 20 | 0.587 | 0.0141 | 0.588 | 0.01341 | 0.586 | 0.0141 | | 20 | 0.590 | 0.0124 | 0.590 | 0.01217 | 0.590 | 0.0124 |
| | 40 | 0.609 | 0.0108 | 0.610 | 0.01058 | 0.609 | 0.0108 | | 40 | 0.616 | 0.0096 | 0.620 | 0.00959 | 0.616 | 0.0096 |
| | 60 | 0.627 | 0.0103 | 0.629 | 0.01005 | 0.627 | 0.0104 | | 60 | 0.624 | 0.0102 | 0.626 | 0.01025 | 0.624 | 0.0103 |
| | 80 | 0.631 | 0.0107 | 0.634 | 0.01040 | 0.630 | 0.0107 | | 80 | 0.634 | 0.0112 | 0.636 | 0.01103 | 0.634 | 0.0113 |
| | 100 | 0.636 | 0.0111 | 0.638 | 0.01081 | 0.636 | 0.0112 | | 100 | 0.636 | 0.0111 | 0.638 | 0.01081 | 0.636 | 0.0112 |
| StratSamp | 10 | 0.544 | 0.0141 | 0.543 | 0.01394 | 0.544 | 0.0142 | Rscoretarg | 10 | 0.557 | 0.0156 | 0.563 | 0.01500 | 0.557 | 0.0156 |
| | 20 | 0.581 | 0.0103 | 0.583 | 0.01026 | 0.581 | 0.0103 | | 20 | 0.576 | 0.0129 | 0.580 | 0.01264 | 0.576 | 0.0130 |
| | 40 | 0.609 | 0.0108 | 0.610 | 0.01045 | 0.609 | 0.0108 | | 40 | 0.612 | 0.0125 | 0.615 | 0.01211 | 0.612 | 0.0125 |
| | 60 | 0.620 | 0.0111 | 0.623 | 0.01070 | 0.620 | 0.0111 | | 60 | 0.626 | 0.0119 | 0.630 | 0.01119 | 0.626 | 0.0120 |
| | 80 | 0.630 | 0.0118 | 0.632 | 0.01120 | 0.629 | 0.0118 | | 80 | 0.632 | 0.0113 | 0.634 | 0.01111 | 0.632 | 0.0113 |
| | 100 | 0.636 | 0.0111 | 0.638 | 0.01081 | 0.636 | 0.0112 | | 100 | 0.636 | 0.0111 | 0.638 | 0.01081 | 0.636 | 0.0112 |
| CD | 10 | 0.550 | 0.0134 | 0.553 | 0.01305 | 0.550 | 0.0134 | Avg_GRM | 10 | 0.211 | 0.0347 | 0.206 | 0.03591 | 0.199 | 0.0346 |
| | 20 | 0.597 | 0.0102 | 0.597 | 0.01016 | 0.597 | 0.0102 | | 20 | 0.276 | 0.0289 | 0.262 | 0.03216 | 0.265 | 0.0291 |
| | 40 | 0.612 | 0.0119 | 0.614 | 0.01128 | 0.611 | 0.0119 | | 40 | 0.407 | 0.0240 | 0.408 | 0.02332 | 0.404 | 0.0238 |
| | 60 | 0.615 | 0.0121 | 0.618 | 0.01138 | 0.614 | 0.0120 | | 60 | 0.501 | 0.0157 | 0.498 | 0.01631 | 0.497 | 0.0158 |
| | 80 | 0.629 | 0.0114 | 0.630 | 0.01146 | 0.629 | 0.0115 | | 80 | 0.577 | 0.0158 | 0.578 | 0.01594 | 0.576 | 0.0159 |
| | 100 | 0.636 | 0.0111 | 0.638 | 0.01081 | 0.636 | 0.0112 | | 100 | 0.636 | 0.0111 | 0.638 | 0.01081 | 0.636 | 0.0112 |
| CDtarg | 10 | 0.543 | 0.0154 | 0.552 | 0.01409 | 0.543 | 0.0154 | Avg_GRMtarg | 10 | 0.296 | 0.0334 | 0.298 | 0.03414 | 0.292 | 0.0340 |
| | 20 | 0.587 | 0.0131 | 0.593 | 0.01227 | 0.586 | 0.0131 | | 20 | 0.447 | 0.0299 | 0.448 | 0.03074 | 0.443 | 0.0310 |
| | 40 | 0.621 | 0.0119 | 0.623 | 0.01157 | 0.621 | 0.0119 | | 40 | 0.493 | 0.0264 | 0.493 | 0.02726 | 0.490 | 0.0265 |
| | 60 | 0.631 | 0.0116 | 0.632 | 0.01133 | 0.630 | 0.0117 | | 60 | 0.541 | 0.0219 | 0.544 | 0.02240 | 0.539 | 0.0221 |
| | 80 | 0.634 | 0.0113 | 0.637 | 0.01096 | 0.633 | 0.0114 | | 80 | 0.614 | 0.0135 | 0.616 | 0.01355 | 0.613 | 0.0136 |
| | 100 | 0.636 | 0.0111 | 0.638 | 0.01081 | 0.636 | 0.0112 | | 100 | 0.636 | 0.0111 | 0.638 | 0.01081 | 0.636 | 0.0112 |
| OvClustCD | 10 | 0.524 | 0.0179 | 0.526 | 0.01626 | 0.523 | 0.0179 | Avg_GRM_MinMax | 10 | 0.550 | 0.0152 | 0.553 | 0.01468 | 0.550 | 0.0150 |
| | 20 | 0.584 | 0.0117 | 0.585 | 0.01167 | 0.584 | 0.0117 | | 20 | 0.581 | 0.0137 | 0.587 | 0.01303 | 0.581 | 0.0136 |
| | 40 | 0.606 | 0.0109 | 0.609 | 0.01094 | 0.606 | 0.0109 | | 40 | 0.611 | 0.0107 | 0.613 | 0.01014 | 0.611 | 0.0107 |
| | 60 | 0.614 | 0.0109 | 0.619 | 0.01071 | 0.614 | 0.0110 | | 60 | 0.621 | 0.0112 | 0.624 | 0.01060 | 0.621 | 0.0113 |
| | 80 | 0.629 | 0.0107 | 0.632 | 0.01041 | 0.629 | 0.0108 | | 80 | 0.628 | 0.0117 | 0.631 | 0.01106 | 0.628 | 0.0117 |
| | 100 | 0.636 | 0.0111 | 0.638 | 0.01081 | 0.636 | 0.0112 | | 100 | 0.636 | 0.0111 | 0.638 | 0.01081 | 0.636 | 0.0112 |
| OvClustCDtarg | 10 | 0.530 | 0.0171 | 0.536 | 0.01632 | 0.530 | 0.0173 | Avg_GRM_MinMaxtarg | 10 | 0.561 | 0.0142 | 0.562 | 0.01500 | 0.561 | 0.0142 |
| | 20 | 0.593 | 0.0124 | 0.596 | 0.01176 | 0.593 | 0.0124 | | 20 | 0.582 | 0.0137 | 0.591 | 0.01273 | 0.582 | 0.0137 |
| | 40 | 0.623 | 0.0121 | 0.625 | 0.01158 | 0.623 | 0.0121 | | 40 | 0.614 | 0.0119 | 0.618 | 0.01150 | 0.613 | 0.0119 |
| | 60 | 0.630 | 0.0117 | 0.633 | 0.01130 | 0.630 | 0.0117 | | 60 | 0.629 | 0.0113 | 0.633 | 0.01090 | 0.629 | 0.0113 |
| | 80 | 0.634 | 0.0112 | 0.637 | 0.01103 | 0.634 | 0.0113 | | 80 | 0.633 | 0.0113 | 0.637 | 0.01091 | 0.633 | 0.0113 |
| | 100 | 0.636 | 0.0111 | 0.638 | 0.01081 | 0.636 | 0.0112 | | 100 | 0.636 | 0.0111 | 0.638 | 0.01081 | 0.636 | 0.0112 |
| WIClustCD | 10 | 0.537 | 0.0113 | 0.541 | 0.01066 | 0.537 | 0.0113 | Avg_GRM_self | 10 | 0.566 | 0.0133 | 0.566 | 0.01362 | 0.566 | 0.0133 |
| | 20 | 0.587 | 0.0104 | 0.588 | 0.01065 | 0.587 | 0.0104 | | 20 | 0.583 | 0.0127 | 0.590 | 0.01220 | 0.583 | 0.0127 |
| | 40 | 0.609 | 0.0104 | 0.610 | 0.01020 | 0.609 | 0.0105 | | 40 | 0.608 | 0.0106 | 0.612 | 0.01034 | 0.608 | 0.0106 |
| | 60 | 0.617 | 0.0107 | 0.621 | 0.01034 | 0.617 | 0.0108 | | 60 | 0.628 | 0.0111 | 0.630 | 0.01054 | 0.628 | 0.0111 |
| | 80 | 0.628 | 0.0107 | 0.631 | 0.01038 | 0.627 | 0.0108 | | 80 | 0.632 | 0.0112 | 0.636 | 0.01058 | 0.633 | 0.0111 |
| | 100 | 0.636 | 0.0111 | 0.638 | 0.01081 | 0.636 | 0.0112 | | 100 | 0.636 | 0.0111 | 0.638 | 0.01081 | 0.636 | 0.0112 |
| WIClustCDtarg | 10 | 0.536 | 0.0160 | 0.541 | 0.01480 | 0.535 | 0.0161 | PAM | 10 | 0.577 | 0.0119 | 0.577 | 0.01132 | 0.577 | 0.0119 |
| | 20 | 0.590 | 0.0123 | 0.593 | 0.01183 | 0.590 | 0.0123 | | 20 | 0.610 | 0.0122 | 0.605 | 0.01198 | 0.610 | 0.0122 |
| | 40 | 0.622 | 0.0128 | 0.625 | 0.01224 | 0.622 | 0.0128 | | 40 | 0.612 | 0.0115 | 0.608 | 0.01137 | 0.612 | 0.0115 |
| | 60 | 0.630 | 0.0119 | 0.633 | 0.01158 | 0.630 | 0.0119 | | 60 | 0.617 | 0.0113 | 0.617 | 0.01125 | 0.617 | 0.0113 |
| | 80 | 0.635 | 0.0112 | 0.637 | 0.01093 | 0.634 | 0.0113 | | 80 | 0.632 | 0.0109 | 0.635 | 0.01056 | 0.632 | 0.0110 |
| | 100 | 0.636 | 0.0111 | 0.638 | 0.01081 | 0.636 | 0.0112 | | 100 | 0.636 | 0.0111 | 0.638 | 0.01081 | 0.636 | 0.0112 |

**Table S30** Average accuracy and its corresponding standard error of the mean (SEM) across the 40 iterations for all training set optimization methods, models and training set (TRS) sizes (expressed as percentage of the candidate set (CS)) for switchgrass dataset and ST trait. If ”targ” is added at the end of the name of a method, it corresponds to targeted optimization. Otherwise, untargeted optimization was performed.

**Switchgrass trait ST**

| Optimization method | TRS size (% of CS) | Average accuracy | | | | | | Optimization method | TRS size (% of CS) | Average accuracy | | | | | |
|---|---|---|---|---|---|---|---|---|---|---|---|---|---|---|---|
| | | GBLUP | | BayesB | | RKHS | | | | GBLUP | | BayesB | | RKHS | |
| | | Mean | SEM | Mean | SEM | Mean | SEM | | | Mean | SEM | Mean | SEM | Mean | SEM |
| RAND | 10 | 0.622 | 0.01007 | 0.617 | 0.01060 | 0.622 | 0.01012 | Rscore | 10 | 0.624 | 0.01175 | 0.616 | 0.01182 | 0.624 | 0.01172 |
| | 20 | 0.638 | 0.01135 | 0.637 | 0.01177 | 0.638 | 0.01134 | | 20 | 0.655 | 0.00897 | 0.652 | 0.00936 | 0.655 | 0.00897 |
| | 40 | 0.678 | 0.00868 | 0.676 | 0.00903 | 0.678 | 0.00869 | | 40 | 0.671 | 0.00871 | 0.671 | 0.00908 | 0.671 | 0.00871 |
| | 60 | 0.689 | 0.00869 | 0.688 | 0.00879 | 0.689 | 0.00871 | | 60 | 0.693 | 0.00814 | 0.691 | 0.00844 | 0.693 | 0.00816 |
| | 80 | 0.702 | 0.00797 | 0.700 | 0.00830 | 0.702 | 0.00798 | | 80 | 0.699 | 0.00848 | 0.700 | 0.00835 | 0.699 | 0.00846 |
| | 100 | 0.706 | 0.00763 | 0.707 | 0.00781 | 0.706 | 0.00764 | | 100 | 0.706 | 0.00763 | 0.707 | 0.00781 | 0.706 | 0.00764 |
| StratSamp | 10 | 0.615 | 0.01173 | 0.612 | 0.01150 | 0.615 | 0.01167 | Rscoretarg | 10 | 0.643 | 0.01048 | 0.639 | 0.01063 | 0.643 | 0.01048 |
| | 20 | 0.647 | 0.00973 | 0.646 | 0.01016 | 0.647 | 0.00972 | | 20 | 0.673 | 0.00860 | 0.671 | 0.00893 | 0.673 | 0.00861 |
| | 40 | 0.681 | 0.00807 | 0.678 | 0.00842 | 0.682 | 0.00805 | | 40 | 0.689 | 0.00876 | 0.689 | 0.00868 | 0.689 | 0.00876 |
| | 60 | 0.690 | 0.00838 | 0.688 | 0.00880 | 0.690 | 0.00840 | | 60 | 0.698 | 0.00844 | 0.697 | 0.00867 | 0.699 | 0.00844 |
| | 80 | 0.701 | 0.00771 | 0.700 | 0.00811 | 0.701 | 0.00771 | | 80 | 0.705 | 0.00756 | 0.705 | 0.00793 | 0.705 | 0.00757 |
| | 100 | 0.706 | 0.00763 | 0.707 | 0.00781 | 0.706 | 0.00764 | | 100 | 0.706 | 0.00763 | 0.707 | 0.00781 | 0.706 | 0.00764 |
| CD | 10 | 0.623 | 0.01002 | 0.619 | 0.01026 | 0.623 | 0.01002 | Avg_GRM | 10 | 0.146 | 0.03071 | 0.136 | 0.03158 | 0.138 | 0.03014 |
| | 20 | 0.654 | 0.00896 | 0.651 | 0.00944 | 0.654 | 0.00894 | | 20 | 0.172 | 0.03453 | 0.195 | 0.03085 | 0.167 | 0.03422 |
| | 40 | 0.674 | 0.00845 | 0.669 | 0.00926 | 0.674 | 0.00850 | | 40 | 0.434 | 0.02411 | 0.422 | 0.02406 | 0.431 | 0.02431 |
| | 60 | 0.683 | 0.00868 | 0.679 | 0.00891 | 0.683 | 0.00871 | | 60 | 0.571 | 0.01586 | 0.562 | 0.01727 | 0.570 | 0.01606 |
| | 80 | 0.696 | 0.00781 | 0.695 | 0.00807 | 0.696 | 0.00782 | | 80 | 0.663 | 0.00843 | 0.658 | 0.00877 | 0.663 | 0.00839 |
| | 100 | 0.706 | 0.00763 | 0.707 | 0.00781 | 0.706 | 0.00764 | | 100 | 0.706 | 0.00763 | 0.707 | 0.00781 | 0.706 | 0.00764 |
| CDtarg | 10 | 0.638 | 0.01069 | 0.633 | 0.01120 | 0.637 | 0.01073 | Avg_GRMtarg | 10 | 0.264 | 0.03123 | 0.289 | 0.03002 | 0.269 | 0.03000 |
| | 20 | 0.678 | 0.00919 | 0.676 | 0.00957 | 0.678 | 0.00919 | | 20 | 0.300 | 0.03240 | 0.315 | 0.03019 | 0.302 | 0.03157 |
| | 40 | 0.697 | 0.00829 | 0.697 | 0.00857 | 0.697 | 0.00830 | | 40 | 0.559 | 0.01860 | 0.552 | 0.01867 | 0.556 | 0.01893 |
| | 60 | 0.702 | 0.00803 | 0.702 | 0.00835 | 0.702 | 0.00807 | | 60 | 0.639 | 0.01660 | 0.639 | 0.01586 | 0.639 | 0.01670 |
| | 80 | 0.703 | 0.00790 | 0.704 | 0.00798 | 0.703 | 0.00792 | | 80 | 0.700 | 0.00815 | 0.700 | 0.00837 | 0.700 | 0.00819 |
| | 100 | 0.706 | 0.00763 | 0.707 | 0.00781 | 0.706 | 0.00764 | | 100 | 0.706 | 0.00763 | 0.707 | 0.00781 | 0.706 | 0.00764 |
| OvClustCD | 10 | 0.618 | 0.01003 | 0.616 | 0.01045 | 0.618 | 0.01005 | Avg_GRM_MinMax | 10 | 0.638 | 0.00941 | 0.635 | 0.01005 | 0.638 | 0.00942 |
| | 20 | 0.652 | 0.00833 | 0.648 | 0.00878 | 0.652 | 0.00833 | | 20 | 0.664 | 0.01027 | 0.662 | 0.01067 | 0.664 | 0.01027 |
| | 40 | 0.676 | 0.00797 | 0.674 | 0.00827 | 0.676 | 0.00797 | | 40 | 0.684 | 0.00857 | 0.684 | 0.00877 | 0.684 | 0.00857 |
| | 60 | 0.692 | 0.00799 | 0.690 | 0.00838 | 0.692 | 0.00801 | | 60 | 0.699 | 0.00768 | 0.698 | 0.00802 | 0.700 | 0.00769 |
| | 80 | 0.699 | 0.00782 | 0.700 | 0.00800 | 0.699 | 0.00785 | | 80 | 0.703 | 0.00744 | 0.703 | 0.00789 | 0.703 | 0.00748 |
| | 100 | 0.706 | 0.00763 | 0.707 | 0.00781 | 0.706 | 0.00764 | | 100 | 0.706 | 0.00763 | 0.707 | 0.00781 | 0.706 | 0.00764 |
| OvClustCDtarg | 10 | 0.652 | 0.01012 | 0.649 | 0.01028 | 0.651 | 0.01010 | Avg_GRM_MinMaxtarg | 10 | 0.651 | 0.00969 | 0.649 | 0.00946 | 0.652 | 0.00961 |
| | 20 | 0.680 | 0.00868 | 0.678 | 0.00887 | 0.680 | 0.00868 | | 20 | 0.685 | 0.00912 | 0.682 | 0.00919 | 0.685 | 0.00911 |
| | 40 | 0.696 | 0.00833 | 0.696 | 0.00840 | 0.696 | 0.00833 | | 40 | 0.697 | 0.00786 | 0.697 | 0.00836 | 0.698 | 0.00787 |
| | 60 | 0.701 | 0.00798 | 0.701 | 0.00815 | 0.701 | 0.00798 | | 60 | 0.704 | 0.00784 | 0.703 | 0.00803 | 0.704 | 0.00785 |
| | 80 | 0.705 | 0.00776 | 0.706 | 0.00799 | 0.705 | 0.00777 | | 80 | 0.705 | 0.00763 | 0.705 | 0.00785 | 0.705 | 0.00763 |
| | 100 | 0.706 | 0.00763 | 0.707 | 0.00781 | 0.706 | 0.00764 | | 100 | 0.706 | 0.00763 | 0.707 | 0.00781 | 0.706 | 0.00764 |
| WIClustCD | 10 | 0.624 | 0.01069 | 0.621 | 0.01097 | 0.623 | 0.01071 | Avg_GRM_self | 10 | 0.652 | 0.01022 | 0.647 | 0.01017 | 0.652 | 0.01022 |
| | 20 | 0.656 | 0.00858 | 0.651 | 0.00888 | 0.656 | 0.00858 | | 20 | 0.674 | 0.00997 | 0.669 | 0.01002 | 0.674 | 0.00997 |
| | 40 | 0.677 | 0.00805 | 0.675 | 0.00854 | 0.677 | 0.00806 | | 40 | 0.689 | 0.00900 | 0.689 | 0.00919 | 0.689 | 0.00901 |
| | 60 | 0.695 | 0.00806 | 0.693 | 0.00847 | 0.696 | 0.00808 | | 60 | 0.697 | 0.00798 | 0.697 | 0.00829 | 0.698 | 0.00798 |
| | 80 | 0.700 | 0.00785 | 0.700 | 0.00792 | 0.700 | 0.00785 | | 80 | 0.704 | 0.00782 | 0.704 | 0.00800 | 0.704 | 0.00783 |
| | 100 | 0.706 | 0.00763 | 0.707 | 0.00781 | 0.706 | 0.00764 | | 100 | 0.706 | 0.00763 | 0.707 | 0.00781 | 0.706 | 0.00764 |
| WIClustCDtarg | 10 | 0.654 | 0.00972 | 0.650 | 0.00991 | 0.654 | 0.00973 | PAM | 10 | 0.655 | 0.00877 | 0.651 | 0.00873 | 0.655 | 0.00878 |
| | 20 | 0.680 | 0.00918 | 0.678 | 0.00922 | 0.680 | 0.00918 | | 20 | 0.671 | 0.00917 | 0.667 | 0.00939 | 0.671 | 0.00917 |
| | 40 | 0.696 | 0.00784 | 0.697 | 0.00794 | 0.696 | 0.00784 | | 40 | 0.681 | 0.00893 | 0.680 | 0.00908 | 0.681 | 0.00893 |
| | 60 | 0.702 | 0.00792 | 0.702 | 0.00803 | 0.702 | 0.00793 | | 60 | 0.689 | 0.00921 | 0.689 | 0.00916 | 0.689 | 0.00922 |
| | 80 | 0.705 | 0.00771 | 0.705 | 0.00810 | 0.705 | 0.00771 | | 80 | 0.700 | 0.00822 | 0.701 | 0.00854 | 0.700 | 0.00824 |
| | 100 | 0.706 | 0.00763 | 0.707 | 0.00781 | 0.706 | 0.00764 | | 100 | 0.706 | 0.00763 | 0.707 | 0.00781 | 0.706 | 0.00764 |

**Table S31** Average accuracy and its corresponding standard error of the mean (SEM) across the 40 iterations for all training set optimization methods, models and training set (TRS) sizes (expressed as percentage of the candidate set (CS)) for switchgrass dataset and the simulated trait. If "targ" is added at the end of the name of a method, it corresponds to targeted optimization. Otherwise, untargeted optimization was performed.

**Switchgrass trait simulated1**

| | | Average accuracy | | | | | | | | Average accuracy | | | | | |
|---|---|---|---|---|---|---|---|---|---|---|---|---|---|---|---|
| | | GBLUP | | BayesB | | RKHS | | | | GBLUP | | BayesB | | RKHS | |
| Optimization method | TRS size (% of CS) | Mean | SEM | Mean | SEM | Mean | SEM | Optimization method | TRS size (% of CS) | Mean | SEM | Mean | SEM | Mean | SEM |
| RAND | 10 | 0.283 | 0.0331 | 0.292 | 0.0289 | 0.283 | 0.0330 | Rscore | 10 | 0.322 | 0.0266 | 0.328 | 0.0225 | 0.322 | 0.0265 |
| | 20 | 0.337 | 0.0238 | 0.341 | 0.0230 | 0.337 | 0.0233 | | 20 | 0.361 | 0.0225 | 0.364 | 0.0218 | 0.362 | 0.0224 |
| | 40 | 0.378 | 0.0232 | 0.381 | 0.0232 | 0.379 | 0.0229 | | 40 | 0.384 | 0.0183 | 0.381 | 0.0188 | 0.384 | 0.0184 |
| | 60 | 0.395 | 0.0227 | 0.399 | 0.0222 | 0.395 | 0.0226 | | 60 | 0.387 | 0.0199 | 0.385 | 0.0198 | 0.387 | 0.0199 |
| | 80 | 0.406 | 0.0227 | 0.409 | 0.0221 | 0.406 | 0.0226 | | 80 | 0.403 | 0.0182 | 0.407 | 0.0175 | 0.403 | 0.0181 |
| | 100 | 0.422 | 0.0189 | 0.420 | 0.0189 | 0.422 | 0.0189 | | 100 | 0.422 | 0.0189 | 0.420 | 0.0189 | 0.422 | 0.0189 |
| StratSamp | 10 | 0.325 | 0.0228 | 0.326 | 0.0224 | 0.326 | 0.0226 | Rscoretarg | 10 | 0.370 | 0.0257 | 0.371 | 0.0269 | 0.371 | 0.0257 |
| | 20 | 0.340 | 0.0273 | 0.356 | 0.0232 | 0.341 | 0.0271 | | 20 | 0.385 | 0.0262 | 0.389 | 0.0251 | 0.385 | 0.0260 |
| | 40 | 0.397 | 0.0194 | 0.401 | 0.0181 | 0.399 | 0.0192 | | 40 | 0.414 | 0.0231 | 0.413 | 0.0227 | 0.414 | 0.0230 |
| | 60 | 0.406 | 0.0212 | 0.406 | 0.0209 | 0.406 | 0.0210 | | 60 | 0.422 | 0.0224 | 0.423 | 0.0213 | 0.423 | 0.0220 |
| | 80 | 0.410 | 0.0208 | 0.411 | 0.0206 | 0.411 | 0.0208 | | 80 | 0.430 | 0.0222 | 0.431 | 0.0211 | 0.430 | 0.0221 |
| | 100 | 0.422 | 0.0189 | 0.420 | 0.0189 | 0.422 | 0.0189 | | 100 | 0.422 | 0.0189 | 0.420 | 0.0189 | 0.422 | 0.0189 |
| CD | 10 | 0.288 | 0.0251 | 0.300 | 0.0244 | 0.289 | 0.0249 | Avg_GRM | 10 | 0.132 | 0.0309 | 0.108 | 0.0340 | 0.135 | 0.0303 |
| | 20 | 0.313 | 0.0245 | 0.325 | 0.0232 | 0.314 | 0.0243 | | 20 | 0.158 | 0.0283 | 0.177 | 0.0293 | 0.169 | 0.0273 |
| | 40 | 0.345 | 0.0246 | 0.343 | 0.0251 | 0.345 | 0.0245 | | 40 | 0.253 | 0.0261 | 0.248 | 0.0256 | 0.248 | 0.0267 |
| | 60 | 0.365 | 0.0242 | 0.366 | 0.0238 | 0.366 | 0.0240 | | 60 | 0.288 | 0.0281 | 0.296 | 0.0284 | 0.285 | 0.0279 |
| | 80 | 0.386 | 0.0221 | 0.391 | 0.0215 | 0.386 | 0.0220 | | 80 | 0.404 | 0.0219 | 0.401 | 0.0217 | 0.404 | 0.0219 |
| | 100 | 0.422 | 0.0189 | 0.420 | 0.0189 | 0.422 | 0.0189 | | 100 | 0.422 | 0.0189 | 0.420 | 0.0189 | 0.422 | 0.0189 |
| CDtarg | 10 | 0.354 | 0.0269 | 0.368 | 0.0243 | 0.354 | 0.0267 | Avg_GRMtarg | 10 | 0.139 | 0.0315 | 0.152 | 0.0337 | 0.145 | 0.0307 |
| | 20 | 0.382 | 0.0258 | 0.399 | 0.0218 | 0.384 | 0.0254 | | 20 | 0.211 | 0.0332 | 0.207 | 0.0353 | 0.212 | 0.0324 |
| | 40 | 0.403 | 0.0245 | 0.409 | 0.0213 | 0.404 | 0.0243 | | 40 | 0.332 | 0.0312 | 0.326 | 0.0314 | 0.331 | 0.0313 |
| | 60 | 0.410 | 0.0234 | 0.414 | 0.0214 | 0.410 | 0.0233 | | 60 | 0.363 | 0.0282 | 0.367 | 0.0283 | 0.361 | 0.0284 |
| | 80 | 0.416 | 0.0222 | 0.417 | 0.0218 | 0.417 | 0.0222 | | 80 | 0.434 | 0.0192 | 0.431 | 0.0195 | 0.434 | 0.0192 |
| | 100 | 0.422 | 0.0189 | 0.420 | 0.0189 | 0.422 | 0.0189 | | 100 | 0.422 | 0.0189 | 0.420 | 0.0189 | 0.422 | 0.0189 |
| OvClustCD | 10 | 0.354 | 0.0301 | 0.370 | 0.0284 | 0.356 | 0.0299 | Avg_GRM_MinMax | 10 | 0.354 | 0.0267 | 0.362 | 0.0239 | 0.353 | 0.0266 |
| | 20 | 0.390 | 0.0238 | 0.392 | 0.0233 | 0.389 | 0.0238 | | 20 | 0.403 | 0.0203 | 0.402 | 0.0197 | 0.403 | 0.0202 |
| | 40 | 0.412 | 0.0242 | 0.418 | 0.0232 | 0.412 | 0.0242 | | 40 | 0.419 | 0.0197 | 0.418 | 0.0198 | 0.420 | 0.0196 |
| | 60 | 0.435 | 0.0236 | 0.438 | 0.0233 | 0.435 | 0.0235 | | 60 | 0.430 | 0.0192 | 0.425 | 0.0192 | 0.430 | 0.0190 |
| | 80 | 0.443 | 0.0229 | 0.447 | 0.0230 | 0.444 | 0.0229 | | 80 | 0.443 | 0.0196 | 0.441 | 0.0193 | 0.443 | 0.0195 |
| | 100 | 0.422 | 0.0189 | 0.420 | 0.0189 | 0.422 | 0.0189 | | 100 | 0.422 | 0.0189 | 0.420 | 0.0189 | 0.422 | 0.0189 |
| OvClustCDtarg | 10 | 0.365 | 0.0228 | 0.365 | 0.0225 | 0.367 | 0.0224 | Avg_GRM_MinMaxtarg | 10 | 0.369 | 0.0223 | 0.379 | 0.0217 | 0.370 | 0.0220 |
| | 20 | 0.405 | 0.0208 | 0.407 | 0.0186 | 0.405 | 0.0207 | | 20 | 0.399 | 0.0223 | 0.403 | 0.0215 | 0.399 | 0.0221 |
| | 40 | 0.418 | 0.0201 | 0.426 | 0.0182 | 0.419 | 0.0200 | | 40 | 0.416 | 0.0198 | 0.424 | 0.0185 | 0.418 | 0.0194 |
| | 60 | 0.428 | 0.0204 | 0.432 | 0.0192 | 0.428 | 0.0203 | | 60 | 0.435 | 0.0191 | 0.437 | 0.0191 | 0.436 | 0.0190 |
| | 80 | 0.435 | 0.0189 | 0.437 | 0.0188 | 0.435 | 0.0189 | | 80 | 0.441 | 0.0195 | 0.441 | 0.0190 | 0.441 | 0.0195 |
| | 100 | 0.422 | 0.0189 | 0.420 | 0.0189 | 0.422 | 0.0189 | | 100 | 0.422 | 0.0189 | 0.420 | 0.0189 | 0.422 | 0.0189 |
| WIClustCD | 10 | 0.372 | 0.0212 | 0.365 | 0.0213 | 0.372 | 0.0211 | Avg_GRM_self | 10 | 0.375 | 0.0245 | 0.376 | 0.0229 | 0.374 | 0.0244 |
| | 20 | 0.390 | 0.0206 | 0.392 | 0.0208 | 0.390 | 0.0204 | | 20 | 0.374 | 0.0248 | 0.384 | 0.0222 | 0.375 | 0.0247 |
| | 40 | 0.413 | 0.0219 | 0.408 | 0.0224 | 0.413 | 0.0218 | | 40 | 0.419 | 0.0211 | 0.421 | 0.0202 | 0.419 | 0.0210 |
| | 60 | 0.426 | 0.0202 | 0.426 | 0.0201 | 0.426 | 0.0202 | | 60 | 0.437 | 0.0180 | 0.434 | 0.0184 | 0.437 | 0.0179 |
| | 80 | 0.437 | 0.0197 | 0.434 | 0.0203 | 0.437 | 0.0197 | | 80 | 0.441 | 0.0189 | 0.439 | 0.0195 | 0.442 | 0.0188 |
| | 100 | 0.422 | 0.0189 | 0.420 | 0.0189 | 0.422 | 0.0189 | | 100 | 0.422 | 0.0189 | 0.420 | 0.0189 | 0.422 | 0.0189 |
| WIClustCDtarg | 10 | 0.372 | 0.0218 | 0.374 | 0.0218 | 0.372 | 0.0218 | PAM | 10 | 0.329 | 0.0231 | 0.343 | 0.0212 | 0.331 | 0.0230 |
| | 20 | 0.403 | 0.0207 | 0.414 | 0.0189 | 0.404 | 0.0206 | | 20 | 0.344 | 0.0248 | 0.365 | 0.0221 | 0.345 | 0.0248 |
| | 40 | 0.430 | 0.0200 | 0.429 | 0.0198 | 0.430 | 0.0199 | | 40 | 0.370 | 0.0234 | 0.376 | 0.0227 | 0.371 | 0.0234 |
| | 60 | 0.444 | 0.0192 | 0.441 | 0.0191 | 0.445 | 0.0190 | | 60 | 0.390 | 0.0220 | 0.393 | 0.0216 | 0.391 | 0.0219 |
| | 80 | 0.444 | 0.0188 | 0.441 | 0.0190 | 0.444 | 0.0188 | | 80 | 0.408 | 0.0212 | 0.412 | 0.0198 | 0.408 | 0.0212 |
| | 100 | 0.422 | 0.0189 | 0.420 | 0.0189 | 0.422 | 0.0189 | | 100 | 0.422 | 0.0189 | 0.420 | 0.0189 | 0.422 | 0.0189 |

**Table S32** Percentage of gain in Area Under the Curve (AUC) for every optimization method and trait compared to random sampling using the GBLUP model in the maize dataset. Area under the curve was generated using agricolae package. AUC is the area under the curves generated when plotting the accuracy of the model against the training set size. As no optimization was performed when using the entire candidate set, results in the table shows the AUC for training set size from 10 to 80% of the candidate set. We present the mean and the standard error of the mean (SEM) over the 40 repetitions. Asterisks represent whether or not there is significant difference between the AUC for the training set optimization method and random sampling in a Wilcoxon signed-rank test. Sig: Significance. $*$ P $<$ 0.05, $**$ P $<$ 0.01, $***$ P $<$ 0.001.

**Maize GBLUP**

| | | % of gain in AUC | | | | | | | | | | | |
|---|---|---|---|---|---|---|---|---|---|---|---|---|---|
| | | YLD | | | HT | | | FT | | | Simulated | | |
| Type | Algorithm | Mean | SEM | Sig | Mean | SEM | Sig | Mean | SEM | Sig | Mean | SEM | Sig |
| Untargeted | StratSamp | -0.93 | 1.24 | | 0.49 | 2.03 | | -2.18 | 1.15 | | 5.59 | 10.19 | |
| | PAM | -1.26 | 1.69 | | 4.92 | 2.10 | * | 2.00 | 0.92 | | 10.70 | 12.27 | |
| | Rscore | -1.82 | 1.18 | | -0.78 | 1.81 | | -1.83 | 0.96 | | 4.78 | 10.49 | |
| | CDmean | -1.01 | 1.26 | | 3.25 | 2.08 | | 1.04 | 0.80 | | 9.77 | 10.41 | |
| | OvClustCDmean | -0.49 | 1.35 | | 2.54 | 2.24 | | -0.33 | 0.90 | | 11.29 | 12.01 | |
| | WIClustCDmean | -1.37 | 1.08 | | -0.44 | 2.03 | | 0.14 | 0.96 | | -5.57 | 12.09 | |
| | Avg_GRM | -11.21 | 2.45 | *** | -13.48 | 3.98 | ** | -18.08 | 2.20 | *** | -33.93 | 11.62 | * |
| | Avg_GRM_MinMax | 1.87 | 1.19 | | 5.23 | 2.07 | * | 4.37 | 0.97 | *** | -6.72 | 11.93 | |
| | Avg_GRM_self | 2.96 | 1.03 | ** | 5.87 | 2.06 | ** | 6.04 | 1.08 | *** | 2.42 | 12.46 | |
| Targeted | Avg_GRM | -1.91 | 1.92 | | -5.06 | 2.93 | | 0.69 | 1.72 | | 2.32 | 12.46 | |
| | Avg_GRM_MinMax | 6.04 | 1.27 | *** | 6.57 | 1.73 | *** | 9.73 | 1.06 | *** | 7.44 | 11.93 | |
| | Rscore | 1.72 | 1.26 | | 1.35 | 1.48 | | 4.44 | 1.03 | *** | 6.60 | 12.82 | |
| | CDmean | 4.41 | 1.41 | ** | 6.47 | 1.81 | ** | 7.02 | 1.18 | *** | 30.91 | 10.79 | * |
| | OvClustCDmean | 5.77 | 1.39 | *** | 7.45 | 1.97 | ** | 8.39 | 1.15 | *** | 16.76 | 12.24 | |
| | WIClustCDmean | 6.16 | 1.31 | *** | 8.67 | 2.18 | *** | 9.01 | 1.19 | *** | 6.03 | 12.98 | |

**Table S33** Percentage of gain in Area Under the Curve (AUC) for every optimization method and trait compared to random sampling using the BayesB model in the maize dataset. Area under the curve was generated using agricolae package. AUC is the area under the curves generated when plotting the accuracy of the model against the training set size. As no optimization was performed when using the entire candidate set, results in the table shows the AUC for training set size from 10 to 80% of the candidate set. We present the mean and the standard error of the mean (SEM) over the 40 repetitions. Asterisks represent whether or not there is significant difference between the AUC for the training set optimization method and random sampling in a Wilcoxon signed-rank test. Sig: Significance. $*$ P $<$ 0.05, $**$ P $<$ 0.01, $***$ P $<$ 0.001.

**Maize BayesB**

| | | % of gain in AUC | | | | | | | | | | | |
|---|---|---|---|---|---|---|---|---|---|---|---|---|---|
| | | YLD | | | HT | | | FT | | | Simulated | | |
| Type | Algorithm | Mean | SEM | Sig | Mean | SEM | Sig | Mean | SEM | Sig | Mean | SEM | Sig |
| Untargeted | StratSamp | -1.36 | 1.17 | | 0.28 | 1.94 | | -1.89 | 1.13 | | 6.71 | 10.15 | |
| | PAM | 0.07 | 1.55 | | 5.62 | 1.95 | * | 2.15 | 0.90 | | 12.81 | 12.07 | |
| | Rscore | -1.54 | 1.12 | | -0.75 | 1.83 | | -1.63 | 0.97 | | 5.73 | 10.69 | |
| | CDmean | -0.32 | 1.18 | | 5.02 | 1.93 | * | 1.69 | 0.78 | | 10.34 | 10.68 | |
| | OvClustCDmean | -0.06 | 1.37 | | 3.39 | 2.28 | | -0.13 | 0.89 | | 11.22 | 11.94 | |
| | WIClustCDmean | -0.95 | 1.12 | | 0.78 | 1.86 | | 0.49 | 0.93 | | -6.07 | 12.12 | |
| | Avg_GRM | -11.35 | 2.30 | *** | -12.02 | 3.82 | ** | -18.90 | 2.24 | *** | -34.28 | 11.62 | * |
| | Avg_GRM_MinMax | 1.48 | 1.13 | | 5.45 | 2.02 | ** | 4.94 | 0.94 | *** | -6.94 | 11.94 | |
| | Avg_GRM_self | 3.41 | 1.03 | ** | 6.34 | 2.06 | ** | 6.48 | 1.06 | *** | 2.07 | 12.43 | |
| Targeted | Avg_GRM | -2.30 | 1.97 | | -2.95 | 2.84 | | 0.46 | 1.75 | | 0.77 | 12.58 | |
| | Avg_GRM_MinMax | 5.70 | 1.27 | *** | 6.91 | 1.70 | *** | 9.87 | 1.05 | *** | 6.20 | 12.03 | |
| | Rscore | 1.33 | 1.24 | | 1.47 | 1.43 | | 4.79 | 1.08 | *** | 6.39 | 12.76 | |
| | CDmean | 3.41 | 1.37 | ** | 5.76 | 1.87 | ** | 6.99 | 1.21 | *** | 29.16 | 11.08 | * |
| | OvClustCDmean | 5.12 | 1.38 | *** | 7.30 | 1.98 | ** | 8.58 | 1.14 | *** | 17.10 | 12.11 | |
| | WIClustCDmean | 5.52 | 1.34 | *** | 8.89 | 2.12 | *** | 9.27 | 1.15 | *** | 6.92 | 12.97 | |

**Table S34** Percentage of gain in Area Under the Curve (AUC) for every optimization method and trait compared to random sampling using the RKHS model in the maize dataset. Area under the curve was generated using agricolae package. AUC is the area under the curves generated when plotting the accuracy of the model against the training set size. As no optimization was performed when using the entire candidate set, results in the table shows the AUC for training set size from 10 to 80percentage of the candidate set. We present the mean and the standard error of the mean (SEM) over the 40 repetitions. Asterisks represent whether or not there is significant difference between the AUC for the training set optimization method and random sampling in a Wilcoxon signed-rank test. Sig: Significance. ∗ P < 0.05, ∗∗ P < 0.01, ∗ ∗ ∗ P < 0.001.

**Maize RKHS**

| | | % of gain in AUC | | | | | | | | | | | |
|---|---|---|---|---|---|---|---|---|---|---|---|---|---|
| | | YLD | | | HT | | | FT | | | Simulated | | |
| Type | Algorithm | Mean | SEM | Sig | Mean | SEM | Sig | Mean | SEM | Sig | Mean | SEM | Sig |
| Untargeted | StratSamp | -0.96 | 1.25 | | 0.51 | 1.90 | | -2.20 | 1.14 | | 5.92 | 10.03 | |
| | PAM | -1.14 | 1.69 | | 5.15 | 2.14 | * | 1.99 | 0.93 | | 8.98 | 12.10 | |
| | Rscore | -1.99 | 1.18 | | -0.75 | 1.87 | | -1.90 | 0.96 | | 3.05 | 10.89 | |
| | CDmean | -0.95 | 1.29 | | 3.64 | 2.08 | | 1.06 | 0.79 | | 8.67 | 10.51 | |
| | OvClustCDmean | -0.55 | 1.37 | | 2.66 | 2.29 | | -0.71 | 0.91 | | 9.93 | 12.18 | |
| | WIClustCDmean | -1.49 | 1.09 | | -0.72 | 2.15 | | -0.08 | 0.99 | | -5.40 | 12.05 | |
| | Avg_GRM | -11.61 | 2.49 | *** | -13.75 | 4.08 | ** | -18.00 | 2.25 | *** | -33.26 | 11.75 | * |
| | Avg_GRM_MinMax | 1.77 | 1.19 | | 5.31 | 2.11 | * | 4.28 | 0.97 | *** | -6.81 | 11.90 | |
| | Avg_GRM_self | 2.92 | 1.03 | ** | 6.03 | 2.06 | ** | 5.96 | 1.08 | *** | 1.84 | 12.47 | |
| Targeted | Avg_GRM | -3.00 | 2.06 | | -5.26 | 3.06 | | 0.32 | 1.76 | | 0.38 | 12.80 | |
| | Avg_GRM_MinMax | 5.90 | 1.26 | *** | 6.77 | 1.74 | *** | 9.57 | 1.07 | *** | 5.78 | 12.22 | |
| | Rscore | 1.42 | 1.27 | | 0.93 | 1.53 | | 4.27 | 1.02 | *** | 6.60 | 12.88 | |
| | CDmean | 4.23 | 1.42 | ** | 6.06 | 1.80 | ** | 6.71 | 1.19 | *** | 28.96 | 11.02 | * |
| | OvClustCDmean | 5.66 | 1.39 | *** | 7.16 | 2.01 | ** | 8.25 | 1.16 | *** | 16.78 | 12.20 | |
| | WIClustCDmean | 5.98 | 1.31 | *** | 8.35 | 2.12 | *** | 8.91 | 1.19 | *** | 5.73 | 13.10 | |

**Table S35** Percentage of gain in Area Under the Curve (AUC) for every optimization method and trait compared to random sampling using the GBLUP model in the rice dataset. Area under the curve was generated using agricolae package. AUC is the area under the curves generated when plotting the accuracy of the model against the training set size. As no optimization was performed when using the entire candidate set, results in the table shows the AUC for training set size from 10 to 80% of the candidate set. We present the mean and the standard error of the mean (SEM) over the 40 repetitions. Asterisks represent whether or not there is significant difference between the AUC for the training set optimization method and random sampling in a Wilcoxon signed-rank test. Sig: Significance. ∗ $P < 0.05$, ∗∗ $P < 0.01$, ∗ ∗ ∗ $P < 0.001$.

**Rice GBLUP**

| | | % of gain in AUC | | | | | | | | | | | |
|---|---|---|---|---|---|---|---|---|---|---|---|---|---|
| | | YLD | | | HT | | | FT | | | Simulated | | |
| Type | Algorithm | Mean | SEM | Sig | Mean | SEM | Sig | Mean | SEM | Sig | Mean | SEM | Sig |
| Untargeted | StratSamp | 0.91 | 5.82 | | -0.05 | 2.93 | | -1.03 | 2.20 | | -6.36 | 6.81 | |
| | PAM | 15.42 | 4.90 | ** | 1.63 | 3.16 | | 1.22 | 2.55 | | 10.89 | 7.32 | |
| | Rscore | 6.96 | 6.40 | | 1.81 | 3.39 | | 0.60 | 2.97 | | 4.46 | 8.22 | |
| | CDmean | 27.59 | 6.04 | *** | 6.74 | 2.94 | * | 8.89 | 2.61 | *** | 1.57 | 7.53 | |
| | OvClustCDmean | 26.29 | 5.62 | *** | 8.13 | 3.02 | * | 8.48 | 2.54 | ** | 4.98 | 8.76 | |
| | WIClustCDmean | 21.88 | 5.15 | *** | 4.83 | 3.17 | | 5.61 | 2.36 | * | 8.50 | 7.99 | |
| | Avg_GRM | -29.67 | 8.35 | ** | -27.47 | 4.10 | *** | -21.04 | 3.44 | *** | -8.49 | 8.37 | |
| | Avg_GRM_MinMax | 9.10 | 6.56 | | 2.16 | 2.76 | | 4.37 | 2.78 | | 1.49 | 7.64 | |
| | Avg_GRM_self | 18.88 | 6.14 | ** | 10.96 | 3.00 | ** | 7.89 | 2.47 | ** | 0.58 | 7.97 | |
| Targeted | Avg_GRM | 27.54 | 7.64 | ** | 11.41 | 3.80 | ** | 9.70 | 3.25 | ** | 1.51 | 7.46 | |
| | Avg_GRM_MinMax | 30.38 | 6.98 | *** | 16.26 | 3.37 | *** | 12.64 | 3.14 | *** | 5.95 | 7.54 | |
| | Rscore | 19.58 | 5.37 | ** | 19.20 | 3.04 | *** | 9.90 | 2.85 | *** | 15.22 | 7.68 | |
| | CDmean | 47.07 | 6.85 | *** | 32.70 | 3.27 | *** | 20.07 | 2.54 | *** | 11.46 | 7.25 | |
| | OvClustCDmean | 49.28 | 6.65 | *** | 32.88 | 3.39 | *** | 19.93 | 2.65 | *** | 24.87 | 7.63 | ** |
| | WIClustCDmean | 43.41 | 7.15 | *** | 30.96 | 3.22 | *** | 19.02 | 2.71 | *** | 7.07 | 8.35 | |

**Table S36** Percentage of gain in Area Under the Curve (AUC) for every optimization method and trait compared to random sampling using the BayesB model in the rice dataset. Area under the curve was generated using agricolae package. AUC is the area under the curves generated when plotting the accuracy of the model against the training set size. As no optimization was performed when using the entire candidate set, results in the table shows the AUC for training set size from 10 to 80% of the candidate set. We present the mean and the standard error of the mean (SEM) over the 40 repetitions. Asterisks represent whether or not there is significant difference between the AUC for the training set optimization method and random sampling in a Wilcoxon signed-rank test. Sig: Significance. $*$ P $<$ 0.05, $**$ P $<$ 0.01, $***$ P $<$ 0.001.

**Rice BayesB**

| | | % of gain in AUC | | | | | | | | | | | |
|---|---|---|---|---|---|---|---|---|---|---|---|---|---|
| | | YLD | | | HT | | | FT | | | Simulated | | |
| Type | Algorithm | Mean | SEM | Sig | Mean | SEM | Sig | Mean | SEM | Sig | Mean | SEM | Sig |
| Untargeted | StratSamp | 1.55 | 5.04 | | 0.52 | 3.14 | | -2.71 | 1.80 | | -5.88 | 6.89 | |
| | PAM | 17.46 | 4.33 | *** | 3.71 | 3.02 | | -2.82 | 2.21 | | 11.10 | 7.43 | |
| | Rscore | 9.17 | 5.67 | | 2.00 | 3.34 | | -0.78 | 2.51 | | 4.24 | 8.29 | |
| | CDmean | 26.50 | 4.77 | *** | 8.41 | 2.93 | ** | 9.01 | 2.29 | *** | 2.58 | 7.45 | |
| | OvClustCDmean | 25.25 | 4.81 | *** | 8.98 | 3.14 | * | 8.50 | 2.11 | *** | 5.18 | 8.75 | |
| | WIClustCDmean | 24.75 | 4.41 | *** | 6.44 | 3.08 | * | 5.16 | 1.84 | * | 8.54 | 7.92 | |
| | Avg_GRM | -32.64 | 7.98 | *** | -26.74 | 4.22 | *** | -19.91 | 2.89 | *** | -9.54 | 8.43 | |
| | Avg_GRM_MinMax | 13.71 | 5.64 | ** | 1.30 | 2.89 | | 1.76 | 2.45 | | 1.98 | 7.41 | |
| | Avg_GRM_self | 19.49 | 5.31 | ** | 10.44 | 3.22 | ** | 4.80 | 2.29 | | 2.66 | 7.78 | |
| Targeted | Avg_GRM | 24.72 | 6.82 | ** | 11.53 | 3.83 | ** | 9.39 | 3.12 | ** | 2.22 | 7.26 | |
| | Avg_GRM_MinMax | 28.69 | 5.83 | *** | 15.20 | 3.46 | *** | 10.45 | 2.80 | *** | 6.19 | 7.45 | |
| | Rscore | 18.54 | 4.63 | *** | 19.27 | 3.13 | *** | 7.98 | 2.60 | ** | 15.68 | 7.55 | * |
| | CDmean | 42.08 | 5.80 | *** | 31.42 | 3.27 | *** | 16.96 | 2.53 | *** | 11.64 | 7.25 | |
| | OvClustCDmean | 44.37 | 5.72 | *** | 31.58 | 3.45 | *** | 16.49 | 2.64 | *** | 26.18 | 7.80 | ** |
| | WIClustCDmean | 38.91 | 6.08 | *** | 29.34 | 3.33 | *** | 16.74 | 2.69 | *** | 8.00 | 8.25 | |

**Table S37** Percentage of gain in Area Under the Curve (AUC) for every optimization method and trait compared to random sampling using the RKHS model in the rice dataset. Area under the curve was generated using agricolae package. AUC is the area under the curves generated when plotting the accuracy of the model against the training set size. As no optimization was performed when using the entire candidate set, results in the table shows the AUC for training set size from 10 to 80% of the candidate set. We present the mean and the standard error of the mean (SEM) over the 40 repetitions. Asterisks represent whether or not there is significant difference between the AUC for the training set optimization method and random sampling in a Wilcoxon signed-rank test. Sig: Significance. $*$ P $<$ 0.05, $**$ P $<$ 0.01, $***$ P $<$ 0.001.

**Rice RKHS**

| | | % of gain in AUC | | | | | | | | | | | |
|---|---|---|---|---|---|---|---|---|---|---|---|---|---|
| | | YLD | | | HT | | | FT | | | Simulated | | |
| Type | Algorithm | Mean | SEM | Sig | Mean | SEM | Sig | Mean | SEM | Sig | Mean | SEM | Sig |
| Untargeted | StratSamp | 1.53 | 4.44 | | 0.88 | 2.86 | | -0.80 | 2.12 | | -5.98 | 6.93 | |
| | PAM | 21.31 | 4.39 | *** | 3.70 | 3.02 | | 4.29 | 2.61 | | 11.21 | 7.28 | |
| | Rscore | 6.13 | 5.55 | | 3.24 | 3.23 | | -0.16 | 2.88 | | 4.11 | 8.17 | |
| | CDmean | 26.14 | 5.09 | *** | 7.59 | 2.73 | * | 9.38 | 2.82 | *** | 1.58 | 7.32 | |
| | OvClustCDmean | 23.46 | 4.61 | *** | 8.98 | 2.80 | ** | 9.55 | 2.65 | ** | 5.16 | 8.80 | |
| | WlClustCDmean | 21.81 | 4.24 | *** | 5.05 | 2.89 | | 6.25 | 2.39 | * | 8.88 | 8.03 | |
| | Avg_GRM | -30.08 | 7.46 | *** | -28.68 | 3.93 | *** | -21.50 | 3.33 | *** | -8.54 | 8.63 | |
| | Avg_GRM_MinMax | 11.99 | 5.56 | * | 3.54 | 2.77 | | 5.03 | 2.73 | | 1.86 | 7.70 | |
| | Avg_GRM_self | 19.13 | 5.20 | *** | 11.94 | 2.98 | *** | 8.76 | 2.50 | ** | 1.53 | 8.04 | |
| Targeted | Avg_GRM | 21.13 | 6.65 | ** | 12.23 | 3.33 | *** | 8.71 | 3.25 | * | 1.14 | 7.64 | |
| | Avg_GRM_MinMax | 26.90 | 5.60 | *** | 17.72 | 3.26 | *** | 12.46 | 3.10 | *** | 6.69 | 7.53 | |
| | Rscore | 17.93 | 4.25 | *** | 21.13 | 2.81 | *** | 10.15 | 2.89 | ** | 15.39 | 7.79 | * |
| | CDmean | 44.42 | 5.07 | *** | 34.05 | 3.01 | *** | 19.86 | 2.57 | *** | 12.80 | 7.30 | |
| | OvClustCDmean | 46.01 | 4.87 | *** | 34.27 | 3.08 | *** | 19.58 | 2.72 | *** | 25.31 | 7.65 | ** |
| | WlClustCDmean | 41.30 | 5.20 | *** | 32.07 | 3.09 | *** | 18.64 | 2.75 | *** | 8.51 | 8.39 | |

**Table S38** Percentage of gain in Area Under the Curve (AUC) for every optimization method and trait compared to random sampling using the GBLUP model in the soybean dataset. Area under the curve was generated using agricolae package. AUC is the area under the curves generated when plotting the accuracy of the model against the training set size. As no optimization was performed when using the entire candidate set, results in the table shows the AUC for training set size from 10 to 80% of the candidate set. We present the mean and the standard error of the mean (SEM) over the 40 repetitions. Asterisks represent whether or not there is significant difference between the AUC for the training set optimization method and random sampling in a Wilcoxon signed-rank test. Sig: Significance. $*$ P $<$ 0.05, $**$ P $<$ 0.01, $***$ P $<$ 0.001.

Soybean GBLUP

| | | % of gain in AUC | | | | | | | | | | | |
|---|---|---|---|---|---|---|---|---|---|---|---|---|---|
| | | YLD | | | HT | | | R8 | | | Simulated | | |
| Type | Algorithm | Mean | SEM | Sig | Mean | SEM | Sig | Mean | SEM | Sig | Mean | SEM | Sig |
| Untargeted | StratSamp | 1.54 | 0.57 | ** | 2.35 | 1.91 | | 0.55 | 1.62 | | 0.96 | 0.47 | * |
| | PAM | 1.53 | 0.60 | * | 3.11 | 1.72 | | 0.92 | 1.70 | | 0.86 | 0.50 | |
| | Rscore | 1.23 | 0.56 | | -0.81 | 1.82 | | -0.54 | 1.57 | | 0.60 | 0.42 | |
| | CDmean | 2.39 | 0.58 | *** | 0.70 | 2.05 | | 0.82 | 1.60 | | 1.60 | 0.43 | *** |
| | OvClustCDmean | 2.63 | 0.49 | *** | 0.35 | 1.83 | | 2.48 | 1.60 | | 1.84 | 0.41 | *** |
| | WIClustCDmean | 2.37 | 0.59 | *** | 0.93 | 2.18 | | 1.16 | 1.57 | | 1.14 | 0.42 | ** |
| | Avg_GRM | 0.55 | 0.69 | | -2.24 | 2.14 | | -8.45 | 1.76 | *** | -0.53 | 0.64 | |
| | Avg_GRM_MinMax | 1.05 | 0.64 | * | 1.45 | 2.11 | | 0.71 | 1.54 | | 1.30 | 0.43 | ** |
| | Avg_GRM_self | 1.58 | 0.58 | ** | 2.83 | 1.55 | * | 3.03 | 1.40 | * | 0.81 | 0.51 | |
| Targeted | Avg_GRM | -0.09 | 0.83 | | 1.38 | 2.03 | | -0.22 | 1.83 | | 0.35 | 0.55 | |
| | Avg_GRM_MinMax | 1.45 | 0.58 | * | 1.11 | 1.54 | | 1.91 | 1.40 | | 0.82 | 0.51 | |
| | Rscore | 0.12 | 0.69 | | 4.41 | 1.64 | ** | 1.98 | 1.39 | | 1.33 | 0.47 | ** |
| | CDmean | 3.06 | 0.57 | *** | 3.97 | 1.71 | * | 3.20 | 1.48 | * | 2.25 | 0.38 | *** |
| | OvClustCDmean | 2.86 | 0.58 | *** | 4.21 | 1.90 | * | 2.99 | 1.56 | | 2.03 | 0.42 | *** |
| | WIClustCDmean | 3.50 | 0.65 | *** | 6.14 | 1.83 | ** | 5.05 | 1.78 | ** | 1.68 | 0.50 | *** |

**Table S39** Percentage of gain in Area Under the Curve (AUC) for every optimization method and trait compared to random sampling using the BayesB model in the soybean dataset. Area under the curve was generated using agricolae package. AUC is the area under the curves generated when plotting the accuracy of the model against the training set size. As no optimization was performed when using the entire candidate set, results in the table shows the AUC for training set size from 10 to 80% of the candidate set. We present the mean and the standard error of the mean (SEM) over the 40 repetitions. Asterisks represent whether or not there is significant difference between the AUC for the training set optimization method and random sampling in a Wilcoxon signed-rank test. Sig: Significance. $*$ $P < 0.05$, $**$ $P < 0.01$, $***$ $P < 0.001$.

Soybean BayesB

| | | % of gain in AUC | | | | | | | | | | | |
|---|---|---|---|---|---|---|---|---|---|---|---|---|---|
| | | YLD | | | HT | | | R8 | | | Simulated | | |
| Type | Algorithm | Mean | SEM | Sig | Mean | SEM | Sig | Mean | SEM | Sig | Mean | SEM | Sig |
| Untargeted | StratSamp | 1.52 | 0.52 | ** | 2.29 | 1.74 | | -0.17 | 1.58 | | 0.94 | 0.48 | * |
| | PAM | 1.41 | 0.58 | * | 2.65 | 1.59 | | 0.35 | 1.59 | | 0.88 | 0.50 | |
| | Rscore | 1.25 | 0.54 | * | -0.71 | 1.65 | | -1.42 | 1.51 | | 0.61 | 0.43 | |
| | CDmean | 2.42 | 0.56 | *** | 1.42 | 1.82 | | 0.50 | 1.51 | | 1.65 | 0.44 | *** |
| | OvClustCDmean | 2.68 | 0.48 | *** | 0.53 | 1.68 | | 2.13 | 1.48 | | 1.83 | 0.41 | *** |
| | WIClustCDmean | 2.39 | 0.57 | *** | 0.96 | 1.94 | | 0.99 | 1.48 | | 1.17 | 0.43 | ** |
| | Avg_GRM | 0.52 | 0.67 | | -3.20 | 1.94 | | -9.61 | 1.68 | *** | -0.55 | 0.67 | |
| | Avg_GRM_MinMax | 1.03 | 0.62 | * | 2.06 | 1.88 | | 0.18 | 1.44 | | 1.35 | 0.43 | ** |
| | Avg_GRM_self | 1.74 | 0.57 | ** | 2.91 | 1.38 | * | 2.81 | 1.33 | | 0.86 | 0.51 | |
| Targeted | Avg_GRM | -0.09 | 0.79 | | 1.08 | 1.76 | | -1.13 | 1.72 | | 0.32 | 0.56 | |
| | Avg_GRM_MinMax | 1.52 | 0.56 | ** | 1.61 | 1.38 | | 1.68 | 1.32 | | 0.88 | 0.52 | |
| | Rscore | 0.25 | 0.67 | | 3.13 | 1.42 | * | 0.94 | 1.35 | | 1.30 | 0.48 | ** |
| | CDmean | 2.91 | 0.57 | *** | 4.03 | 1.49 | * | 2.54 | 1.38 | | 2.23 | 0.39 | *** |
| | OvClustCDmean | 2.76 | 0.58 | *** | 3.72 | 1.68 | * | 2.28 | 1.48 | | 2.05 | 0.44 | *** |
| | WIClustCDmean | 3.49 | 0.63 | *** | 5.71 | 1.66 | ** | 4.35 | 1.60 | * | 1.68 | 0.50 | *** |

**Table S40** Percentage of gain in Area Under the Curve (AUC) for every optimization method and trait compared to random sampling using the RKHS model in the soybean dataset. Area under the curve was generated using agricolae package. AUC is the area under the curves generated when plotting the accuracy of the model against the training set size. As no optimization was performed when using the entire candidate set, results in the table shows the AUC for training set size from 10 to 80% of the candidate set. We present the mean and the standard error of the mean (SEM) over the 40 repetitions. Asterisks represent whether or not there is significant difference between the AUC for the training set optimization method and random sampling in a Wilcoxon signed-rank test. Sig: Significance. $*$ P $<$ 0.05, $**$ P $<$ 0.01, $***$ P $<$ 0.001.

Soybean RKHS

| | | % of gain in AUC | | | | | | | | | | | |
|---|---|---|---|---|---|---|---|---|---|---|---|---|---|
| | | YLD | | | HT | | | R8 | | | Simulated | | |
| Type | Algorithm | Mean | SEM | Sig | Mean | SEM | Sig | Mean | SEM | Sig | Mean | SEM | Sig |
| Untargeted | StratSamp | 1.76 | 0.54 | ** | 2.66 | 1.80 | | 1.77 | 1.64 | | 1.00 | 0.49 | * |
| | PAM | 1.72 | 0.59 | ** | 2.47 | 1.61 | | 1.59 | 1.73 | | 0.94 | 0.51 | |
| | Rscore | 1.21 | 0.56 | | 0.10 | 1.69 | | 0.39 | 1.52 | | 0.69 | 0.44 | |
| | CDmean | 2.59 | 0.59 | *** | 0.45 | 2.01 | | 2.42 | 1.71 | | 1.69 | 0.47 | *** |
| | OvClustCDmean | 2.94 | 0.52 | *** | 0.84 | 1.79 | | 4.04 | 1.61 | * | 1.82 | 0.43 | *** |
| | WIClustCDmean | 2.61 | 0.61 | *** | 1.17 | 2.02 | | 3.04 | 1.58 | | 1.20 | 0.44 | ** |
| | Avg_GRM | 0.72 | 0.66 | | -0.65 | 1.88 | | -7.09 | 1.61 | *** | -0.49 | 0.67 | |
| | Avg_GRM_MinMax | 1.13 | 0.62 | | 1.29 | 1.95 | | 2.20 | 1.67 | | 1.43 | 0.44 | ** |
| | Avg_GRM_self | 1.74 | 0.59 | ** | 1.70 | 1.65 | | 3.33 | 1.53 | * | 0.90 | 0.53 | |
| Targeted | Avg_GRM | -0.14 | 0.82 | | 1.13 | 1.87 | | 1.12 | 1.76 | | 0.51 | 0.57 | |
| | Avg_GRM_MinMax | 1.46 | 0.60 | * | 0.62 | 1.58 | | 2.72 | 1.53 | * | 0.97 | 0.53 | |
| | Rscore | 0.16 | 0.71 | | 2.37 | 1.59 | | 1.58 | 1.51 | | 1.31 | 0.50 | ** |
| | CDmean | 3.30 | 0.55 | *** | 4.48 | 1.88 | * | 4.21 | 1.63 | * | 2.26 | 0.42 | *** |
| | OvClustCDmean | 3.05 | 0.58 | *** | 3.34 | 2.02 | | 3.64 | 1.71 | * | 2.01 | 0.46 | *** |
| | WIClustCDmean | 3.78 | 0.62 | *** | 4.94 | 1.92 | * | 5.28 | 1.93 | * | 1.57 | 0.53 | ** |

**Table S41** Percentage of gain in Area Under the Curve (AUC) for every optimization method and trait compared to random sampling using the GBLUP model in the spruce dataset. Area under the curve was generated using agricolae package. AUC is the area under the curves generated when plotting the accuracy of the model against the training set size. As no optimization was performed when using the entire candidate set, results in the table shows the AUC for training set size from 10 to 80% of the candidate set. We present the mean and the standard error of the mean (SEM) over the 40 repetitions. Asterisks represent whether or not there is significant difference between the AUC for the training set optimization method and random sampling in a Wilcoxon signed-rank test. Sig: Significance. $*$ P $<$ 0.05, $**$ P $<$ 0.01, $***$ P $<$ 0.001.

**Spruce GBLUP**

| | | % of gain in AUC | | | | | | | | | | | |
|---|---|---|---|---|---|---|---|---|---|---|---|---|---|
| | | HT | | | DBH | | | DE | | | Simulated | | |
| Type | Algorithm | Mean | SEM | Sig | Mean | SEM | Sig | Mean | SEM | Sig | Mean | SEM | Sig |
| Untargeted | StratSamp | 0.89 | 1.04 | | -0.33 | 1.94 | | -1.44 | 0.84 | | 3.54 | 2.36 | |
| | PAM | -1.93 | 1.23 | | -0.80 | 1.99 | | -0.77 | 0.94 | | 3.98 | 3.12 | |
| | Rscore | -1.18 | 0.87 | | -2.00 | 1.77 | | 0.72 | 0.73 | | 6.57 | 2.62 | ** |
| | CDmean | 0.95 | 1.19 | | 0.51 | 1.68 | | 1.37 | 0.58 | * | 0.77 | 2.56 | |
| | OvClustCDmean | 0.11 | 1.21 | | 0.55 | 1.77 | | 0.43 | 0.78 | | 1.26 | 2.86 | |
| | WIClustCDmean | -0.30 | 1.12 | | -0.11 | 1.88 | | 2.26 | 0.66 | *** | 0.54 | 2.83 | |
| | Avg_GRM | -16.43 | 1.78 | *** | -19.99 | 2.04 | *** | -11.86 | 1.10 | *** | -11.27 | 3.07 | *** |
| | Avg_GRM_MinMax | 1.08 | 1.16 | | 1.34 | 1.79 | | 0.46 | 0.69 | | 0.38 | 2.84 | |
| | Avg_GRM_self | 0.90 | 1.11 | | 0.70 | 1.80 | | 0.00 | 0.87 | | 1.00 | 2.80 | |
| Targeted | Avg_GRM | -5.66 | 1.66 | ** | -3.68 | 1.90 | * | -4.53 | 1.11 | *** | -3.14 | 2.72 | |
| | Avg_GRM_MinMax | 2.47 | 1.31 | * | 2.72 | 2.10 | | 1.53 | 0.66 | * | 1.51 | 2.69 | |
| | Rscore | -0.14 | 1.11 | | -2.39 | 1.73 | | 1.10 | 0.71 | | 5.28 | 3.27 | |
| | CDmean | 5.31 | 1.40 | *** | 8.13 | 2.15 | *** | 3.22 | 0.75 | *** | 2.47 | 2.98 | |
| | OvClustCDmean | 4.33 | 1.32 | ** | 5.64 | 1.95 | * | 3.19 | 0.89 | ** | 5.02 | 2.54 | |
| | WIClustCDmean | 5.60 | 1.49 | *** | 8.43 | 2.19 | *** | 4.68 | 0.85 | *** | 3.65 | 2.75 | |

**Table S42** Percentage of gain in Area Under the Curve (AUC) for every optimization method and trait compared to random sampling using the BayesB model in the spruce dataset. Area under the curve was generated using agricolae package. AUC is the area under the curves generated when plotting the accuracy of the model against the training set size. As no optimization was performed when using the entire candidate set, results in the table shows the AUC for training set size from 10 to 80% of the candidate set. We present the mean and the standard error of the mean (SEM) over the 40 repetitions. Asterisks represent whether or not there is significant difference between the AUC for the training set optimization method and random sampling in a Wilcoxon signed-rank test. Sig: Significance. $*$ P $<$ 0.05, $**$ P $<$ 0.01, $***$ P $<$ 0.001.

**Spruce BayesB**

| | | % of gain in AUC | | | | | | | | | | | |
|---|---|---|---|---|---|---|---|---|---|---|---|---|---|
| | | HT | | | DBH | | | DE | | | Simulated | | |
| Type | Algorithm | Mean | SEM | Sig | Mean | SEM | Sig | Mean | SEM | Sig | Mean | SEM | Sig |
| Untargeted | StratSamp | 1.19 | 1.04 | | -0.01 | 1.90 | | -1.51 | 0.88 | | 3.63 | 2.39 | |
| | PAM | -1.04 | 1.25 | | 0.02 | 2.00 | | -0.85 | 0.99 | | 4.06 | 3.16 | |
| | Rscore | -0.66 | 0.89 | | -2.32 | 1.75 | | 0.61 | 0.76 | | 6.67 | 2.58 | ** |
| | CDmean | 1.31 | 1.19 | | 0.84 | 1.74 | | 1.41 | 0.64 | * | 0.87 | 2.59 | |
| | OvClustCDmean | 0.43 | 1.20 | | 0.79 | 1.79 | | 0.25 | 0.82 | | 1.21 | 2.88 | |
| | WIClustCDmean | 0.21 | 1.11 | | 0.43 | 1.76 | | 2.14 | 0.71 | ** | 0.62 | 2.83 | |
| | Avg_GRM | -16.59 | 1.77 | *** | -20.37 | 2.15 | *** | -12.49 | 1.13 | *** | -11.07 | 3.06 | *** |
| | Avg_GRM_MinMax | 1.64 | 1.17 | | 1.30 | 1.80 | | 0.37 | 0.74 | | 0.49 | 2.85 | |
| | Avg_GRM_self | 1.32 | 1.14 | | 1.10 | 1.85 | | -0.14 | 0.88 | | 1.07 | 2.80 | |
| Targeted | Avg_GRM | -5.68 | 1.67 | ** | -5.07 | 1.85 | ** | -4.47 | 1.12 | *** | -3.04 | 2.74 | |
| | Avg_GRM_MinMax | 2.84 | 1.37 | * | 2.90 | 2.10 | | 1.59 | 0.70 | * | 1.66 | 2.69 | |
| | Rscore | 0.22 | 1.13 | | -2.28 | 1.77 | | 0.85 | 0.74 | | 5.42 | 3.26 | |
| | CDmean | 5.74 | 1.40 | *** | 8.27 | 2.22 | ** | 3.14 | 0.78 | *** | 2.59 | 2.97 | |
| | OvClustCDmean | 4.69 | 1.32 | ** | 5.59 | 1.98 | * | 3.29 | 0.91 | ** | 5.24 | 2.56 | |
| | WIClustCDmean | 6.15 | 1.51 | *** | 8.82 | 2.30 | *** | 4.71 | 0.87 | *** | 3.78 | 2.79 | |

**Table S43** Percentage of gain in Area Under the Curve (AUC) for every optimization method and trait compared to random sampling using the RKHS model in the spruce dataset. Area under the curve was generated using agricolae package. AUC is the area under the curves generated when plotting the accuracy of the model against the training set size. As no optimization was performed when using the entire candidate set, results in the table shows the AUC for training set size from 10 to 80% of the candidate set. We present the mean and the standard error of the mean (SEM) over the 40 repetitions. Asterisks represent whether or not there is significant difference between the AUC for the training set optimization method and random sampling in a Wilcoxon signed-rank test. Sig: Significance. * P < 0.05, ** P < 0.01, * * * P < 0.001.

**Spruce RKHS**

| | | % of gain in AUC | | | | | | | | | | | |
|---|---|---|---|---|---|---|---|---|---|---|---|---|---|
| | | HT | | | DBH | | | DE | | | Simulated | | |
| Type | Algorithm | Mean | SEM | Sig | Mean | SEM | Sig | Mean | SEM | Sig | Mean | SEM | Sig |
| Untargeted | StratSamp | 0.68 | 0.91 | | -0.44 | 1.73 | | -1.30 | 0.84 | | 3.65 | 2.37 | |
| | PAM | -1.42 | 1.20 | | 0.08 | 1.89 | | -0.48 | 0.92 | | 4.02 | 3.14 | |
| | Rscore | -1.13 | 0.86 | | -1.94 | 1.68 | | 0.68 | 0.73 | | 6.71 | 2.64 | ** |
| | CDmean | 0.68 | 1.10 | | 0.30 | 1.60 | | 1.45 | 0.59 | * | 0.68 | 2.60 | |
| | OvClustCDmean | -0.33 | 1.14 | | -0.02 | 1.70 | | 0.57 | 0.79 | | 1.22 | 2.87 | |
| | WIClustCDmean | -0.56 | 1.06 | | -0.33 | 1.77 | | 2.33 | 0.66 | *** | 0.62 | 2.85 | |
| | Avg_GRM | -15.05 | 1.61 | *** | -18.08 | 1.84 | *** | -11.76 | 1.09 | *** | -11.30 | 3.10 | *** |
| | Avg_GRM_MinMax | 1.06 | 1.12 | | 1.11 | 1.67 | | 0.48 | 0.68 | | 0.42 | 2.87 | |
| | Avg_GRM_self | 0.81 | 1.05 | | 0.45 | 1.68 | | -0.03 | 0.87 | | 1.11 | 2.84 | |
| Targeted | Avg_GRM | -5.69 | 1.54 | *** | -4.02 | 1.69 | * | -4.32 | 1.09 | *** | -2.93 | 2.76 | |
| | Avg_GRM_MinMax | 2.13 | 1.17 | | 2.13 | 1.88 | | 1.51 | 0.66 | * | 1.59 | 2.72 | |
| | Rscore | -0.18 | 1.06 | | -2.37 | 1.61 | | 1.03 | 0.74 | | 5.26 | 3.25 | |
| | CDmean | 4.91 | 1.31 | *** | 7.13 | 1.97 | ** | 3.23 | 0.74 | *** | 2.43 | 2.99 | |
| | OvClustCDmean | 4.08 | 1.24 | ** | 5.09 | 1.76 | * | 3.23 | 0.87 | *** | 5.08 | 2.56 | |
| | WIClustCDmean | 5.35 | 1.38 | *** | 7.93 | 2.04 | *** | 4.60 | 0.83 | *** | 3.72 | 2.79 | |

**Table S44** Percentage of gain in Area Under the Curve (AUC) for every optimization method and trait compared to random sampling using the GBLUP model in the sorghum dataset. Area under the curve was generated using agricolae package. AUC is the area under the curves generated when plotting the accuracy of the model against the training set size. As no optimization was performed when using the entire candidate set, results in the table shows the AUC for training set size from 10 to 80% of the candidate set. We present the mean and the standard error of the mean (SEM) over the 40 repetitions. Asterisks represent whether or not there is significant different between the AUC for the training set optimization method and random sampling in a Wilcoxon signed-rank test. Sig: Significance. $*$ P $<$ 0.05, $**$ P $<$ 0.01, $***$ P $<$ 0.001.

**Sorghum GBLUP**

| | | % of gain in AUC | | | | | | | | | | | |
|---|---|---|---|---|---|---|---|---|---|---|---|---|---|
| | | YLD | | | HT | | | MO | | | Simulated | | |
| Type | Algorithm | Mean | SEM | Sig | Mean | SEM | Sig | Mean | SEM | Sig | Mean | SEM | Sig |
| Untargeted | StratSamp | 4.21 | 1.94 | * | -0.08 | 0.94 | | 0.45 | 0.97 | | 3.46 | 7.77 | |
| | PAM | -4.13 | 2.77 | | -0.48 | 1.02 | | 6.33 | 1.60 | *** | 1.80 | 7.30 | |
| | Rscore | 0.47 | 2.54 | | 0.19 | 1.01 | | 0.00 | 1.08 | | -0.31 | 7.25 | |
| | CDmean | -0.85 | 1.82 | | 1.12 | 0.99 | | 1.37 | 1.23 | | 0.89 | 8.25 | |
| | OvClustCDmean | -0.59 | 2.13 | | 1.16 | 0.85 | | -0.45 | 1.36 | | -3.06 | 7.37 | |
| | WIClustCDmean | 1.02 | 2.65 | | 1.26 | 1.12 | | -0.90 | 1.65 | | -4.02 | 7.56 | |
| | Avg_GRM | -42.01 | 7.84 | *** | -29.09 | 2.93 | *** | -31.05 | 2.76 | *** | -33.76 | 7.62 | *** |
| | Avg_GRM_MinMax | 6.05 | 1.88 | ** | 3.43 | 0.80 | *** | 2.09 | 1.09 | * | -3.10 | 7.79 | |
| | Avg_GRM_self | 5.99 | 2.10 | * | 4.91 | 1.00 | *** | 5.17 | 1.36 | *** | -2.29 | 7.79 | |
| Targeted | Avg_GRM | -15.25 | 4.75 | ** | -10.66 | 2.32 | *** | -13.26 | 2.73 | *** | -16.95 | 8.18 | |
| | Avg_GRM_MinMax | 11.16 | 2.16 | *** | 8.18 | 1.02 | *** | 7.09 | 1.24 | *** | 0.57 | 7.59 | |
| | Rscore | 8.47 | 1.76 | *** | 5.96 | 0.86 | *** | 6.17 | 1.30 | *** | 3.55 | 7.89 | |
| | CDmean | 14.08 | 2.35 | *** | 10.52 | 1.08 | *** | 7.57 | 1.29 | *** | 4.44 | 6.92 | |
| | OvClustCDmean | 13.17 | 2.34 | *** | 10.77 | 1.16 | *** | 7.39 | 1.30 | *** | 2.82 | 6.11 | |
| | WIClustCDmean | 11.90 | 2.49 | *** | 10.52 | 1.27 | *** | 7.44 | 1.34 | *** | 2.14 | 7.85 | |

**Table S45** Percentage of gain in Area Under the Curve (AUC) for every optimization method and trait compared to random sampling using the BayesB model in the sorghum dataset. Area under the curve was generated using agricolae package. AUC is the area under the curves generated when plotting the accuracy of the model against the training set size. As no optimization was performed when using the entire candidate set, results in the table shows the AUC for training set size from 10 to 80% of the candidate set. We present the mean and the standard error of the mean (SEM) over the 40 repetitions. Asterisks represent whether or not there is significant difference between the AUC for the training set optimization method and random sampling in a Wilcoxon signed-rank test. Sig: Significance. $*$ P $<$ 0.05, $**$ P $<$ 0.01, $***$ P $<$ 0.001.

**Sorghum BayesB**

| | | % of gain in AUC | | | | | | | | | | | |
|---|---|---|---|---|---|---|---|---|---|---|---|---|---|
| | | YLD | | | HT | | | MO | | | Simulated | | |
| Type | Algorithm | Mean | SEM | Sig | Mean | SEM | Sig | Mean | SEM | Sig | Mean | SEM | Sig |
| Untargeted | StratSamp | 4.19 | 2.22 | | -0.51 | 0.82 | | 0.60 | 0.93 | | 2.83 | 7.62 | |
| | PAM | -5.70 | 2.78 | | -0.22 | 1.03 | | 5.50 | 1.55 | ** | 0.98 | 7.17 | |
| | Rscore | 0.01 | 2.73 | | -0.04 | 0.96 | | 0.02 | 1.11 | | -0.36 | 7.08 | |
| | CDmean | -0.10 | 1.80 | | 1.39 | 1.06 | | 1.79 | 1.11 | | 0.67 | 7.88 | |
| | OvClustCDmean | -0.04 | 2.36 | | 0.88 | 0.85 | | 0.16 | 1.29 | | -3.02 | 7.24 | |
| | WIClustCDmean | 0.90 | 2.94 | | 1.46 | 1.06 | | 0.20 | 1.52 | | -3.71 | 7.43 | |
| | Avg_GRM | -44.20 | 7.80 | *** | -28.85 | 2.94 | *** | -31.36 | 2.78 | *** | -32.78 | 7.52 | *** |
| | Avg_GRM_MinMax | 6.39 | 2.05 | ** | 3.33 | 0.77 | *** | 2.01 | 1.06 | * | -2.80 | 7.67 | |
| | Avg_GRM_self | 6.33 | 2.17 | ** | 4.97 | 0.96 | *** | 4.89 | 1.26 | *** | -2.21 | 7.72 | |
| Targeted | Avg_GRM | -14.82 | 4.14 | ** | -10.67 | 2.34 | *** | -13.32 | 2.74 | *** | -15.77 | 8.07 | |
| | Avg_GRM_MinMax | 11.27 | 2.27 | *** | 8.13 | 0.97 | *** | 7.01 | 1.24 | *** | 0.90 | 7.53 | |
| | Rscore | 7.88 | 1.88 | *** | 6.08 | 0.79 | *** | 5.83 | 1.29 | *** | 3.34 | 7.58 | |
| | CDmean | 13.62 | 2.44 | *** | 10.20 | 1.04 | *** | 7.20 | 1.30 | *** | 4.01 | 6.72 | |
| | OvClustCDmean | 13.72 | 2.43 | *** | 10.38 | 1.10 | *** | 7.13 | 1.31 | *** | 1.80 | 5.91 | |
| | WIClustCDmean | 12.34 | 2.65 | *** | 10.43 | 1.20 | *** | 7.19 | 1.38 | *** | 2.09 | 7.66 | |

**Table S46** Percentage of gain in Area Under the Curve (AUC) for every optimization method and trait compared to random sampling using the RKHS model in the sorghum dataset. Area under the curve was generated using agricolae package. AUC is the area under the curves generated when plotting the accuracy of the model against the training set size. As no optimization was performed when using the entire candidate set, results in the table shows the AUC for training set size from 10 to 80% of the candidate set. We present the mean and the standard error of the mean (SEM) over the 40 repetitions. Asterisks represent whether or not there is significant difference between the AUC for the training set optimization method and random sampling in a Wilcoxon signed-rank test. Sig: Significance. $*$ P $<$ 0.05, $**$ P $<$ 0.01, $***$ P $<$ 0.001.

**Sorghum RKHS**

| | | % of gain in AUC | | | | | | | | | | | |
|---|---|---|---|---|---|---|---|---|---|---|---|---|---|
| | | YLD | | | HT | | | MO | | | Simulated | | |
| Type | Algorithm | Mean | SEM | Sig | Mean | SEM | Sig | Mean | SEM | Sig | Mean | SEM | Sig |
| Untargeted | StratSamp | 3.79 | 2.06 | | -0.37 | 0.94 | | 0.36 | 1.03 | | 3.65 | 7.81 | |
| | PAM | -4.41 | 2.89 | | 0.17 | 1.05 | | 6.37 | 1.65 | *** | 1.13 | 7.37 | |
| | Rscore | 0.15 | 2.50 | | 0.16 | 1.03 | | -0.38 | 1.10 | | -0.17 | 7.32 | |
| | CDmean | -0.48 | 1.84 | | 1.72 | 1.07 | | 1.03 | 1.31 | | 1.10 | 8.16 | |
| | OvClustCDmean | -0.66 | 2.12 | | 1.55 | 0.92 | | -0.82 | 1.43 | | -3.74 | 7.39 | |
| | WIClustCDmean | 2.24 | 2.75 | | 1.45 | 1.09 | | -1.29 | 1.73 | | -3.57 | 7.51 | |
| | Avg_GRM | -39.13 | 7.21 | *** | -28.28 | 2.98 | *** | -30.23 | 2.65 | *** | -32.98 | 7.49 | *** |
| | Avg_GRM_MinMax | 5.78 | 1.92 | ** | 3.67 | 0.81 | *** | 2.19 | 1.09 | * | -2.35 | 7.84 | |
| | Avg_GRM_self | 5.91 | 2.10 | * | 5.40 | 1.06 | *** | 4.96 | 1.38 | ** | -1.44 | 7.83 | |
| Targeted | Avg_GRM | -13.16 | 4.25 | ** | -10.82 | 2.25 | *** | -12.32 | 2.60 | *** | -15.82 | 8.10 | |
| | Avg_GRM_MinMax | 10.55 | 2.23 | *** | 8.49 | 1.02 | *** | 6.95 | 1.26 | *** | 1.18 | 7.58 | |
| | Rscore | 8.20 | 1.84 | *** | 6.66 | 0.90 | *** | 5.74 | 1.33 | *** | 3.75 | 7.86 | |
| | CDmean | 12.79 | 2.41 | *** | 10.28 | 1.10 | *** | 7.53 | 1.32 | *** | 4.67 | 6.93 | |
| | OvClustCDmean | 12.22 | 2.43 | *** | 10.61 | 1.16 | *** | 7.51 | 1.33 | *** | 2.63 | 6.13 | |
| | WIClustCDmean | 11.57 | 2.58 | *** | 10.40 | 1.27 | *** | 7.57 | 1.38 | *** | 2.67 | 7.87 | |

**Table S47** Percentage of gain in Area Under the Curve (AUC) for every optimization method and trait compared to random sampling using the GBLUP model in the switchgrass dataset. Area under the curve was generated using agricolae package. AUC is the area under the curves generated when plotting the accuracy of the model against the training set size. As no optimization was performed when using the entire candidate set, results in the table shows the AUC for training set size from 10 to 80% of the candidate set. We present the mean and the standard error of the mean (SEM) over the 40 repetitions. Asterisks represent whether or not there is significant difference between the AUC for the training set optimization method and random sampling in a Wilcoxon signed-rank test. Sig: Significance. $*$ P $<$ 0.05, $**$ P $<$ 0.01, $***$ P $<$ 0.001.

**Switchgrass GBLUP**

| | | % of gain in AUC | | | | | | | | | | | |
|---|---|---|---|---|---|---|---|---|---|---|---|---|---|
| | | AN | | | HT | | | ST | | | Simulated | | |
| Type | Algorithm | Mean | SEM | Sig | Mean | SEM | Sig | Mean | SEM | Sig | Mean | SEM | Sig |
| Untargeted | StratSamp | 0.07 | 0.36 | | -0.55 | 0.54 | | 0.37 | 0.38 | | 3.55 | 7.62 | |
| | PAM | 0.66 | 0.36 | | 0.88 | 0.63 | | 1.52 | 0.46 | ** | 0.48 | 9.11 | |
| | Rscore | 0.28 | 0.41 | | 0.45 | 0.51 | | 0.37 | 0.38 | | 1.92 | 8.03 | |
| | CDmean | 1.14 | 0.33 | ** | -0.07 | 0.70 | | -0.02 | 0.38 | | -6.91 | 9.00 | |
| | OvClustCDmean | 1.33 | 0.37 | *** | -1.11 | 0.68 | | 0.36 | 0.44 | | 11.62 | 9.56 | |
| | WIClustCDmean | 1.72 | 0.34 | *** | -0.60 | 0.57 | | 0.79 | 0.41 | | 11.11 | 6.29 | |
| | Avg_GRM | -35.87 | 1.98 | *** | -31.68 | 3.31 | *** | -36.25 | 2.08 | *** | -31.35 | 8.26 | ** |
| | Avg_GRM_MinMax | 1.81 | 0.43 | *** | -0.34 | 0.63 | | 1.73 | 0.36 | *** | 12.50 | 6.44 | |
| | Avg_GRM_self | 1.95 | 0.40 | *** | 0.12 | 0.57 | | 2.33 | 0.34 | *** | 11.74 | 6.39 | |
| Targeted | Avg_GRM | -16.39 | 1.67 | *** | -17.83 | 2.65 | *** | -21.95 | 1.86 | *** | -15.01 | 7.41 | * |
| | Avg_GRM_MinMax | 3.35 | 0.50 | *** | 0.42 | 0.77 | | 3.32 | 0.37 | *** | 12.75 | 6.22 | |
| | Rscore | 1.33 | 0.54 | ** | -0.09 | 0.70 | | 2.29 | 0.41 | *** | 10.27 | 8.67 | |
| | CDmean | 0.59 | 0.56 | | 0.79 | 0.66 | | 2.80 | 0.40 | *** | 7.56 | 9.02 | |
| | OvClustCDmean | 1.99 | 0.58 | *** | 0.96 | 0.68 | | 3.01 | 0.36 | *** | 12.31 | 8.80 | |
| | WIClustCDmean | 2.59 | 0.56 | *** | 0.86 | 0.71 | | 3.07 | 0.37 | *** | 14.86 | 6.28 | |

**Table S48** Percentage of gain in Area Under the Curve (AUC) for every optimization method and trait compared to random sampling using the BayesB model in the switchgrass dataset. Area under the curve was generated using agricolae package. AUC is the area under the curves generated when plotting the accuracy of the model against the training set size. As no optimization was performed when using the entire candidate set, results in the table shows the AUC for training set size from 10 to 80% of the candidate set. We present the mean and the standard error of the mean (SEM) over the 40 repetitions. Asterisks represent whether or not there is significant difference between the AUC for the training set optimization method and random sampling in a Wilcoxon signed-rank test. Sig: Significance. $*$ P $<$ 0.05, $**$ P $<$ 0.01, $***$ P $<$ 0.001.

**Switchgrass BayesB**

| | | % of gain in AUC | | | | | | | | | | | |
|---|---|---|---|---|---|---|---|---|---|---|---|---|---|
| | | AN | | | HT | | | ST | | | Simulated | | |
| Type | Algorithm | Mean | SEM | Sig | Mean | SEM | Sig | Mean | SEM | Sig | Mean | SEM | Sig |
| Untargeted | StratSamp | 0.00 | 0.33 | | -0.56 | 0.51 | | 0.29 | 0.36 | | 3.75 | 7.17 | |
| | PAM | -0.77 | 0.33 | ** | 0.35 | 0.60 | | 1.55 | 0.40 | ** | 1.70 | 8.45 | |
| | Rscore | 0.15 | 0.36 | | 0.53 | 0.47 | | 0.35 | 0.33 | | 0.94 | 7.88 | |
| | CDmean | 1.30 | 0.30 | *** | -0.01 | 0.63 | | -0.38 | 0.37 | | -6.77 | 8.63 | |
| | OvClustCDmean | 1.55 | 0.32 | *** | -0.92 | 0.62 | | 0.28 | 0.39 | | 11.71 | 9.29 | |
| | WIClustCDmean | 1.72 | 0.30 | *** | -0.51 | 0.54 | | 0.62 | 0.38 | | 9.44 | 6.07 | |
| | Avg_GRM | -34.78 | 1.92 | *** | -32.50 | 3.45 | *** | -36.50 | 2.17 | *** | -31.24 | 8.13 | *** |
| | Avg_GRM_MinMax | 1.83 | 0.36 | *** | -0.08 | 0.56 | | 1.79 | 0.32 | *** | 10.95 | 6.30 | |
| | Avg_GRM_self | 1.99 | 0.33 | *** | 0.54 | 0.50 | | 2.35 | 0.32 | *** | 11.04 | 6.09 | |
| Targeted | Avg_GRM | -15.26 | 1.60 | *** | -17.84 | 2.80 | *** | -21.24 | 1.81 | *** | -16.14 | 7.59 | * |
| | Avg_GRM_MinMax | 3.47 | 0.44 | *** | 0.97 | 0.70 | * | 3.37 | 0.36 | *** | 12.72 | 5.99 | |
| | Rscore | 1.71 | 0.48 | *** | 0.22 | 0.64 | | 2.31 | 0.38 | *** | 9.44 | 8.16 | |
| | CDmean | 1.09 | 0.49 | * | 1.06 | 0.62 | | 2.94 | 0.37 | *** | 8.50 | 8.34 | |
| | OvClustCDmean | 2.63 | 0.53 | *** | 1.16 | 0.63 | | 3.13 | 0.35 | *** | 12.26 | 8.43 | |
| | WIClustCDmean | 3.19 | 0.50 | *** | 1.11 | 0.68 | | 3.25 | 0.35 | *** | 13.90 | 6.14 | |

**Table S49** Percentage of gain in Area Under the Curve (AUC) for every optimization method and trait compared to random sampling using the RKHS model in the switchgrass dataset. Area under the curve was generated using agricolae package. AUC is the area under the curves generated when plotting the accuracy of the model against the training set size. As no optimization was performed when using the entire candidate set, results in the table shows the AUC for training set size from 10 to 80% of the candidate set. We present the mean and the standard error of the mean (SEM) over the 40 repetitions. Asterisks represent whether or not there is significant difference between the AUC for the training set optimization method and random sampling in a Wilcoxon signed-rank test. Sig: Significance. $*$ P $<$ 0.05, $**$ P $<$ 0.01, $***$ P $<$ 0.001.

**Switchgrass RKHS**

| | | % of gain in AUC | | | | | | | | | | | |
|---|---|---|---|---|---|---|---|---|---|---|---|---|---|
| | | AN | | | HT | | | ST | | | Simulated | | |
| Type | Algorithm | Mean | SEM | Sig | Mean | SEM | Sig | Mean | SEM | Sig | Mean | SEM | Sig |
| Untargeted | StratSamp | 0.07 | 0.36 | | -0.53 | 0.54 | | 0.39 | 0.38 | | 3.67 | 7.55 | |
| | PAM | 0.64 | 0.36 | | 0.91 | 0.64 | | 1.52 | 0.46 | ** | 0.54 | 9.05 | |
| | Rscore | 0.28 | 0.41 | | 0.48 | 0.51 | | 0.37 | 0.38 | | 1.79 | 8.00 | |
| | CDmean | 1.13 | 0.33 | ** | -0.07 | 0.70 | | -0.04 | 0.38 | | -6.80 | 8.91 | |
| | OvClustCDmean | 1.33 | 0.37 | *** | -1.11 | 0.68 | | 0.34 | 0.44 | | 11.50 | 9.51 | |
| | WIClustCDmean | 1.72 | 0.34 | *** | -0.63 | 0.57 | | 0.78 | 0.41 | | 10.96 | 6.25 | |
| | Avg_GRM | -35.83 | 1.98 | *** | -32.50 | 3.37 | *** | -36.67 | 2.13 | *** | -31.33 | 8.26 | ** |
| | Avg_GRM_MinMax | 1.81 | 0.43 | *** | -0.33 | 0.64 | | 1.74 | 0.36 | *** | 12.42 | 6.38 | |
| | Avg_GRM_self | 1.95 | 0.39 | *** | 0.16 | 0.56 | | 2.34 | 0.34 | *** | 11.62 | 6.35 | |
| Targeted | Avg_GRM | -16.37 | 1.66 | *** | -18.27 | 2.72 | *** | -21.95 | 1.86 | *** | -15.23 | 7.41 | * |
| | Avg_GRM_MinMax | 3.36 | 0.50 | *** | 0.42 | 0.77 | | 3.32 | 0.37 | *** | 12.75 | 6.14 | |
| | Rscore | 1.33 | 0.54 | ** | -0.12 | 0.71 | | 2.28 | 0.41 | *** | 10.28 | 8.56 | |
| | CDmean | 0.60 | 0.56 | | 0.79 | 0.65 | | 2.79 | 0.40 | *** | 7.56 | 8.94 | |
| | OvClustCDmean | 1.99 | 0.58 | *** | 0.96 | 0.68 | | 3.00 | 0.36 | *** | 12.30 | 8.74 | |
| | WIClustCDmean | 2.60 | 0.56 | *** | 0.86 | 0.71 | | 3.07 | 0.37 | *** | 14.78 | 6.26 | |

**Table S50** Percentage of gain in Area Under the Curve (AUC) for every optimization method and trait compared to random sampling using the GBLUP model in the ricePopStr dataset. Area under the curve was generated using agricolae package. AUC is the area under the curves generated when plotting the accuracy of the model against the training set size. As no optimization was performed when using the entire candidate set, results in the table shows the AUC for training set size from 10 to 80% of the candidate set. We present the mean and the standard error of the mean (SEM) over the 40 repetitions. Asterisks represent whether or not there is significant difference between the AUC for the training set optimization method and random sampling in a Wilcoxon signed-rank test. Sig: Significance. $*$ P $<$ 0.05, $**$ P $<$ 0.01, $***$ P $<$ 0.001.

RicePopStr GBLUP

| | | % of gain in AUC | | | | | | | | | | | | | | |
|---|---|---|---|---|---|---|---|---|---|---|---|---|---|---|---|---|
| | | FP | | | FT | | | HT | | | PC | | | Simulated | | |
| Type | Algorithm | Mean | SEM | Sig | Mean | SEM | Sig | Mean | SEM | Sig | Mean | SEM | Sig | Mean | SEM | Sig |
| Untargeted | StratSamp | -0.63 | 1.24 | | -1.27 | 0.94 | | -0.50 | 0.52 | | -1.55 | 1.29 | | -0.25 | 0.86 | |
| | PAM | -1.56 | 1.60 | | -1.15 | 1.02 | | 3.55 | 0.59 | *** | -14.23 | 1.55 | *** | -1.27 | 1.60 | |
| | Rscore | -2.24 | 1.02 | * | -3.56 | 1.00 | ** | 2.51 | 0.64 | *** | -8.00 | 1.32 | *** | -1.07 | 0.79 | |
| | CDmean | 1.66 | 1.11 | | -3.51 | 1.05 | ** | 3.29 | 0.57 | *** | -9.42 | 1.12 | *** | 0.78 | 1.22 | |
| | OvClustCDmean | -0.38 | 0.99 | | -5.44 | 0.97 | *** | 0.54 | 0.51 | | -1.31 | 1.04 | | -0.01 | 1.29 | |
| | WIClustCDmean | 4.39 | 0.97 | *** | -1.43 | 0.96 | | 0.54 | 0.56 | | 0.01 | 1.38 | | -0.75 | 1.01 | |
| | Avg_GRM | -33.99 | 2.07 | *** | -38.22 | 3.48 | *** | -35.50 | 3.53 | *** | -49.55 | 4.86 | *** | -39.35 | 6.30 | *** |
| | Avg_GRM_MinMax | 3.61 | 0.91 | *** | 2.05 | 0.89 | * | 3.10 | 0.58 | *** | 2.76 | 1.12 | * | 1.37 | 0.94 | |
| | Avg_GRM_self | 4.84 | 1.00 | *** | 3.81 | 1.04 | *** | 3.95 | 0.53 | *** | 3.56 | 1.29 | * | 2.63 | 1.23 | * |
| Targeted | Avg_GRM | -17.77 | 2.04 | *** | -24.73 | 2.93 | *** | -21.82 | 2.89 | *** | -42.87 | 5.05 | *** | -24.51 | 3.70 | *** |
| | Avg_GRM_MinMax | 6.25 | 1.26 | *** | 5.85 | 1.00 | *** | 5.64 | 0.59 | *** | 2.85 | 1.44 | | 1.77 | 1.63 | |
| | Rscore | 4.39 | 1.21 | ** | 4.22 | 1.05 | *** | 5.22 | 0.59 | *** | 2.09 | 1.58 | | 2.03 | 1.60 | |
| | CDmean | 7.16 | 1.13 | *** | 6.61 | 1.03 | *** | 6.98 | 0.61 | *** | 7.51 | 1.76 | *** | 4.10 | 1.70 | ** |
| | OvClustCDmean | 7.21 | 1.28 | *** | 7.42 | 1.04 | *** | 6.83 | 0.58 | *** | 7.42 | 1.57 | *** | 4.53 | 1.98 | * |
| | WIClustCDmean | 8.18 | 1.25 | *** | 6.02 | 0.97 | *** | 5.30 | 0.56 | *** | 8.75 | 1.75 | *** | 3.61 | 1.75 | * |

**Table S51** Percentage of gain in Area Under the Curve (AUC) for every optimization method and trait compared to random sampling using the BayesB model in the ricePopStr dataset. Area under the curve was generated using agricolae package. AUC is the area under the curves generated when plotting the accuracy of the model against the training set size. As no optimization was performed when using the entire candidate set, results in the table shows the AUC for training set size from 10 to 80% of the candidate set. We present the mean and the standard error of the mean (SEM) over the 40 repetitions. Asterisks represent whether or not there is significant difference between the AUC for the training set optimization method and random sampling in a Wilcoxon signed-rank test. Sig: Significance. $*$ P $<$ 0.05, $**$ P $<$ 0.01, $***$ P $<$ 0.001.

RicePopStr BayesB

| | | % of gain in AUC | | | | | | | | | | | | | | |
|---|---|---|---|---|---|---|---|---|---|---|---|---|---|---|---|---|
| | | FP | | | FT | | | HT | | | PC | | | Simulated | | |
| Type | Algorithm | Mean | SEM | Sig | Mean | SEM | Sig | Mean | SEM | Sig | Mean | SEM | Sig | Mean | SEM | Sig |
| Untargeted | StratSamp | -0.59 | 0.88 | | -0.85 | 0.85 | | -0.36 | 0.47 | | -1.65 | 1.35 | | 0.28 | 0.89 | |
| | PAM | 1.87 | 0.95 | | -1.01 | 0.82 | | 2.75 | 0.55 | *** | -7.87 | 1.45 | *** | 0.46 | 1.08 | |
| | Rscore | -0.87 | 0.86 | | -2.29 | 0.86 | * | 2.35 | 0.60 | *** | -6.47 | 1.39 | *** | 0.87 | 0.76 | |
| | CDmean | 1.83 | 0.80 | * | -2.34 | 0.88 | ** | 2.85 | 0.56 | *** | -4.27 | 1.22 | ** | 0.83 | 1.22 | |
| | OvClustCDmean | 0.54 | 0.76 | | -4.39 | 0.86 | *** | 0.28 | 0.51 | | -0.67 | 1.09 | | 1.76 | 1.24 | |
| | WIClustCDmean | 3.45 | 0.71 | *** | -0.37 | 0.80 | | 0.39 | 0.55 | | 0.68 | 1.35 | | 0.15 | 1.06 | |
| | Avg_GRM | -34.94 | 2.11 | *** | -39.01 | 3.42 | *** | -36.43 | 3.40 | *** | -50.76 | 4.61 | *** | -36.62 | 6.02 | *** |
| | Avg_GRM_MinMax | 4.09 | 0.65 | *** | 2.73 | 0.79 | *** | 2.95 | 0.54 | *** | 3.30 | 1.09 | ** | 1.92 | 1.10 | |
| | Avg_GRM_self | 5.02 | 0.66 | *** | 4.45 | 0.83 | *** | 3.93 | 0.48 | *** | 3.60 | 1.15 | ** | 2.66 | 1.08 | * |
| Targeted | Avg_GRM | -18.46 | 1.95 | *** | -24.59 | 2.66 | *** | -22.11 | 2.97 | *** | -43.51 | 4.83 | *** | -24.22 | 3.67 | *** |
| | Avg_GRM_MinMax | 6.66 | 0.76 | *** | 7.08 | 0.80 | *** | 5.70 | 0.54 | *** | 2.91 | 1.51 | | 1.88 | 1.46 | |
| | Rscore | 5.06 | 0.82 | *** | 4.96 | 0.94 | *** | 4.97 | 0.53 | *** | 2.43 | 1.35 | | 2.65 | 1.41 | |
| | CDmean | 8.31 | 0.91 | *** | 8.78 | 0.82 | *** | 7.29 | 0.56 | *** | 5.77 | 1.61 | ** | 4.09 | 1.58 | ** |
| | OvClustCDmean | 8.54 | 0.92 | *** | 9.44 | 0.78 | *** | 7.20 | 0.52 | *** | 6.41 | 1.50 | *** | 3.84 | 1.67 | * |
| | WIClustCDmean | 8.46 | 1.02 | *** | 7.97 | 0.77 | *** | 5.62 | 0.50 | *** | 6.65 | 1.57 | *** | 3.81 | 1.66 | * |

**Table S52** Percentage of gain in Area Under the Curve (AUC) for every optimization method and trait compared to random sampling using the RKHS model in the ricePopStr dataset. Area under the curve was generated using agricolae package. AUC is the area under the curves generated when plotting the accuracy of the model against the training set size. As no optimization was performed when using the entire candidate set, results in the table shows the AUC for training set size from 10 to 80% of the candidate set. We present the mean and the standard error of the mean (SEM) over the 40 repetitions. Asterisks represent whether or not there is significant difference between the AUC for the training set optimization method and random sampling in a Wilcoxon signed-rank test. Sig: Significance. $*$ $P < 0.05$, $**$ $P < 0.01$, $***$ $P < 0.001$.

RicePopStr RKHS

| | | % of gain in AUC | | | | | | | | | | | | | | |
|---|---|---|---|---|---|---|---|---|---|---|---|---|---|---|---|---|
| | | FP | | | FT | | | HT | | | PC | | | Simulated | | |
| Type | Algorithm | Mean | SEM | Sig | Mean | SEM | Sig | Mean | SEM | Sig | Mean | SEM | Sig | Mean | SEM | Sig |
| Untargeted | StratSamp | -0.04 | 0.94 | | -0.60 | 0.88 | | -0.41 | 0.54 | | -1.24 | 1.29 | | 0.04 | 0.77 | |
| | PAM | 1.02 | 1.49 | | 0.68 | 1.07 | | 3.82 | 0.63 | *** | -9.15 | 1.64 | *** | -0.82 | 1.43 | |
| | Rscore | -0.45 | 0.99 | | -2.44 | 0.97 | * | 2.70 | 0.66 | *** | -6.46 | 1.40 | *** | -0.45 | 0.79 | |
| | CDmean | 2.06 | 1.07 | | -1.14 | 0.91 | | 3.53 | 0.58 | *** | -5.17 | 1.19 | *** | 0.17 | 1.17 | |
| | OvClustCDmean | 0.75 | 0.92 | | -3.47 | 0.99 | ** | 0.81 | 0.54 | | 1.12 | 1.00 | | -0.18 | 1.30 | |
| | WIClustCDmean | 5.52 | 0.87 | *** | 0.91 | 1.10 | | 0.68 | 0.58 | | 1.55 | 1.41 | | -1.14 | 1.24 | |
| | Avg_GRM | -33.95 | 2.06 | *** | -34.61 | 3.23 | *** | -36.46 | 3.75 | *** | -40.26 | 3.52 | *** | -40.56 | 6.03 | *** |
| | Avg_GRM_MinMax | 4.68 | 0.89 | *** | 2.62 | 0.90 | ** | 3.13 | 0.59 | *** | 1.99 | 1.08 | | 1.29 | 1.15 | |
| | Avg_GRM_self | 5.99 | 0.92 | *** | 4.06 | 0.91 | *** | 3.97 | 0.56 | *** | 3.95 | 1.16 | ** | 2.79 | 1.11 | * |
| Targeted | Avg_GRM | -16.74 | 2.04 | *** | -23.04 | 2.86 | *** | -21.51 | 2.88 | *** | -33.26 | 3.68 | *** | -24.11 | 3.47 | *** |
| | Avg_GRM_MinMax | 7.34 | 1.01 | *** | 6.28 | 0.98 | *** | 5.70 | 0.61 | *** | 2.89 | 1.51 | | 2.11 | 1.52 | |
| | Rscore | 5.32 | 0.94 | *** | 4.94 | 0.96 | *** | 5.31 | 0.60 | *** | 1.93 | 1.31 | | 2.23 | 1.55 | |
| | CDmean | 7.62 | 1.02 | *** | 7.03 | 0.91 | *** | 7.04 | 0.62 | *** | 5.51 | 1.60 | ** | 4.21 | 1.60 | ** |
| | OvClustCDmean | 7.95 | 1.05 | *** | 7.63 | 0.92 | *** | 6.87 | 0.59 | *** | 6.16 | 1.52 | *** | 3.85 | 1.77 | * |
| | WIClustCDmean | 7.92 | 1.14 | *** | 6.51 | 0.86 | *** | 5.40 | 0.58 | *** | 6.23 | 1.54 | *** | 3.57 | 1.69 | * |

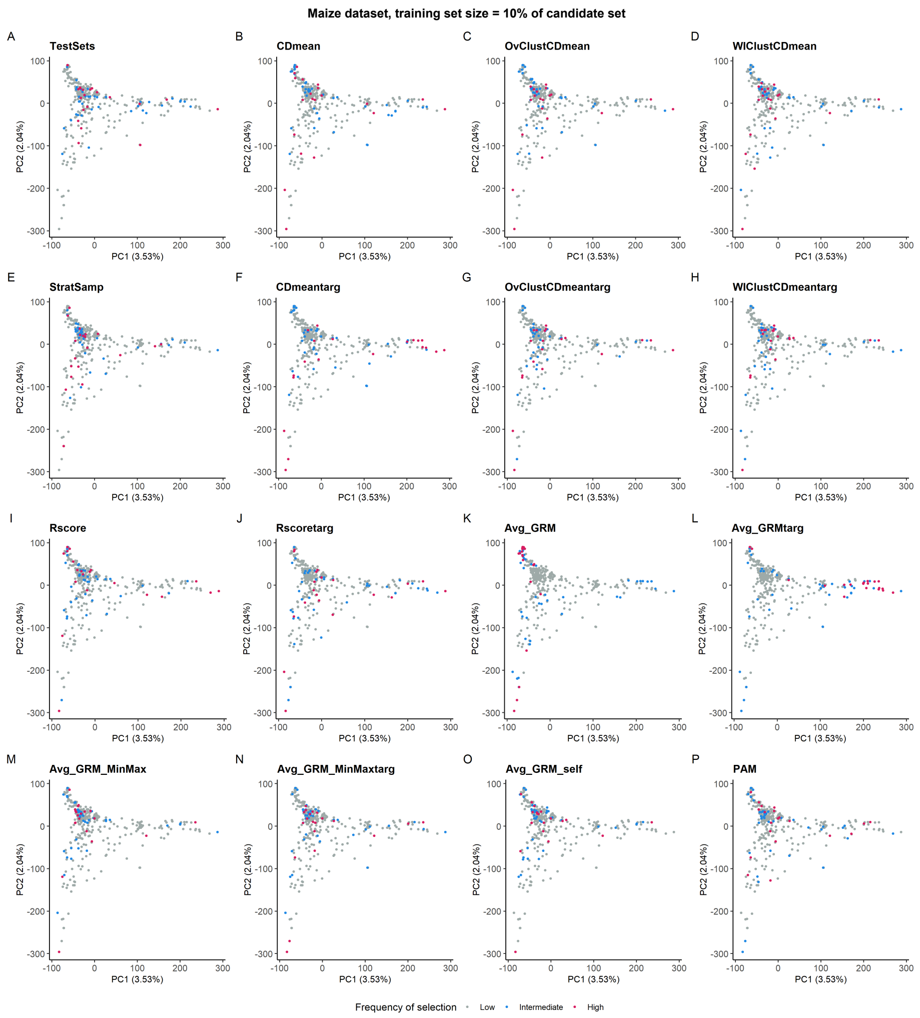


**Fig. S2** Frequency of selection of individuals for the training set across the 40 iterations for all training set optimization methods (plots B-P). If "targ" is added at the end of the name of a method, it corresponds to targeted optimization. Otherwise, untargeted optimization was performed. The frequency of the individuals randomly sampled for the test sets is also shown in plot A. All plots belong to the maize dataset with a training set size of 10% of the candidate set. The two axes in the plots are the first two principal components that summarize the genetic space and each point is an individual within the dataset. Red colour is used to highlight the 5% most frequently selected individuals, blue colour corresponds to the next 10% most selected individuals and grey is used for the rest.

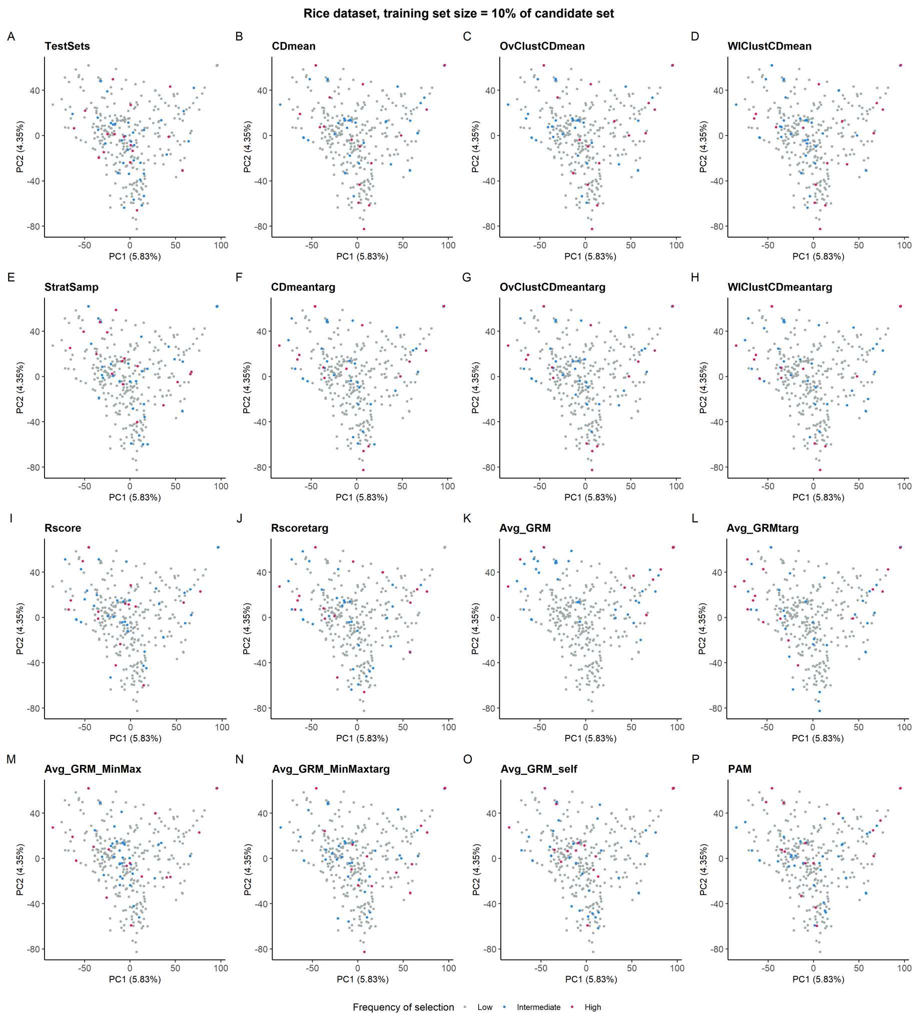


**Fig. S3** Frequency of selection of individuals for the training set across the 40 iterations for all training set optimization methods (plots B-P). If "targ" is added at the end of the name of a method, it corresponds to targeted optimization. Otherwise, untargeted optimization was performed. The frequency of the individuals randomly sampled for the test sets is also shown in plot A. All plots belong to the rice dataset with a training set size of 10% of the candidate set. The two axes in the plots are the first two principal components that summarize the genetic space and each point is an individual within the dataset. Red colour is used to highlight the 5% most frequently selected individuals, blue colour corresponds to the next 10% most selected individuals and grey is used for the rest.

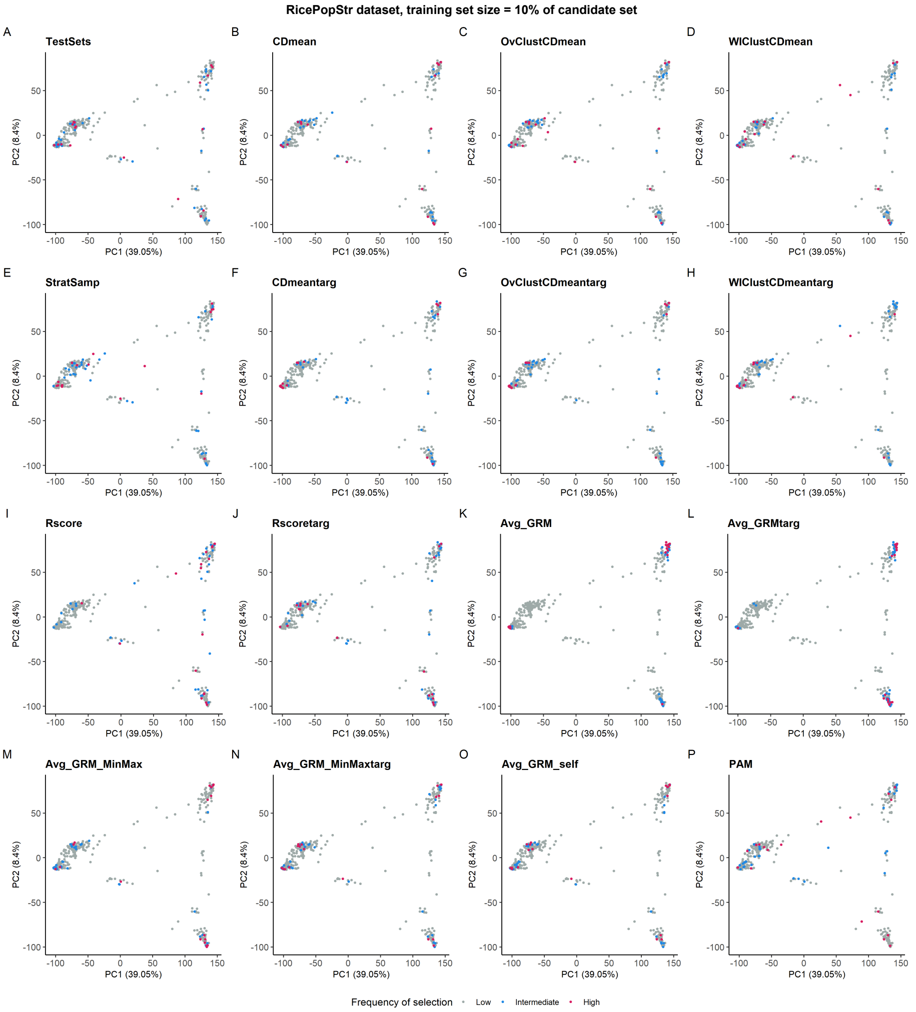


**Fig. S4** Frequency of selection of individuals for the training set across the 40 iterations for all training set optimization methods (plots B-P). If "targ" is added at the end of the name of a method, it corresponds to targeted optimization. Otherwise, untargeted optimization was performed. The frequency of the individuals randomly sampled for the test sets is also shown in plot A. All plots belong to the ricePopStr dataset with a training set size of 10% of the candidate set. The two axes in the plots are the first two principal components that summarize the genetic space and each point is an individual within the dataset. Red colour is used to highlight the 5% most frequently selected individuals, blue colour corresponds to the next 10% most selected individuals and grey is used for the rest.

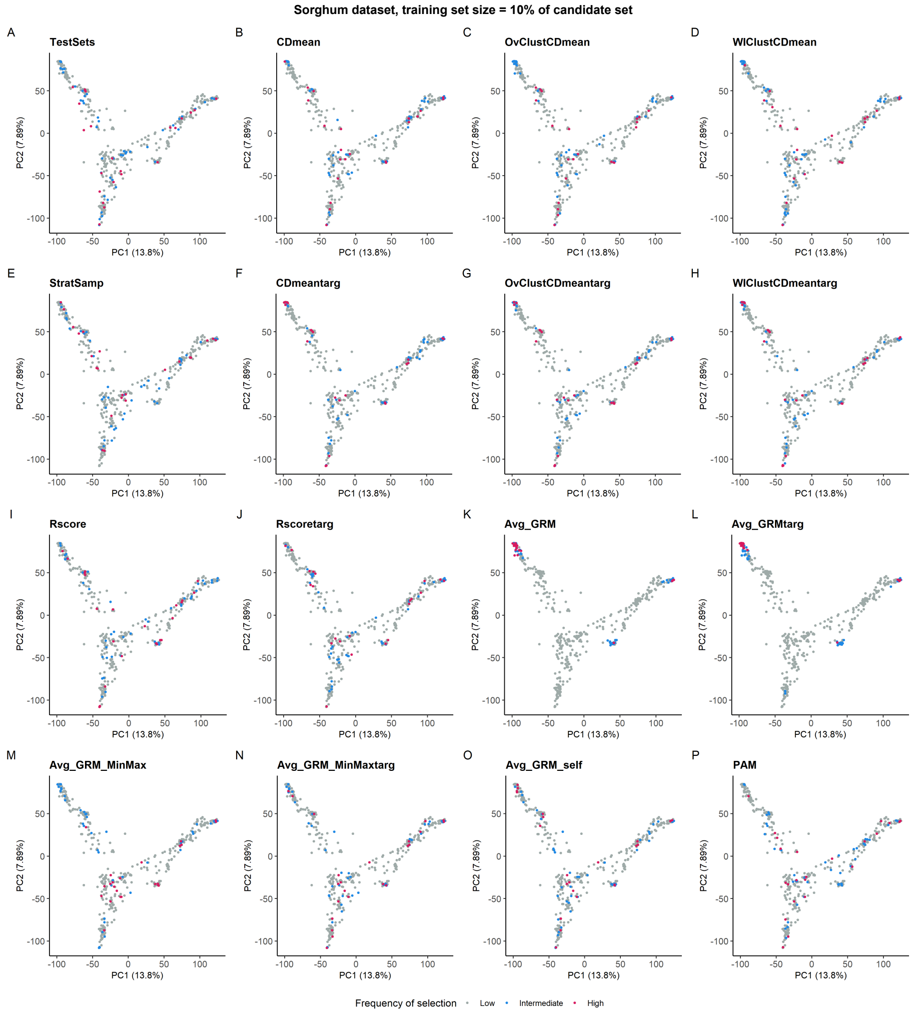


**Fig. S5** Frequency of selection of individuals for the training set across the 40 iterations for all training set optimization methods (plots B-P). If "targ" is added at the end of the name of a method, it corresponds to targeted optimization. Otherwise, untargeted optimization was performed. The frequency of the individuals randomly sampled for the test sets is also shown in plot A. All plots belong to the sorghum dataset with a training set size of 10% of the candidate set. The two axes in the plots are the first two principal components that summarize the genetic space and each point is an individual within the dataset. Red colour is used to highlight the 5% most frequently selected individuals, blue colour corresponds to the next 10% most selected individuals and grey is used for the rest.

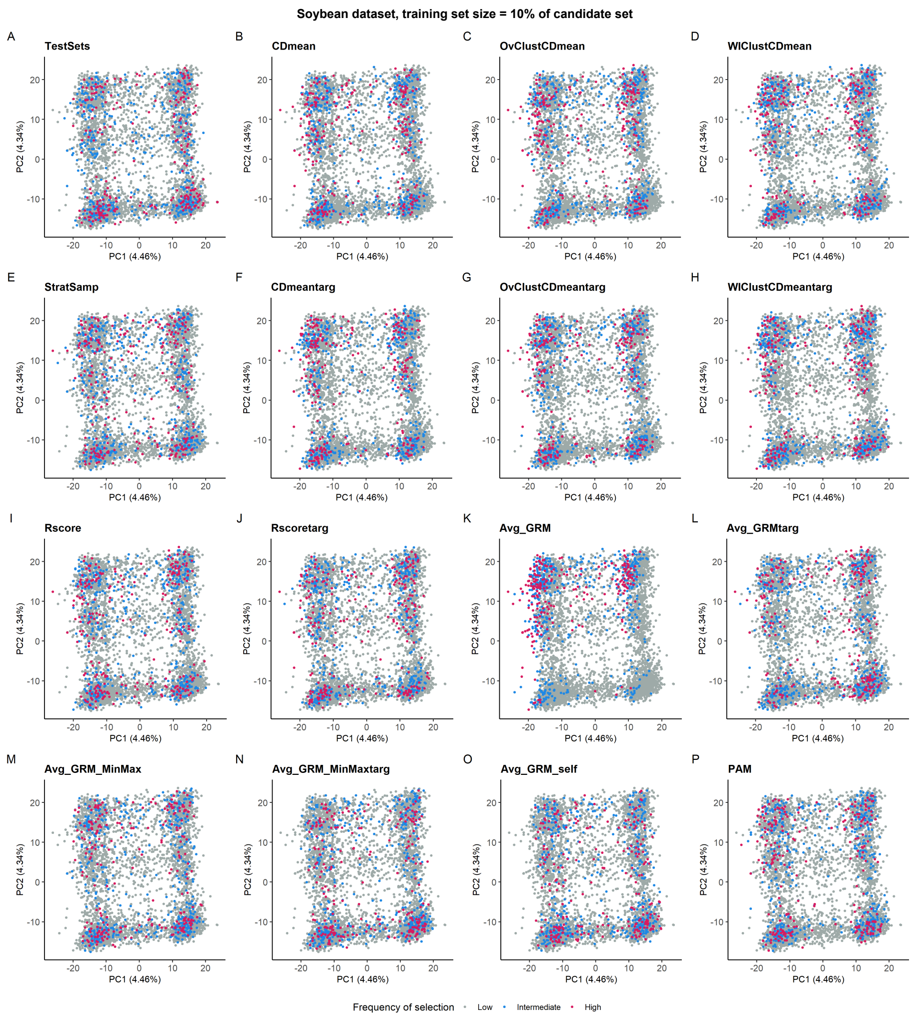


**Fig. S6** Frequency of selection of individuals for the training set across the 40 iterations for all training set optimization methods (plots B-P). If "targ" is added at the end of the name of a method, it corresponds to targeted optimization. Otherwise, untargeted optimization was performed. The frequency of the individuals randomly sampled for the test sets is also shown in plot A. All plots belong to the soybean dataset with a training set size of 10% of the candidate set. The two axes in the plots are the first two principal components that summarize the genetic space and each point is an individual within the dataset. Red colour is used to highlight the 5% most frequently selected individuals, blue colour corresponds to the next 10% most selected individuals and grey is used for the rest.

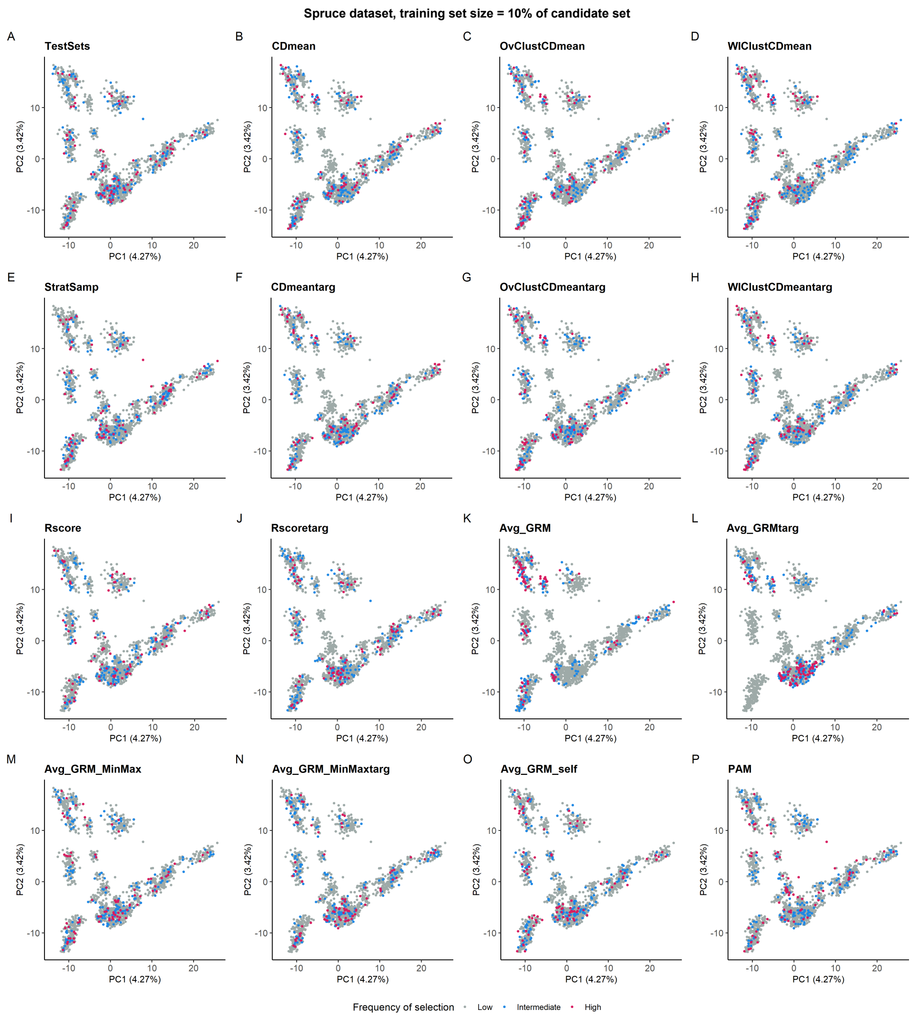


**Fig. S7** Frequency of selection of individuals for the training set across the 40 iterations for all training set optimization methods (plots B-P). If "targ" is added at the end of the name of a method, it corresponds to targeted optimization. Otherwise, untargeted optimization was performed. The frequency of the individuals randomly sampled for the test sets is also shown in plot A. All plots belong to the spruce dataset with a training set size of 10% of the candidate set. The two axes in the plots are the first two principal components that summarize the genetic space and each point is an individual within the dataset. Red colour is used to highlight the 5% most frequently selected individuals, blue colour corresponds to the next 10% most selected individuals and grey is used for the rest.

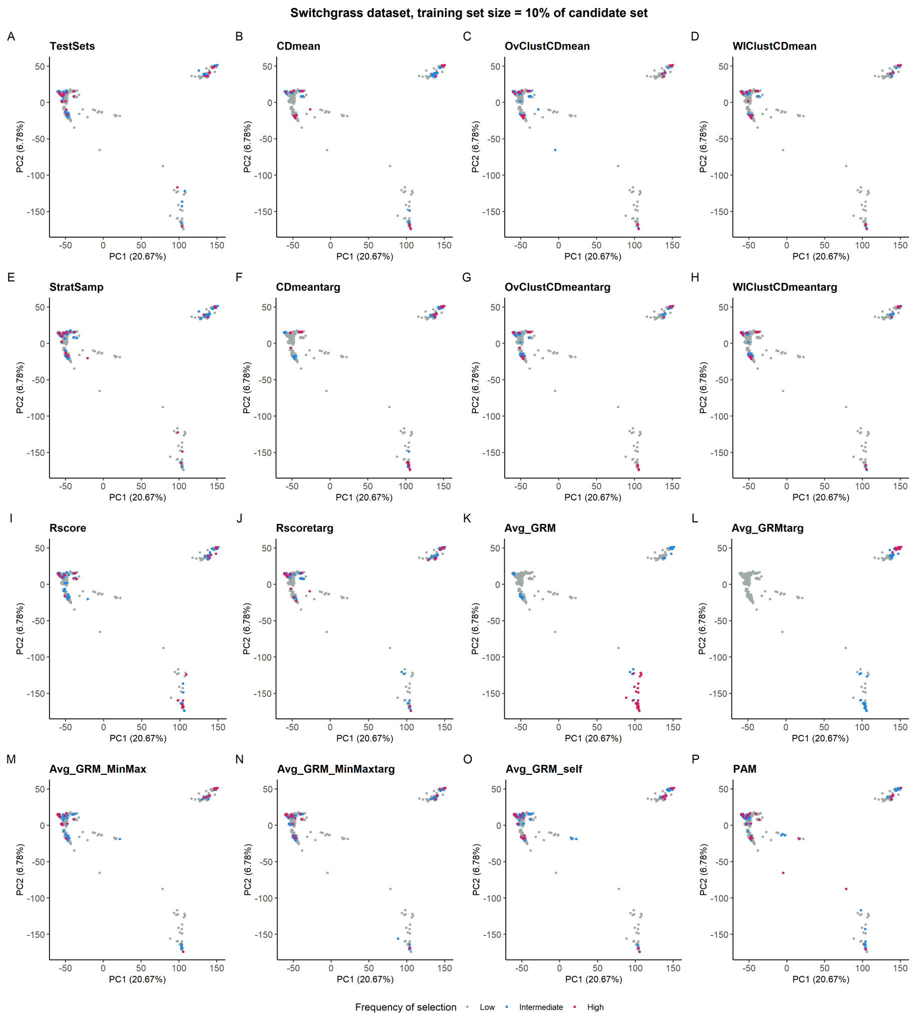


**Fig. S8** Frequency of selection of individuals for the training set across the 40 iterations for all training set optimization methods (plots B-P). If "targ" is added at the end of the name of a method, it corresponds to targeted optimization. Otherwise, untargeted optimization was performed. The frequency of the individuals randomly sampled for the test sets is also shown in plot A. All plots belong to the switchgrass dataset with a training set size of 10% of the candidate set. The two axes in the plots are the first two principal components that summarize the genetic space and each point is an individual within the dataset. Red colour is used to highlight the 5% most frequently selected individuals, blue colour corresponds to the next 10% most selected individuals and grey is used for the rest.

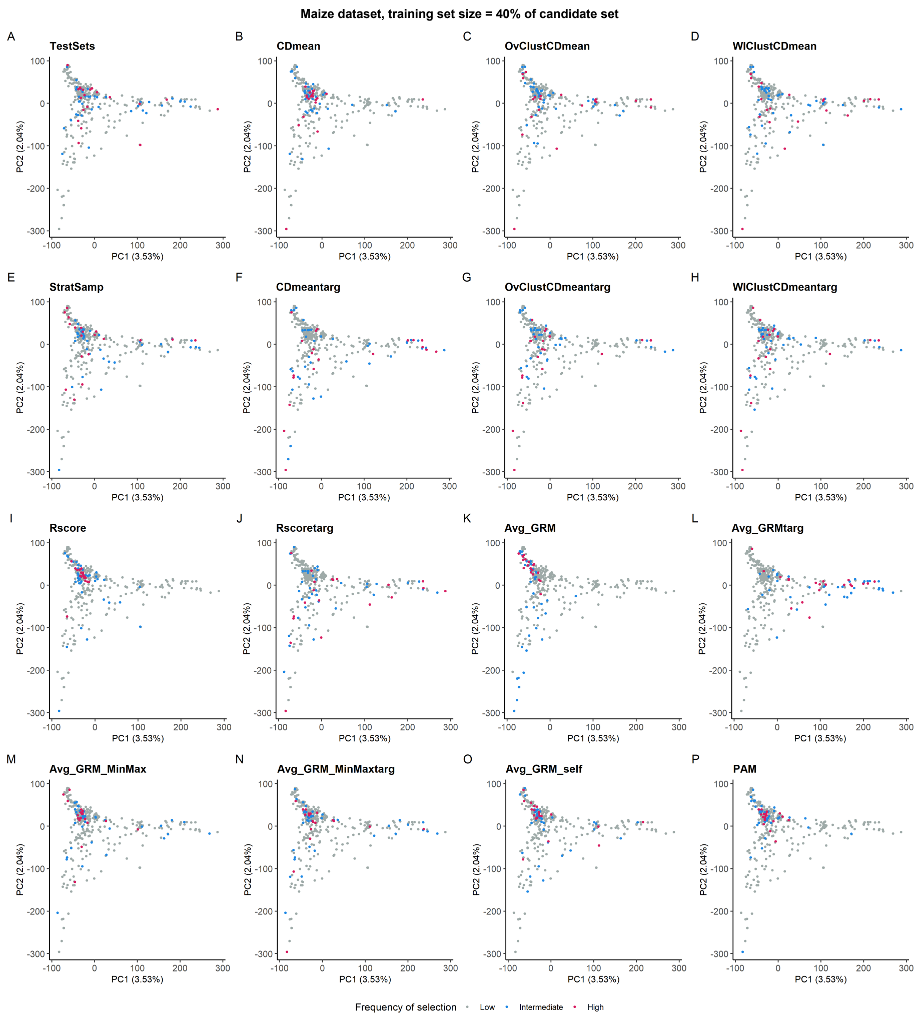


**Fig. S9** Frequency of selection of individuals for the training set across the 40 iterations for all training set optimization methods (plots B-P). If "targ" is added at the end of the name of a method, it corresponds to targeted optimization. Otherwise, untargeted optimization was performed. The frequency of the individuals randomly sampled for the test sets is also shown in plot A. All plots belong to the maize dataset with a training set size of 40% of the candidate set. The two axes in the plots are the first two principal components that summarize the genetic space and each point is an individual within the dataset. Red colour is used to highlight the 5% most frequently selected individuals, blue colour corresponds to the next 10% most selected individuals and grey is used for the rest.

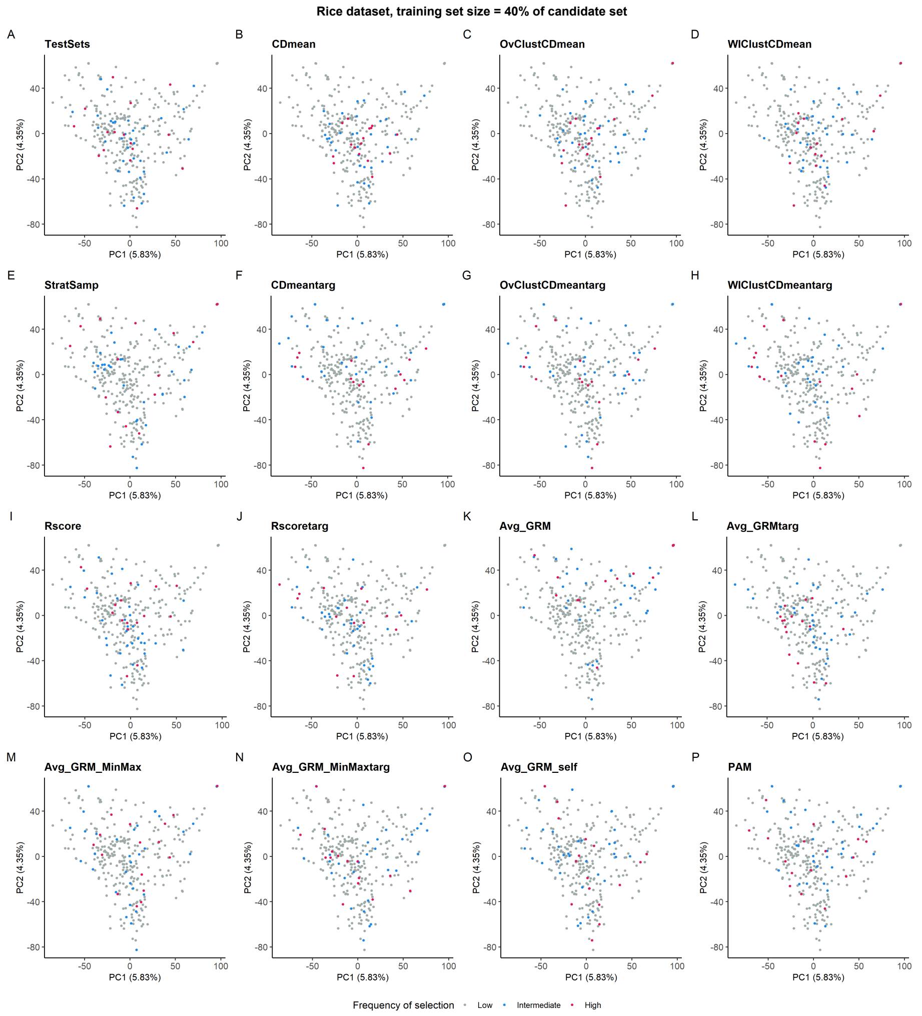


**Fig. S10** Frequency of selection of individuals for the training set across the 40 iterations for all training set optimization methods (plots B-P). If "targ" is added at the end of the name of a method, it corresponds to targeted optimization. Otherwise, untargeted optimization was performed. The frequency of the individuals randomly sampled for the test sets is also shown in plot A. All plots belong to the rice dataset with a training set size of 40% of the candidate set. The two axes in the plots are the first two principal components that summarize the genetic space and each point is an individual within the dataset. Red colour is used to highlight the 5% most frequently selected individuals, blue colour corresponds to the next 10% most selected individuals and grey is used for the rest.

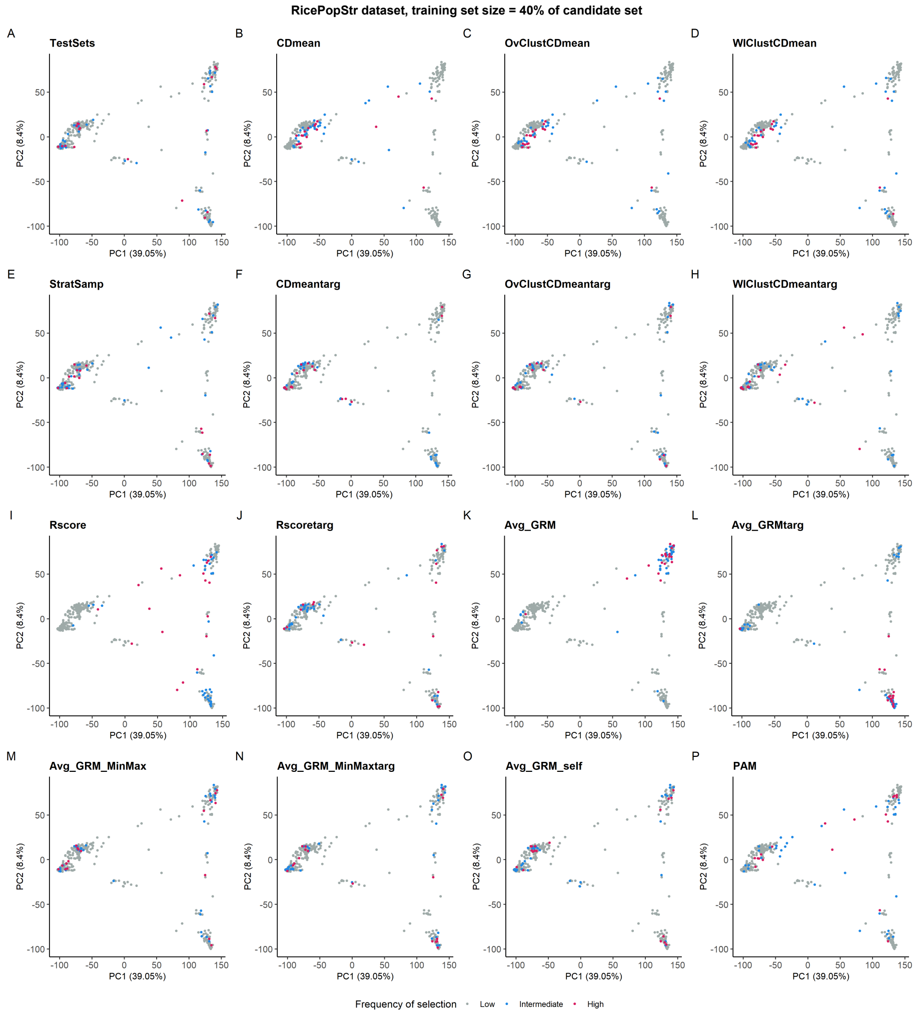


**Fig. S11** Frequency of selection of individuals for the training set across the 40 iterations for all training set optimization methods (plots B-P). If "targ" is added at the end of the name of a method, it corresponds to targeted optimization. Otherwise, untargeted optimization was performed. The frequency of the individuals randomly sampled for the test sets is also shown in plot A. All plots belong to the ricePopStr dataset with a training set size of 40% of the candidate set. The two axes in the plots are the first two principal components that summarize the genetic space and each point is an individual within the dataset. Red colour is used to highlight the 5% most frequently selected individuals, blue colour corresponds to the next 10% most selected individuals and grey is used for the rest.

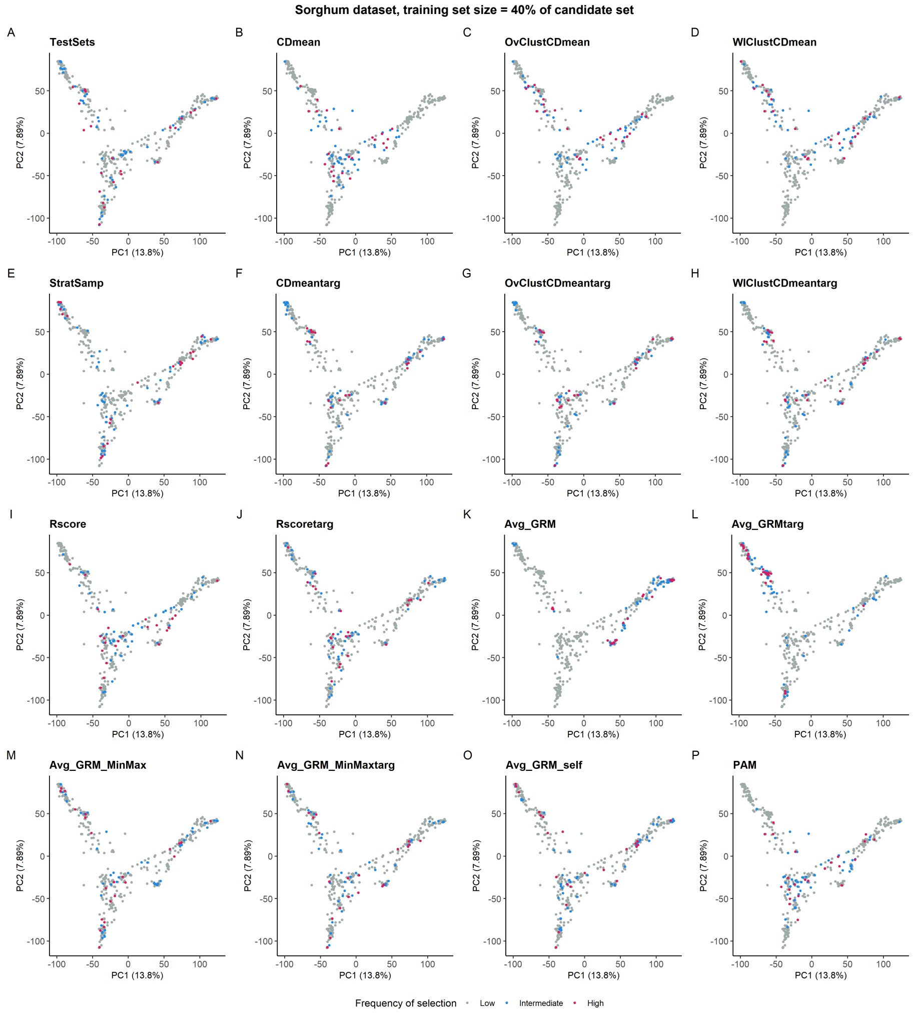


**Fig. S12** Frequency of selection of individuals for the training set across the 40 iterations for all training set optimization methods (plots B-P). If "targ" is added at the end of the name of a method, it corresponds to targeted optimization. Otherwise, untargeted optimization was performed. The frequency of the individuals randomly sampled for the test sets is also shown in plot A. All plots belong to the sorghum dataset with a training set size of 40% of the candidate set. The two axes in the plots are the first two principal components that summarize the genetic space and each point is an individual within the dataset. Red colour is used to highlight the 5% most frequently selected individuals, blue colour corresponds to the next 10% most selected individuals and grey is used for the rest.

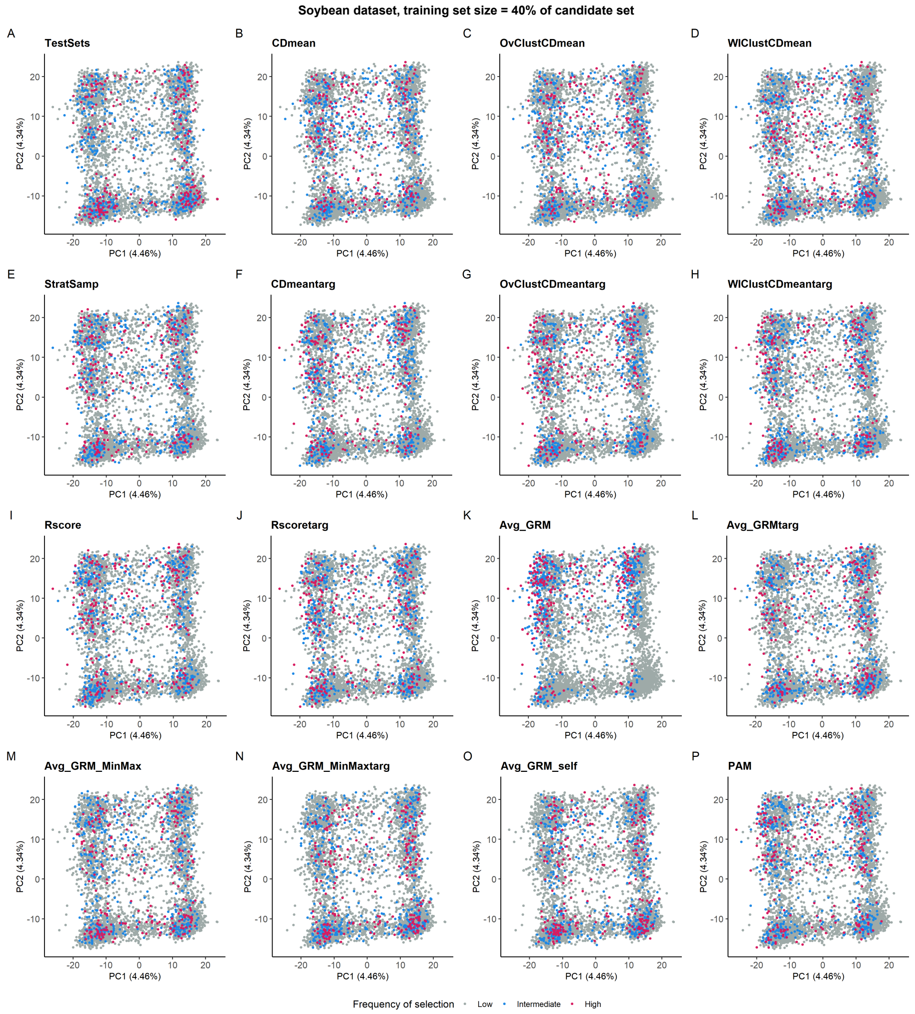


**Fig. S13** Frequency of selection of individuals for the training set across the 40 iterations for all training set optimization methods (plots B-P). If "targ" is added at the end of the name of a method, it corresponds to targeted optimization. Otherwise, untargeted optimization was performed. The frequency of the individuals randomly sampled for the test sets is also shown in plot A. All plots belong to the soybean dataset with a training set size of 40% of the candidate set. The two axes in the plots are the first two principal components that summarize the genetic space and each point is an individual within the dataset. Red colour is used to highlight the 5% most frequently selected individuals, blue colour corresponds to the next 10% most selected individuals and grey is used for the rest.

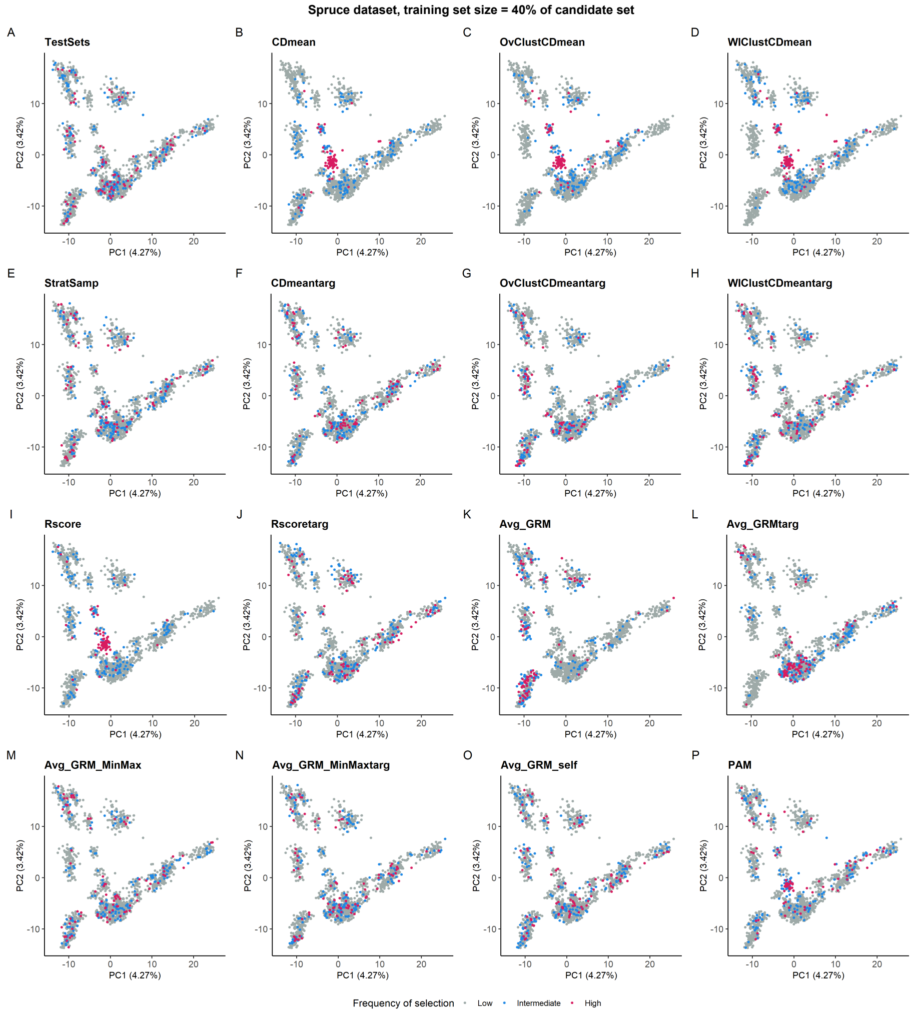


**Fig. S14** Frequency of selection of individuals for the training set across the 40 iterations for all training set optimization methods (plots B-P). If "targ" is added at the end of the name of a method, it corresponds to targeted optimization. Otherwise, untargeted optimization was performed. The frequency of the individuals randomly sampled for the test sets is also shown in plot A. All plots belong to the spruce dataset with a training set size of 40% of the candidate set. The two axes in the plots are the first two principal components that summarize the genetic space and each point is an individual within the dataset. Red colour is used to highlight the 5% most frequently selected individuals, blue colour corresponds to the next 10% most selected individuals and grey is used for the rest.

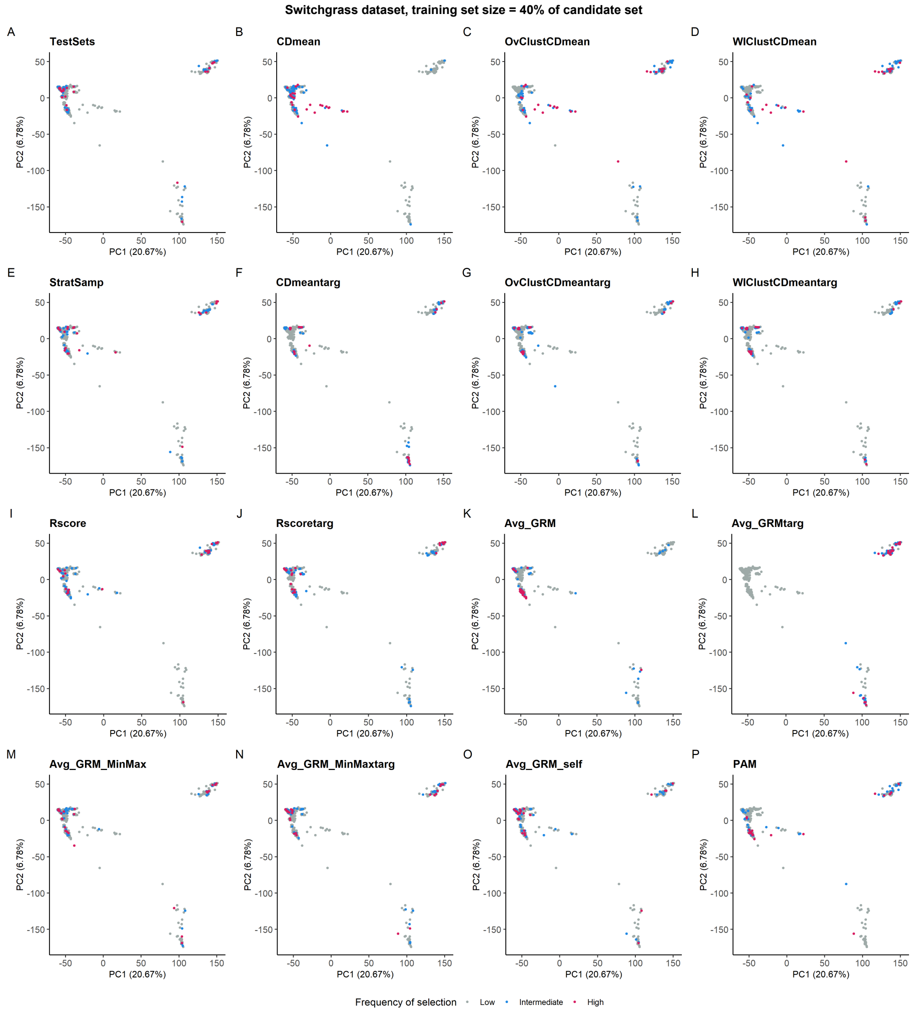


**Fig. S15** Frequency of selection of individuals for the training set across the 40 iterations for all training set optimization methods (plots B-P). If "targ" is added at the end of the name of a method, it corresponds to targeted optimization. Otherwise, untargeted optimization was performed. The frequency of the individuals randomly sampled for the test sets is also shown in plot A. All plots belong to the switchgrass dataset with a training set size of 40% of the candidate set. The two axes in the plots are the first two principal components that summarize the genetic space and each point is an individual within the dataset. Red colour is used to highlight the 5% most frequently selected individuals, blue colour corresponds to the next 10% most selected individuals and grey is used for the rest.

## Note 2. Representative iteration

With the aim to validate the results obtained using the frequency of selection (Figures S2 - S15), we employed a representative iteration of the cross validation and plotted which individuals were selected by each optimization method for said iteration (Figures S16 - S22). The representative iteration was the one whose accuracies across traits, models and optimization methods had the highest correlation with the average accuracies for the 40 iterations. This is needed because the absence of frequently selected individuals in a zone of the genetic space can be due to two different causes. It is possible that in said zone the method does not select any individuals (Cause 1) or it may perform a very inconsistent selection (Cause 2), leading many individuals to be selected with a frequency lower than the cutoff needed to be highlighted in Figures S2 - S15. A mixture of Causes 1 and 2 is also possible. Plotting a representative iteration (Figures S16 - S22) allows us to discriminate if Cause 1 or Cause 2 is happening. If the representative iteration shows that no individual was selected in the zone with low frequency of selection, Cause 1 is taking place. Otherwise, Cause 2 is happening. The sampling performed by all methods for the representative iteration when the training set size was 10% of thecandidate set was extremely similar to the results obtained by the frequency of selection (data not shown). However, when the training set size was 40% of thecandidate set some differences could be found (Figures S16 - S22). In Avg_GRM, the lack of frequently selected individuals in most of the genetic space was due to Cause 1 mainly. Avg_GRM only sampled individuals from a small zone of the genetic space. In Rscore the low frequency of selection in the extremes of the plots was due to Cause 2. In CDmean there is a mixture of both causes. There was generally an inconsistent selection in the edges of the genetic space (Cause 2) and the most extreme individuals were largely not selected, selecting more individuals from the center of the plot than from the edges (Cause 1). This may explain the poor performance of untargeted CDmean when population structure was strong, as in this situation focusing on selecting individuals from the center of the plot may cause an overrepresentation or underrepresentation of some clusters. It is important to note that in targeted CDmean this trend is no longer observed and the genetic space is evenly sampled, which is consistent with its good performance regardless of population structure (Table 3).

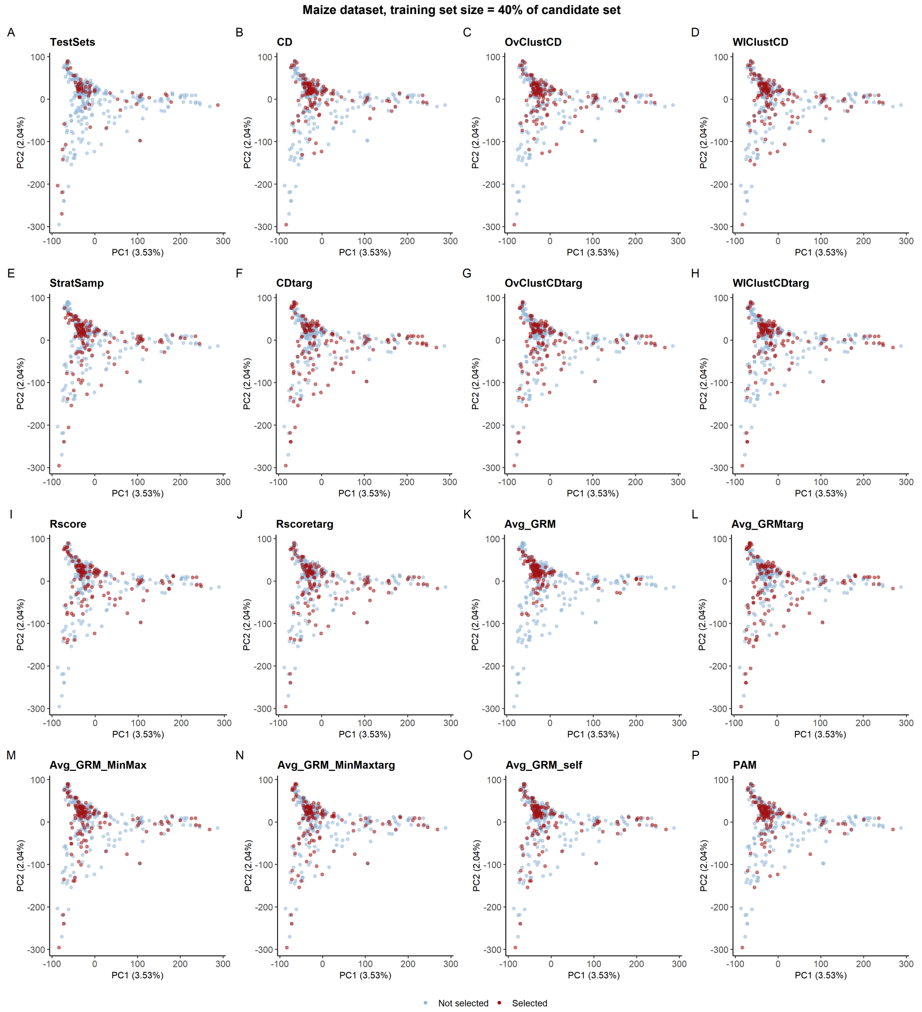


**Fig. S16** Selected individuals for the training set in a single iteration by all training set optimization methods (plots B-P). If "targ" is added at the end of the name of a method, it corresponds to targeted optimization. Otherwise, untargeted optimization was performed. The individuals randomly selected as the test set are also shown in plot A. All plots belong to the maize dataset with a training set size of 40% of the candidate set. The two axes in the plots are the first two principal components that summarize the genetic space and each point is an individual within the dataset. Blue colour indicates non-selected individuals and red colour indicates selected individuals. The iteration to be plotted was chosen as the one whose accuracies across traits, models and optimization methods achieved the highest correlation with the corresponding average values for the 40 iterations.

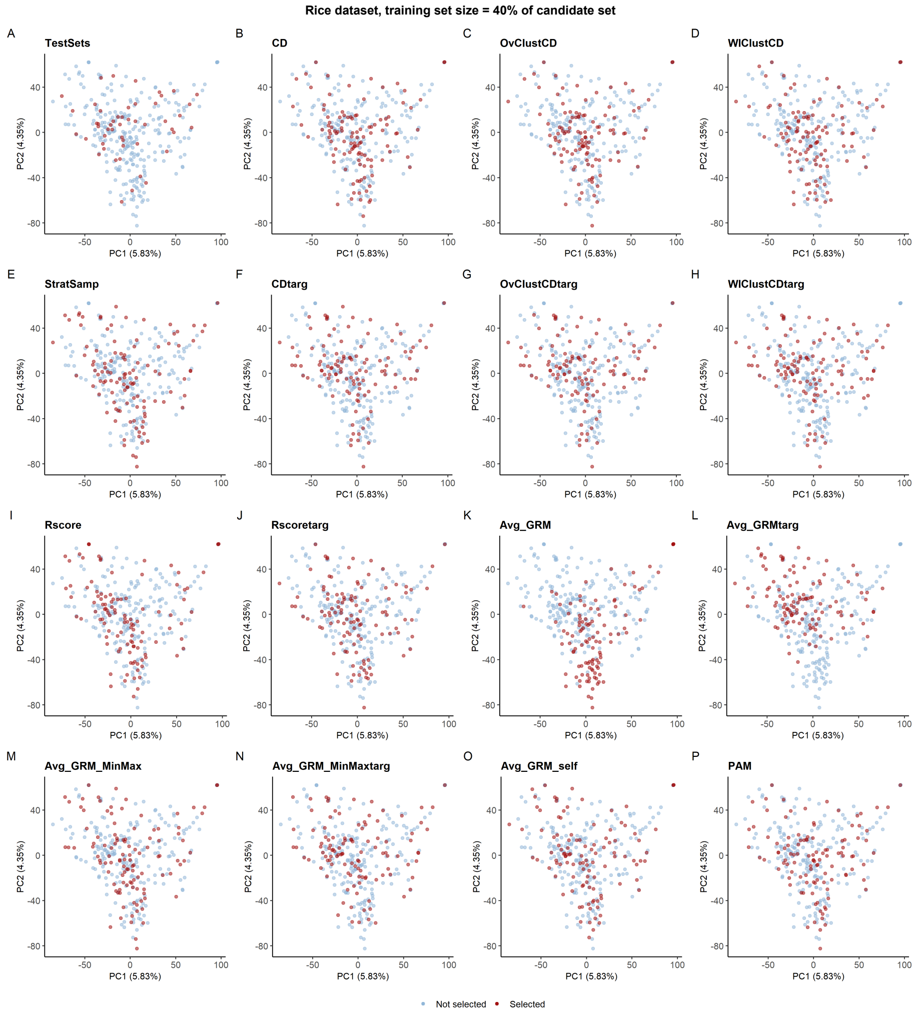


**Fig. S17** Selected individuals for the training set in a single iteration by all training set optimization methods (plots B-P). If "targ" is added at the end of the name of a method, it corresponds to targeted optimization. Otherwise, untargeted optimization was performed. The individuals randomly selected as the test set are also shown in plot A. All plots belong to the rice dataset with a training set size of 40% of the candidate set. The two axes in the plots are the first two principal components that summarize the genetic space and each point is an individual within the dataset. Blue colour indicates non-selected individuals and red colour indicates selected individuals. The iteration to be plotted was chosen as the one whose accuracies across traits, models and optimization methods achieved the highest correlation with the corresponding average values for the 40 iterations.

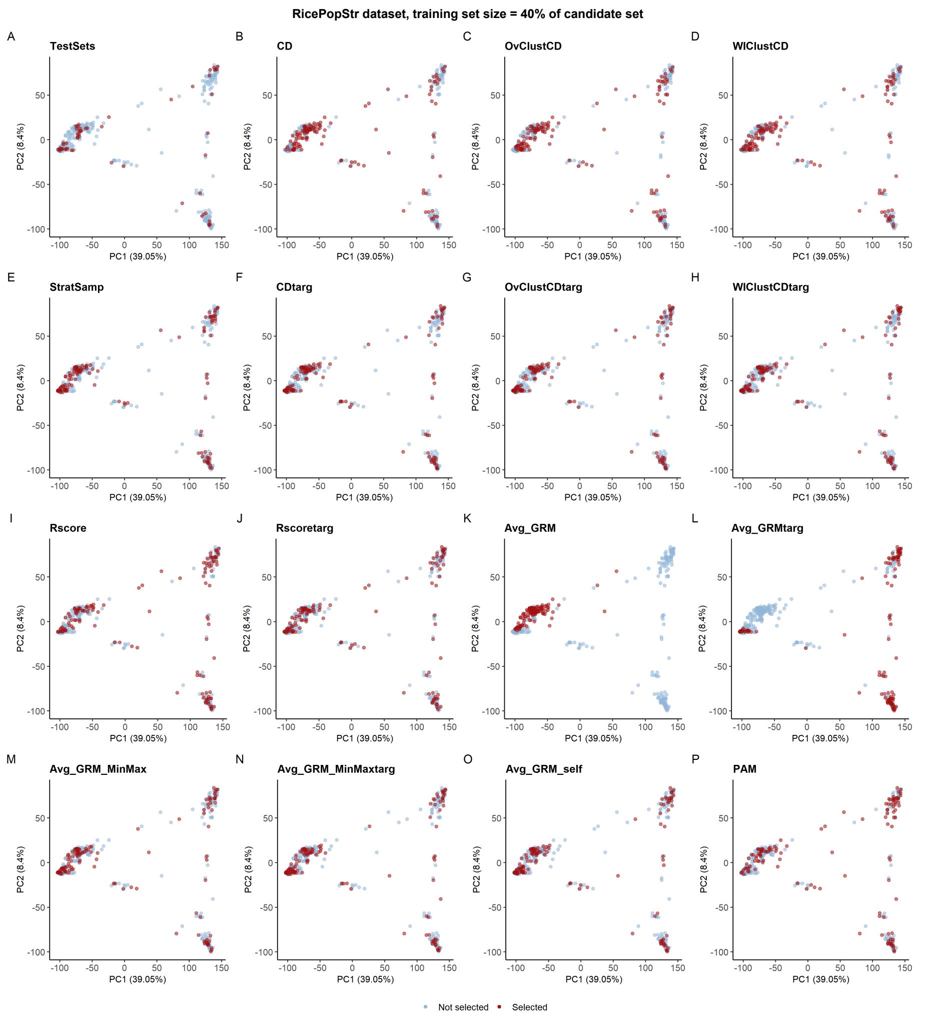


**Fig. S18** Selected individuals for the training set in a single iteration by all training set optimization methods (plots B-P). If "targ" is added at the end of the name of a method, it corresponds to targeted optimization. Otherwise, untargeted optimization was performed. The individuals randomly selected as the test set are also shown in plot A. All plots belong to the ricePopStr dataset with a training set size of 40% of the candidate set. The two axes in the plots are the first two principal components that summarize the genetic space and each point is an individual within the dataset. Blue colour indicates non-selected individuals and red colour indicates selected individuals. The iteration to be plotted was chosen as the one whose accuracies across traits, models and optimization methods achieved the highest correlation with the corresponding average values for the 40 iterations.

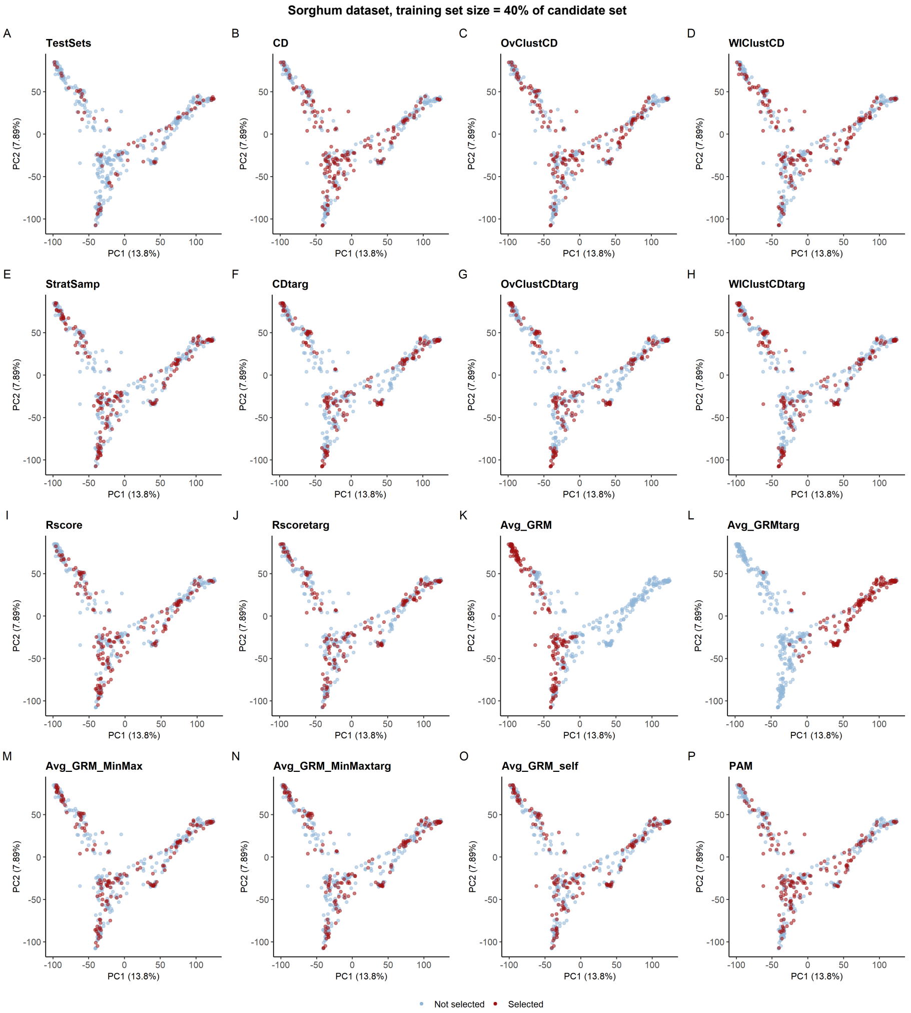


**Fig. S19** Selected individuals for the training set in a single iteration by all training set optimization methods (plots B-P). If "targ" is added at the end of the name of a method, it corresponds to targeted optimization. Otherwise, untargeted optimization was performed. The individuals randomly selected as the test set are also shown in plot A. All plots belong to the sorghum dataset with a training set size of 40% of the candidate set. The two axes in the plots are the first two principal components that summarize the genetic space and each point is an individual within the dataset. Blue colour indicates non-selected individuals and red colour indicates selected individuals. The iteration to be plotted was chosen as the one whose accuracies across traits, models and optimization methods achieved the highest correlation with the corresponding average values for the 40 iterations.

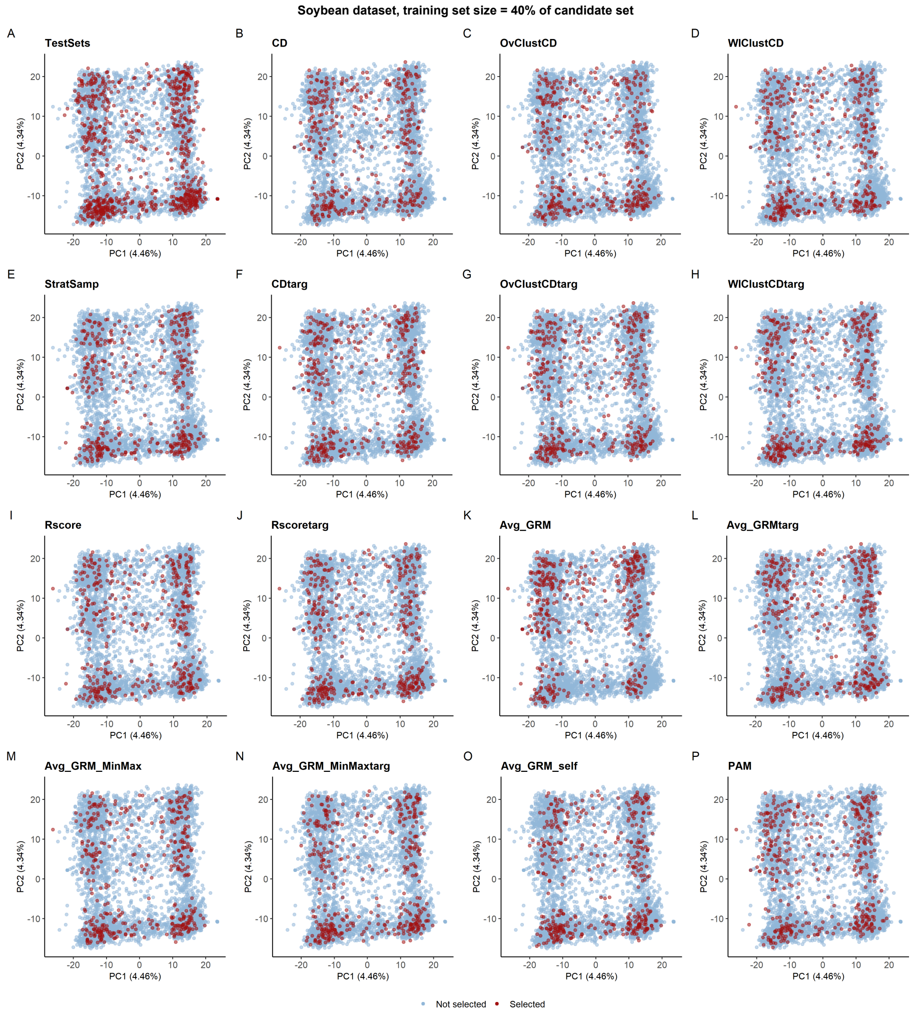


**Fig. S20** Selected individuals for the training set in a single iteration by all training set optimization methods (plots B-P). If "targ" is added at the end of the name of a method, it corresponds to targeted optimization. Otherwise, untargeted optimization was performed. The individuals randomly selected as the test set are also shown in plot A. All plots belong to the soybean dataset with a training set size of 40% of the candidate set. The two axes in the plots are the first two principal components that summarize the genetic space and each point is an individual within the dataset. Blue colour indicates non-selected individuals and red colour indicates selected individuals. The iteration to be plotted was chosen as the one whose accuracies across traits, models and optimization methods achieved the highest correlation with the corresponding average values for the 40 iterations.

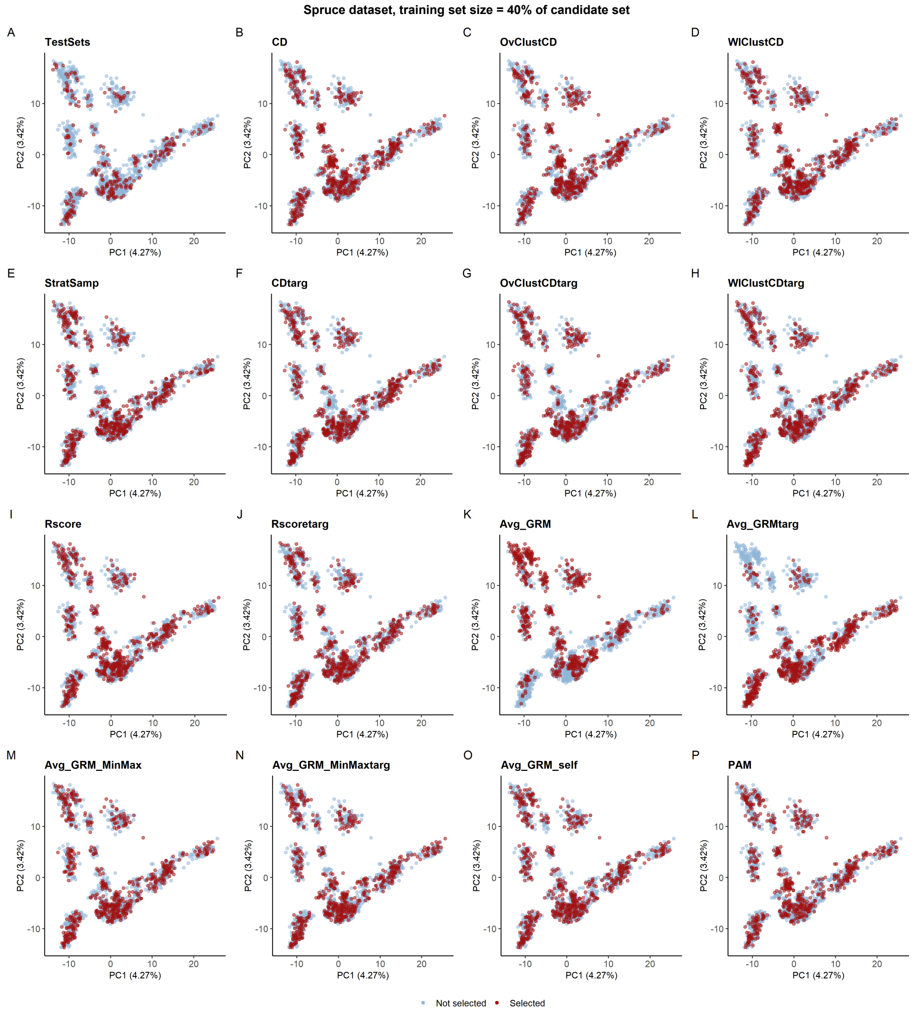


**Fig. S21** Selected individuals for the training set in a single iteration by all training set optimization methods (plots B-P). If "targ" is added at the end of the name of a method, it corresponds to targeted optimization. Otherwise, untargeted optimization was performed. The individuals randomly selected as the test set are also shown in plot A. All plots belong to the spruce dataset with a training set size of 40% of the candidate set. The two axes in the plots are the first two principal components that summarize the genetic space and each point is an individual within the dataset. Blue colour indicates non-selected individuals and red colour indicates selected individuals. The iteration to be plotted was chosen as the one whose accuracies across traits, models and optimization methods achieved the highest correlation with the corresponding average values for the 40 iterations.

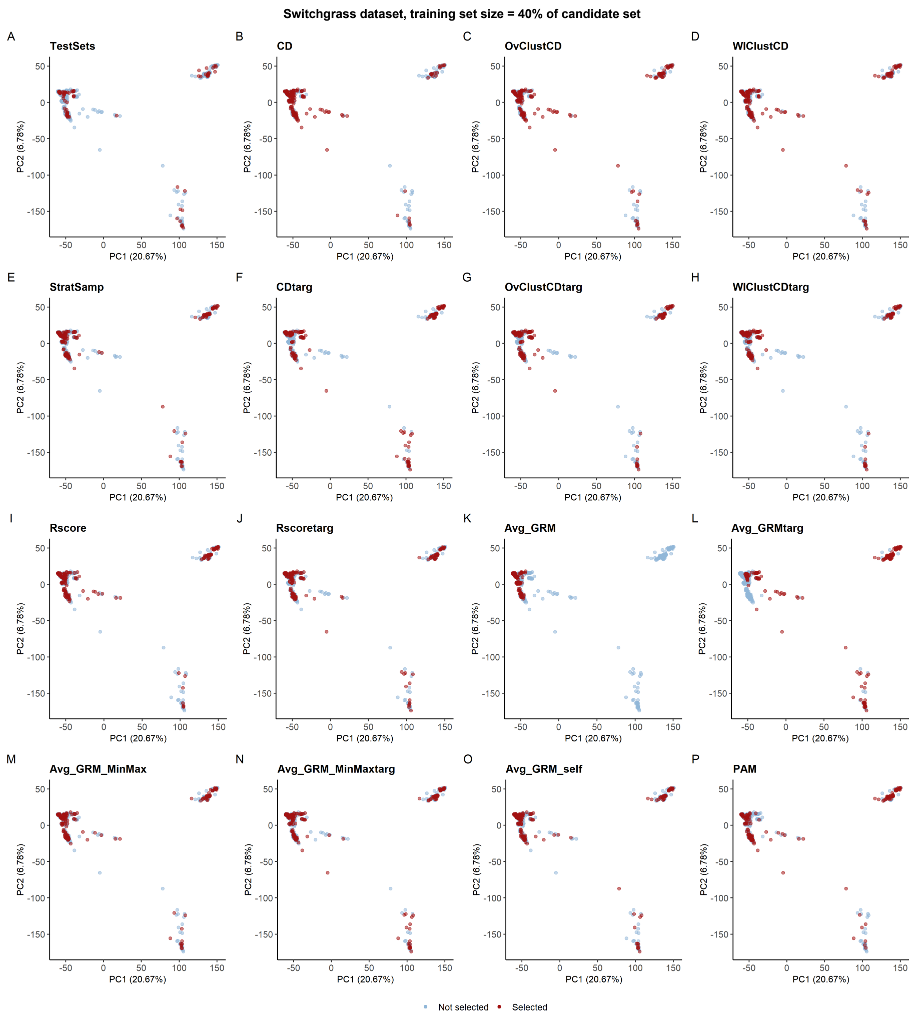


**Fig. S22** Selected individuals for the training set in a single iteration by all training set optimization methods (plots B-P). If "targ" is added at the end of the name of a method, it corresponds to targeted optimization. Otherwise, untargeted optimization was performed. The individuals randomly selected as the test set are also shown in plot A. All plots belong to the switchgrass dataset with a training set size of 40% of the candidate set. The two axes in the plots are the first two principal components that summarize the genetic space and each point is an individual within the dataset. Blue colour indicates non-selected individuals and red colour indicates selected individuals. The iteration to be plotted was chosen as the one whose accuracies across traits, models and optimization methods achieved the highest correlation with the corresponding average values for the 40 iterations.

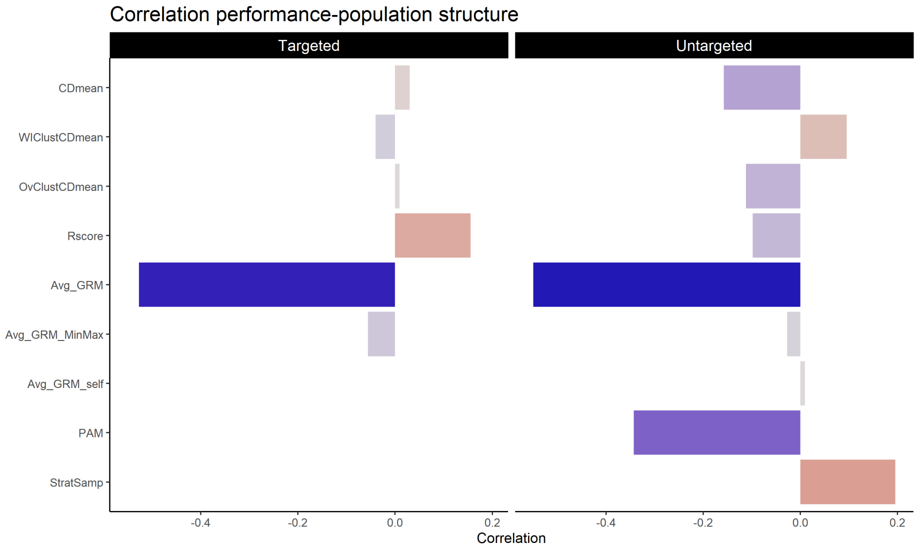


**Fig. S23** Correlation between the average performance of the optimization methods in the non-simulated traits and the population structure of the datasets. The performance of the methods was measured as the gain in area under the curve (AUC) relative to random sampling across training set sizes for each dataset-trait-model combination. population structure was measured as the percentage of variance explained by the first 20 principal components of a principal component analysis made over the marker data.

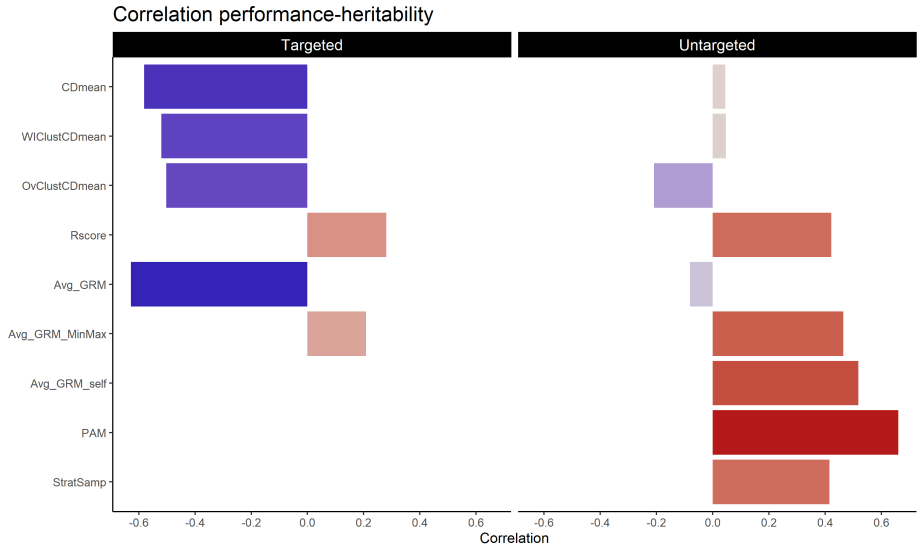


**Fig. S24** Correlation between the average performance of the optimization methods for each trait and its heritability. The performance of the methods was measured as the gain in area under the curve (AUC) relative to random sampling across training set sizes for each dataset-trait-model combination. It is important to note that, before calculating the average, the values within each dataset-trait-model combination were normalized.

**Table S53** Time needed in seconds to run different training set optimization methods for a dataset whose size is indicated in the header. ($X_{n\times p}$) indicates that the marker matrix ($X$) for the dataset contains $n$ individuals and $p$ markers per line. The TrainSel parameters used are 100 iterations, 100 training sets in the population of the genetic algorithm, 5 simulated annealing steps per iteration and 5 elite lines used as parents of the next generation. All methods were tested for untargeted optimization with candidate set = 85% of the total, training set size = 50% of the candidate set and target population = entire candidate set for all methods except CDmean, where the target population is the remaining set (individuals in the candidate set not selected for the training set). The values shown in this table are the average over 20 repetitions. The time complexity shown is an approximation that assumes that the training set, target population and candidate set are a constant fraction of $n$ and that the time complexity of the product and the inversion for a $n \times n$ matrix is $O(n^3)$. It is important to note that the time complexity specified in the last column and the empiric values obtained for the computational time don't match for CDmean, Rscore and the Avg_GRM variants because the time complexity refers only to the evaluation criterion, but it does not take into account the genetic algorithm and simulated annealing performed by TrainSel. However, for large values of $n$ and $p$ the time taken by the evaluation criterion dominates and the time complexity shown is a good approximation of the real one. For PAM, the time complexity doesn't take into account the time needed to calculate the dissimilarity matrix and that explains the disparity between it and the empiric values. Stratified sampling was not included in this table because it is close to instant for any dataset size. All other methods tested in this work are variations of the ones shown in this table and their computational time would be very similar to them.

**Computational time**

| Algorithms | Time in seconds | | | | | Time complexity |
|---|---|---|---|---|---|---|
| | $X_{75\times100}$ | $X_{100\times75}$ | $X_{100\times100}$ | $X_{100\times200}$ | $X_{200\times100}$ | |
| CD | 3.963 | 5.764 | 5.782 | 5.752 | 15.73 | $O(n^3)$ |
| Rscore | 7.782 | 9.347 | 10.858 | 19.071 | 27.495 | $O(pn^2)$ |
| Avg_GRM_MinMax | 0.786 | 0.984 | 0.978 | 0.982 | 2.215 | $O(n^2)$ |
| Avg_GRM_self | 0.476 | 0.594 | 0.61 | 0.598 | 1.237 | $O(n^2)$ |
| PAM | 0.00353 | 0.00558 | 0.00627 | 0.0106 | 0.038 | $O(n^3)$ |

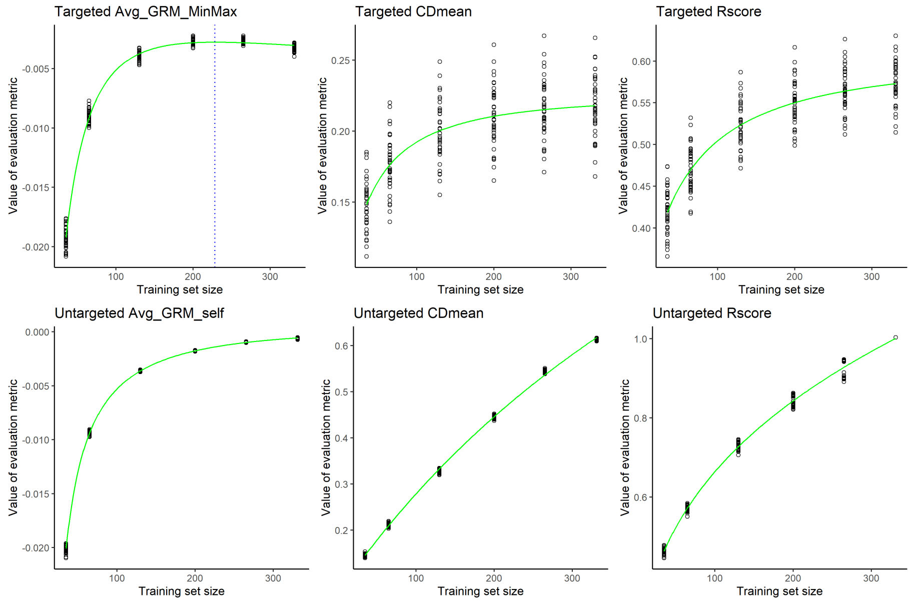


**Fig. S25** Evolution of the tested evaluation criteria as the training set size increases in the maize dataset. For each training set size tested there are 40 values that correspond to the 40 iterations performed in the cross validation. The green line is the function fitted to the data ($evaluation\_metric = \frac{ln(TRSsize-d)}{m(TRSsize-d)^p} + n$). The vertical dotted line in targeted Avg_GRM_MinMax shows the point at which the function reaches its maximum.

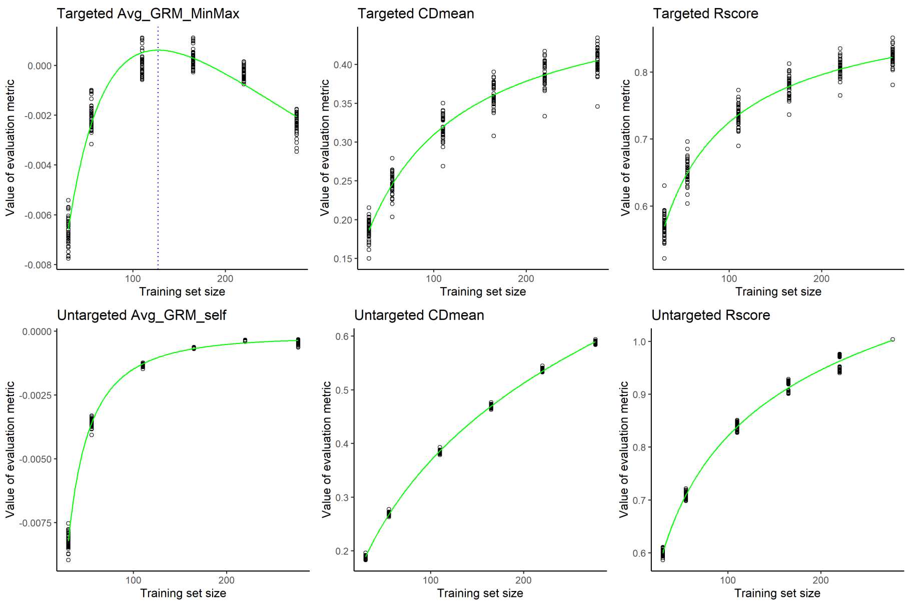


**Fig. S26** Evolution of the tested evaluation criteria as the training set size increases in the rice dataset. For each training set size tested there are 40 values that correspond to the 40 iterations performed in the cross validation. The green line is the function fitted to the data ($evaluation\_metric = \frac{ln(TRSsize-d)}{m(TRSsize-d)^p} + n$). The vertical dotted line in targeted Avg_GRM_MinMax shows the point at which the function reaches its maximum.

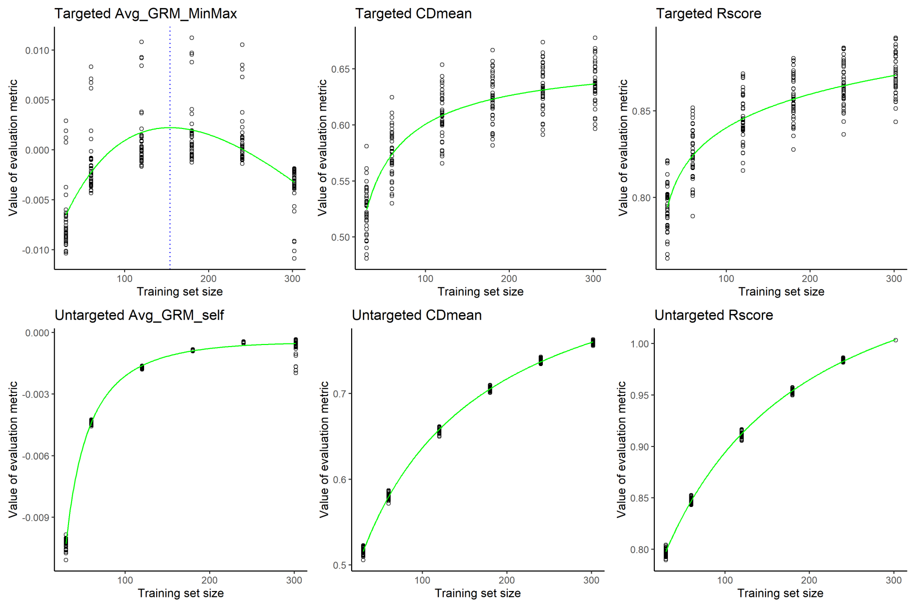


**Fig. S27** Evolution of the tested evaluation criteria as the training set size increases in the ricePopStr dataset. For each training set size tested there are 40 values that correspond to the 40 iterations performed in the cross validation. The green line is the function fitted to the data ($evaluation\_metric = \frac{ln(TRSsize-d)}{m(TRSsize-d)^p} + n$). The vertical dotted line in targeted Avg_GRM_MinMax shows the point at which the function reaches its maximum.

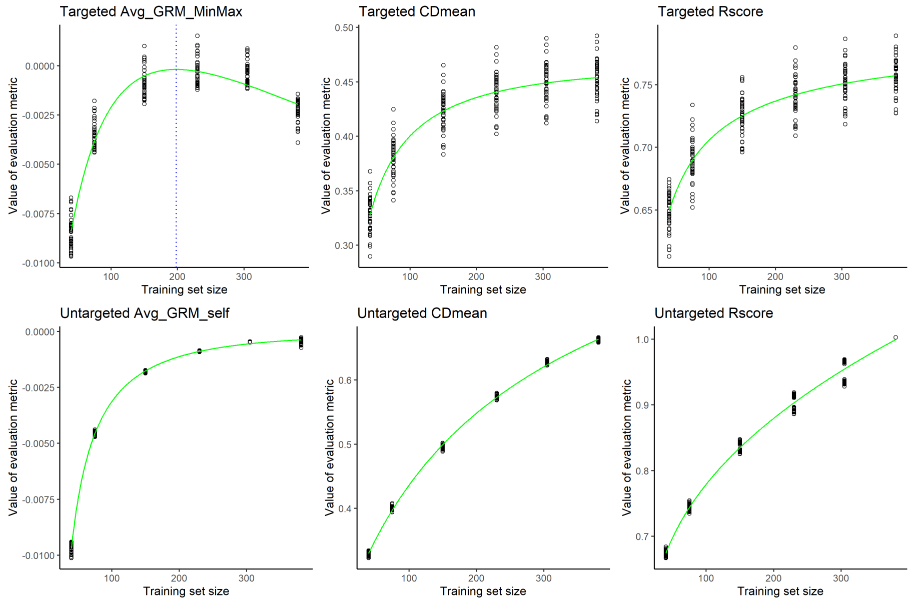


**Fig. S28** Evolution of the tested evaluation criteria as the training set size increases in the sorghum dataset. For each training set size tested there are 40 values that correspond to the 40 iterations performed in the cross validation. The green line is the function fitted to the data ($evaluation\_metric = \frac{ln(TRSsize-d)}{m(TRSsize-d)^p} + n$). The vertical dotted line in targeted Avg_GRM_MinMax shows the point at which the function reaches its maximum.

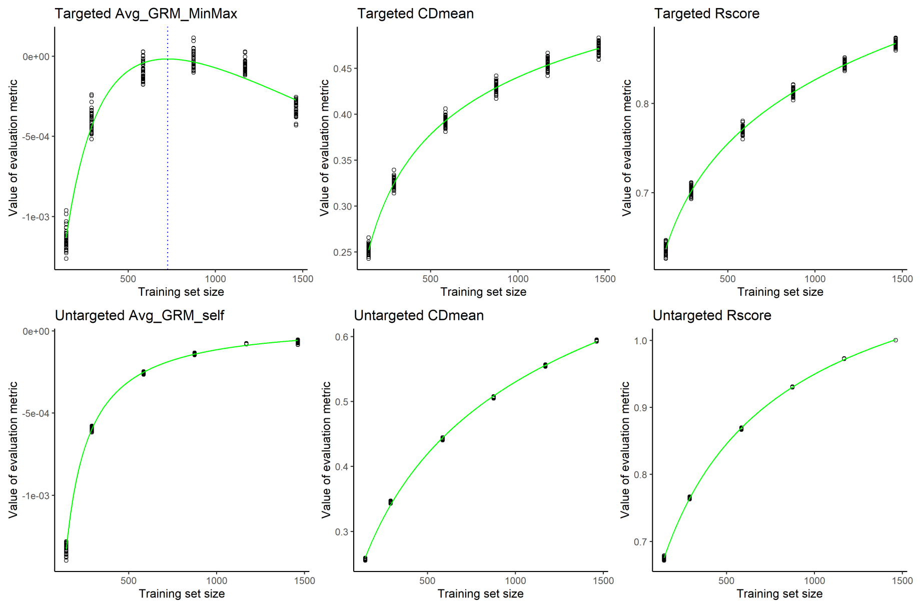


**Fig. S29** Evolution of the tested evaluation criteria as the training set size increases in the spruce dataset. For each training set size tested there are 40 values that correspond to the 40 iterations performed in the cross validation. The green line is the function fitted to the data ($evaluation\_metric = \frac{ln(TRSsize-d)}{m(TRSsize-d)^p} + n$). The vertical dotted line in targeted Avg_GRM_MinMax shows the point at which the function reaches its maximum.

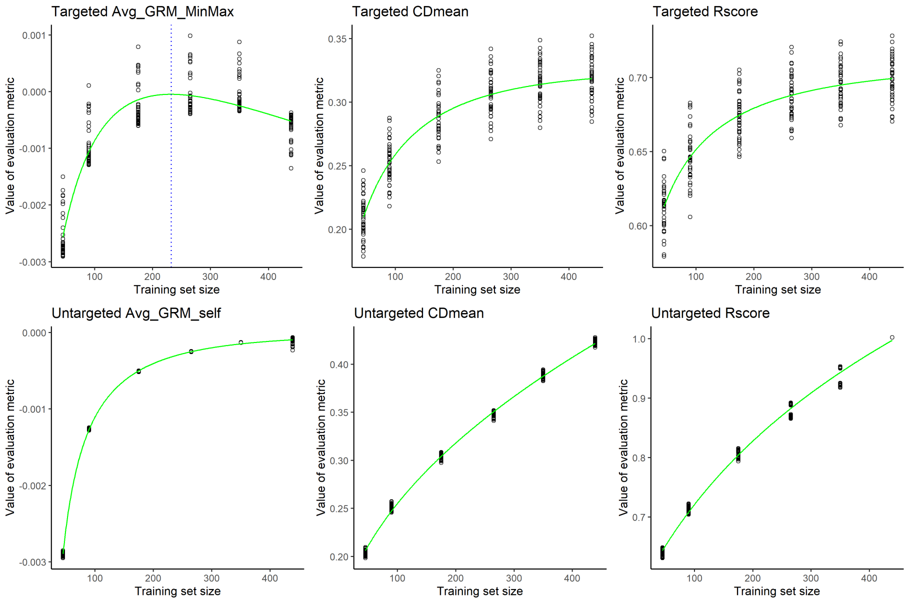


**Fig. S30** Evolution of the tested evaluation criteria as the training set size increases in the switchgrass dataset. For each training set size tested there are 40 values that correspond to the 40 iterations performed in the cross validation. The green line is the function fitted to the data ($evaluation\_metric = \frac{ln(TRSsize-d)}{m(TRSsize-d)^p} + n$). The vertical dotted line in targeted Avg_GRM_MinMax shows the point at which the function reaches its maximum.

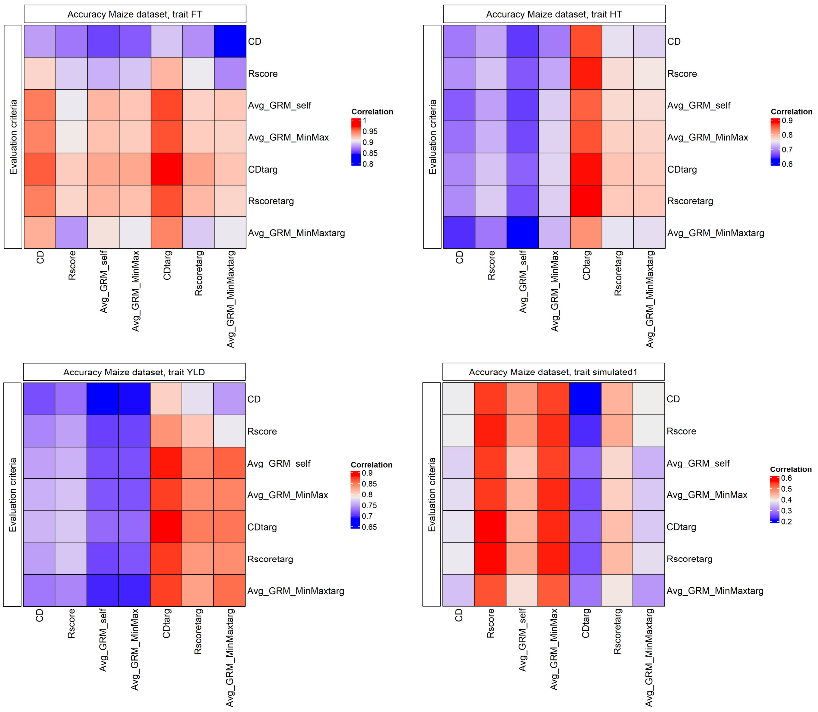


**Fig. S31** Average correlation between the accuracy and the evaluation metrics across the tested training set sizes (10, 20, 40, 60 and 80% of the candidate set) for all traits in maize dataset. The horizontal axis contains the optimization methods used to obtain the different training sets. For each training set the evaluation metrics in the vertical axis were computed and were correlated with the accuracy obtained using GBLUP model.

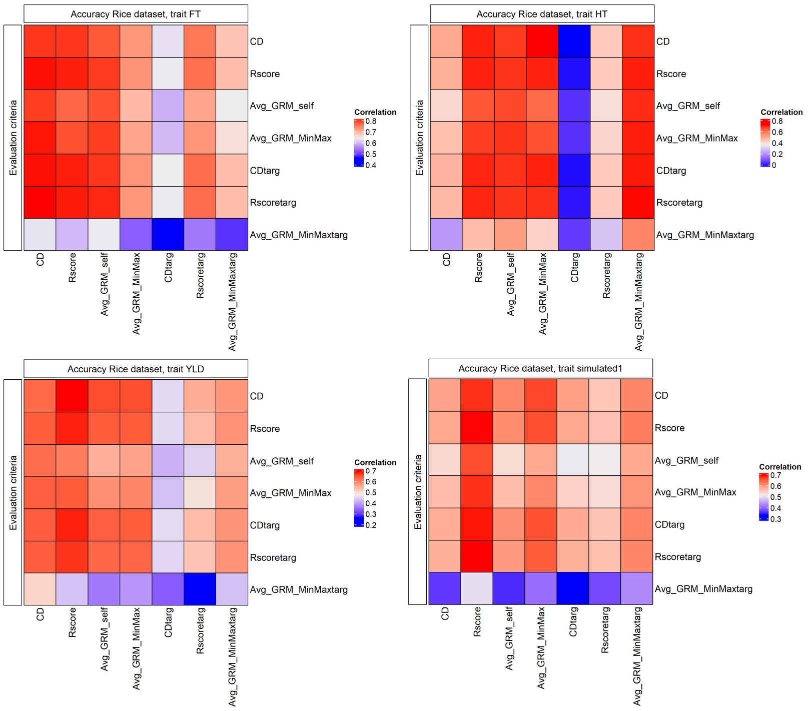


**Fig. S32** Average correlation between the accuracy and the evaluation metrics across the tested training set sizes (10, 20, 40, 60 and 80% of the candidate set) for all traits in rice dataset. The horizontal axis contains the optimization methods used to obtain the different training sets. For each training set the evaluation metrics in the vertical axis were computed and were correlated with the accuracy obtained using GBLUP model.

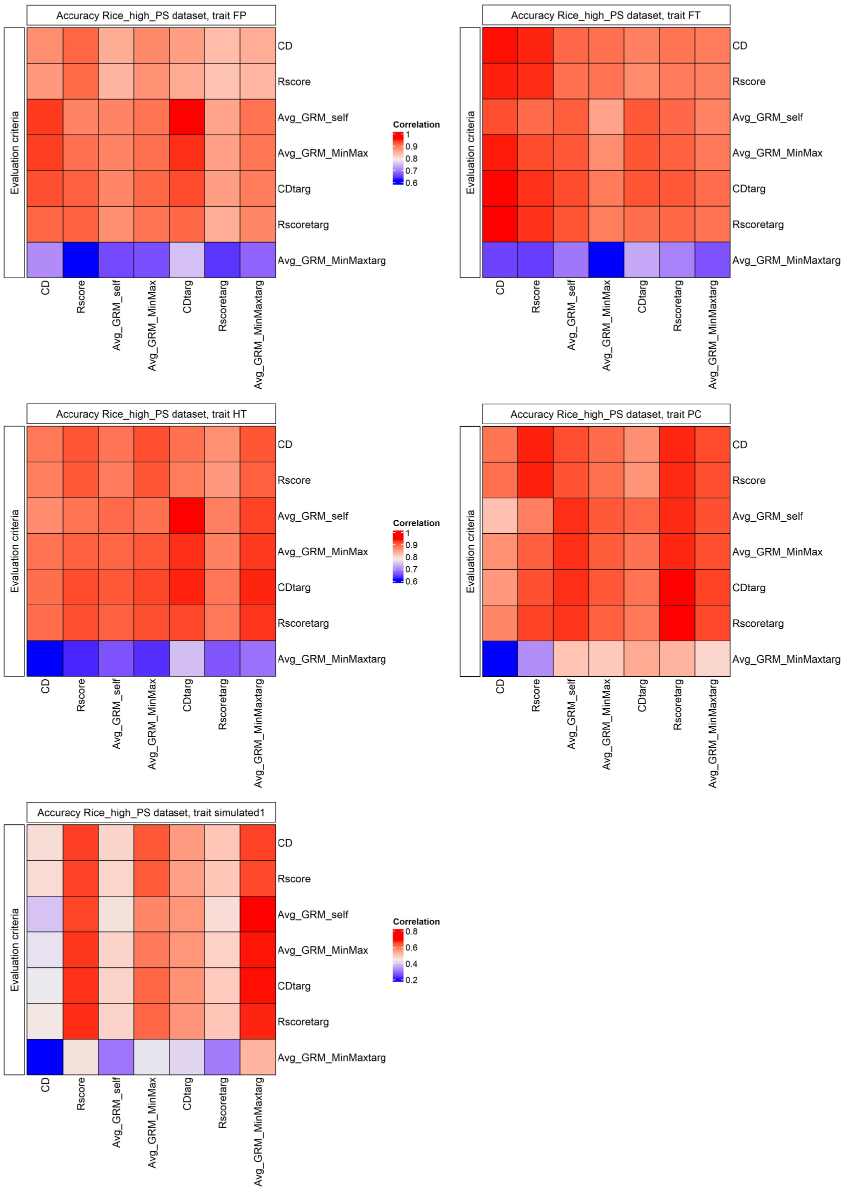


**Fig. S33** Average correlation between the accuracy and the evaluation metrics across the tested training set sizes (10, 20, 40, 60 and 80% of the candidate set) for all traits in ricePopStr dataset. The horizontal axis contains the optimization methods used to obtain the different training sets. For each training set the evaluation metrics in the vertical axis were computed and were correlated with the accuracy obtained using GBLUP model.

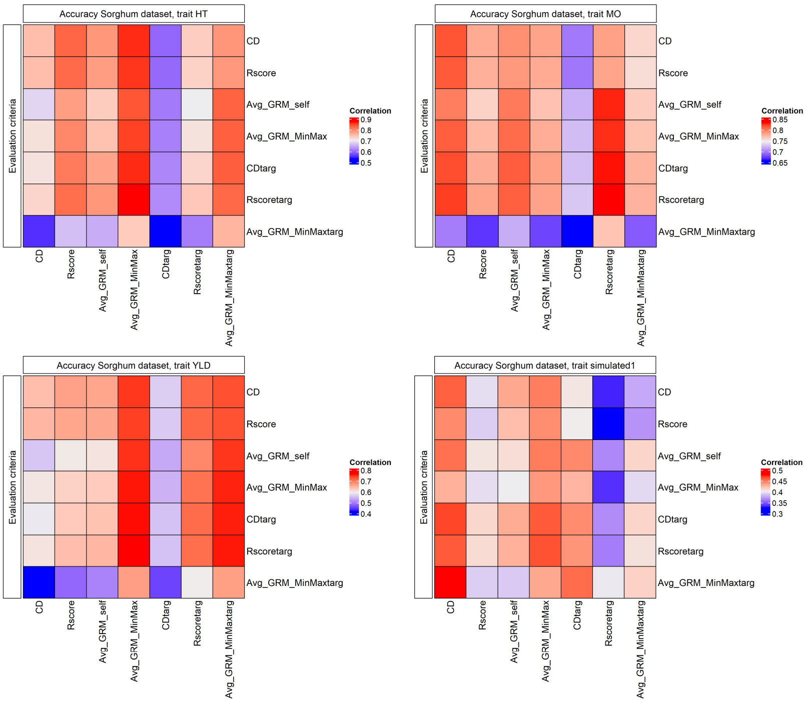


**Fig. S34** Average correlation between the accuracy and the evaluation metrics across the tested training set sizes (10, 20, 40, 60 and 80% of the candidate set) for all traits in sorghum dataset. The horizontal axis contains the optimization methods used to obtain the different training sets. For each training set the evaluation metrics in the vertical axis were computed and were correlated with the accuracy obtained using GBLUP model.

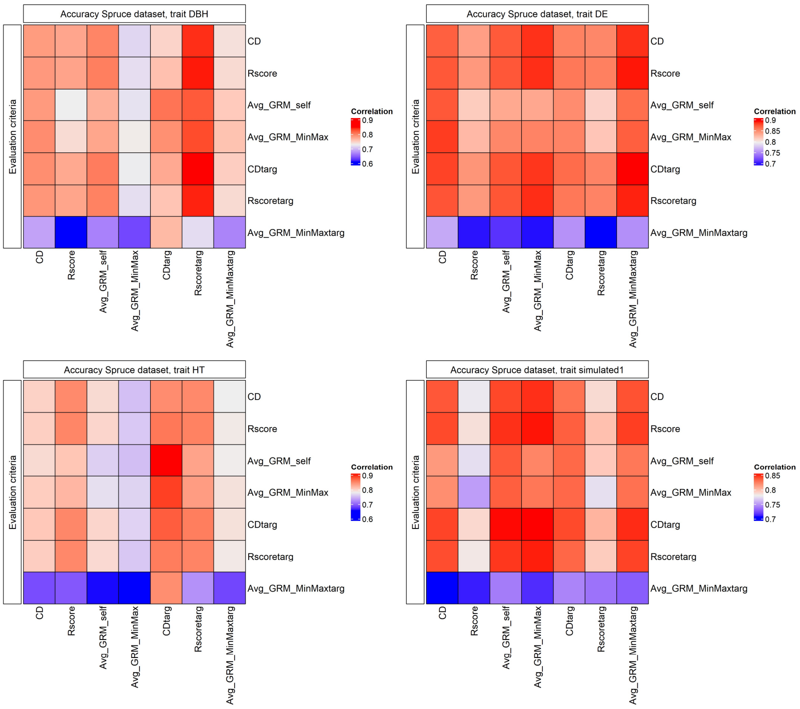


**Fig. S35** Average correlation between the accuracy and the evaluation metrics across the tested training set sizes (10, 20, 40, 60 and 80% of the candidate set) for all traits in spruce dataset. The horizontal axis contains the optimization methods used to obtain the different training sets. For each training set the evaluation metrics in the vertical axis were computed and were correlated with the accuracy obtained using GBLUP model.

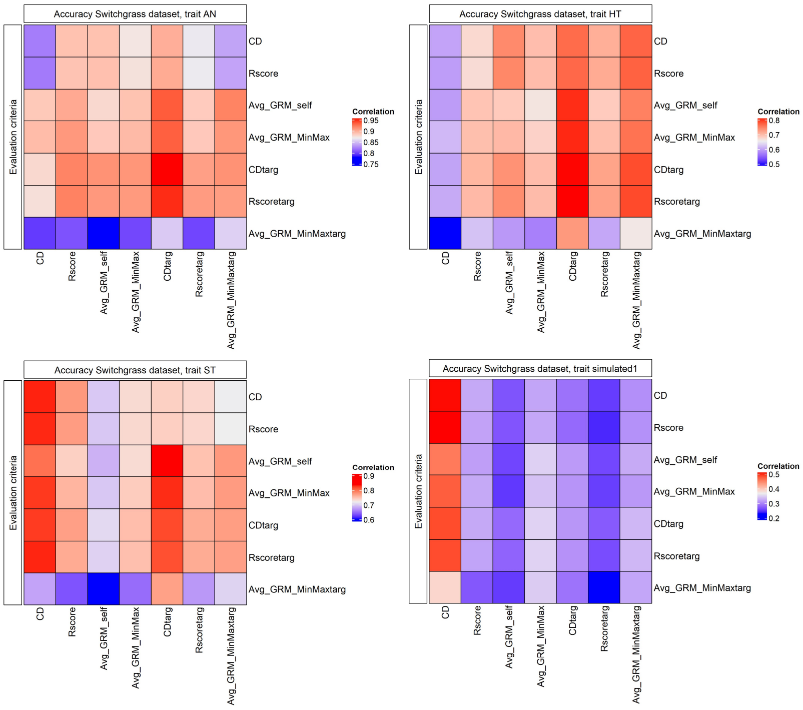


**Fig. S36** Average correlation between the accuracy and the evaluation metrics across the tested training set sizes (10, 20, 40, 60 and 80% of the candidate set) for all traits in switchgrass dataset. The horizontal axis contains the optimization methods used to obtain the different training sets. For each training set the evaluation metrics in the vertical axis were computed and were correlated with the accuracy obtained using GBLUP model.
